# Optimal dosage of exercise combined with intermittent fasting for body composition and cardiometabolic health in adults: a systematic review and multilevel meta-analysis

**DOI:** 10.3389/fnut.2026.1772836

**Published:** 2026-03-10

**Authors:** Mingyue Jiao, Henghao Yan, Binbin Zhang, Xiaohui Zhao, Jian Li, Mohd Taib Harun

**Affiliations:** 1 School of Teacher Education, Hezhou University, Hezhou, Guangxi, China; 2 School of Physical Education, Southwest University, Chongqing, China; 3 Faculty of Sports and Leisure, Guangdong Ocean University, Zhanjiang, Guangdong, China; 4 Faculty of Education and Liberal Arts, INTI International University, Nilai, Negeri Sembilan, Malaysia; 5 Shanxi Institute of Science and Technology, Jincheng, Shanxi, China

**Keywords:** body composition, cardiometabolic health, exercise, intermittent fasting, meta analysis

## Abstract

**Purpose:**

This meta-analysis evaluated the effects of exercise combined with intermittent fasting (EX + IF) on body composition, cardiometabolic health, and muscle performance in adults and examined potential moderators. (2) PubMed, Web of Science, Embase, and the Cochrane Library were searched, and reference lists of eligible studies were screened. Effect sizes were calculated as Hedges’*g*. A three-level random-effects model was fitted using the metafor package in R, with moderation and meta-regression analyses conducted to identify influential factors.

**Results:**

Sixty-five randomized controlled trials (RCTs) including 3,293 participants (18–75 years) were included; 42% were overweight/obese and 11% were trained individuals. Compared with control conditions (exercise alone, intermittent fasting alone, or neither), EX + IF significantly reduced body mass, body mass index, body fat percentage, fat mass, waist circumference, and visceral fat, with no significant effects on fat-free mass or lean body mass. For cardiometabolic outcomes, EX + IF reduced total cholesterol, triglycerides, low-density lipoprotein cholesterol, and interleukin-6; improved fasting blood glucose, fasting insulin, and homeostasis model assessment of insulin resistance (HOMA-IR); and modestly increased VO_2_max. Multivariable meta-regression indicated maximal effects with 45–60 min per session, 4 sessions/week (230–300 min/week), over 14–30 weeks (cumulative training time 7,463–8,592 min).

**Conclusion:**

EX + IF improves body composition in adults, benefits selected lipid and glycemic markers, and enhances cardiorespiratory fitness. Based on current evidence, it may be particularly suitable for middle-aged adults with overweight/obesity whose primary goals are weight loss and improved insulin resistance (45–60 min/session, 4 sessions/week, for ≥14 weeks).

**Systematic review registration:**

https://www.crd.york.ac.uk/PROSPERO/view/CRD420251131430.

## 1 Introduction

Body composition—particularly visceral adiposity and skeletal muscle mass—is a key phenotypic determinant of cardiometabolic health (1–3). Adipose tissue functions as an active endocrine organ; expansion of visceral fat is often accompanied by adipocyte hypertrophy and dysfunction, which can disrupt adiponectin–leptin signaling and activate pro-inflammatory pathways, thereby reducing insulin sensitivity and impairing vascular endothelial function (4–7). Notably, insulin resistance and related cardiometabolic diseases are multifactorial and arise from interactions among chronic hyperinsulinemia, glucosamine toxicity, lipotoxicity, genetic susceptibility, and exposure to obesogenic diets and lifestyles (4, 8, 9). Within adipose tissue, local hypoxia and lipotoxicity are major triggers of endoplasmic reticulum stress and dysregulated adipokine secretion, further amplifying systemic inflammation and metabolic dysfunction (6, 7). In addition, persistent hyperinsulinemia and glucolipotoxicity can induce pancreatic β-cell stress, dysfunction, and apoptosis, progressively diminishing insulin secretory capacity and facilitating progression to type 2 diabetes (T2D) (8–10). In contrast, skeletal muscle is the primary site of glucose disposal and contributes to systemic energy homeostasis through both its quantity and metabolic activity (2–4). When visceral fat increases while skeletal muscle declines, a pathological milieu characterized by insulin resistance, dyslipidemia, hypertension, endothelial dysfunction, and chronic low-grade inflammation emerges (4, 11–13) progression of T2D (12), atherosclerotic cardiovascular disease (ASCVD) (14), nonalcoholic fatty liver disease (2), and chronic kidney disease (5). These interrelated processes are reflected in the global disease burden: cardiovascular diseases cause nearly 18 million deaths annually (32% of all deaths worldwide) (15); approximately 500 million people have diabetes, a figure projected to reach 783 million by 2045 (16); and obesity—a shared upstream driver—already affects more than 1 billion people and continues to increase (17). Accordingly, identifying safe, effective, and sustainable lifestyle interventions that improve body composition and cardiometabolic risk markers is of urgent practical importance and substantial societal value.

Intermittent fasting (IF) is an increasingly studied dietary strategy for weight management and improved metabolic health (18). Consistent with international consensus terminology, IF refers to dietary patterns that alternate between recurring periods of fasting (complete or modified, i.e., minimal energy intake) and periods of eating (19). Notably, eating periods are not necessarily ad libitum; depending on the protocol and study design, intake may be ad libitum within a restricted time window [time-restricted eating (TRE)] or structured around prespecified energy targets and diet-quality recommendations (19, 20). IF is conceptually distinct from continuous daily energy restriction, in which energy intake is reduced every day (19). Common IF regimens include TRE (e.g., the 16:8 schedule), alternate-day fasting (ADF), and the 5:2 diet (habitual intake on 5 days per week and substantial energy restriction on 2 days) (19). Because fasting-based regimens may require individualization to ensure safety and nutritional adequacy, implementation—particularly among adults with cardiometabolic disease and/or those using glucose-lowering therapy—should ideally occur under qualified healthcare guidance and within an overall nutrient-dense dietary pattern rich in vegetables, fruits, and legumes and containing adequate protein (21–23). Meta-analyses indicate that IF can reduce body weight and visceral fat, improve lipid profiles, lower blood pressure, and decrease concentrations of inflammatory markers such as tumor necrosis factor-*α* (TNF-α) (24–28). Evidence further suggests that IF enhances insulin sensitivity, promotes fat oxidation, and lowers fasting glucose and insulin concentrations (29–32); proposed upstream mechanisms include autophagy induction and modulation of the gut microbiota (20). Collectively, these effects may improve metabolic flexibility. Nevertheless, prolonged fasting intervals may adversely affect skeletal muscle mass.

Meta-analyses indicate that exercise (EX) effectively reduces body fat and helps preserve lean mass during weight loss (33). EX also improves metabolic health, including insulin sensitivity and blood lipid profiles (34), lowers blood pressure (35), enhances vascular function (36), and reduces inflammation (37). However, the weight-loss effects of exercise alone are often constrained by overall energy balance, which can limit sustained and clinically meaningful reductions in body fat. Consequently, this limitation has prompted researchers to investigate whether combining exercise with dietary strategies yields synergistic benefits.

Compared with exercise or dietary intervention alone, EX + IF is hypothesized to yield superior improvements in body composition and cardiometabolic health (38, 39). Exercising in the fasted state may accelerate fatty acid mobilization and oxidation, thereby reducing fat mass (40). In parallel, regular exercise may partially offset the muscle protein breakdown associated with IF and support the maintenance—or even increase—of lean mass, which is essential for long-term metabolic homeostasis and physical function (41). At the molecular and cellular levels, EX + IF has been reported to improve metabolic flexibility, promote mitochondrial biogenesis, activate autophagy–lysosomal pathways, and modulate hormonal adaptation, potentially producing synergistic effects beyond those of either intervention alone (42, 43).

Several systematic reviews and meta-analyses have examined IF combined with exercise, with particular attention to TRE alongside aerobic and/or resistance training. Overall, these syntheses report reductions in body weight and adiposity, with largely neutral effects on lean mass; however, findings for cardiometabolic biomarkers are inconsistent across studies (44, 45). In a broader meta-analysis pooling multiple IF regimens combined with EX, improvements were observed in body weight, low-density lipoprotein (LDL), and systolic blood pressure, whereas effects on total cholesterol (TC), triglycerides (TG), and high-density lipoprotein cholesterol were null (46). Although these findings are encouraging, they differ from more recent EX + IF trials that have reported improvements in other lipid-related outcomes (47, 48). Such discrepancies may reflect heterogeneity in participant characteristics (e.g., age and sex) and intervention protocols (e.g., IF regimen, exercise modality, and intervention duration) across studies. In addition, the meta-analysis was constrained by the limited evidence base available at the time (14 studies) and did not adequately address potential confounding, publication bias, or the statistical dependence among multiple effect sizes derived from the same study (e.g., selecting a single estimate from several correlated indicators), which may have reduced power and narrowed the scope of inference (49). Overall, the cardiometabolic effects of EX + IF remain uncertain, given small sample sizes, methodological limitations, and substantial between-study variability. Moreover, limited investigation of intervention dose and key EX prescription variables further constrains clinical translation. Accordingly, rigorously conducted, adequately powered meta-analyses are needed to synthesize the expanding literature, identify sources of heterogeneity, and inform more targeted, evidence-based intervention programs for diverse adult populations.

This study synthesizes evidence from 65 randomized controlled trials (RCTs) using a three-level meta-analysis to evaluate the effects of EX + IF on body composition [e.g., body mass, body mass index (BMI), body fat percentage, fat mass, waist circumference, visceral fat, fat-free mass (FFM), and lean body mass (LBM)], cardiometabolic health (e.g., blood pressure, glycemic outcomes, lipid profiles, and inflammatory markers), and muscle performance. Outcomes are compared across relevant control conditions, including EX alone, IF alone, and neither intervention. The study further examines potential moderators by assessing whether participant characteristics and intervention parameters influence effect estimates. Collectively, these findings may inform evidence-based clinical decision-making and the development of tailored EX prescriptions to maximize intervention effectiveness.

## 2 Methods and materials

This review was conducted in accordance with the preferred reporting items for systematic reviews and meta-analyses (PRISMA) 2020 statement (50). The full PRISMA 2020 checklist is provided in Supplementary Table 1. The review protocol was prospectively registered in PROSPERO in September 2025 (ID: CRD420251131430).

### 2.1 Information sources

Two researchers (MJ and HY) independently reviewed the screening process and verified the results for the studies included in this review. We searched PubMed, Web of Science, Embase, and the Cochrane Library from inception to September 2025.

### 2.2 Search strategy

The search strategy was adapted from a previous review of comparable interventions. The search query was: (“time-restricted feeding” OR “time restricted feeding” OR “time-restricted eating” OR “time restricted eating” OR “time-restricted diet” OR “time restricted diet” OR “time-restricted fasting” OR “time fast restricted” OR “intermittent fasting” OR “intermittent energy restriction” OR “alternate fasting” OR “periodic fasting” OR “reduced meal frequency” OR “alternate-day fasting”) and (exercise OR “exercise training” OR “physical activity” OR “aerobic training” OR “aerobic exercise” OR “resistance training” OR “resistance exercise” OR “combined training” OR “combined exercise” OR “concurrent training” OR “concurrent exercise” OR “interval training” OR “interval exercise”). In addition, PROSPERO and the Cochrane Database of Systematic Reviews were searched to determine whether relevant systematic review protocols or reviews had been registered or published. The search dates and results for each database are reported in Supplementary Table 2.

### 2.3 Selection process

An independent reviewer (MJ) manually removed duplicate records using EndNote X9. The deduplicated records were then exported and assessed independently by two researchers (MJ and HY), who screened titles and abstracts against predefined inclusion and exclusion criteria. Disagreements were resolved by a third reviewer (JL), who adjudicated eligibility. Next, MJ and HY independently evaluated the full texts of potentially eligible articles to determine the final set of included studies. For included reports with insufficient data for detailed analysis, we contacted the corresponding authors to request additional information.

### 2.4 Eligibility criteria

Eligibility criteria were defined using the population, intervention, comparison, outcomes, and study design (PICOS) framework. Population: adults aged ≥18 years were eligible, regardless of biological sex; no restrictions were placed on participants’ health status. Intervention: Studies evaluating EX + IF interventions lasting ≥2 weeks were included. No restrictions were imposed on exercise modality, intensity, frequency, or session duration. IF protocols included ADF, TRE, the 5:2 diet, and Ramadan intermittent fasting (RIF). Comparison: Eligible control conditions comprised exercise alone, IF alone, no EX and no IF. Outcomes: Studies were included if they reported at least one of the following: body composition (body mass, BMI, body fat percentage, fat mass, waist circumference, visceral fat, FFM, LBM); glycaemic markers (fasting blood glucose, fasting insulin, HOMA-IR); lipid profile (TC, low density lipoprotein, TG, high density lipoprotein); cardiovascular measures [systolic/diastolic blood pressure, resting heart rate, cardiorespiratory fitness (VO_2_max)]; adipokines/inflammatory markers [leptin, adiponectin, C-reactive protein (CRP), IL-6, TNF-α]; and muscle performance (grip strength, bench press, leg press, jump height). Study design: Only randomized controlled trials were included. Additional criteria were peer-reviewed, English-language publications. Acute studies, commentaries, opinion pieces, validation studies, books, and case studies were excluded.

### 2.5 Data extraction

Data were extracted independently by two reviewers (MJ and HY) using a custom Microsoft Excel worksheet (version 16.93; Microsoft Corp., Redmond, WA, United States) that was developed before full-text screening. The reviewers extracted information on study authorship, study characteristics, participant demographics, IF and training protocols, and outcome measures. When data were reported only in graphical format, values were extracted using WebPlotDigitizer (version 4.1) (51). Studies for which complete information could not be obtained were excluded from the analysis.

### 2.6 Risk of bias assessment

The Risk of Bias Tool 2 (RoB2) recommended by the Cochrane Collaboration (52) was used. The tool assesses the following areas: random sequence generation, random assignment concealment, outcome assessment blinding, outcome data integrity, and outcome reporting selectivity. If there were disagreements among the reviewers, we were resolved through discussion first. If a consensus could not be reached after discussion, a third reviewer (JL) arbitrated.

### 2.7 Evidence certainty assessment

Carey et al. (53) argued that assessing the overall quality of evidence requires evaluating the validity of individual study findings. Therefore, the quality of evidence in this meta-analysis was assessed using the recommendation grading, evaluation, development and evaluation (GRADE) system, which consists of five criteria (54): limitations, inconsistencies, indirectness, imprecision, and publication bias. Each criterion was rated on the following severity levels: not serious (no downgrade), serious (downgraded by one level), and very serious (downgraded by two levels). The overall quality of evidence was divided into four levels: high, medium, low, and very low, and the results were presented in the form of an evidence summary table. The GRADE assessment was completed by one reviewer (MJ) and reviewed by a second reviewer (HY).

### 2.8 Statistical analysis

#### 2.8.1 Data synthesis and effect measures

In this study, between-group comparisons were conducted between EX + IF group and the control group. The mean change and its standard deviation were calculated using the formulas below. First, the mean change (MD) from pre- to post-intervention was computed as follows (55): ${\mathrm{MD}}_{\text{diff}}=M_{\text{post}}-M_{\mathrm{pre}}$ , where *M*_post_ and *M*_pre_ denote the reported post-intervention and pre-intervention means, respectively.

The standard deviation (SD) of the pre-post change was calculated using the following formula (56):


$${\mathrm{SD}}_{\text{diff}}=\sqrt{{{\mathrm{SD}}_{\mathrm{pre}}}^{2}+{{\mathrm{SD}}_{\text{post}}}^{2}-2r\times {\mathrm{SD}}_{\mathrm{pre}}\times {\mathrm{SD}}_{\text{post}}}$$

To enable pooling of outcomes assessed using different measurement techniques or reported in different units across studies (e.g., mg/dL vs. mmol/L for glycaemic and lipid markers), we used the standardized mean difference (SMD) as the effect size, in accordance with Cochrane guidance (50). Because several included studies had small sample sizes, we applied ${\text{Hedges}}^{’}\;g$ (a small-sample bias-corrected SMD) (57). ${\text{Hedges}}^{’}\;g$ was calculated as: ${\text{Hedges}}^{’}\;g=\frac{\left(M_{e\_\text{post}}-M_{e\_\mathrm{pre}})-(M_{c\_\text{post}}-M_{c\_\mathrm{pre}}\right)}{{\mathrm{SD}}_{\text{pooled}\_\mathrm{pre}}}\;$ ×(1 $-\frac{3}{\;4\times N-9}$ ), where *M*_e_post_ and *M*_e_pre_ are the post- and pre-intervention means in the Ex + IF group, *M*_c_post_ and *M*_c_pre_ are the corresponding means in the control group, SD_pooled_pre_ is the pooled pre-intervention standard deviation across groups, and *N* is the total sample size. This approach is appropriate for estimating standardized mean differences in repeated-measures intervention studies (58). Effect sizes were interpreted using conventional thresholds: very small (0.2), small (0.2–0.5), medium (0.5–0.8) and large (>0.8) (59).

#### 2.8.2 Multi-level meta analysis and heterogeneity

Because the included EX + IF studies contributed multiple comparisons and effect sizes (*g*), we analyzed the data using a multilevel mixed-effects model (60–62). The model specified random intercepts for study, comparison type [EX + IF vs. EX, EX + IF vs. IF, and EX + IF vs. (no EX and no IF)], and effect size, with a nested structure in which effect sizes were nested within comparison types and comparison types were nested within studies. Model fit was evaluated against conventional two-level and three-level specifications, and the final model was selected using the Akaike information criterion (AIC) and Bayesian information criterion (BIC) (63). Effect sizes were weighted by the inverse of their sampling variances to account for within-study and between-study dependence and heterogeneity (64).

To further address statistical dependence, we used cluster-robust variance estimation (CRVE) based on the variance–covariance matrix and applied a small-sample correction to obtain unbiased standard errors under correlated effects (65). Model parameters were estimated using restricted maximum likelihood (REML) and sensitivity-checked using maximum likelihood (ML) to assess the robustness of the results (63). All three-level meta-analyses were conducted in R (version 4.4.0) using the metafor package (57), and CRVE with small-sample correction was implemented using the clubSandwich package (66).

Statistical heterogeneity was evaluated using the *I*^2^ statistic, with values of 25, 50, and 75% conventionally interpreted as low, moderate, and high heterogeneity, respectively. Prediction intervals (PIs) were also calculated to quantify the expected range of effects in comparable future studies. In addition, the statistical power of the primary pooled effect was estimated to evaluate the risk of false-negative findings arising from insufficient power (67).

#### 2.8.3 Subgroup and meta-regression analysis

To investigate potential sources of heterogeneity and moderator effects, we conducted subgroup analyses and meta-regression for continuous moderators (68). As recommended, meta-regression was performed only when ≥10 studies were available per model, and subgroup analyses were conducted only when each subgroup contained ≥5 studies (69). Subgroups were defined by: (i) sex; (ii) age; (iii) obesity status; (iv) comparator type (exercise alone, intermittent fasting alone, or neither exercise nor fasting); (v) training modality [high-intensity interval training (HIIT), moderate-intensity aerobic exercise [AE(M)], moderate-to-vigorous aerobic exercise [AE(MV)], resistance training (RT), or concurrent training]; (vi) IF regimen (TRE, 5:2, ADF, RIF, or IER); and (iv) training status (athletes, active/trained, or normal adults). Obesity status was classified using BMI categories, with participants categorized as non-obese or obese (overweight: BMI 25–29.9 kg/m^2^ and/or obesity: BMI ≥30 kg/m^2^) (70). Following Chen et al. (70), exercise intensity was classified as AE(M): 45–65% VO_2_max, >50–65% heart-rate reserve (HRR), or 65–75% maximum heart rate (MHR); AE(MV): moderate-to-vigorous intensity; RT: ≥50% one-repetition maximum (1RM); and HIIT: >65% VO_2_max, >65% HRR, or >75% MHR during high-intensity intervals.

Cubic spline models were used in multivariable meta-regression to examine potential nonlinear associations between study-level predictors and effect sizes, with study identifiers specified as random effects to account for dependence among estimates. Predictors captured participant characteristics (age) and exercise and intermittent fasting prescription variables, including: (i) training frequency; (ii) total number of sessions; (iii) total weekly exercise duration; (iv) total intervention duration; and (v) intervention length (weeks). We fitted cubic spline models with 3, 4, and 5 knots and compared model fit using likelihood ratio tests to identify the optimal specification (71).

#### 2.8.4 Risk of publication bias and sensitivity analysis

Publication bias was assessed using contour-enhanced funnel plots (72) and Egger’s regression test (73), which was conducted only when at least 10 studies were available (*k* ≥ 10) (74). A *p*-value >0.05 was interpreted as indicating no statistically significant evidence of small-study effects. Funnel plots and Egger’s test evaluate asymmetry in the distribution of effect sizes using visual inspection and regression-based quantification, respectively; such asymmetry may suggest publication bias or other sources of small-study effects.

Sensitivity analyses were performed using a leave-one-out approach, whereby each study was sequentially removed to evaluate its influence on the overall pooled effect.

## 3 Results

### 3.1 Studies retrieved

**Figure 1:**
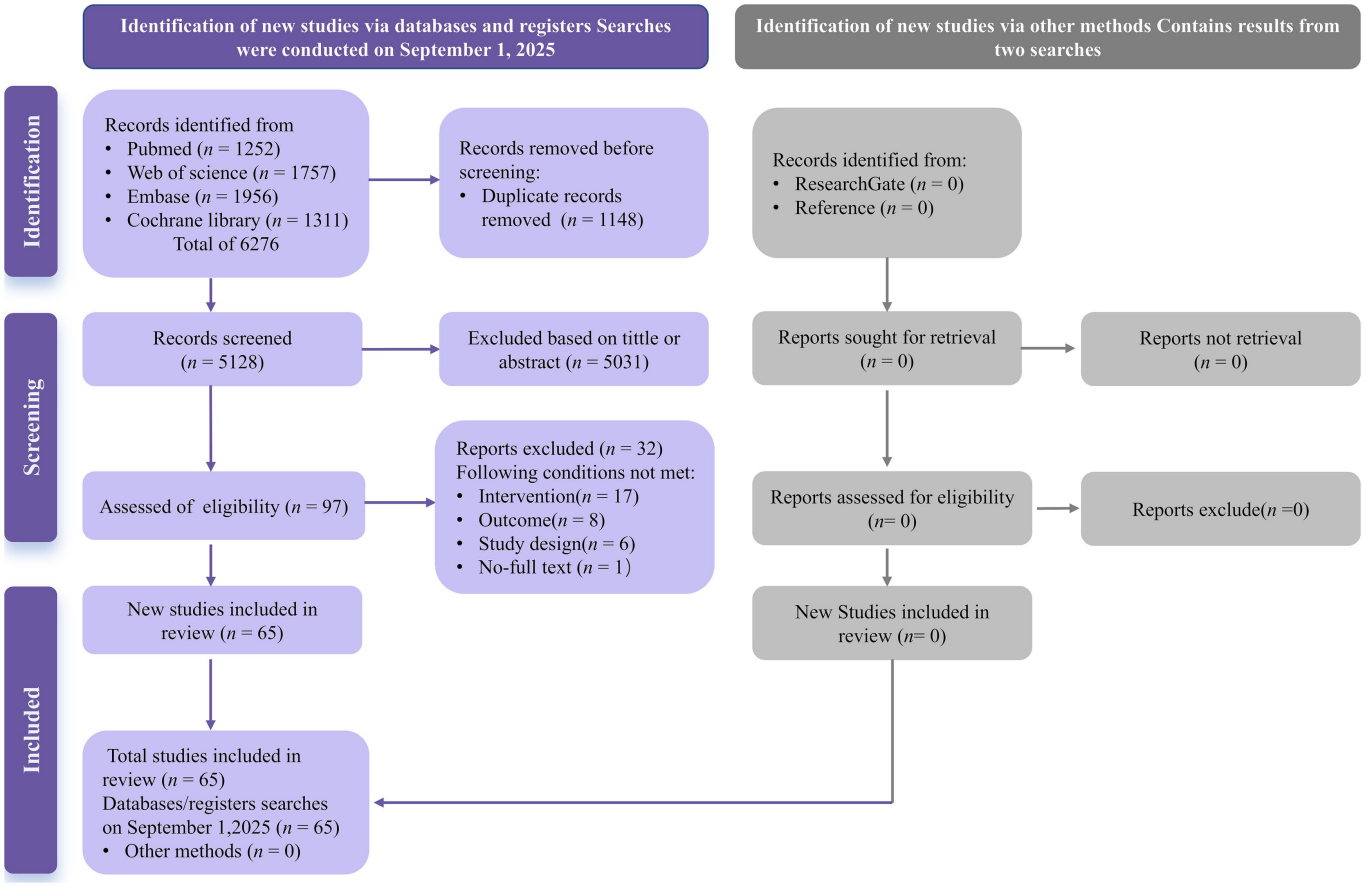
PRISMA flow diagram depicting the study selection.

The initial search (from database inception to September 2025) identified 6,276 records that met the inclusion criteria. After removing 1,148 duplicates, 97 records were retained for full-text assessment based on their titles and abstracts. Ultimately, 65 studies (47, 48, 75–137) met the inclusion criteria (Figure 1).

### 3.2 Characteristics of included studies

The study included 3,293 participants (1,172 men and 2,081 women) aged 18–75 years. Overall, 42% of participants were classified as overweight or obese, 47% as non-overweight/non-obese, and 11% were active/trained individuals, including athletes. Among the 65 RCTs included, exercise modalities in EX + IF group comprised aerobic training [*n* = 23, 35.4%; primarily [AE(M)], *n* = 20], resistance training (*n* = 19, 29.2%), HIIT (*n* = 13, 20.0%), and combined training (*n* = 10, 15.4%). Most interventions incorporated at least moderate-intensity exercise. IF regimens were primarily TRE (*n* = 30, 46.2%) and RIF (*n* = 14, 21.5%), followed by other IER (*n* = 8, 12.3%), the 5:2 diet (*n* = 7, 10.8%), and ADF (*n* = 6, 9.2%). Additional details on participant characteristics and the intervention protocol are provided in Supplementary Tables 3, 6.

### 3.3 Main effects

#### 3.3.1 Effects of EX + IF on body composition compared to the control group

**Figure 2:**
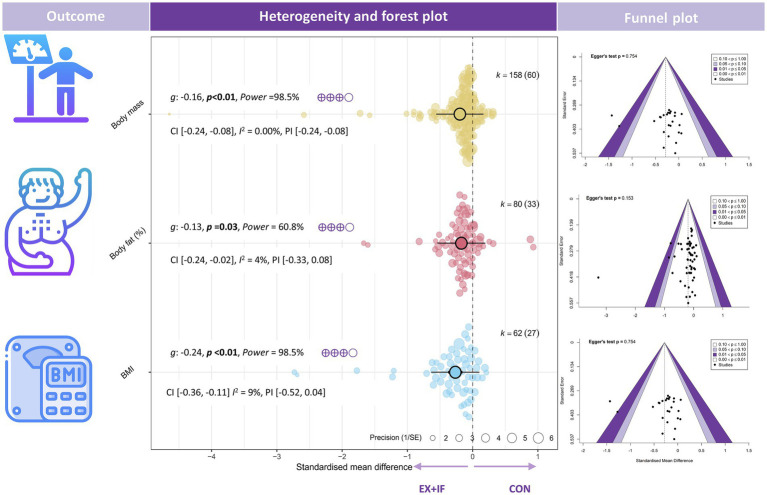
Effects and funnel plot of EX + IF on body composition. Hedge’s *g*: the effect size indicators used in the pooled; *I*^2^: quantitative indicators of heterogeneity; *K*: the total number of effects included in the pooled effect size; *p*-value: statistically significant *p*-values for pooled results; power: statistical power for pooled effect size. 95% CI, 95% confidence interval; GRADE, grading of recommendations assessment, development, and evaluation (a system for evaluating the quality of evidence and strength of recommendations); BMI, body mass index; EX + IF, exercise combined with intermittent fasting.

**Figure 3:**
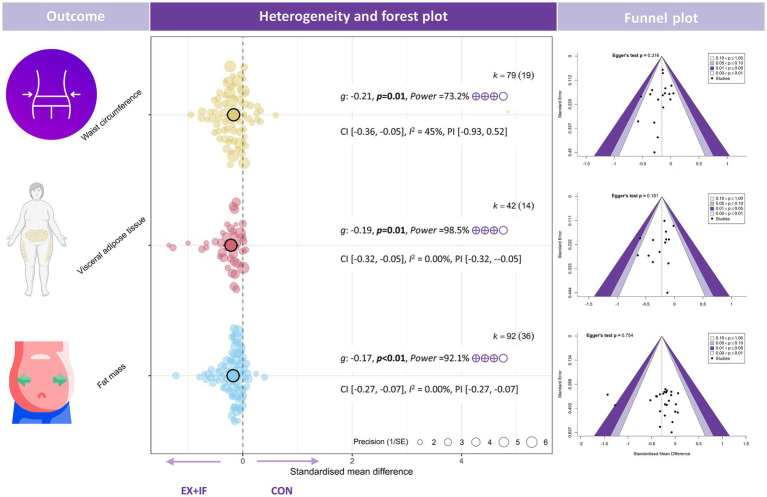
Effects and funnel plot of EX + IF on body composition.

Regarding body composition, EX + IF was associated with improvements versus the control group across multiple outcomes: body mass [*k* = 158, *g* = −0.16, 95% CI (−0.24, −0.08), *I*^2^ = 0% (low), PI (−0.24, −0.08), *p* < 0.01; very small effect; moderate GRADE], BMI [*k* = 62, *g* = −0.24, 95% CI (−0.36, −0.11), *I*^2^ = 9% (low), PI (−0.52, 0.04), *p* < 0.01; small effect; low GRADE], body fat (%) [*k* = 80, *g* = −0.13, 95% CI (−0.24, −0.02), *I*^2^ = 4% (low), PI (−0.33, 0.08), *p* = 0.03; very small effect; moderate GRADE], fat mass [*k* = 92, *g* = −0.17, 95% CI (−0.27, −0.07), *I*^2^ = 0% (low), PI (−0.27, −0.07), *p* < 0.01; very small effect; moderate GRADE], waist circumference [*k* = 79, *g* = −0.21, 95% CI (−0.36, 0.05), *I*^2^ = 45% (moderate), PI (−0.93, 0.52), *p* = 0.01; small effect; moderate GRADE], and visceral adipose tissue [*k* = 42, *g* = −0.19, 95% CI (−0.32, −0.05), *I*^2^ = 0% (low), PI (−0.32, −0.05), *p* = 0.01; very small effect; moderate GRADE] (Figures 2, 3). In contrast, EX + IF did not significantly affect FFM or LBM relative to the control group (Supplementary Table 4).

#### 3.3.2 Effects of EX + IF and control group on blood lipids

**Figure 4:**
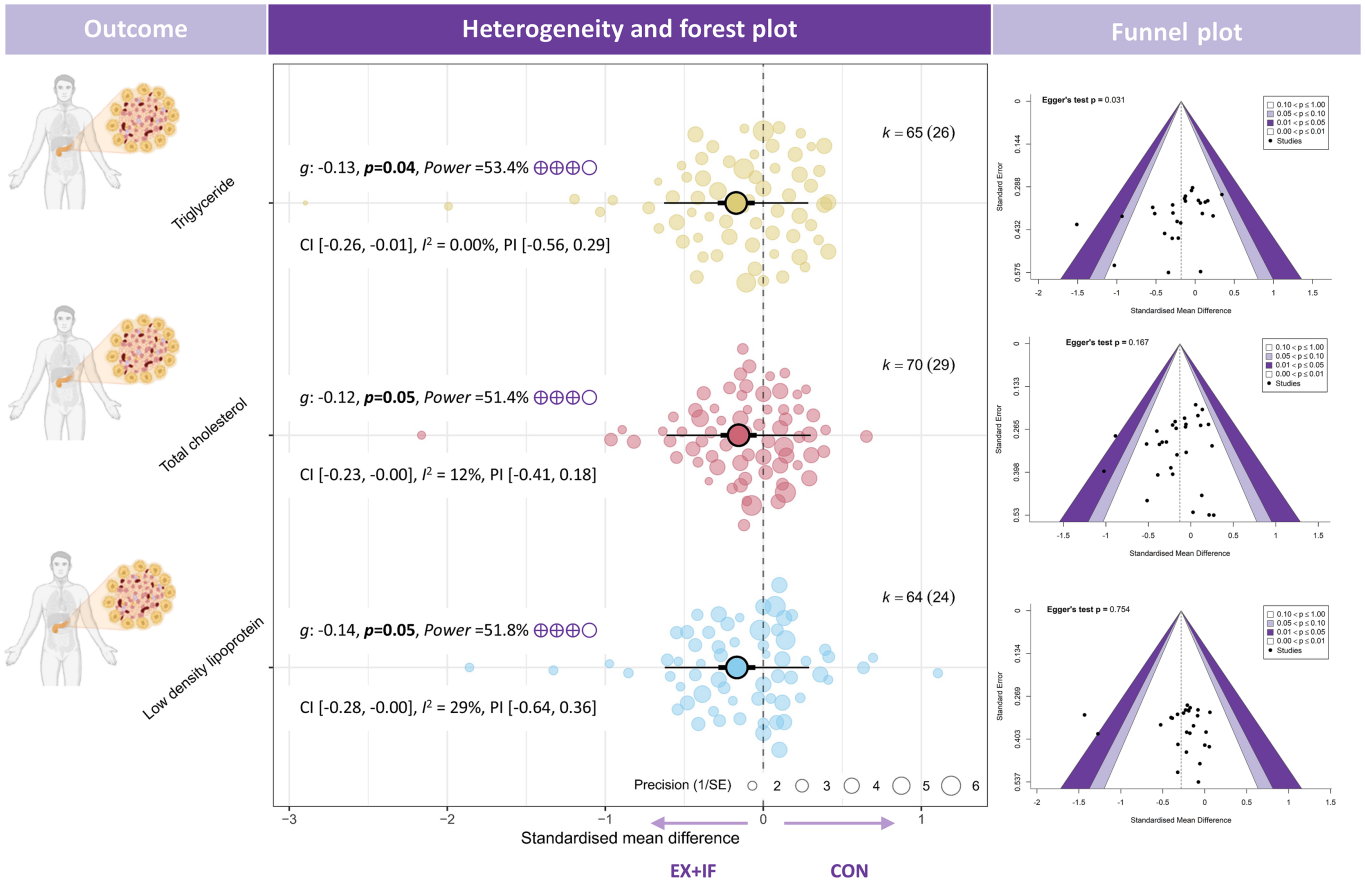
Effects and funnel plot of EX + IF on lipid profiles.

Regarding lipid outcomes, the meta-analysis reported small reductions in TC [*k* = 70; *g* = −0.12; 95% CI, −0.23 to −0.00; *I*^2^ = 12% (low); PI, −0.41 to 0.18; *p* = 0.05; very small effect; GRADE: moderate], TG [*k* = 65; *g* = −0.13; 95% CI, −0.26 to −0.01; *I*^2^ = 22% (low); PI, −0.56 to 0.29; *p* = 0.04; very small effect; GRADE: moderate], and LDL [*k* = 64; *g* = −0.14; 95% CI, −0.28 to −0.00; *I*^2^ = 29% (moderate); PI, −0.64 to 0.36; *p* = 0.05; very small effect; GRADE: moderate] (Figure 4). By contrast, no significant difference was observed for high-density lipoprotein cholesterol between the EX + IF and control groups (Supplementary Table 4).

#### 3.3.3 Effects of EX + IF and control group on blood glucose

**Figure 5:**
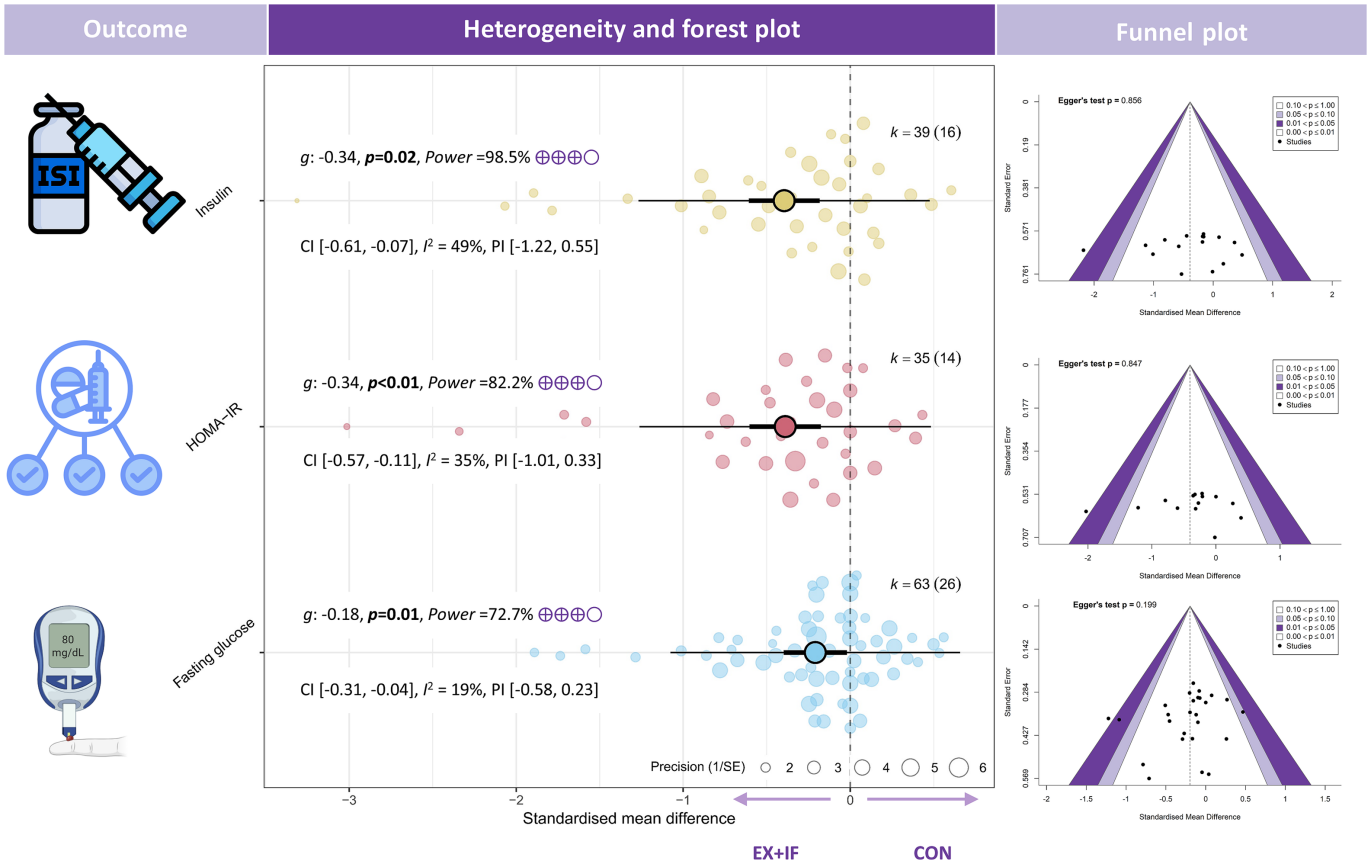
Effects and funnel plot of EX + IF on glycemic outcomes.

Regarding glycemic outcomes, the meta-analysis reported reductions in fasting blood glucose [*k* = 63; *g* = −0.18; 95% CI, −0.31 to −0.04; *I*^2^ = 19% (low); PI, −0.58 to 0.23; *p* = 0.01; very small effect; GRADE: moderate], insulin [*k* = 39; *g* = −0.34; 95% CI, −0.61 to −0.07; *I*^2^ = 49% (moderate); PI, −1.22 to 0.55; *p* = 0.02; small effect; GRADE: moderate], and HOMA-IR [*k* = 35; *g* = −0.34; 95% CI, −0.57 to −0.11; *I*^2^ = 35% (moderate); PI, −1.01 to 0.33; *p* < 0.01; small effect; GRADE: moderate] (Figure 5). By contrast, no significant difference was observed in HbA1c between the EX + IF and control groups (Supplementary Table 4).

#### 3.3.4 Effects of EX + IF versus control group on blood pressure and CRF

**Figure 6:**
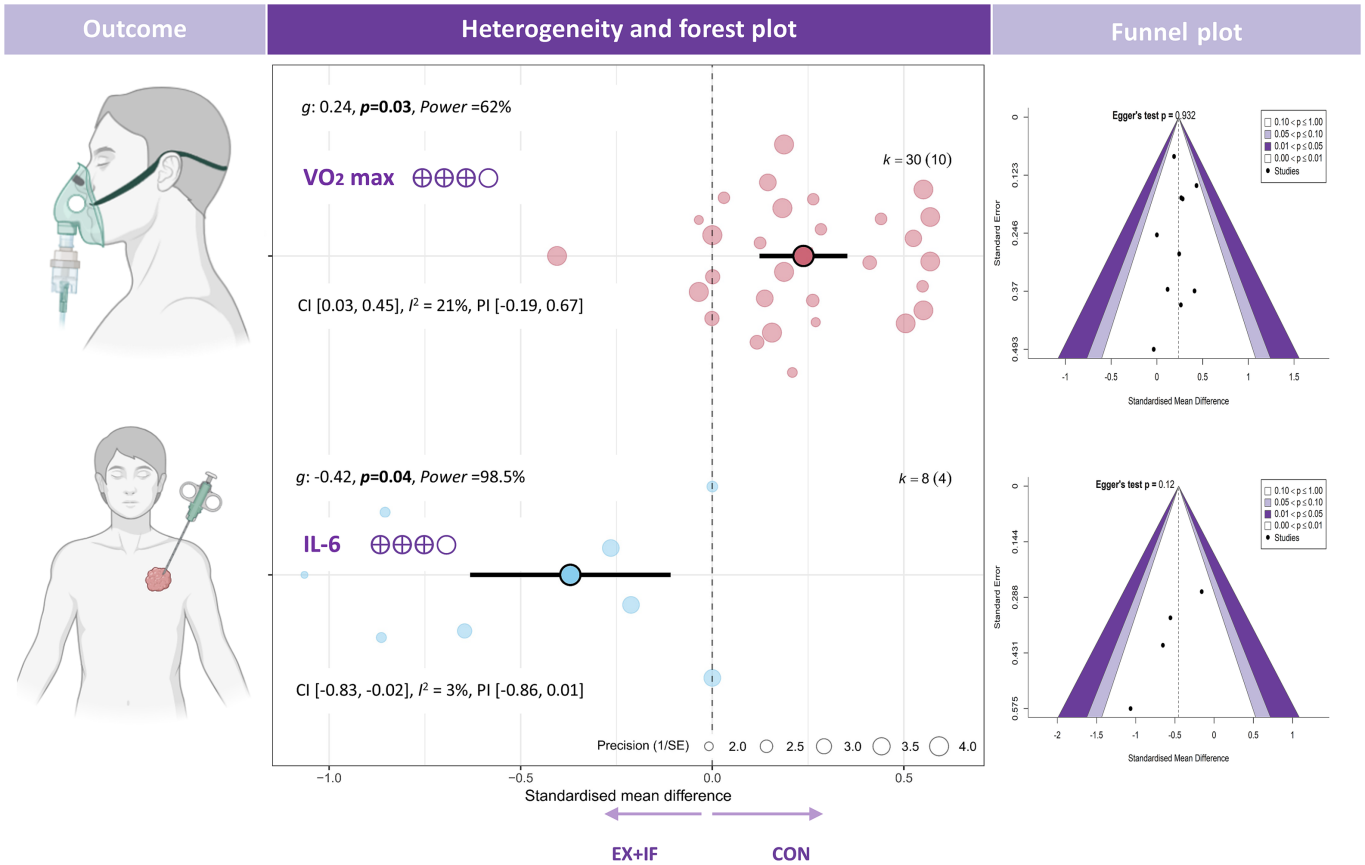
Effects and funnel plot of EX + IF on CRF and IL-6.

Regarding blood pressure and cardiorespiratory fitness (CRF), the meta-analysis reported a small improvement in VO_2_max [*k* = 30; *g* = 0.24; 95% CI, 0.03 to 0.45; *I*^2^ = 21% (low); PI, −0.19 to 0.67; *p* = 0.03; small effect; GRADE: moderate] (Figure 6). By contrast, no significant differences were observed for systolic blood pressure, diastolic blood pressure, or heart rate between the EX + IF and control groups (Supplementary Table 4).

#### 3.3.5 Effects of EX + IF and control group on adipokines

Regarding adipokine-related outcomes, the meta-analysis reported a reduction in interleukin-6 [IL-6; *k* = 30; *g* = −0.42; 95% CI, −0.83 to −0.02; *I*^2^ = 3% (low); PI, −0.86 to 0.01; *p* = 0.04; small effect; GRADE: moderate] (Figure 6). By contrast, no significant differences were observed for adiponectin, leptin, C-reactive protein (CRP), or tumor necrosis factor-α (TNF-α) between the EX + IF and control groups (Supplementary Table 4).

#### 3.3.6 Effects of EX + IF on muscle performance compared to the control group

The meta-analysis of muscle performance found no significant differences between the EX + IF and control groups across four outcomes: handgrip strength, bench press, leg press, and jump height (Supplementary Table 4).

Detailed merged forest plots for each outcome are presented in Supplementary Figure 1. In addition, a visualization of the statistical power for the pooled results across all outcomes is provided in Supplementary Figure 2.

### 3.4 Subgroup analysis

**Figure 7:**
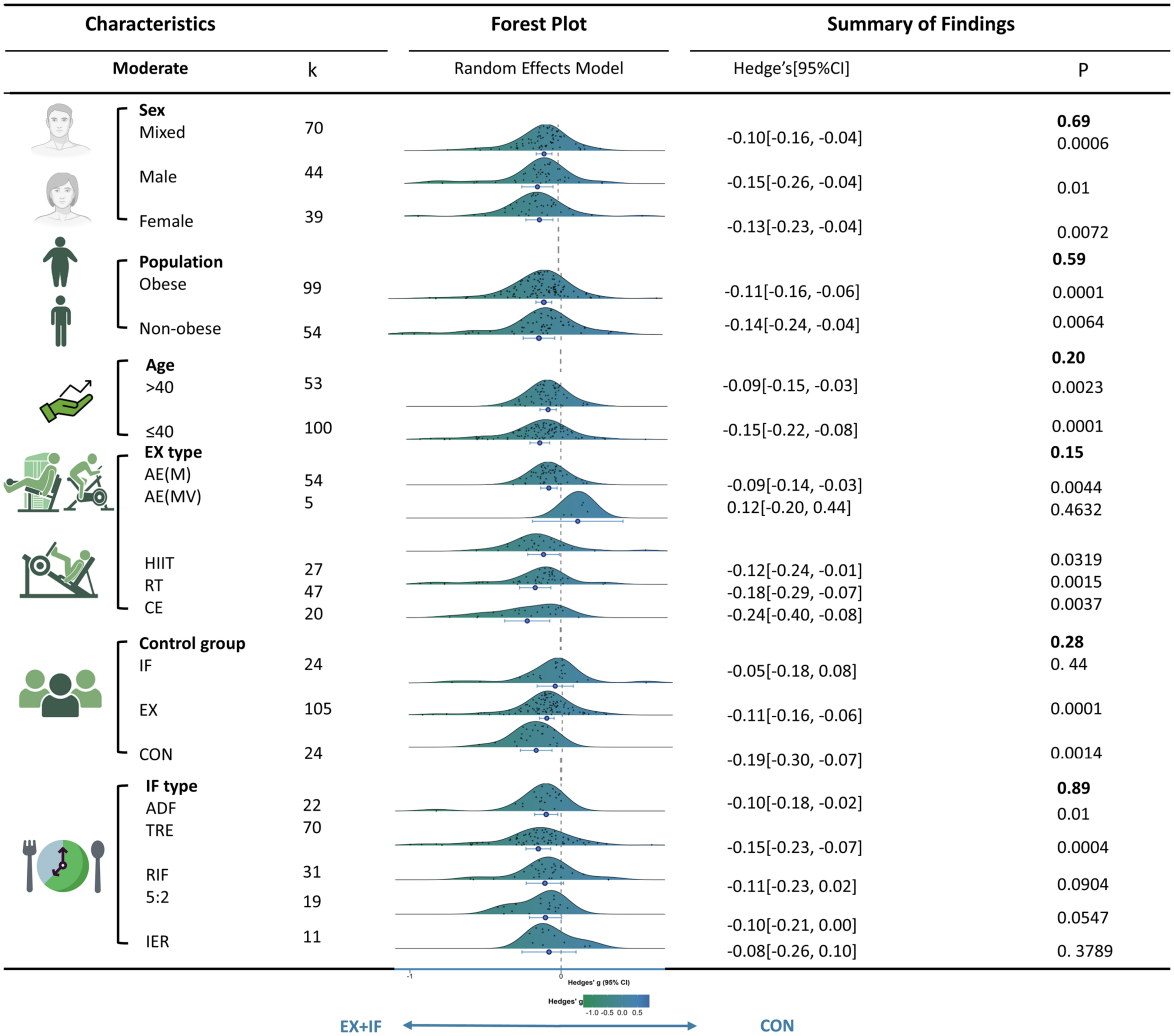
Subgroup analyses based on body mass.

This study performed moderator analyses to examine whether sex, obesity status, age, exercise modality, control group type, or IF regimen moderated the effects of EX + IF on body mass (Figure 7) and other outcomes (Supplementary Table 5). For body mass, subgroup analyses indicated no significant differences in effect estimates between subgroups (*p* > 0.05; Figure 7 and Supplementary Table 7), suggesting that these candidate moderators did not materially influence the overall intervention effect. Nevertheless, EX + IF was associated with significant reductions in body mass in several prespecified subgroups compared with controls. Specifically, significant improvements were observed in men, women, and mixed-sex samples; across all obesity categories; in participants aged ≤40 and >40 years; and for aerobic exercise (moderate intensity), high-intensity interval training, resistance training, and concurrent training. Significant effects were also observed when the comparator was exercise alone and when both exercise and IF were absent. By IF regimen, significant effects were observed for ADF, TRE, and the 5:2 diet. However, the number of trials differed across IF protocols (TRE, *n* = 30; RIF, *n* = 14; IER, *n* = 8; 5:2 diet, *n* = 7; ADF, *n* = 6). EX prescriptions also varied both within and across protocols; therefore, protocol-specific findings should be interpreted with caution. In the training status subgroup analysis, no significant differences were observed among athletes, active/trained individuals, and general adults (Supplementary Table 7). The seven trials conducted in athletes employed either RIF (*n* = 4) or TRE (*n* = 3) and encompassed weightlifting, bodybuilding, soccer, cycling, running, and basketball (Supplementary Table 6).

Supplementary Table 5 summarizes the subgroup analyses, reporting the SMD and corresponding 95% CI for each subgroup. Significant between-subgroup differences were identified for BMI (by control group type), FFM (by control group type), HbA1c (by sex), diastolic blood pressure (by sex, obesity status, and IF regimen), and leg press performance (by control group type). No significant subgroup differences were observed for the remaining outcomes.

### 3.5 Multivariate meta-regression

**Figure 8:**
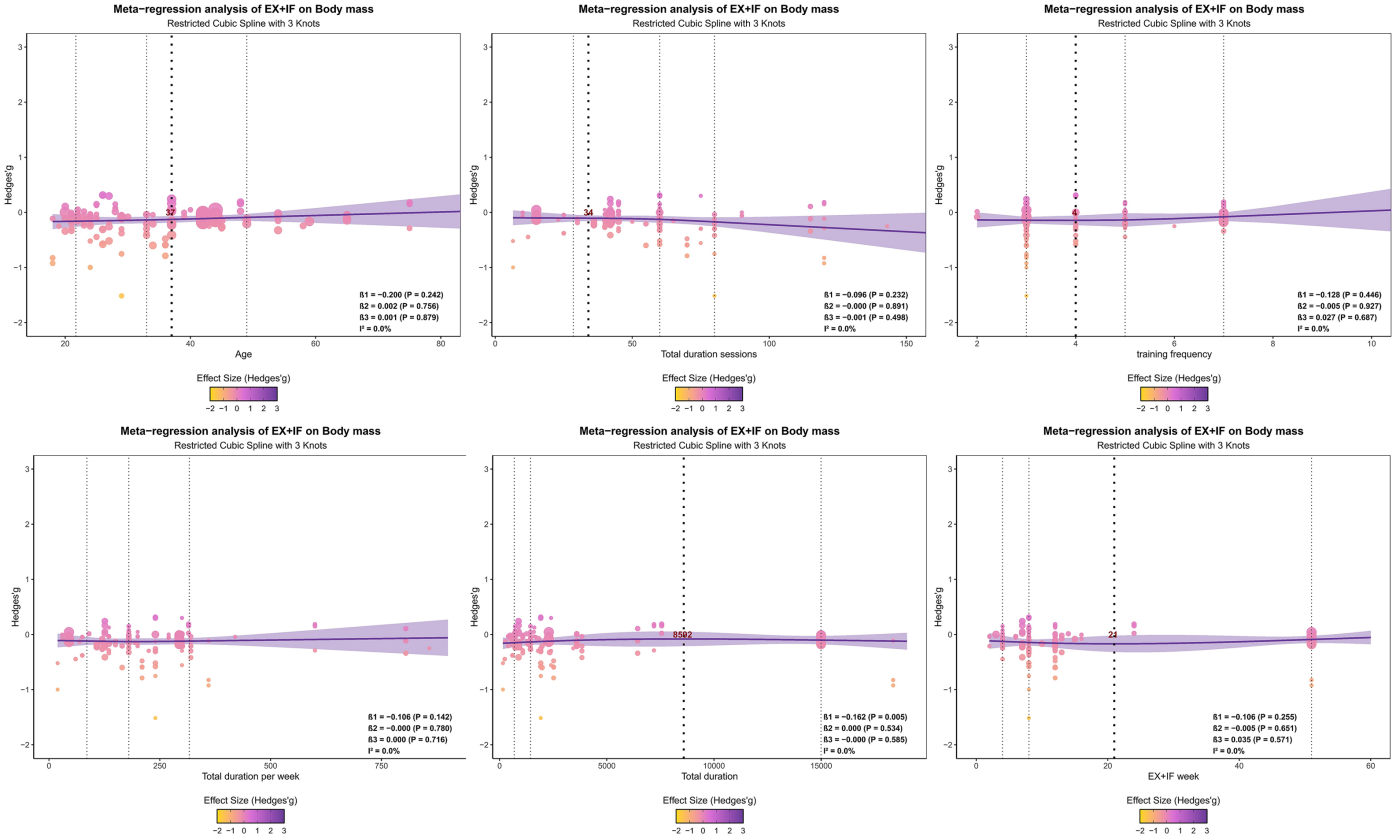
Regression analysis of body mass. *β*0 represents the intercept; *β*1, *β*2, and *β*3 represent the slopes; *I*^2^ means heterogeneity; the purple shaded part represents the 95% confidence interval.

Exploratory multivariable meta-regression indicated that total training duration significantly moderated the effect on BMI. A cubic model further suggested a statistically significant nonlinear association (*β*1 = −0.16; *I*^2^ = 0%; *p* = 0.01) (Figure 8). Curve-fitting analyses indicated that the estimated peak effect occurred at a total EX + IF duration of 8,592 min.

Supplementary Figure 3 presents the meta-regression results for additional outcomes, examining potential moderators including age, session duration, training frequency, weekly training volume, number of training weeks (EX + IF weeks), and total intervention duration. Statistically significant nonlinear associations were reported for the following outcomes: (i) body fat percentage, with estimated peak effects at a session duration of 47 min, a frequency of four sessions/week, and a weekly volume of 263 min, as well as in relation to total duration and EX + IF weeks; (ii) fat mass, with a peak at a total duration of 7,463 min; (iii) FFM, with a peak at 14 EX + IF weeks; (iv) waist circumference, with peaks by session duration and weekly volume, and additional peaks at a total duration of 7,527 min and 29 EX + IF weeks; (v) visceral adipose tissue, with the most favorable effect at age 38 years; (vi) TC, with peaks at age 38 years, a session duration of 65 min, and a weekly volume of 257 min; (vii) TG, with peaks at age 39 years and a weekly volume of 321 min, with the EX + IF regimen details not reported in the provided text; (viii) high-density lipoprotein cholesterol, with the most favorable effect at age 34 years and a peak at a session duration of 45 min; (ix) LDL, with the most favorable effect at age 36 years and a peak at a weekly volume of 278 min; (x) fasting blood glucose, with a peak at a session duration of 41 min; (xi) insulin, with a peak at a weekly volume of 308 min; (xii) diastolic blood pressure, with peaks at a session duration of 58 min, a weekly volume of 232 min, and 31 EX + IF weeks; (xiii) bench press performance, which was associated with total duration; and (xiv) leg press performance, with a peak at a session duration of 70 min. No significant associations were reported for the remaining outcomes.

### 3.6 Risk of bias

The risk-of-bias assessment indicated “some concerns” for all included studies. Using the RoB 2.0 tool, the primary sources of bias were (i) incomplete follow-up leading to missing outcome data and (ii) the absence of preregistration in most studies, which increased the risk of selective outcome reporting (Supplementary Figure 4).

Publication bias was assessed using funnel plots and Egger’s regression test for outcomes related to body composition, cardiometabolic health, and muscle performance (Supplementary Figure 5). Egger’s test suggested potential small-study effects for TG (*p* = 0.03), whereas results for the remaining outcomes were not statistically significant, providing no evidence of publication bias.

### 3.7 Sensitivity analysis

Sensitivity analyses were conducted for all major pooled effects using a leave-one-out approach (Supplementary Figure 6). Excluding individual studies materially changed the pooled estimates for some outcomes, including blood lipids and leptin.

## 4 Discussion

This systematic review and meta-analysis evaluated the effects of EX + IF on body composition, cardiometabolic health, and muscle performance. Potential moderators, including participant characteristics and intervention parameters, were examined. Compared with control conditions, EX + IF was associated with statistically significant in body composition and selected cardiometabolic outcomes.

Based on the multilevel meta-regression results of this study and prior literature, and considering both adult weight-loss goals and cardiovascular health needs, we propose a preliminary prescription range for EX + IF. Specifically, for adults whose primary goal is weight loss and fat reduction, a regimen of 45–60 min per session, approximately four sessions per week (total weekly training time ≈230–300 min), sustained for at least 14 weeks, may be appropriate. For middle-aged adults—particularly those who are overweight or obese—EX + IF may provide additional benefits by reducing body fat and improving insulin resistance.

This systematic review and multilevel meta-analysis found that, compared with control conditions, EX + IF yielded greater improvements in body composition in adults, including significant reductions in body mass, BMI, body fat percentage, fat mass, waist circumference, and visceral adipose tissue, with no significant effects on FFM or LBM. Previous systematic reviews and meta-analyses similarly indicate that adding IF to exercise can further reduce weight, BMI, fat mass, and visceral fat, and improve waist circumference relative to exercise alone or IF alone, while having limited effects on LBM. Khalafi et al. (138), based on 11 randomized controlled trials, reported that EX + IF significantly reduced weight, BMI, body fat, and visceral fat compared with exercise alone, without significantly affecting LBM, which is consistent with our findings for weight, fat mass, and visceral fat. Hays et al. (44) compared TRE + EX with the same exercise dose alone and found a significant reduction in fat mass, while FFM remained stable. Reviews of IF + RT also suggest that, under energy restriction, combined interventions generally maintain or slightly increase LBM while reducing body fat, consistent with our observation that fat mass decreased without significant LBM loss (139). In contrast, large meta-analyses of IF alone show effective reductions in weight and BMI among adults with overweight or obesity, but improvements in waist circumference and waist-to-hip ratio are often not significant (24). In our analysis, waist circumference and visceral adipose tissue decreased significantly with EX + IF, suggesting that exercise may be a key synergistic component for improving abdominal adiposity and fat distribution; this is consistent with network meta-analytic evidence that combinations such as “ADF + AE” and “IF + EX” outperform IF alone or exercise alone for weight loss and fat mass reduction (140). Notably, the effect estimates for FFM and LBM were close to zero, with confidence intervals spanning zero and very low heterogeneity, indicating that current trial evidence does not support systematic exacerbation of lean-mass loss with EX + IF. This interpretation aligns with most IF+RT trials showing that, with adequate protein intake and structured strength training, TRE does not compromise muscle mass and may even yield small increases in LBM in some studies (97). Mechanistically, EX + IF may increase net energy deficit by combining higher energy expenditure from exercise with reduced energy intake during fasting, thereby promoting whole-body and visceral fat mobilization (139); concurrently, exercise-induced mechanical loading can stimulate muscle protein synthesis and help preserve skeletal muscle, allowing FFM/LBM to be maintained under energy-restricted conditions (141–143).

Regarding lipid outcomes, a recent systematic review of EX + IF reported that TRE + AE/RT significantly reduced only TC and LDL (45). In contrast, our multilevel meta-analysis showed a consistent reduction trend in TC, TG, and LDL with EX + IF, whereas no significant between-group difference was observed for high-density lipoprotein (HDL). For glycemic outcomes, a recent meta-analysis in adults with overweight or obesity found that IF combined with exercise improved HOMA-IR and fasting insulin compared with IF alone (144). Similarly, our results indicated consistent reductions in fasting blood glucose, insulin, and HOMA-IR with EX + IF relative to control conditions. Notably, meta-analyses suggest that, under isoenergetic conditions, IF is not superior to continuous energy restriction for metabolic improvement, implying that some benefits of IF may largely reflect negative energy balance and weight loss (145). However, the present findings indicate that, across heterogeneous energy-control and weight-loss contexts, EX + IF can still improve fasting blood glucose and insulin, supporting a synergistic effect of exercise-stimulated skeletal muscle glucose uptake and IF-related metabolic reprogramming rather than a simple additive “calorie-deficit” mechanism. Mechanistically, improvements in atherosclerosis-related lipids with EX + IF may reflect both negative energy balance and fasting-induced metabolic switching: fasting lowers insulin and increases glucagon and catecholamines, shifting substrate use from glucose oxidation toward fatty-acid oxidation and ketogenesis, thereby enhancing hepatic β-oxidation and utilization of very-low-density lipoprotein triglycerides (VLDL-TG) (146). IF may also remodel hepatic fatty-acid metabolism and mitochondrial function via pathways such as AMPK–SIRT1–PPARα, reducing intrahepatic fat accumulation and VLDL production and, in turn, improving circulating TG and LDL (147, 148). In parallel, regular exercise upregulates lipoprotein lipase activity in skeletal muscle and myocardial capillary endothelium, accelerates hydrolysis and clearance of triglyceride-rich lipoproteins (e.g., VLDL and chylomicrons), and increases LDL receptor expression and cholesterol uptake, contributing to lower plasma TG and LDL (149). Finally, significant subgroup differences in HbA1c by sex suggest potential sex-specific patterns of glucose regulation. Consistent with this possibility, some randomized trials in women with overweight or obesity report that TRE combined with endurance or resistance training reduces HbA1c and improves body composition, although effect sizes vary across studies (130).

Regarding cardiorespiratory fitness (CRF), our analyses indicated that EX + IF was associated with a small improvement in CRF in adults compared with control conditions. This finding is broadly consistent with prior systematic reviews and meta-analyses. For example, Khalafi et al. (138) synthesized randomized trials in adults with overweight or obesity and reported that EX + IF significantly increased VO_2_max relative to exercise alone. In addition, a narrative review by Gabel et al. (43), drawing on 23 combined-intervention trials, suggested that EX + IF can reduce fat mass and improve selected CRF indices across diverse populations. Although our meta-analysis did not detect a statistically significant difference in diastolic blood pressure between the combined-intervention group and IF alone, subgroup analyses suggested differential responses by sex and obesity status, underscoring the need to account for these characteristics when tailoring EX + IF interventions.

Multivariable meta-regression in this study identified a significant nonlinear dose–response relationship between EX + IF effects and outcomes related to body composition and cardiometabolic health. Total training duration showed a significant nonlinear association with reductions in body mass and body fat, with peak reductions in body mass and body fat percentage at an estimated total duration of 8,592 min. These findings suggest that EX + IF benefits may plateau beyond a certain training dose, such that additional training may not yield further fat loss and could produce diminishing returns via fatigue or reduced adherence. This pattern aligns with large cohort evidence showing a steep risk reduction as non-occupational physical activity increases from very low levels to 150–300 min/week of moderate-intensity activity, followed by a plateau (150). Consistently, the World Health Organization and many national guidelines recommend 150–300 min/week of moderate-intensity or 75–150 min/week of vigorous-intensity aerobic activity for adults (150). In our analysis, the range associated with more favorable cardiometabolic outcomes (230–320 min/week over 20–30 weeks) was at or slightly above the upper end of these recommendations, suggesting that a moderate training volume near the guideline upper bound may be particularly beneficial in EX + IF programs. The estimated optimal session duration (47 min) also falls within the commonly cited 30–60 min training window and is consistent with evidence that ≥30 min of sustained moderate-intensity exercise can elicit cardiopulmonary and metabolic adaptations (150). For individuals practicing IF, exercise sessions may be scheduled before or after the eating window and could primarily comprise moderate-intensity aerobic exercise or resistance training lasting 45–60 min per session. Moreover, the strongest associations were observed at 47 min per session for body fat percentage, 7,463 min total duration for fat mass, and 14 weeks for FFM, suggesting a temporal shift from early fat-loss emphasis to later muscle preservation and functional improvement. Cardiometabolic benefits (e.g., lipids, glycemic indices, and blood pressure) were likewise concentrated within a moderate training volume (230–320 min/week) and a 20–30-week intervention period. In line with this pattern, Kazeminasab et al. (46) reported that cardiometabolic benefits tended to emerge after more than 4 weeks. Finally, the most favorable responses for several cardiometabolic indicators (e.g., visceral adipose tissue and multiple lipid measures) were observed at ages 34–39 years.

### 4.1 Study limitations and implications

To our knowledge, this review is the first to examine multiple moderators and to explore dose thresholds of EX + IF for body composition and cardiometabolic outcomes in adults, thereby offering practical guidance for implementation. Several limitations should be considered when interpreting these findings. First, the small number of studies for certain outcomes (e.g., cardiovascular indices, inflammatory markers, and muscle strength) may have limited statistical power and yielded imprecise or unstable estimates. Second, heterogeneity in exercise modalities and IF protocols across studies may complicate interpretation. Third, the included trials were dominated by young and middle-aged adults and participants with overweight or obesity, which limits generalizability to older adults, normal-weight individuals, and those with multiple comorbidities. Accordingly, future studies should evaluate different exercise prescriptions and fasting regimens across diverse populations. Further work is also needed to optimize the coordination of exercise with eating/fasting windows, combinations of exercise modalities, and protein and energy-intake strategies to support fat loss and muscle preservation, ideally integrating mechanistic considerations such as metabolic pathways and circadian biology. Furthermore, subgroup analyses by IF protocol were based on an uneven distribution of trials and may have been confounded by differences in exercise dose (e.g., modality, intensity, and weekly training volume), thereby limiting causal inference regarding whether any specific protocol is superior.

In this meta-analysis, although many findings were statistically significant, the pooled effects were generally small. In addition, although sustained weight loss of approximately 3–5% is often associated with clinically meaningful cardiometabolic improvements, our pooled results—based on SMD, heterogeneous populations, and varying control conditions—do not indicate that EX + IF consistently achieves these thresholds compared with EX alone or IF alone (151).

## 5 Conclusion

EX + IF may improve body composition, cardiometabolic health, and cardiorespiratory fitness. Based on the available evidence, EX + IF appears most appropriate for adults whose primary goals are weight loss and fat reduction, with a suggested prescription of 45–60 min per session, four sessions per week (approximately 230–300 min/week), for at least 14 weeks. For middle-aged adults, particularly those with overweight or obesity, EX + IF may represent an effective strategy to reduce adiposity and improve insulin resistance without adversely affecting LBM. Although this meta-analysis synthesizes the current literature, limitations in data availability and study quality preclude it from substituting for large, well-designed primary studies. Accordingly, future research should include adequately powered randomized controlled trials to confirm these findings.

## Data Availability

The original contributions presented in the study are included in the article/Supplementary material, further inquiries can be directed to the corresponding author.
