# Supplementary figures and images for: Optimal dosage of exercise combined with intermittent fasting for body composition and cardiometabolic health in adults: a systematic review and multilevel meta-analysis
Source: Front Nutr. 2026 Mar 10;13:1772836. doi: 10.3389/fnut.2026.1772836 (PMC13008886; doi:10.3389/fnut.2026.1772836)

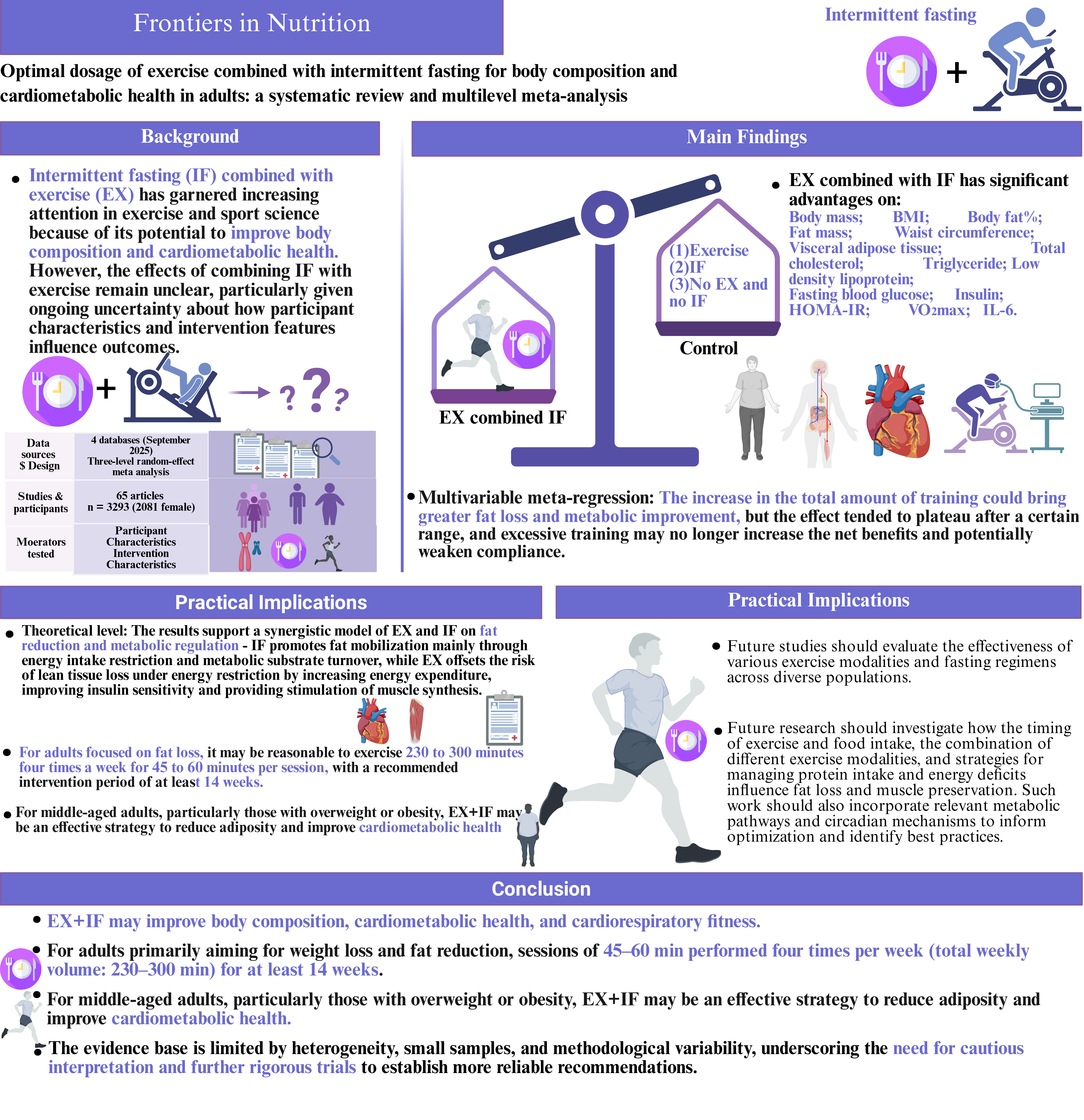

Supplement: Supplementary file 1 [file Image_1.PNG]
