# Supplementary material for: Optimal dosage of exercise combined with intermittent fasting for body composition and cardiometabolic health in adults: a systematic review and multilevel meta-analysis
Source: Front Nutr. 2026 Mar 10;13:1772836. doi: 10.3389/fnut.2026.1772836 (PMC13008886; doi:10.3389/fnut.2026.1772836)
Supplement: Supplementary file 2 [file Data_Sheet_1.docx]

Optimal dosage of exercise combined with intermittent fasting for body composition and cardiometabolic health in adults: a systematic review and multilevel meta-analysis

**Mingyue Jiao^1†^, Henghao Yan^2†^, Binbin Zhang^3,4*^, Xiaohui Zhao^1^, Jian Li^4,5^, Mohd Taib Harun^4^**

^1^School of Teacher Education, Hezhou University, Hezhou Guangxi 542899, China.

^2^School of Physical Education, Southwest University, Chongqing 400715, China.

^3^Faculty of Sports and Leisure,Guangdong Ocean University,Zhanjiang, Guangdong 524088, China.

^4^Faculty of Education and Liberal Arts, INTI International University, Nilai Negeri Sembilan 71800, Malaysia.

^5^Shanxi Institute of Science and Technology, Jincheng Shanxi, 048000, China.

* Corresponding author.

Email address: DrZhangBinbin@163.com

**Supplementary Table 1**

| **Section/topic** |  | **Checklist item** | **Reported on page #** |
| --- | --- | --- | --- |
| **Optimal dosage of exercise combined with intermittent fasting for body composition and cardiometabolic health in adults: a systematic review and multilevel meta-analysis** | | |  |
| Title |  | Identify the report as a systematic review, meta-analysis, or both. | 1 |
|  | | |  |
| Structured summary |  | Provide a structured summary including, as applicable: background; objectives; data sources; study eligibility criteria, participants, and interventions; study appraisal and synthesis methods; results; limitations; conclusions and implications of key findings; systematic review registration number. | 1 |
| **INTRODUCTION** | | |  |
| Rationale |  | Describe the rationale for the review in the context of what is already known. | 2 |
| Objectives |  | Provide an explicit statement of questions being addressed with reference to participants, interventions, comparisons, outcomes, and study design (PICOS). | 3 |
| **METHODS** | | |  |
| Protocol and registration |  | Indicate if a review protocol exists, if and where it can be accessed (e.g., Web address）, and, if available, provide registration information including registration number. | 3 |
| Eligibility criteria |  | Specify study characteristics (e.g., PICOS, length of follow-up） and report characteristics (e.g., years considered, language, publication status） used as criteria for eligibility, giving rationale. | 3 |
| Information sources |  | Describe all information sources (e.g., databases with dates of coverage, contact with study authors to identify additional studies） in the search and date last searched. | 3 |

| Search |  | Present full electronic search strategy for at least one database, including any limits used, such that it could be repeated. | 3 |
| --- | --- | --- | --- |
| Study selection |  | State the process for selecting studies (i.e., screening, eligibility, included in systematic review, and, if applicable, included in the meta-analysis）. | 3 |
| Data collection process | 0 | Describe method of data extraction from reports (e.g., piloted forms, independently, in duplicate） and any processes for obtaining and confirming data from investigators. | 3 |
| Data items | 1 | List and define all variables for which data were sought (e.g., PICOS, funding sources） and any assumptions and  simplifications made. | 3 |
| Risk of bias in individual studies | 2 | Describe methods used for assessing risk of bias of individual studies (including specification of whether this was done at the study or outcome level）, and how this information is to be used in any data synthesis. | 3 |
| Summary measures | 3 | State the principal summary measures (e.g., risk ratio, difference in means）. | 4 |
| Synthesis of results | 4 | Describe the methods of handling data and combining results of studies, if done, including measures of consistency  (e.g., I^2^ for each meta-analysis. | 4 |

| **Section/topic** | **#** | **Checklist item** | **Reported on page #** |
| --- | --- | --- | --- |
| Risk of bias across studies | 15 | Specify any assessment of risk of bias that may affect the cumulative evidence (e.g., publication bias, selective reporting within studies）. | 4-5 |
| Additional analyses | 16 | Describe methods of additional analyses (e.g., sensitivity or subgroup analyses, meta-regression）, if done,  indicating which were pre-specified. | 4-5 |
| **RESULTS** | | |  |
| Study selection | 17 | Give numbers of studies screened, assessed for eligibility, and included in the review, with reasons for | 5 |

|  |  | exclusions at each stage, ideally with a flow diagram. |  |
| --- | --- | --- | --- |
| Study characteristics | 18 | For each study, present characteristics for which data were extracted (e.g., study size, PICOS, follow-up period）  and provide the citations. | 5 |
| Risk of bias within studies | 19 | Present data on risk of bias of each study and, if available, any outcome level assessment (see item 1^2^）. | 10 |
| Results of individual studies | 20 | For all outcomes considered (benefits or harms）, present, for each study: (a） simple summary data for each intervention group (b） effect estimates and confidence intervals, ideally with a forest plot. | 5-9 |
| Synthesis of results | 21 | Present results of each meta-analysis done, including confidence intervals and measures of consistency. | 5-9 |
| Risk of bias across studies | 22 | Present results of any assessment of risk of bias across studies (see Item 15）. | 10 |
| Additional analysis | 23 | Give results of additional analyses, if done (e.g., sensitivity or subgroup analyses, meta-regression [see Item 16]）. | 10-11 |
| **DISCUSSION** | | |  |
| Summary of evidence | 24 | Summarize the main findings including the strength of evidence for each main outcome; consider their relevance to key groups (e.g., healthcare providers, users, and policy makers）. | 11 |
| Limitations | 25 | Discuss limitations at study and outcome level (e.g., risk of bias）, and at review-level (e.g., incomplete retrieval of identified research, reporting bias）. | 12 |
| Conclusions | 26 | Provide a general interpretation of the results in the context of other evidence, and implications for future research. | 12 |
| **FUNDING** | | |  |
| Funding | 27 | Describe sources of funding for the systematic review and other support (e.g., supply of data）; role of funders  for the systematic review. | n/a |

**Supplementary Table 2**

| **Data** | **Query** | **Results** |
| --- | --- | --- |
| **PubMed** | (("Intermittent Fasting"[Mesh] OR "Intermittent fasting"[Title/Abstract] OR "alternate-day fasting"[Title/Abstract] OR "intermittent energy restriction"[Title/Abstract] OR "periodic fasting"[Title/Abstract] OR "time-restricted feeding"[Title/Abstract] OR "time-restricted eating"[Title/Abstract] OR "5:2 diet"[Title/Abstract] OR "5:2 fasting"[Title/Abstract] OR "Ramadan"[Title/Abstract] OR "Ramadhan"[Title/Abstract] OR "time-restricted fasting"[Title/Abstract] OR "reduced meal frequency"[Title/Abstract] OR "alternate day modified  fasting"[Title/Abstract] OR "modified alternate-day fasting"[Title/Abstract] OR "whole day fasting"[Title/Abstract]) AND (Exercise*[Title/Abstract] OR aerobic*[Title/Abstract]  OR running[Title/Abstract] OR jogging[Title/Abstract] OR walk*[Title/Abstract] OR  hiking[Title/Abstract] OR swim*[Title/Abstract] OR aquatic*[Title/Abstract] OR  cycling[Title/Abstract] OR bicycle*[Title/Abstract] OR strength*[Title/Abstract] OR  physical activity*[Title/Abstract] OR fitness[Title/Abstract] OR train*[Title/Abstract] OR  resistance [Title/Abstract])) | 1252 |
| **Web of Science** | (Intermittent fasting) OR (alternate-day fasting) OR (intermittent energy restriction) OR (intermittent calorie restriction) OR (intermittent restrictive diet) OR (periodic fasting) OR (sporadic fasting) OR (time-restricted feeding) OR (time restricted eating) OR (5:2 diet) OR (5:2 fasting) OR (Ramadan) OR (Ramadhan) OR (time-restricted fasting) OR (periodic diet) OR (reduced meal frequency) OR (alternate day modified fasting) OR (modified alternate-day fasting) OR (whole day fasting) AND (Exercise*) OR (aerobic*) OR (running) OR (jogging) OR (walk*) OR (hiking) OR (swim* OR aquatic*) OR (cycling) OR (bicycle*) OR (strength*) OR (physical activity*) OR (fitness) OR (train*) OR (resistance) AND (random) OR (quasi-experimental) OR (quasi experimental) | 1757 |
| **Embase** | ('Intermittent fasting' OR 'alternate-day fasting' OR 'intermittent energy restriction' OR 'intermittent calorie restriction' OR 'intermittent restrictive diet' OR 'periodic fasting' OR 'sporadic fasting' OR 'time-restricted feeding' OR 'time-restricted eating' OR '5:2 diet' OR '5:2 fasting' OR 'Ramadan' OR 'Ramadan' OR 'time-restricted fasting' OR 'periodic diet' OR 'reduced meal frequency' OR 'alternate day modified fasting' OR 'modified alternate-day fasting' OR 'whole day fasting'):ti,ab,kw AND ('Exercise*' OR 'aerobic*' OR 'running or jogging' OR 'walk*' OR 'hiking' OR 'swim*' OR 'aquatic*' OR 'cycling' OR 'bicycle*' OR 'strength*' OR 'physical activity*' OR 'fitness or train*' OR 'resistance'):ti,ab,kw | 1956 |
| **Cochrane library** | ('Intermittent fasting' OR 'alternate-day fasting' OR 'intermittent energy restriction' OR 'intermittent calorie restriction' OR 'intermittent restrictive diet' OR 'periodic fasting' OR 'sporadic fasting' OR 'time-restricted feeding' OR 'time-restricted eating' OR '5:2 diet' OR '5:2 fasting' OR 'Ramadan' OR 'Ramadan' OR 'time-restricted fasting' OR 'periodic diet' OR 'reduced meal frequency' OR 'alternate day modified fasting' OR 'modified alternate-day fasting' OR 'whole day fasting'):ti,ab,kw AND ('Exercise*' OR 'aerobic*' OR 'running or jogging' OR 'walk*' OR 'hiking' OR 'swim*' OR 'aquatic*' OR 'cycling' OR 'bicycle*' OR 'strength*' OR 'physical activity*' OR 'fitness or train*' OR 'resistance'):ti,ab,kw | 1311 |

**Supplementary Table 3 (The characteristics for the studies included)**

| **Author (year)** | **Study characteristics** | | **Participant characteristics** | | | **Intermittent fasting intervention characteristics** | **Exercise intervention characteristics** | | | | |
| --- | --- | --- | --- | --- | --- | --- | --- | --- | --- | --- | --- |
|  | **Intervention Groups** | **Sample size(female/male)** | **Age (Years)**  **Mean ± SD** | **BMI (kg·m^−2^)**  **Mean ± SD** | **Participant** |  | **Type** | **Protocol** | **Fre** | **Sess** | **Week** |
| Haghdoost et al.2009 | RIF+AE(M)  RIF | RIF+AE(M)  : 51(0/51)  RIF: 42(0/42) | RIF+AE(M)  : 19.25 ± 3.5  RIF: 20.4 ± 3.2 | RIF+AE(M)  : 22.8 ± 3.6  RIF: 23.2 ± 3.6 | College students | During the Ramadan phase, participants fasted from dawn until sunset, abstaining from both food and drink | AE(M) | Warm-up: 10 min of jogging.  AE(M):40 min.  Cool down: 10 min exercises. | 4 | 40-60 | 3 |
| Tayebi et al.2010 | RIF+RT  RT  RIF  No EX and no IF | RIF+RT:10(0/10)  RT:10(0/10)  RIF:10(0/10)  No EX and no IF:10(0/10) | 21.3± 1.6 | NR | Weight lifter | During the Ramadan phase, participants fasted from dawn until sunset, abstaining from both food and drink | RT | RT: Weightlifting technique training and hypertrophy body building.  Intensity: 65%-75% 1-RM | 3 | 90 | 30 days |
| Trabelsi et al.2012 | RIF+AE(M)  AE(M) | RIF+AE(M):10(0/10)  AE(M):9(0/9) | RIF+AE(M):26.6 ± 3.0  AE(M):27.6 ± 1.8 | RIF+AE(M):24.6 ± 1.4  AE(M):24.5 ± 1.6 | ~~Physically active men~~ | Fasted for roughly 15 h each day | AE(M) | AE(M): Bicycle, running and rowing machine once each.  Intensity: 60-80% heart rate max | 3 | 40-60 | 30 days |
| Bhutani 2013-1 et al.2013 | ADF+AE(M)  AE(M)  ADF  No EX and no IF | ADF+AE(M):18(18/0)  AE(M):24(23/1)  ADF:25(24/1)  No EX and no IF:16(15/1) | ADF+AE(M):45 ± 5  AE(M):42 ± 2  ADF:42 ± 2  No EX and no IF:49 ± 2 | 30-39.9 | Obesity | Fast and feed days alternated every other day | AE(M) | AE(M): using stationary bikes and elliptical machines (elliptical machines). Intensity: Moderate Intensity. The initial intensity was 60% HRmax, which was gradually increased to 75% HRmax. | 3 | 40 | 12 |
| Bhutani 2013-2 et al.2013 | ADF+AE(M)  AE(M)  ADF  No EX and no IF | ADF+AE(M):18(18/0)  AE(M):24(23/1)  ADF:25(24/1)  No EX and no IF:16(15/1) | ADF+AE(M):45 ± 5  AE(M):42 ± 2  ADF:42 ± 2  No EX and no IF:49 ± 2 | 30-39.9 | Obesity | Fast and feed days alternated every other day | AE(M) | AE(M): using stationary bikes and elliptical machines (elliptical machines). Intensity: Moderate Intensity. The initial intensity was 60% HRmax, which was gradually increased to 75% HRmax. | 3 | 40 | 12 |
| Trabelsi et al.2013 | RIF+RE  RT | RIF+RE:8(0/8)  RT:8(0/8) | RIF+RE:25 ± 3  RT:25 ± 2 | RIF+RE:25.8 ± 0.4  RT:26.0 ± 1.7 | Bodybuilder | Fasted for roughly 15 h each day | RT | RT: Each training session consisted of four to six specific exercises. Each exercise was performed in four sets with a load of 10 RM and 2-3 min intervals between sets. | 4 | 60 | 4 |
| Moro et al.2016 | TRF+RT  RT | TRF+RT:17(0/17)  RT:17(0/17) | TRF+RT:29.94 ± 4.07  RT:28.47 ± 3.48 | NR | Male Healthy participants (Resistance trained) Trained | Fasting for the remaining 16 h each day. | RT | Engaged in a split resistance training routine, completing three weekly sessions. Each session comprised three sets of 6–8 reps at 85–90% of 1-RM, performed to failure with 3 min rest intervals between sets and exercises. | 3 | 40-60 | 8 |
| Tinsley et al.2017 | TRF+RT  RT | TRF+RT:10(0/10)  RT:8(0/8) | TRF+RT:22.9 ± 4.1  RT:22.0± 2.4 | NR | Male Healthy participants Trained | Instructed to consume all their calories within a flexible four-hour window between 4:00 p.m. and midnight. | RT | Engaged in a split resistance training routine, completing three weekly sessions. Each session comprised three sets of 6–8 reps at 85–90% of 1-RM, performed to failure with 3 min rest intervals between sets and exercises. | 3 | 45-60 | 8 |
| Tinsley et al.2019 | TRF+RT  RT | TRF+RT:21(21/0)  RT:17(17/0) | TRF+RT:22.3 ± 3.4  RT:22.0 ± 2.4 | NR | ~~Active females~~ | On non-training days (four days per week), participants were instructed to consume all their calories within a flexible four-hour window between 4:00 p.m. and midnight. | RT | Alternating upper and lower body workouts, completing four sets of 8–12 repetitions per exercise, with 90 s of rest between sets, three times per week. | 3 | 45-60 | 8 |
| Aloui et al.2018 | RIF+RT  RIF+RT  RIF | RIF+RT:10(0/10)  RIF:10(0/10) | 22.9±1.3 | 22.5 | Amateur male football player | Participants fasted from 3:00 am (last meal before dawn) until sunset (about 19:20) and continued throughout Ramadan (about 16 hours). | RT | Each training session consisted of three sets × six 40-m round-trip sprints (20 m +180° turning) with a 4-min rest between sets and a 20-s rest between sprints | 4 | 30 | 4 |
| Cho et al.2019 | ADF+AE+RT  ADF  AE+RT  No EX and no IF | ADF+AE+RT:9(4/5)  ADF:8(6/2)  AE+RT:9(4/5)  No EX and no IF:5(2/3) | ADF+AE+RT:34.5 ± 5.7  ADF:33.5 ± 5.0  AE+RT:38.6 ± 8.2  No EX and no IF:42.6 ± 10.6 | ADF+AE+RT:28.0 ± 2.6  ADF:27.8 ± 3.4  AE+RT:26.9 ± 3.9  No EX and no IF:25.8 ± 3.4 | Obesity | Fast day: energy intake: 25% of the recommended daily energy (about 500 kcal), intake of 1 meal between 12:00 and 14:00. 3 days a week (alternate days) | AE+RT | Resistance training (40 min/session) : Use equipment (such as chest push, leg lift, shoulder push, high pull). Aerobic exercise (20 min/session) : Treadmill training with intensity individualized based on maximal oxygen uptake (VO_2_max). | 3 | 60 | 8 |
| Campbell et al.2020 | IER+RT+AE(M)  RT+AE(M) | IER+RT+AE(M):13(7/6)  RT+AE(M):14(6/8) | IER+RT+AE(M):26.3 ± 8.0  RT+AE(M):24.2 ± 3.7 | IER+RT+AE(M):27.6  RT+AE(M):28 | Healthy | The refeed group reduced caloric intake by 35% for 5 days (Monday–Friday) and consumed maintenance calories for two consecutive days (Saturday–Sunday), comprising increased calories as carbohydrate only. | RT+AE(M) | RT: Divided into upper/lower limb training days, including bench press, row, deadpull, hip press, etc. AE(M): running, cycling, swimming, etc. | 2 | 30 | 7 |
| Hottenrott et al.2020 | 5:2+ET+ST  ET+ST | 5:2+ET+RT:18(35/33)  ET+RT:17(/) | 45.5 ± 7.8 | 25-30 | Obesity | (first 3 weeks) : Half-day fasting for 3 days per week (800 kcal/ day for women and 1200 kcal/ day for men). (last 9 weeks) : Strict fasting (400 kcal/ day for women and 600 kcal/ day for men) for 2 days per week, normal diet for the remaining 5 days | ET+ RT | ET+ RT: 30–60 min running and 20 min of strength training 3 to 4 times a week. walking program up to 2 h every weekend. | 3-4 | 80 | 12 |
| Jospe et al.2020 | 5:2+AE(M)  AE(M)  No EX and no IF | 5:2+AE(M):136(76/60)  AE(M):68(46/22)  No EX and no IF:46(34/12) | 5:2+AE(M):43.9±11.1  AE(M):44.2±11.7  No EX and no IF:42.6±9.6 | 5:2+AE(M):32.9±4.2  AE(M):32.5±4.1  No EX and no IF:34.1±5.3 | Obesity | The 5:2 pattern (normal diet, 5 days a week, very low calorie intake, 2 days a week). Caloric restriction: ≤500 kcal/ day for women and ≤600 kcal/ day for men (actual mean: 784±402 kcal/ day). Fasting days could be either consecutive or nonconsecutive. | AE(M) | 150 minutes of moderate-intensity aerobic activity weekly. | 3 | 50 | 12 mos |
| Moro et al.2020 | TRE+AE(M)  AE(M) | TRE+AE(M):8(0/8)  AE(M):8(0/8) | TRE+AE(M):19.38 ± 2.39  AE(M):19.38 ± 1.60 | TRE+AE(M):21.85 ± 1.65  AE(M):22.47 ± 1.83 | Elite cyclists | The TRE group consumed 100% of its estimated daily energy needs in an 8-h time window (from 10:00 a.m. to 6:00 p.m.) | AE(M) | AE(M): 500 ± 50 km cycling per week, mainly steady state training of moderate intensity. | 6 | 143 | 4 |
| Stratton et al.2020 | TRE+RT  RT | TRE+RT:13(0/13)  RT: 13(0/13) | TRE+RT:22.9 ± 3.6  RT: 22.5 ± 2.2 | TRE+RT:25.8  RT:26.4 | Amateur Active Men | Daily 16-hour fasting with an 8-hour feeding window (12 noon to 8 PM or 1 PM to 9 PM). | RT | RT: Squats, bench press, rowing, shoulder training, etc | 3 | 60 | 4 |
| Martínez-Rodríguez et al.2020 | TRE+HIIT  HIIT | TRE+HIIT:7(7/0)  HIIT:7(7/0) | 27 ± 6 | 21.1 ± 1.8 | ~~Active women~~ | Time-restricted feeding (fasting window >14 hours, breakfast consumed immediately after waking, followed by meal distribution within remaining hours) on alternate days (3 fasting days/week in odd weeks, 4 days/week in even weeks) | HIIT | HIIT: three sets ×10 reps ×30 seconds of all-out aerobic exercise (cycling, rowing, running) with 30 seconds of passive recovery interval. | 3 | 40 | 8 |
| Moro et al.2021 | TRE+RT  RT | TRE+RT:10(0/10)  RT:10(0/10) | >18 | NR | Healthy | The daily eating window was 8 hours (13:00, 16:00, 20:00), and 40% of calories were allocated for breakfast, 25% for lunch, and 35% for dinner. The protein intake was about 1.9 g/kg body weight. | RT | 1RM tests of Leg Press and Bench Press, alternating strength and muscle hypertrophy training with 1RM at 75% to 90% intensity | 3 | 120 | 12 mos |
| Berk et al.2021 | TRE+AE(M)  TRE | TRE+AE(M):18(-)  TRE:18(-) | 20-24 | TRE+AE(M):24.55±3.32  TRE:23.90±3.77 | Healthy | 16:8 time-restricted eating (fasting for 16 hours/day, eating window: 10:00–18:00). | AE(M) | 12 exercises per session (e.g., Jumping Jack, Squat, Crunches), each for 30 seconds, followed by 1-min rest after 4 exercises | 5 | 36 | 30 days |
| Brady et al.2021 | TRE+AE(M)  AE(M) | TRE+AE(M):10(0/10)  AE(M):7(0/7) | TRE+AE(M):35.9 ± 8.6  AE(M):39.9 ± 3.0 | TRE+AE(M):22.52 ± 2.44  AE(M):22.32 ± 2.37 | Male middle- and long-distance runners | Participants in the TRE group were instructed to consume all of their dietary intake within an 8-h window, typically between 1200 and 2000 h. Only water was permitted outside the window. | AE(M) | Average weekly training load (Session-RPE method) : TRE group: 1614±410 AU. Control group: 1718±476 AU. Training ≥5 days per week | 5 | 40 | 8 |
| Brini et al.2021 | RIF+HIIT  HIIT | RIF+HIIT:12(0/12)  HIIT:12(0/12) | RIF+HIIT:25.32 ± 2.56  HIIT:24.85 ± 1.55 | RIF+HIIT:23.43 ± 1.19  HIIT:23.41 ± 1.46 | Professional basketball players | The daily fasting period was about 16-17 hours. They ate only at night and ate less frequently than usual. Daytime training sessions were held between 5:00 and 6:30 p.m | HIIT | A 3-on-3 game was played in a half-court (14×15 m) with a total duration of 12 min per game (divided into three 4-min sessions with a 2-min rest between sessions). Only man - to - man strategies are allowed. | 5 | 12 | 4 |
| Isenmann et al.2021 | TRE+RT+AE  RT+AE | TRE+RT+AE:18(10/8)  RT+AE:17(11/6) | TRE+RT+AE:27.9 ± 5.3  RT+AE:27.4 ± 5.8 | TRE+RT+AE:26.3 ± 3.0  RT+AE:25.7 ± 3.3 | Healthy | 16:8 method (12:00-20:00 eating window, 16-hour fasting) | RT+AE | NR | 2 | 45 | 14 |
| Kotarsky et al.2021 | TRE+RT+AE(MV)  RT+AE(MV) | TRE+RT+AE(MV):11(9/2)  RT+AE(MV):10(9/1) | TRE+RT+AE(MV):45.0 ± 9.9  RT+AE(MV):44.0 ± 6.3 | TRE+RT+AE(MV):29.8 ± 2.7  RT+AE(MV):29.4 ± 2.5 | Obesity | TRE protocol: consume all their calories between 12:00 p.m. and 8:00 p.m. each day,inducing a fasting window of 16 h | RT+AE(MV) | RT: three sets of 12 reps with up to 60 s rest between sets.  AE(MV): 75 min at moderate-to-vigorous intensity (≥55% HRR) on a treadmill or similar equipment. | 3 | 75 | 8 |
| O’Reilly et al.2021 | IER+AE(M)  AE(M) | IER+AE(M):29(14/15)  AE(M):30(17/13) | IER+AE(M):48.9±4.4  AE(M):48.9 ± 4.0 | IER+AE(M):31.2±3.3  AE(M):31.0 ± 3.1 | East Asian Adults | Restrict energy to 25% (~ 500 kcal) 2 days a week | AE(M) | Walking for 1 hour 5 days a week (or equivalent activity) | 5 | 60 | 12 |
| Peos et al.2021 | IER+RT:  RT | IER+RT:30(15/15)  RT:31(17/13) | 28.7±6.5 | NR | Adult | The total duration was 15 weeks, consisting of 4 3-week rounds of moderate energy restriction (with a weekly weight loss target of 0.7% of initial body weight) and 3 1-week rounds of dietary breaks (energy balance). | RT | NR | 2 | 60 | 15 |
| Steger et al.2021 | IER+AE(MV)  AE(MV) | IER+AE(MV):18(13/5)  AE(MV):17(14/3) | IER+AE(MV):43.4 ± 11  AE(MV):48 ± 10 | IER+AE(MV):31.1 ± 2.4  AE(MV):31.4 ± 2.5 | Obesity | A very-low-energy diet (VLED, 550-800 kcal/ day) with 2 to 3 servings of meal replacement drinks, 1 serving of staple food, 1 serving of fruit, and unlimited nonstarches vegetables was prescribed for 3 days per week, with no specific caloric restriction for the remaining 4 days | AE(MV) | Brisk walking and running increased to ≥150 minutes per week (60 minutes at week 1 and ≥150 minutes per week from week 4), with participants averaging 205.9 to 226.5 minutes per week during the weight loss phase (weeks 0-12) and 248.5 to 314.8 minutes per week during the maintenance phase (weeks 12-24) | 7 | 45 | 24 |
| Xu et al.2021 | IER+AE(M)  AE(M) | IER+AE(M):8(7/1)  AE(M):10(7/3) | IER+AE(M):34.3±5.1  AE(M):29.0±6.4 | IER+AE(M):23.7±3.2  AE(M):23.9±3.3 | Sedentary, normal-weight adults | Strategy with energy-restricted days (800±40 kcal/day) alternating with ad libitum "cheat days | AE(M) | self-selected type (Walking, running)totaling 2400-2600 kcal/week. | 7 | 30-60 | 4 |
| Correia et al.2021 | TRE+HIIT  HIIT | TRE+HIIT  :12(0/12)  HIIT:12(0/12) | 22.4±2.8 | 24.2±2.0 | Male physical education students | The daily eating window is 8 hours (1 PM to 9 PM), and the remaining 16 hours are fast (only non-caloric drinks such as water, tea or coffee are allowed) | HIIT | NR | 3 | 45 | 4 |
| Tovar et al.2021 | TRE+AE(MV)  AE(MV) | TRE+AE(MV):15(0/15)  AE(MV):15(0/15) | 28.7 ± 5.2 | 23.3 | Healthy participants Trained | The 16/8 protocol required participants to consume all meals within a consistent 8 h daily window. During the fasting period, only water and non-caloric beverages such as unsweetened black coffee or plain tea were permitted. | AE(MV) | One day with high-intensity exercise, one day with moderate-intensity exercise | 3 | 23 | 4 |
| Batitucci et al.2022 | 5:2+HIIT  5:2  HIIT | 5:2+HIIT:15(15/0)  5:2:10(10/0)  HIIT:11(11/0) | 5:2+HIIT:32.0 ± 4.4  5:2:30.0 ± 5.0  HIIT:33.0 ± 3.0 | 5:2+HIIT:34.0 ± 2.6  5:2:34.8 ± 3.2  HIIT:31.8 ± 2.02 | Obesity | 5:2 (5 days’ ad libitum & 2 days fasting); 6:18 h (6 h eating period & 18 h of complete fasting); 25% of the total daily energy needs on IF days (approximate total of 600 kcal that were divided into two meals), while on the other days of the week food was allowed ad libitum | HIIT | Exercise execution 30-45 s at 70–85% HRmax with 15-30 s recovery, total 25 min duration | 3 | 25 | 8 |
| Cooke et al.2022 | 5:2+HIIT  5:2  HIIT | 5:2+HIIT:11(8/3)  5:2:12(10/2)  HIIT:11(10/1) | 5:2+HIIT:39.0 ± 6.8  5:2:37.0 ± 5.9  HIIT:32.0 ± 8.3 | 5:2+HIIT:34.0 ± 2.1  5:2:30.0 ± 3.9  HIIT:32.0 ± 4.4 | Obesity | 5:2 (2 days fasting & 5 days’ ad libitum), fasting day intake was limited to a single 600 kilocalorie (kcal) foe men and 500 kcal meal for women | HIIT | 4–6 × 20 s cycling at 150% VO_2_peak followed by 40 s of active recovery, total 10 min duration | 3 | 10 | 16 |
| Haganes et al.2022 | TRE+HIIT  TRE  HIIT  No EX and no IF | TRE+HIIT:32(32/0)  TRE:33(33/0)  HIIT:33(33/0)  No EX and no IF:33(33/0) | TRE+HIIT:37.3 ± 5.7  TRE:36.2 ± 5.9  HIIT:34.9 ± 7.0  No EX and no IF:36.4 ± 6.2 | TRE+HIIT:31.4 ± 4.0  TRE:31.8 ± 3.3  HIIT:32.5 ± 4.5  CON:33.1 ± 4.2 | Obesity | ≤ 10-h daily eating window, with ad libitum energy intake | HIIT | 4 × 4-min work bouts at 90–95% HR_max_, separated by 3 min moderate-intensity recovery, while the third session comprised 10 × 1-min work bouts at ≥ 90% of HR_max_ separated by 1 min low-intensity recovery | 3 | 36 | 7 |
| Kammoun et al.2022 | RIF+HIIT  HIIT | RIF+HIIT:18(0/18)  HIIT:13(0/13) | RIF+HIIT:53.70 ± 7.3  HIIT:52.75 ± 4.2 | RIF+HIIT:28.2 ± 3.0  HIIT:27.9 ± 2.3 | Healthy sedentary middle-aged men | From sunrise to sunset (15-16 hours daily). | HIIT | Warm-up (10 min), two 20-min periods of play (10 min interval), stretch (5 min). It was carried out in a reduced size venue (30-45m × 45-60m). | 3 | 65 | 4 |
| Keenan et al.2022 | 5:2+RT  RT | 5:2+RT:17(8/9)  RT:17(9/8) | 5:2+RT:24.7 ± 4.8  RT:23.2±3.9 | 25:2+RT:27.0 ± 2.7  RT:27.1 ± 2.9 | Healthy | Twice-weekly fast (5:2 IF). Fasting days involved consumption of high-protein shakes and soups, as well as non-starchy vegetables. providing ~20% of energy requirements. Participants aimed for 100% energy requirements on the remaining 5 days. ≥1.4 g of protein per kilogram of body weight per day Fasting on non-consecutive, non-training days each week. | RT | RE: Supervised resistance training sessions. Two 45-minute supervised session and one 30-minute unsupervised session per week. | 3 | 45 | 12 |
| Keenan et al.2022 | TRE+RT+AE  RT+AE | TRE+RT+AE:17(8/9)  RT+AE:17(9/8) | TRE+RT+AE:24.7 ± 4.8  RT+AE:23.2 ± 3.9 | TRE+RT+AE:27.0 ± 2.7  RT+AE:27.1 ± 2.9 | Healthy | TRE: ~70% energy restriction (~2100 kJ/day females, ~2500 kJ/day males) 2 non-consecutive days/week, euenergetic intake 5 days/week, mean ≥1.4 g protein/kg/day | RT+AE | RT: (2x/week) + body weight aerobic/resistance combination (1x/week). Push-ups, squats, rows, lunges, bicep curls, dips etc., aiming for 12-15 reps/set. Combination: Planks, mountain climbers, crunches, burpees, lying side toe-touches, hip bridges etc. | 3 | 45 | 12 |
| Xu et al.2022 | ADF+HIIT  HIIT | ADF+HIIT:16(9/7)  HIIT:16(10/6) | 21.3 ± 2.24 | ADF+HIIT:26.90 ± 1.46  HIIT:26.39 ± 1.63 | Obesity | Intermittent energy restriction protocol: 2:5, 30% of their daily recommended energy intake (approximately 500–1,000 kcal) | HIIT | 5 intervals of 3 min at 80% of VO_2_max separated by brief periods at 50%(Total time:30 min). | 5 | 30 | 4 |
| Correia et al.2023 | TRE+HIIT  HIIT | TRE+HIIT:18(0/18)  HIIT:18(0/18) | TRE+HIIT:23.7 ± 2.6  HIIT:23.7 ± 2.6 | 23.9 | Healthy participants Trained | TRE: participants ate two to three ad libitum meals within an 8 h window (1–9 p.m.), consuming only water, tea, and black coffee outside this period. | HIIT | Participants followed a structured training program during each dietary intervention, performing four sets of 8–10 repetitions at 85% of their 1-RM on leg press, bench press, leg extension, leg curl, shoulder press, and lat pulldown | 3 | 60 | 4 |
| Ezpeleta et al.2023a | RIF+AE(M)  RIF  AE(M)  No EX and no IF | RIF+AE(M):20(17/3)  RIF:20(16/4)  AE(M):20(16/4)  No EX and no IF:20(16/4) | RIF+AE(M):44.0 ± 13.4  RIF:44.0 ± 13.4  AE(M):44.0 ± 13.4  No EX and no IF:44 ± 12 | RIF+AE(M):37 ± 5  RIF:36 ± 8  AE(M):37 ± 6  No EX and no IF:37 ± 5 | Obesity | 600 kcal/2,500 kJ “fast day” alternated with an ad libitum intake “feast day”. The feast and fast days began at 12 am each day. Therefore, subjects fasted for approximately 17–20 h on the fast day | AE(M) | 60 min at 65–80% of heart rate max | 5 | 60 | 12 |
| Ezpeleta et al.2023b | RIF+AE(M)  RIF  AE(M)  No EX and no IF | RIF+AE(M):20(17/3)  RIF:20(16/4)  AE(M):20(16/4)  No EX and no IF:20(16/4) | RIF+AE(M):44.0 ± 13.4  RIF:44.0 ± 13.4  AE(M):44.0 ± 13.4  No EX and no IF:44 ± 12 | RIF+AE(M):37 ± 5  RIF:36 ± 8  AE(M):37 ± 6  No EX and no IF:37 ± 5 | Obesity | 600 kcal/2,500 kJ “fast day” alternated with an ad libitum intake “feast day”. The feast and fast days began at 12 am each day. Therefore, subjects fasted for approximately 17–20 h on the fast day | AE(M) | 60 min at 65–80% of HRmax | 5 | 60 | 12 |
| Richardson et al.2023 | TRE+AE(M)  AE(M) | TRE+AE(M):15(0/15)  AE(M):15(0/15) | 28.7±5.2 | 23.3 | Long-distance runner | Daily fasting was 16 h with an 8-h feeding window | AE(M) | The weekly running volume was 52.9±10.8 km | 4 | 80 | 4 |
| Liu et al.2023 | TRE+AE  TRE  AE(M)  No EX and no IF | TRE+AE:19(19/0)  TRE:19(19/0)  AE(M):20(20/0)  No EX and no IF:19(19/0) | TRE+AE:19.93 ± 0.61  TRE:20.29 ± 1.79  AE(M):20.09 ± 1.35  CON:20.08 ± 1.76 | TRE+AE:21.86 ± 1.48  TRE:21.63 ± 1.24  AE(M):21.35 ± 1.56  No EX and no IF:20.32 ± 1.06 | Obesity | Time-restricted feeding for 8 h, that is, eating at their discretion between 10:00 and 18:00 daily and fasting for the remainder of the day | AE(M) | 11,000–12,499 steps per day | 7 | 115 | 8 |
| Maaloul et al.2023 | RIF+HIIT+RT  HIIT+RT | RIF+HIIT+RT:10(0/10)  HIIT+RT:10(0/10) | RIF+HIIT+RT:30.2 ± 6.1  HIIT+RT:33.4 ± 7.9 | RIF+HIIT+RT:34.1 ± 4.9  HIIT+RT:32.06 ± 3.6 | Obesity | Ramadan diurnal intermittent fasting protocol: the daily fasting duration during this study was approximately 16h | HIIT+RT | 8 intervals of 1 min at 90% of VO2max alternated with 2 min recovery at 45% of VO_2_max, and resistance exercised; 3 sets with 10–12 reps at 60–75% of 1RM | 4 | 50 | 4 |
| Siedler et al.2023 | ADF+RT  RT | ADF+RT:18(18/0)  RT:20(20/0) | ADF+RT:23.3±4.4  RT:21.3±3.8 | ADF+RT:24.3  RT:22.5 | Healthy participants Trained | An intermittent energy-restricted diet (INT) that included a one-week period of energy balance after the second and fourth weeks of energy restriction. | RT | An alternating upper body/lower body split. | 4 | 30 | 8 |
| Triki et al.2023 | RIF+RT  RT | RIF+RT:20(0/20)  RT:20(0/20) | 25.7±5.6 | 28.3±5.7 | Healthy | Daily fasting from dawn to sunset (~14h/day) with complete abstention from food/fluid intake during daylight hours, ad libitum eating permitted at night | RT | Two weekly exercise pairs (Monday/Thursday: lower-body; Tuesday/Friday: upper-body). | 4 | 45-60 | 8 |
| Correia et al.2024 | TRE+AE+HIIT  AE+HIIT | TRE+AE+HIIT:15(0/15)  AE+HIIT:15(0/15) | TRE+AE+HIIT:23.7 ± 2.6  AE+HIIT:23.7 ± 2.6 | NR | Healthy participants Trained | The TRE intervention used a 16/8 protocol, where participants consumed 2 to 3 meals within an 8 h window (1:00–9:00 p.m.) and were allowed only water, tea, and black coffee outside this period. | AE+HIIT | During the first 2 weeks of the intervention, participants completed 10 km runs per session with 24 h of rest between workouts. In the final 2 weeks, they continued the 10-km runs and added 1 km running intervals after each continuous run, separated by 4 min of active recovery | 3 | 60 | 4 |
| Ameur et al.2024 | TRE+HIIT  TRE  HIIT | TRE+HIIT:20(20/0)  TRE:20(20/0)  HIIT:24(24/0) | TRE+HIIT:33.8 ± 5.3  TRE:30.2 ± 6.1  HIIT:31.8 ± 2.2 | TRE+HIIT:35.2 ± 3.5  TRE:35 ± 4.3  HIIT:34.8 ± 3.6 | Obesity | 16-hour fasting window (4:00 p.m. to 8:00 a.m.) 8-hour eating window (8:00 a.m. to 4:00 p.m.) Ad libitum energy intake during eating window. | HIIT | Each session consists of 8 sets of 8 functional exercises (aerobic and resistance). Using self-selected pace and weight/resistance (body weight or free weights). 20-30s work/10s rest per exercise. Maintain RPE ≥7Duration/Frequency: 3 sessions/week (Monday, Wednesday, Friday). | 3 | 45-55 | 12 |
| Batitucci et al.2024 | 5:2 + HIIT  5:2  HIIT | 5:2+ HIIT:15(15/0)  5:2: 10(10/0)  HIIT:11(11/0) | 18-40 | 5:2+ HIIT:34.0 ± 3.2  5:2: 34.8 ± 3.2  HIIT:31.8 ± 2 | Obesity | 5:2 Protocol: 6-hour feeding window with 2 meals (e.g., 12:00-18:00), 18-hour fasting (water/tea/coffee allowed) | HIIT | 4-min warm-up, 18-min main training (70-85% HRmax), 3-min cool-down | 3 | 25 | 8 |
| Czerwi ´nska-Ledwig et al.2024a | RIF+AE(M)  No EX and no IF | RIF+AE(M):25(25/0)  No EX and no IF:26(26/0) | RIF+AE(M):65.2 ± 4.8  No EX and no IF:65.2 ± 4.8 | RIF+AE(M):29.41±4.53  No EX and no IF:28.43±5.57 | Obesity | Fasting for 14 hours daily (10-hour feeding window) with no change in dietary pattern | AE(M) | Three sessions (technical training, strength stretching, and endurance training) were performed at an intensity of 70% of maximum heart rate | 3 | 60 | 12 |
| Czerwi ´nska-Ledwig et al.2024b | RIF+AE(M)  No EX and no IF | RIF+AE(M):21(14/7)  No EX and no IF:14(3/8) | RIF+AE(M):65.4 ± 5.5  No EX and no IF:65.2 ± 4.8 | RIF+AE(M):30.2 ± 3.5  No EX and no IF:28.6±4.2 | Obesity | Fasting for 14 hours daily (10-hour feeding window) with no change in dietary pattern | AE(M) | Three sessions (technical training, strength stretching, and endurance training) were performed at an intensity of 70% of maximum heart rate | 3 | 60 | 6 |
| Hikmat et al.2024 | ADF+RT  ADF  RT  No EX and no IF | ADF+RT:10(0/10)  ADF:10(0/10)  RT:10(0/10)  No EX and no IF:10(0/10) | ADF+RT:27.10±4.6  ADF:26.2±4.3  RT:28.7±4.8  No EX and no IF:27.9±3.3 | ADF+RT:32.05±2.3  ADF:33.5±2.8  RT:32.3±3.3  No EX and no IF:33.1±2.3 | Obesity | Fasting days (Sunday, Tuesday, Thursday): Consumed 25% of daily recommended energy intake (400-500 kcal), with eating window from 12:00 to 14:00. Feed days: Ad libitum food intake | RT | Barbell bench press, Knee extension, Knee flexion, Seated row, Barbell shoulder press, Barbell biceps curl, Seated dumbbell triceps extension, Leg press, Fly dumbbell, Crunch, Barbell squat, Incline barbell bench press.  Intensity and load: Week 1: 50% of 1RM. Weeks 2-5: Gradually increased by ~8% per week to 80% 1RM. Last 3 weeks: 85% 1RM. | 3 | 80 | 8 |
| Keawtep et al.2024 | 5:2 +RT+AE  5:2  RT+AE  No EX and no IF | 5:2+RT+AE:23(23/0)  5:2: 23(23/0)  RT+AE:23(23/0)  No EX and no IF:23(23/0) | 5:2+RT+AE:52.17±3.35  5:2: 52.87±3.88  RT+AE:52.70±3.60  No EX and no IF:53.61±2.81 | 5:2+RT+AE:29.73±4.57  5:2: 28.28±2.78  RT+AE:29.06±2.90  No EX and no IF:29.18±2.85 | Obesity | Fasting days/week (non-consecutive): Weeks 1–4: 75% of estimated energy needs. Weeks 5–8: 50%. Weeks 9–12: 25%. | RT+AE | Type: Home-based physical-cognitive dual-task training (YouTube-guided). Warm-up (10 min). Main session (40 min): Physical: Moderate-intensity resistance + aerobic exercises (e.g., cuff weights, aerobic dance). | 3 | 60 | 3mos |
| Rejeki et al.2024 | TRE+AE(M)  TRE  AE(M)  No EX and no IF | TRE+AE(M):10(10/0)  TRE:10(10/0)  AE(M):10(10/0)  No EX and no IF:10(10/0) | TRE+AE(M):22.90±1.59  TRE:24.20±2.69  AE(M):23.30 ± 1.57  No EX and no IF:23.90 ± 0.99 | TRE+AE(M):31.57 ± 3.61  TRE:31.22 ± 5.01  AE(M):28.36 ± 2.87  No EX and no IF:29.19 ± 2.49 | Obesity | Method: 16:8 fasting (16-h fasting, 8-h eating), water permitted during fasting. Frequency: 5×/week (Monday–Thursday + Saturday) for 2 weeks | AE(M) | Type: Moderate-intensity treadmill aerobic exercise (60–70% HRmax). Duration: 40 min/session (5 min warm-up, 30 min core, 5 min cool-down) | 5 | 40 | 2 |
| Sertdemir et al.2024 | TRE+AE(M)  TRE  AE(M)  No EX and no IF | TRE+AE(M):12(/)  TRE:12(/)  AE(M):12(/)  No EX and no IF:8(/) | TRE+AE(M):67.3±9  TRE:66±6  AE(M):70±8.9  No EX and no IF:75±9 | 31.1 | Elderly volunteers | Intermittent fasting group followed a 16-hour food restriction schedule on two non-consecutive days of the week (from 18:00 in the evening to 10:00 the next day). | AE(M) | 60–75% of HRmax | 5 | 120 | 12 |
| Triki et al.2024 | RIF+RT  RT | RIF+RT:20(0/20)  RT:20(0/20) | RIF+RT:26.8±5.1  RT:24.6±5.9 | RIF+RT:28.1±5.2  RT:28.5±6.2 | Healthy | Daily fasting from dawn to sunset (~15 h/day) for 29 days, with food/fluid intake only at night | RT | Whole-body RT (e.g., squats, deadlifts, bench press). 4 sessions/week (Mon, Tue, Thu, Fri), 5 exercises/session, 4 sets×12 reps at 75–85% 1-RM, 2-min rest between sets. | 4 | 45-60 | 8 |
| Bagherpour et al.2025 | TRE+RT  RT | TRE+RT:8(8/0)  RT:8(8/0) | TRE+RT:25 ± 3.4  RT:23.00 ± 2.1 | TRE+RT:27.29 ± 1.56  RT:27.72±1.01 | Obesity | A 10-hour eating window (8 am - 6 pm) with 14-hour fasting TRE protocol, combined with 25% calorie restriction. | RT | 8 exercises (3 upper-body: chest press, lat pull down, bicep curls; 3 lower-body: leg press, leg extension, leg flexion; 2 core: plank and back extensions). 3 sets of 8-10 reps, progressing from 65% to 80% 1RM. | 3 | 45-60 | 8 |
| Blake et al.2025 | TRE+RT  RT | TRE+RT:10(4/6)  RT:7(3/4) | TRE+RT:25.8±3.9  RT:25.1±3.0 | TRE+RT:25.9±2.0  RT:26.6±2.0 | Healthy | A 10-hour eating window (8 am - 6 pm) with 14-hour fasting TRE protocol, combined with 25% calorie restriction. | RT | Autoregulatory Progressive Resistance Exercise (APRE).  Program: Two alternating workouts (A/B), 4 sessions/week (e.g., A: Romanian deadlifts, leg press; B: back squats, bench press). | 4 | 75 | 8 |
| Breit et al.2024 | IER+AE(M)  AE(M) | IER+AE(M):84(62/22)  AE(M):81(60/21) | IER+AE(M):42 ± 10  AE(M):42 ± 8 | IER+AE(M):34.3 ± 4.4  AE(M):33.9±4.4 | Obesity | Three non-consecutive fast days/week (80% energy restriction), ad libitum intake on non-fast days | AE(M) | Progressive increase to 300 min/week during first 6 months, maintained thereafter.  Frequency/Duration: Distributed weekly for 12 months | 5 | 60 | 12mos |
| Catenacci et al.2025 | IER+AE(M)  AE(M) | IER+AE(M):84(62/22)  AE(M):81(60/21) | IER+AE(M):42 ± 10  AE(M):42 ± 8 | IER+AE(M):34.3 ± 4.4  AE(M):33.9±4.4 | Obesity | Three non-consecutive fast days/week (80% energy restriction), ad libitum intake on non-fast days | AE(M) | Progressive increase to 300 min/week during first 6 months, maintained thereafter.  Frequency/Duration: Distributed weekly for 12 months | 5 | 60 | 12mos |
| Cui et al.2025 | TRE+RT  TRE  RT  No EX and no IF | TRE+RT:14(8/6)  TRE:15(9/6)  RT:13(9/4)  No EX and no IF:12(10/2) | TRE+RT:21±2  TRE:20±1.1  RT:20.2±0.9  No EX and no IF:20 ± 1 | TRE+RT:26.8 ± 1.6  TRE:26.3±1.4  RT:26.7±01.6  No EX and no IF:27.3±3.4 | Obesity | Participants in the TRE trials restricted to an eating window of 10-hour/day for 8-week. During fasting hours, only water was permitted | RT | Resistance training (systemic) exercises Upper limbs: bench press, high pull down, dumbbell bending; Lower limbs: leg lift, sitting leg bend lift, leg extension. Intensity: 60-70% 1RM, 8-12 reps per set. | 3 | 45 | 8 |
| Haganes et al.2025 | TRE+HIIT  TRE  HIIT  No EX and no IF | TRE+HIIT:32(32/0)  TRE:33(33/0)  HIIT:33(33/0)  No EX and no IF:33(33/0) | TRE+HIIT:37.3±5.7  TRE:36.2±5.9  HIIT:34.9±7.0  No EX and no IF:36.4±6.2 | TRE+HIIT:31.4±4.0  TRE:31.8±3.3  HIIT:32.5±4.5  CON:33.1±4.2 | Obesity | Ad libitum eating within a ≤10-h window, ending by 20:00 h; only non-energy beverages during fasting | HIIT | HIIT. Content: Three sessions per week, two of which were 4×4 min (90 to 95% of maximum heart rate) and one of which was 10×1 min (maximum tolerated intensity). Duration and cycle: Each training session was completed on a treadmill or bicycle for 7 weeks (total 21 sessions). | 3 | 42 | 7 |
| Harvie et al.2025 | IER+RT  RT | IER+RT:35(35/0)  RT:33(33/0) | IER+RT:57.5±10.6  RT:58.5±9.2 | IER+RT:29.2  RT:30 | Obesity | A low-energy (~ 1000 kcal/ day), low-carbohydrate (50g/ day) diet for 2 days per week (for 2 consecutive days before chemotherapy), followed by a Mediterranean diet (25% total weekly energy restriction) for the remaining 5 days. | RT | RE programme adapted from Cormie et al., including exercises for lower limbs, upper limbs, and trunk.  Content: 5–10 standard exercises (e.g., seated/lying adaptations for safety), with online videos (Physiotec) and paper instructions. | 3 | 45 | 10 |
| Hofstätter et al.2025 | TRE+AE  AE | TRE+AE:14(13/6)  AE:10(1/4) | TRE+AE:24.2±3.4  AE:24.8±5.1 | TRE+AE:22.3±1.8  AE:24.2±3.3 | Undergraduate | A 16-h fast (8-h feeding window) was used to train complete fasting | AE | Aerobic cycling (Fatmax training). Content: 60 min each session at an intensity of 90%-100% of the individual Fatmax heart rate interval. | 3 | 60 | 8 |
| Miladi et al.2025 | TRE+AE+RT  TRE+AE+RT  TRE  No EX and no IF | TRE+AE+RT:15(15/0)  TRE+AE+RT:15(15/0)  TRE:15(15/0)  No EX and no IF:16(16/0) | TRE+AE+RT:31.8±10.76  TRE+AE+RT:30.60±7.94  TRE:27.93±9.79  No EX and no IF:36.25±11.52 | TRE+AE+RT:33.5±5.53  TRE+AE+RT:34.37±7.09  TRE:32.71±5.15  No EX and no IF:33.66±6.18 | Obesity | The feeding window was 8 hours (ETRE: 8:00-16:00, LTRE: 12:00-20:00). The fasting window was 16 hours (16:00 to 8:00 in ETRE-PA group and 20:00 to 12:00 in LTRE-PA/LTRE group). | AE+RT | Aerobic training: progressive intensity (50-75% peak heart rate), using auditory rhythms (135-152 BPM) Resistance training: circuit training (hand weights, instruments, body weights). | 3 | 70 | 12 |
| Valenzano et al.2025 | TRE+HIIT  HIIT | TRE+HIIT:15(15/0)  HIIT:15(15/0) | 57.5 ± 6.5 | 27 | Healthy postmenopausal women | 16:8 fasting (eating window: 7:00-15:00), only drinking water or unsweetened tea/coffee during fasting, no calorie restriction during eating, but a balanced diet is recommended | HIIT | Content: Each 40 min session consisted of 30 s of all-out exercise (e.g., sprint, bobbi jump) and 30 s of rest in 3 sets ×10 sessions. Intensity: Increase progressively from 75% to 85% maximum heart rate (HRmax). | 3 | 40 | 8 |
| Yu et al.2025 | TRE+RT  TRE+RT  RT | TRE+RT:8(8/0)  TRE+RT:8(8/0)  RT:8(8/0) | TRE+RT:23.3 ± 0.89  TRE+RT:24.1 ± 2.10  RT:22.1 ± 2.53 | TRE+RT:21.1  TRE+RT:20.5  RT:20.8 | Healthy | eTRE group: eating window from 8:00 AM to 2:00 PM (6 hours), allowed to float for ±30 minutes. dTRE group: food intake window was 12:00 pm-6:00 PM (6 h), allowed to float for ±30 min. | RT | Resistance training (knee-supported push-ups).  Protocol: 4 sets × 10 reps (2s eccentric/1s concentric), 90s rest between sets. | 3 | 6.5 | 8 |

Abbreviations: AE = aerobic exercise; RT = resistance exercise; BMI = body mass index; HIIT = high-intensity interval training; AE(M) = AE moderate intensity; AE(MV) = AE moderate to vigorous intensity; HRmax = maximum heart rate; RIF = Ramadan-Intermittent Fasting; 5:2 = 5:2 diet; ADF = alternate-day fasting; NR = not available/not reported; TRE = time-restricted eating; CR = calorie restrict; CON = no fasting and no exercise; Mos = months; IER = intermittent energy restriction; Fre = frequency; Sess = session; VO2max/VO2peak = maximum/peak oxygen uptake.

**Supplementary Table 4 (Primary pooled effect sizes for the outcomes)**

| **Outcome** | **K** | **Hedges' *g*** | **95%CI** | ***p*** | ***I*2** | **Prediction interval** | **Power** | **GRADE** |
| --- | --- | --- | --- | --- | --- | --- | --- | --- |
| Fat free mass | 45 | 0.0072 | [-0.16, 0.17] | 0.93 | 6% | [-0.26, 0.27] | 98.5% | **⨁⨁◯◯** |
| Lean body mass | 43 | -0.07 | [-0.21, 0.06] | 0.28 | 0% | [-0.21, 0.06] | 18.6% | **⨁⨁◯◯** |
| HDL | 62 | 0.09 | [-0.05, 0.22] | 0.20 | 18% | [-0.30, 0.47] | 25.2% | **⨁◯◯◯** |
| HbA1c | 21 | -0.06 | [-0.24, 0.13] | 0.54 | 16% | [-0.37, 0.26] | 9.2% | **⨁◯◯◯** |
| SBP | 44 | -0.08 | [-0.22, 0.07] | 0.29 | 23% | [-0.45, 0.29] | 18.2% | **⨁◯◯◯** |
| DBP | 44 | -0.02 | [-0.15, 0.10] | 0.71 | 0% | [-0.15, 0.10] | 6.5% | **⨁◯◯◯** |
| Heart rate | 29 | -0.05 | [-0.26, 0.16] | 0.63 | 23% | [-0.46, 0.36] | 7.5% | **⨁⨁◯◯** |
| Adiponection | 18 | 0.17 | [-0.04, 0.37] | 0.10 | 16% | [-0.21, 0.54] | 37.1% | **⨁⨁◯◯** |
| Leptin | 21 | -0.25 | [-0.54, 0.04] | 0.08 | 16% | [-0.21, 0.54] | 40.8% | **⨁⨁◯◯** |
| CRP | 16 | -0.04 | [-0.25, 0.17] | 0.70 | 0% | [-0.25, 0.17] | 6.6% | **⨁⨁◯◯** |
| TNF-α | 7 | -0.10 | [-0.98, 0.77] | 0.78 | 67% | [-1.88, 1.67] | 5.7% | **⨁◯◯◯** |
| Hand grip | 8 | 0.19 | [-0.19, 0.58] | 0.28 | 4% | [-0.24, 0.62] | 17.5% | **⨁⨁◯◯** |
| Bench press | 43 | 0.04 | [-0.15, 0.24] | 0.65 | 0% | [-0.15, 0.24] | 7.3% | **⨁⨁◯◯** |
| Leg press | 32 | 0.05 | [-0.22, 0.33] | 0.70 | 33% | [-0.62, 0.73] | 6.6% | **⨁⨁◯◯** |
| Jump height | 15 | 0.15 | [-0.46, 0.76] | 0.61 | 64% | [-1.15, 1.45] | 7.7% | **⨁◯◯◯** |

Abbreviations: Hedge’s g: the effect size indicators used in the pooled; *I*^2^: quantitative indicators of heterogeneity; K: the total number of effects included in the pooled effect size; p value: statistically significant p values for pooled results; Power: statistical power for pooled effect size. 95%CI = 95% confidence interval; GRADE = Grading of Recommendations Assessment, Development, and Evaluation (a system for evaluating the quality of evidence and strength of recommendations). ⨁OOO: Very low; ⨁⨁OO: Low; ⨁⨁⨁O: Moderate; ⨁⨁⨁⨁: High.

HDL: high density lipoprotein; HbA1c: Hemoglobin A1c; SBP: systolic blood pressure; DBP: diastolic blood pressure; CRP: C-reactive protein; TNF-α: tumor necrosis factor-alpha.

**Supplementary Table 5 (Subgroup analyses results)**

| **No. of studies** | **K** | **Hedges' g** | **95%CI** | ***p*** | ***p*_subgroup_** |
| --- | --- | --- | --- | --- | --- |
| **BMI** | | | | | |
| **Subgroup = sex** |  |  |  |  | 0.33 |
| Mixed | 28 | -0.18 | (-0.39, 0.02) | 0.08 |  |
| Male | 10 | -0.43 | (-0.77, -0.08) | 0.02 |  |
| Female | 19 | -0.38 | (-0.62, -0.13) | <0.01 |  |
| **Subgroup = population** |  |  |  |  | 0.23 |
| Obese | 45 | -0.34 | (-0.50, -0.18) | <0.01 |  |
| Non-obese | 12 | -0.13 | (-0.43, 0.18) | 0.41 |  |
| **Subgroup = age** |  |  |  |  |  |
| ≤40 | 33 | -0.39 | (-0.57, -0.21) | <0.01 | 0.11 |
| >40 | 24 | -0.17 | (-0.37, 0.03) | 0.09 |  |
| **Subgroup = EX type** |  |  |  |  | 0.32 |
| Aerobic exercise (moderate) | 22 | -0.17 | (-0.39, 0.05) | 0.13 |  |
| Aerobic exercise (moderate to vigorous) | 3 | 0.09 | (-0.53, 0.71) | 0.78 |  |
| High-intensity interval training | 8 | -0.33 | (-0.67, 0.01) | 0.05 |  |
| Resistance exercise | 12 | -0.45 | (-0.77, -0.12) | <0.01 |  |
| Concurrent exercise | 12 | -0.46 | (-0.79, -0.13) | <0.01 |  |
| **Subgroup=Control group** |  |  |  |  | **0.01** |
| Intermittent fasting | 14 | -0.10 | (-0.33, 0.13) | 0.38 |  |
| Exercise | 29 | -0.25 | (-0.43, -0.07) | 0.01 |  |
| No EX and no IF | 14 | -0.60 | (-0.85, -0.35) | <0.01 |  |
| **Subgroup = IF type** |  |  |  |  | 0.64 |
| Alternate-day fasting | 10 | -0.45 | (-0.80, -0.10) | 0.01 |  |
| Time-restricted eating | 19 | -0.39 | (-0.65, -0.12) | 0.01 |  |
| Ramadan intermittent fasting | 16 | -0.18 | (-0.46, 0.10) | 0.21 |  |
| 5:2 diet | 6 | -0.17 | (-0.60, 0.26) | 0.45 |  |
| Intermittent energy restriction | 6 | -0.18 | (-0.63, 0.26) | 0.41 |  |
| **Body fat (%)** |  |  |  |  |  |
| **Subgroup = sex** |  |  |  |  | 0.17 |
| Mixed | 22 | -0.09 | (-0.23, 0.05) | 0.21 |  |
| Male | 24 | -0.28 | (-0.43, -0.13) | <0.01 |  |
| Female | 29 | -0.14 | (-0.27, -0.01) | 0.04 |  |
| **Subgroup = population** |  |  |  |  | 0.82 |
| Obese | 37 | -0.16 | (-0.27, -0.04) | 0.01 |  |
| Non-obese | 38 | -0.18 | (-0.30, -0.05) | 0.01 |  |
| **Subgroup = age** |  |  |  |  | 0.16 |
| ≤40 | 52 | -0.21 | (-0.32, -0.11) | <0.01 |  |
| >40 | 23 | -0.09 | (-0.22, 0.04) | 0.17 |  |
| **Subgroup = EX type** |  |  |  |  | 0.14 |
| Aerobic exercise (moderate) | 25 | -0.05 | (-0.45, 0.02) | 0.07 |  |
| Aerobic exercise (moderate to vigorous) | 3 | -0.05 | (-0.38, -0.09) | <0.01 |  |
| High-intensity interval training | 10 | -0.36 | (-0.60, -0.11) | 0.01 |  |
| Resistance exercise | 27 | -0.24 | (-0.44, 0.33) | 0.78 |  |
| Concurrent exercise | 10 | -0.22 | (-0.18, 0.07) | 0.39 |  |
| **Subgroup=Control group** |  |  |  |  | 0.14 |
| Intermittent fasting | 12 | -0.01 | (-0.23, 0.22) | -0.50 |  |
| Exercise | 51 | -0.17 | (-0.29, -0.06) | -0.06 |  |
| No EX and no IF | 12 | -0.30 | (-0.50, -0.10) | 0.96 |  |
| **Subgroup = IF type** |  |  |  |  | 0.18 |
| Alternate-day fasting | 6 | -0.50 | (-0.88, -0.12) | 0.07 |  |
| Time-restricted eating | 33 | -0.06 | (-0.20, 0.08) | 0.13 |  |
| Ramadan intermittent fasting | 20 | -0.23 | (-0.39, -0.08) | <0.01 |  |
| 5:2 diet | 9 | -0.13 | (-0.30, 0.04) | 0.37 |  |
| Intermittent energy restriction | 7 | -0.22 | (-0.46, 0.02) | 0.01 |  |
| **Fat mass** |  |  |  |  |  |
| **Subgroup = sex** |  |  |  |  | 0.91 |
| Mixed | 31 | -0.16 | (-0.26, -0.05) | <0.01 |  |
| Male | 14 | -0.20 | (-0.40, 0.01) | 0.06 |  |
| Female | 38 | -0.15 | (-0.25, -0.05) | <0.01 |  |
| **Subgroup = population** |  |  |  |  | 0.71 |
| Obese | 52 | -0.17 | (-0.24, 0.09) | <0.01 |  |
| Non-obese | 31 | -0.13 | (-0.28, 0.01) | 0.06 |  |
| **Subgroup = age** |  |  |  |  | 0.83 |
| ≤40 | 60 | -0.16 | (-0.25, -0.08) | <0.01 |  |
| >40 | 23 | -0.15 | (-0.26, -0.03) | 0.01 |  |
| **Subgroup = EX type** |  |  |  |  | 0.75 |
| Aerobic exercise (moderate) | 22 | -0.15 | (-0.27, -0.03) | 0.01 |  |
| Aerobic exercise (moderate to vigorous) | 3 | 0.09 | (-0.29, 0.47) | 0.65 |  |
| High-intensity interval training | 19 | -0.17 | (-0.29, -0.04) | 0.01 |  |
| Resistance exercise | 28 | -0.19 | (-0.34, -0.04) | 0.01 |  |
| Concurrent exercise | 11 | -0.17 | (-0.39, 0.05) | 0.13 |  |
| **Subgroup=Control group** |  |  |  |  | 0.47 |
| Intermittent fasting | 14 | -0.15 | (-0.31, 0.01) | 0.07 |  |
| Exercise | 57 | -0.13 | (-0.22, -0.04) | <0.01 |  |
| No EX and no IF | 12 | -0.25 | (-0.42, -0.08) | 0.01 |  |
| **Subgroup = IF type** |  |  |  |  | 0.81 |
| Alternate-day fasting | 9 | -0.19 | (-0.34, -0.04) | 0.99 |  |
| Time-restricted eating | 53 | -0.16 | (-0.25, -0.06) | 0.20 |  |
| Ramadan intermittent fasting | 11 | -0.14 | (-0.35, 0.08) | 0.21 |  |
| 5:2 diet | 5 | -0.19 | (-0.48, 0.10) | <0.01 |  |
| Intermittent energy restriction | 5 | 0.0024 | (-0.27, 0.28) | 0.02 |  |
| **Fat free mass** |  |  |  |  |  |
| **Subgroup = sex** |  |  |  |  | 0.26 |
| Mixed | 13 | 0.01 | (-0.19, 0.21) | 0.92 |  |
| Male | 9 | -0.17 | (-0.43, 0.10) | 0.21 |  |
| Female | 19 | 0.09 | (-0.08, 0.27) | 0.29 |  |
| **Subgroup = population** |  |  |  |  | 0.83 |
| Obese | 8 | 0.04 | (-0.14, 0.14) | 0.99 |  |
| Non-obese | 33 | <0.01 | (-0.27, 0.34) | 0.81 |  |
| **Subgroup = age** |  |  |  |  | 0.47 |
| ≤40 | 32 | -0.02 | (-0.18, 0.13) | 0.75 |  |
| >40 | 9 | 0.08 | (-0.16, 0.31) | 0.52 |  |
| **Subgroup = EX type** |  |  |  |  | 0.29 |
| Aerobic exercise (moderate) | 5 | 0.08 | (-0.24, 0.39) | 0.97 |  |
| Aerobic exercise (moderate to vigorous) | 3 | 0.09 | (-0.31, 0.49) | 0.26 |  |
| High-intensity interval training | 4 | 0.31 | (-0.03, 0.66) | 0.07 |  |
| Resistance exercise | 24 | -0.10 | (-0.27, 0.07) | 0.65 |  |
| Concurrent exercise | 5 | 0.01 | (-0.29, 0.30) | 0.63 |  |
| **Subgroup=Control group** |  |  |  |  | **0.05** |
| Intermittent fasting | 4 | 0.38 | (0.06, 0.71) | 0.02 |  |
| Exercise | 34 | -0.06 | (-0.19, 0.08) | 0.39 |  |
| No EX and no IF | 3 | 0.07 | (-0.32, 0.45) | 0.73 |  |
| **Subgroup = IF type** |  |  |  |  | 0.99 |
| Alternate-day fasting | 3 | 0.14 | (-0.38, 0.65) | 0.98 |  |
| Time-restricted eating | 25 | -0.03 | (-0.23, 0.18) | 0.96 |  |
| Ramadan intermittent fasting | 5 | -0.01 | (-0.52, 0.50) | 0.97 |  |
| 5:2 diet | 3 | 0.01 | (-0.48, 0.51) | 0.79 |  |
| Intermittent energy restriction | 5 | -0.0046 | (-0.36, 0.35) | 0.60 |  |
| **Lean body mass** |  |  |  |  |  |
| **Subgroup = sex** |  |  |  |  | 0.38 |
| Mixed | 14 | -0.04 | (-0.24, 0.16) | 0.69 |  |
| Male | 13 | -0.20 | (-0.40, -0.0021) | 0.05 |  |
| Female | 9 | -0.20 | (-0.24, 0.20) | 0.86 |  |
| **Subgroup = population** |  |  |  |  | 0.22 |
| Obese | 21 | -0.03 | (-0.18, 0.12) | 0.65 |  |
| Non-obese | 15 | -0.18 | (-0.37, 0.01) | 0.06 |  |
| **Subgroup = age** |  |  |  |  | 0.42 |
| ≤40 | 24 | -0.13 | (-0.28, 0.02) | 0.78 |  |
| >40 | 12 | -0.03 | (-0.22, 0.17) | 0.09 |  |
| **Subgroup = EX type** |  |  |  |  | 0.32 |
| Aerobic exercise (moderate) | 15 | -0.02 | (-0.19， 0.15) | 0.79 |  |
| Aerobic exercise (moderate to vigorous) | 3 | 0.17 | (-0.35, 0.69) | 0.51 |  |
| High-intensity interval training | 2 | -0.15 | (-0.73, 0.44) | 0.62 |  |
| Resistance exercise | 9 | -0.28 | (-0.51, -0.06) | 0.02 |  |
| Concurrent exercise | 7 | -0.03 | (-0.33, 0.26) | 0.82 |  |
| **Subgroup=Control group** |  |  |  |  | 0.38 |
| Intermittent fasting | 14 | -0.04 | (-0.24, 0.16) | 0.69 |  |
| Exercise | 13 | -0.20 | (-0.40, -0.0021) | 0.05 |  |
| No EX and no IF | 9 | -0.02 | (-0.24, 0.20) | 0.86 |  |
| **Subgroup = IF type** |  |  |  |  | 0.84 |
| Alternate-day fasting | - | - | - | - |  |
| Time-restricted eating | 13 | -0.04 | (-0.25, 0.16) | 0.62 |  |
| Ramadan intermittent fasting | 21 | -0.11 | (-0.26, 0.04) | 0.13 |  |
| 5:2 diet | 2 | -0.15 | (-0.73, 0.44) | 0.67 |  |
| Intermittent energy restriction | - | - | - | - |  |
| **Waist circumference** |  |  |  |  |  |
| **Subgroup = sex** |  |  |  |  | 0.35 |
| Mixed | 42 | -0.19 | (-0.27, 0.05) | <0.01 |  |
| Male | - | - | - | - |  |
| Female | 26 | -0.11 | (-0.28, -0.11) | 0.18 |  |
| **Subgroup = population** |  |  |  |  | 0.39 |
| Obese | 64 | -0.18 | (-0.26, -0.10) | <0.01 |  |
| Non-obese | 4 | -0.01 | (-0.40,0.38) | 0.98 |  |
| **Subgroup = age** |  |  |  |  | 0.36 |
| ≤40 | 38 | -0.22 | (-0.34, -0.09) | <0.01 |  |
| >40 | 30 | -0.15 | (-0.24, -0.05) | <0.01 |  |
| **Subgroup = EX type** |  |  |  |  | 0.45 |
| Aerobic exercise (moderate) | 30 | -0.15 | (-0.24, -0.05) | 0.23 |  |
| Aerobic exercise (moderate to vigorous) | 2 | 0.15 | (-0.32, 0.62) | 0.08 |  |
| High-intensity interval training | 18 | -0.28 | (-0.45, -0.10) | <0.01 |  |
| Resistance exercise | 13 | -0.20 | (-0.44, 0.03) | 0.53 |  |
| Concurrent exercise | 5 | -0.21 | (-0.55, 0.13) | <0.01 |  |
| **Subgroup=Control group** |  |  |  |  | 0.72 |
| Intermittent fasting | 15 | -0.11 | (-0.30, 0.09) | 0.29 |  |
| Exercise | 43 | -0.18 | (-0.27, -0.09) | <0.01 |  |
| No EX and no IF | 10 | -0.22 | (-0.46, 0.02) | 0.07 |  |
| **Subgroup = IF type** |  |  |  |  | 0.77 |
| Alternate-day fasting | 9 | -0.21 | (-0.38, -0.05) | 0.01 |  |
| Time-restricted eating | 24 | -0.13 | (-0.30, 0.03) | 0.11 |  |
| Ramadan intermittent fasting | 19 | -0.11 | (-0.30, 0.09) | 0.28 |  |
| 5:2 diet | 10 | -0.30 | (-0.56, -0.04) | 0.03 |  |
| Intermittent energy restriction | 6 | -0.20 | (-0.50, 0.10) | 0.18 |  |
| **Visceral adipose tissue** |  |  |  |  |  |
| **Subgroup = sex** |  |  |  |  | 0.74 |
| Mixed | 11 | -0.24 | (-0.46, -0.02) | 0.04 |  |
| Male | 3 | -0.40 | (-0.92, 0.11) | 0.12 |  |
| Female | 18 | -0.21 | (-0.34, -0.07) | <0.01 |  |
| **Subgroup = population** |  |  |  |  | 0.46 |
| Obese | 29 | -0.21 | (-0.33, -0.10) | <0.01 |  |
| Non-obese | 3 | -0.40 | (-0.92, 0.11) | 0.12 |  |
| **Subgroup = age** |  |  |  |  | 0.90 |
| ≤40 | 20 | -0.23 | (-0.36, -0.10) | 0.04 |  |
| >40 | 12 | -0.21 | (-0.41, -0.01) | <0.01 |  |
| **Subgroup = EX type** |  |  |  |  | 0.14 |
| Aerobic exercise (moderate) | 8 | -0.14 | (-0.36, 0.08) | 0.02 |  |
| Aerobic exercise (moderate to vigorous) | - | - | - | - |  |
| High-intensity interval training | 13 | -0.18 | (-0.32, -0.04) | <0.01 |  |
| Resistance exercise | 7 | -0.52 | (-0.88, -0.16) | 0.02 |  |
| Concurrent exercise | 4 | -0.57 | (-1.06, -0.08) | 0.19 |  |
| **Subgroup=Control group** |  |  |  |  | 0.95 |
| Intermittent fasting | 7 | -0.20 | (-0.42, 0.01) | 0.06 |  |
| Exercise | 16 | -0.24 | (-0.42, -0.07) | 0.01 |  |
| No EX and no IF | 9 | -0.22 | (-0.41, -0.03) | 0.03 |  |
| **Subgroup = IF type** |  |  |  |  | 0.27 |
| Alternate-day fasting | -0.79 | -0.79 | (-1.38, -0.19) | 0.01 |  |
| Time-restricted eating | -0.19 | -0.19 | (-0.33, -0.05) | 0.01 |  |
| Ramadan intermittent fasting | -0.22 | -0.22 | (-0.42, -0.02) | 0.03 |  |
| 5:2 diet | -0.34 | -0.34 | (-1.19, 0.51) | 0.41 |  |
| Intermittent energy restriction | - | - | - | - |  |
| **Total cholesterol** |  |  |  |  |  |
| **Subgroup = sex** |  |  |  |  | 0.79 |
| Mixed | 16 | -0.13 | (-0.35, 0.09) | 0.23 |  |
| Male | 19 | -0.21 | (-0.43, 0.02) | 0.07 |  |
| Female | 30 | -0.11 | (-0.30, 0.08) | 0.24 |  |
| **Subgroup = population** |  |  |  |  | 0.24 |
| Obese | 43 | -0.09 | (-0.23, 0.04) | 0.17 |  |
| Non-obese | 22 | -0.24 | (-0.44, -0.03) | 0.02 |  |
| **Subgroup = age** |  |  |  |  | 0.37 |
| ≤40 | 51 | -0.18 | (-0.32, -0.04) | 0.01 |  |
| >40 | 14 | -0.06 | (-0.28, 0.15) | 0.56 |  |
| **Subgroup = EX type** |  |  |  |  | 0.38 |
| Aerobic exercise (moderate) | 15 | -0.06 | (-0.26, 0.14) | 0.47 |  |
| Aerobic exercise (moderate to vigorous) | 3 | 0.24 | (-0.41, 0.89) | 0.02 |  |
| High-intensity interval training | 17 | -0.20 | (-0.45, 0.04) | 0.11 |  |
| Resistance exercise | 18 | -0.32 | (-0.58, -0.06) | 0.47 |  |
| Concurrent exercise | 12 | -0.10 | (-0.39, 0.18) | 0.57 |  |
| **Subgroup=Control group** |  |  |  |  | 0.54 |
| Intermittent fasting | 14 | -0.15 | (-0.33, 0.03) | 0.11 |  |
| Exercise | 39 | -0.17 | (-0.30, -0.03) | 0.02 |  |
| No EX and no IF | 12 | -0.04 | (-0.25, 0.16) | 0.67 |  |
| **Subgroup = IF type** |  |  |  |  | 0.53 |
| Alternate-day fasting | 15 | 0.004 | (-0.26, 0.27) | 0.97 |  |
| Time-restricted eating | 21 | -0.15 | (-0.35, 0.06) | 0.15 |  |
| Ramadan intermittent fasting | 17 | -0.23 | (-0.47, 0.002) | 0.05 |  |
| 5:2 diet | 10 | -0.10 | (-0.38, 0.18) | 0.47 |  |
| Intermittent energy restriction | 2 | -0.50 | (-1.14, 0.13) | 0.12 |  |
| **Triglyceride** |  |  |  |  |  |
| **Subgroup = sex** |  |  |  |  | 0.33 |
| Mixed | 17 | -0.18 | (-0.47, 0.10) | 0.20 |  |
| Male | 17 | -0.42 | (-0.71, -0.12) | 0.01 |  |
| Female | 26 | -0.13 | (-0.40, 0.14) | 0.32 |  |
| **Subgroup = population** |  |  |  |  | 0.28 |
| Obese | 40 | -0.17 | (-0.38, 0.03) | 0.09 |  |
| Non-obese | 20 | -0.36 | (-0.63, -0.08) | 0.01 |  |
| **Subgroup = age** |  |  |  |  | 0.23 |
| ≤40 | 45 | -0.30 | (-0.50, -0.11) | <0.01 |  |
| >40 | 15 | -0.09 | (-0.39, 0.21) | 0.55 |  |
| **Subgroup = EX type** |  |  |  |  | 0.40 |
| Aerobic exercise (moderate) | 15 | -0.11 | (-0.39, 0.17) | 0.43 |  |
| Aerobic exercise (moderate to vigorous) | 2 | 0.29 | (-0.63, 1.22) | 0.53 |  |
| High-intensity interval training | 17 | -0.21 | (-0.56, 0.14) | 0.23 |  |
| Resistance exercise | 18 | -0.44 | (-0.77, -0.10) | 0.01 |  |
| Concurrent exercise | 8 | -0.40 | (-0.85, 0.06) | 0.09 |  |
| **Subgroup=Control group** |  |  |  |  | 0.32 |
| Intermittent fasting | 12 | -0.10 | (-0.36, 0.15) | 0.43 |  |
| Exercise | 36 | -0.27 | (-0.45, -0.08) | 0.01 |  |
| No EX and no IF | 12 | -0.30 | (-0.55, -0.04) | 0.02 |  |
| **Subgroup = IF type** |  |  |  |  | 0.61 |
| Alternate-day fasting | 5 | -0.20 | (-0.72, 0.31) | 0.05 |  |
| Time-restricted eating | 26 | -0.26 | (-0.54, 0.02) | 0.70 |  |
| Ramadan intermittent fasting | 17 | -0.25 | (-0.57, 0.07) | 0.13 |  |
| 5:2 diet | 10 | -0.08 | (-0.49, 0.33) | 0.06 |  |
| Intermittent energy restriction | 2 | -0.86 | (-1.74, 0.02) | 0.43 |  |
| **High density lipoprotein** |  |  |  |  |  |
| **Subgroup = sex** |  |  |  |  | 0.62 |
| Mixed | 18 | 0.07 | (-0.23, 0.37) | 0.63 |  |
| Male | 13 | 0.26 | (-0.09, 0.61) | 0.14 |  |
| Female | 26 | 0.05 | (-0.25, 0.35) | 0.74 |  |
| **Subgroup = population** |  |  |  |  | 0.75 |
| Obese | 38 | 0.09 | (-0.13, 0.32) | 0.41 |  |
| Non-obese | 19 | 0.15 | (-0.15, 0.45) | 0.32 |  |
| **Subgroup = age** |  |  |  |  | 0.65 |
| ≤40 | 44 | 0.14 | (-0.07, 0.36) | 0.20 |  |
| >40 | 13 | 0.05 | (-0.28, 0.38) | 0.75 |  |
| **Subgroup = EX type** |  |  |  |  | 0.99 |
| Aerobic exercise (moderate) | 14 | 0.08 | (-0.25, 0.40) | 0.63 |  |
| Aerobic exercise (moderate to vigorous) | 2 | -0.06 | (-1.09, 0.96) | 0.90 |  |
| High-intensity interval training | 17 | 0.15 | (-0.25, 0.56) | 0.45 |  |
| Resistance exercise | 18 | 0.14 | (-0.25, 0.52) | 0.48 |  |
| Concurrent exercise | 6 | 0.17 | (-0.38, 0.72) | 0.54 |  |
| **Subgroup=Control group** |  |  |  |  | 0.79 |
| Intermittent fasting | 11 | 0.16 | (-0.10, 0.42) | 0.22 |  |
| Exercise | 35 | 0.11 | (-0.08, 0.31) | 0.25 |  |
| No EX and no IF | 11 | 0.07 | (-0.18, 0.32) | 0.59 |  |
| **Subgroup = IF type** |  |  |  |  | 0.45 |
| Alternate-day fasting | 5 | 0.09 | (-0.43, 0.61) | 0.73 |  |
| Time-restricted eating | 26 | 0.27 | (-0.01, 0.56) | 0.06 |  |
| Ramadan intermittent fasting | 17 | -0.07 | (-0.40, 0.26) | 0.69 |  |
| 5:2 diet | 7 | -0.05 | (-0.55, 0.44) | 0.83 |  |
| Intermittent energy restriction | 2 | 0.52 | (-0.37, 1.41) | 0.25 |  |
| **Low Density Lipoprotein** |  |  |  |  |  |
| **Subgroup = sex** |  |  |  |  | 0.65 |
| Mixed | 16 | -0.15 | (-0.34, 0.05) | 0.13 |  |
| Male | 12 | -0.24 | (-0.51, 0.02) | 0.07 |  |
| Female | 31 | -0.10 | (-0.27, 0.07) | 0.24 |  |
| **Subgroup = population** |  |  |  |  | 0.17 |
| Obese | 40 | -0.09 | (-0.21, 0.03) | 0.16 |  |
| Non-obese | 19 | -0.26 | (-0.46, -0.05) | 0.02 |  |
| **Subgroup = age** |  |  |  |  | 0.66 |
| ≤40 | 48 | -0.16 | (-0.30, -0.02) | 0.02 |  |
| >40 | 11 | -0.11 | (-0.32, 0.11) | 0.33 |  |
| **Subgroup = EX type** |  |  |  |  | 0.65 |
| Aerobic exercise (moderate) | 14 | -0.15 | (-0.34, 0.04) | 0.12 |  |
| Aerobic exercise (moderate to vigorous) | 2 | 0.27 | (-0.46, 0.99) | 0.46 |  |
| High-intensity interval training | 17 | -0.09 | (-0.32, 0.14) | 0.44 |  |
| Resistance exercise | 18 | -0.27 | (-0.53, -0.01) | 0.04 |  |
| Concurrent exercise | 8 | -0.17 | (-0.52, 0.19) | 0.35 |  |
| **Subgroup=Control group** |  |  |  |  | 0.08 |
| Intermittent fasting | 12 | -0.15 | (-0.36, 0.05) | 0.14 |  |
| Exercise | 33 | -0.20 | (-0.35, -0.06) | 0.01 |  |
| No EX and no IF | 14 | 0.05 | (-0.14, 0.24) | 0.57 |  |
| **Subgroup = IF type** |  |  |  |  | 0.69 |
| Alternate-day fasting | 4 | -0.18 | (-0.53, 0.17) | 0.31 |  |
| Time-restricted eating | 29 | -0.09 | (-0.26, 0.09) | 0.32 |  |
| Ramadan intermittent fasting | 17 | -0.25 | (-0.47, -0.03) | 0.03 |  |
| 5:2 diet | 7 | -0.05 | (-0.35, 0.25) | 0.73 |  |
| Intermittent energy restriction | 2 | -0.34 | (-0.94, 0.25) | 0.25 |  |
| **Fasting glucose** |  |  |  |  |  |
| **Subgroup = sex** |  |  |  |  | 0.18 |
| Mixed | 13 | -0.25 | (-0.54, 0.04) | 0.09 |  |
| Male | 19 | -0.43 | (-0.67, -0.18) | <0.01 |  |
| Female | 26 | -0.11 | (-0.34, 0.12) | 0.35 |  |
| **Subgroup = population** |  |  |  |  | 0.98 |
| Obese | 36 | -0.26 | (-0.46, -0.05) | 0.02 |  |
| Non-obese | 22 | -0.26 | (-0.51, -0.02) | 0.04 |  |
| **Subgroup = age** |  |  |  |  | 0.87 |
| ≤40 | 45 | -0.27 | (-0.45, -0.08) | 0.01 |  |
| >40 | 13 | -0.24 | (-0.54, 0.06) | 0.12 |  |
| **Subgroup = EX type** |  |  |  |  | 0.95 |
| Aerobic exercise (moderate) | 12 | -0.19 | (-0.48, 0.11) | 0.22 |  |
| Aerobic exercise (moderate to vigorous) | 2 | -0.21 | (-1.12, 0.70) | 0.65 |  |
| High-intensity interval training | 15 | -0.23 | (-0.61, 0.15) | 0.24 |  |
| Resistance exercise | 21 | -0.34 | (-0.65, -0.03) | 0.03 |  |
| Concurrent exercise | 8 | -0.34 | (-0.79, 0.11) | 0.13 |  |
| **Subgroup=Control group** |  |  |  |  | 0.10 |
| Intermittent fasting | 10 | -0.21 | (-0.46, 0.04) | 0.10 |  |
| Exercise | 36 | -0.21 | (-0.38, -0.04) | 0.02 |  |
| No EX and no IF | 12 | -0.46 | (-0.70, -0.22) | <0.01 |  |
| **Subgroup = IF type** |  |  |  |  | 0.34 |
| Alternate-day fasting | 8 | -0.55 | (-0.93, -0.16) | 0.01 |  |
| Time-restricted eating | 25 | -0.26 | (-0.50, -0.02) | 0.03 |  |
| Ramadan intermittent fasting | 17 | -0.19 | (-0.47, 0.09) | 0.17 |  |
| 5:2 diet | 8 | -0.05 | (-0.47, 0.37) | 0.81 |  |
| Intermittent energy restriction | - | - | - | - |  |
| **Insulin** |  |  |  |  |  |
| **Subgroup = sex** |  |  |  |  | 0.22 |
| Mixed | 12 | -0.26 | (-0.87, 0.35) | 0.40 |  |
| Male | 9 | -0.93 | (-1.57, -0.29) | 0.01 |  |
| Female | 14 | -0.27 | (-0.85, 0.31) | 0.34 |  |
| **Subgroup = population** |  |  |  |  | 0.53 |
| Obese | 24 | -0.56 | (-1.04, -0.08) | 0.02 |  |
| Non-obese | 11 | -0.32 | (-0.93, 0.29) | 0.30 |  |
| **Subgroup = age** |  |  |  |  | 0.84 |
| ≤40 | 23 | -0.50 | (-0.97, -0.03) | 0.04 |  |
| >40 | 12 | -0.41 | (-1.08, 0.25) | 0.21 |  |
| **Subgroup = EX type** |  |  |  |  | 0.89 |
| Aerobic exercise (moderate) | 5 | -0.11 | (-1.08, 0.87) | 0.83 |  |
| Aerobic exercise (moderate to vigorous) | 3 | -0.16 | (-1.73, 1.41) | 0.84 |  |
| High-intensity interval training | 5 | -0.48 | (-1.59, 0.64) | 0.39 |  |
| Resistance exercise | 16 | -0.64 | (-1.31, 0.03) | 0.06 |  |
| Concurrent exercise | 6 | -0.60 | (-1.57, 0.38) | 0.22 |  |
| **Subgroup=Control group** |  |  |  |  | 0.33 |
| Intermittent fasting | 6 | -0.50 | (-0.97, -0.03) | 0.04 |  |
| Exercise | 23 | -0.42 | (-0.80, -0.04) | 0.03 |  |
| No EX and no IF | 6 | -0.71 | (-1.20, -0.22) | 0.01 |  |
| **Subgroup = IF type** |  |  |  |  | 0.26 |
| Alternate-day fasting | 8 | -1.17 | (-1.99, -0.35) | 0.01 |  |
| Time-restricted eating | 14 | -0.39 | (-0.90, 0.11) | 0.12 |  |
| Ramadan intermittent fasting | 7 | -0.20 | (-1.15, 0.75) | 0.68 |  |
| 5:2 diet | 6 | -0.05 | (-1.01, 0.90) | 0.91 |  |
| Intermittent energy restriction | - | - | - | - |  |
| **HOMA-IR** |  |  |  |  |  |
| **Subgroup = sex** |  |  |  |  | 0.96 |
| Mixed | 14 | -0.39 | (-0.95, 0.17) | 0.17 |  |
| Male | 5 | -0.52 | (-1.38, 0.35) | 0.23 |  |
| Female | 12 | -0.39 | (-1.06, 0.28) | 0.24 |  |
| **Subgroup = population** |  |  |  |  | 0.68 |
| Obese | 24 | -0.46 | (-0.89, -0.04) | 0.03 |  |
| Non-obese | 7 | -0.29 | (-1.00, 0.42) | 0.41 |  |
| **Subgroup = age** |  |  |  |  | 0.71 |
| ≤40 | 18 | -0.36 | (-0.84, 0.12) | 0.14 |  |
| >40 | 13 | -0.50 | (-1.07, 0.08) | 0.09 |  |
| **Subgroup = EX type** |  |  |  |  | 0.54 |
| Aerobic exercise (moderate) | 6 | -0.08 | (-0.77, 0.62) | 0.83 |  |
| Aerobic exercise (moderate to vigorous) | 3 | 0.00 | (-1.31, 1.30) | 1.00 |  |
| High-intensity interval training | 7 | -0.43 | (-1.20, 0.34) | 0.26 |  |
| Resistance exercise | 9 | -0.62 | (-1.42, 0.19) | 0.13 |  |
| Concurrent exercise | 6 | -0.98 | (-1.94, -0.02) | 0.05 |  |
| **Subgroup=Control group** |  |  |  |  | 0.14 |
| Intermittent fasting | 8 | -0.49 | (-0.90, -0.08) | 0.02 |  |
| Exercise | 17 | -0.32 | (-0.68, 0.04) | 0.08 |  |
| No EX and no IF | 6 | -0.70 | (-1.15, -0.24) | <0.01 |  |
| **Subgroup = IF type** |  |  |  |  | 0.35 |
| Alternate-day fasting | 10 | -0.89 | (-1.54, -0.25) | 0.01 |  |
| Time-restricted eating | 7 | -0.18 | (-0.84, 0.49) | 0.59 |  |
| Ramadan intermittent fasting | 7 | -0.27 | (-1.15, 0.62) | 0.54 |  |
| 5:2 diet | 7 | -0.17 | (-0.93, 0.58) | 0.64 |  |
| Intermittent energy restriction | - | - | - | - |  |
| **HbA1C** |  |  |  |  |  |
| **Subgroup = sex** |  |  |  |  | **0.05** |
| Mixed | 7 | 0.10 | (-0.09, 0.30) | 0.28 |  |
| Male | 1 | -0.15 | (-0.49, 0.18) | 0.34 |  |
| Female | 9 | -0.23 | (-0.41, -0.06) | 0.01 |  |
| **Subgroup = population** |  |  |  |  | 0.69 |
| Obese | 6 | -0.0077 | (-0.35, 0.33) | 0.96 |  |
| Non-obese | 11 | -0.09 | (-0.33, 0.16) | 0.45 |  |
| **Subgroup = age** |  |  |  |  | 0.28 |
| ≤40 | 11 | -0.17 | (-0.41, 0.08) | 0.16 |  |
| >40 | 6 | 0.0049 | (-0.22, 0.23) | 0.96 |  |
| **Subgroup = EX type** |  |  |  |  | 0.25 |
| Aerobic exercise (moderate) | 5 | -0.03 | (-0.26, 0.20) | 0.78 |  |
| Aerobic exercise (moderate to vigorous) | - | - | - | - |  |
| High-intensity interval training | 11 | -0.16 | (-0.41, 0.08) | 0.18 |  |
| Resistance exercise | - | - | - | - | - |
| Concurrent exercise | 1 | 0.59 | (-0.36, 1.54) | 0.20 |  |
| **Subgroup=Control group** |  |  |  |  | 0.30 |
| Intermittent fasting | 5 | 0.04 | (-0.23, 0.31) | 0.75 |  |
| Exercise | 7 | -0.17 | (-0.40, 0.07) | 0.15 |  |
| No EX and no IF | 5 | 0.004 | (-0.32, 0.33) | 0.98 |  |
| **Subgroup = IF type** |  |  |  |  | 0.11 |
| Alternate-day fasting | 1 | -0.15 | (-0.50, 0.18) | 0.34 |  |
| Time-restricted eating | 10 | -0.20 | (-0.38, -0.03) | 0.03 |  |
| Ramadan intermittent fasting | 2 | -0.19 | (-0.67, 0.28) | 0.40 |  |
| 5:2 diet | 4 | 0.14 | (-0.09, 0.36) | 0.20 |  |
| Intermittent energy restriction | - | - | - | - |  |
| **Systolic Blood Pressure** |  |  |  |  |  |
| **Subgroup = sex** |  |  |  |  | 0.73 |
| Mixed | 19 | -0.05 | (-0.16, 0.07) | 0.42 |  |
| Male | 1 | -0.25 | (-0.98, 0.47) | 0.48 |  |
| Female | 19 | -0.10 | (-0.22, 0.03) | 0.13 |  |
| **Subgroup = population** |  |  |  |  | 0.21 |
| Obese | 35 | -0.06 | (-0.14, 0.03) | 0.18 |  |
| Non-obese | 4 | -0.30 | (-0.68, 0.07) | 0.11 |  |
| **Subgroup = age** |  |  |  |  | 0.71 |
| ≤40 | 25 | -0.09 | (-0.20, 0.03) | 0.15 |  |
| >40 | 14 | -0.06 | (-0.17, 0.06) | 0.36 |  |
| **Subgroup = EX type** |  |  |  |  | 0.62 |
| Aerobic exercise (moderate) | 15 | -0.08 | (-0.20, 0.04) | 0.18 |  |
| Aerobic exercise (moderate to vigorous) | 2 | 0.01 | (-0.46, 0.49) | 0.96 |  |
| High-intensity interval training | 15 | -0.02 | (-0.16, 0.12) | 0.74 |  |
| Resistance exercise | 6 | -0.24 | (-0.55, 0.07) | 0.13 |  |
| Concurrent exercise | 1 | -0.46 | (-1.32, 0.41) | 0.29 |  |
| **Subgroup=Control group** |  |  |  |  | 0.43 |
| Intermittent fasting | 9 | -0.03 | (-0.23, 0.17) | 0.78 |  |
| Exercise | 18 | -0.01 | (-0.13, 0.11) | 0.88 |  |
| No EX and no IF | 8 | -0.14 | (-0.32, 0.03) | 0.10 |  |
| **Subgroup = IF type** |  |  |  |  | 0.93 |
| Alternate-day fasting | 4 | -0.07 | (-0.33, 0.19) | 0.57 |  |
| Time-restricted eating | 20 | -0.09 | (-0.23, 0.06) | 0.25 |  |
| Ramadan intermittent fasting | 3 | -0.10 | (-0.49, 0.29) | 0.60 |  |
| 5:2 diet | 6 | 0.01 | (-0.18, 0.21) | 0.88 |  |
| Intermittent energy restriction | 2 | 0.01 | (-0.48, 0.51) | 0.96 |  |
| **Diastolic Blood Pressure** |  |  |  |  |  |
| **Subgroup = sex** |  |  |  |  | **0.01** |
| Mixed | 19 | 0.03 | (-0.08, 0.15) | -0.08 |  |
| Male | 1 | -0.72 | (-1.46, 0.03) | 0.06 |  |
| Female | 20 | -0.22 | (-0.34, -0.09) | <0.01 |  |
| **Subgroup = population** |  |  |  |  | **0.02** |
| Obese | 36 | -0.04 | (-0.16, 0.08) | 0.50 |  |
| Non-obese | 4 | -0.59 | (-1.01, -0.16) | <0.01 |  |
| **Subgroup = age** |  |  |  |  | 0.06 |
| ≤40 | 26 | -0.18 | (-0.32, -0.03) | 0.02 |  |
| >40 | 14 | 0.02 | (-0.13, 0.18) | 0.75 |  |
| **Subgroup = EX type** |  |  |  |  | 0.50 |
| Aerobic exercise (moderate) | 15 | -0.06 | (-0.25, 0.14) | 0.57 |  |
| Aerobic exercise (moderate to vigorous) | 2 | 0.08 | (-0.50, 0.66) | 0.78 |  |
| High-intensity interval training | 16 | -0.10 | (-0.33, 0.14) | 0.40 |  |
| Resistance exercise | 6 | -0.29 | (-0.68, 0.10) | 0.14 |  |
| Concurrent exercise | 1 | 0.55 | (-0.38, 1.47) | 0.24 |  |
| **Subgroup=Control group** |  |  |  |  | 0.43 |
| Intermittent fasting | 10 | -0.04 | (-0.25, 0.18) | 0.73 |  |
| Exercise | 20 | -0.06 | (-0.21, 0.09) | 0.45 |  |
| No EX and no IF | 10 | -0.17 | (-0.37, 0.02) | 0.08 |  |
| **Subgroup = IF type** |  |  |  |  | **0.02** |
| Alternate-day fasting | 4 | 0.15 | (-0.09, 0.40) | 0.20 |  |
| Time-restricted eating | 25 | -0.20 | (-0.32, -0.08) | <0.01 |  |
| Ramadan intermittent fasting | 3 | -0.35 | (-0.72, 0.02) | 0.06 |  |
| 5:2 diet | 6 | 0.04 | (-0.12,0.20) | 0.63 |  |
| Intermittent energy restriction | 2 | 0.08 | (-0.40, 0.56) | 0.74 |  |
| **Heart rate** |  |  |  |  |  |
| **Subgroup = sex** |  |  |  |  | 0.27 |
| Mixed | 10 | -0.12 | (-0.36, 0.12) | 0.31 |  |
| Male | - | - | - | - |  |
| Female | 15 | 0.04 | (-0.14, 0.23) | 0.63 |  |
| **Subgroup = population** |  |  |  |  | 0.06 |
| Obese | 22 | -0.05 | (-0.19, 0.09) | 0.44 |  |
| Non-obese | 3 | 0.41 | (-0.05, 0.88) | 0.08 |  |
| **Subgroup = age** |  |  |  |  | 0.14 |
| ≤40 | 18 | 0.04 | (-0.10, 0.17) | 0.56 |  |
| >40 | 7 | -0.17 | (-0.43, 0.08) | 0.17 |  |
| **Subgroup = EX type** |  |  |  |  | 0.17 |
| Aerobic exercise (moderate) | 5 | -0.26 | (-0.56, 0.03) | 0.08 |  |
| Aerobic exercise (moderate to vigorous) | 2 | 0.08 | (-0.41, 0.57) | 0.75 |  |
| High-intensity interval training | 12 | -0.01 | (-0.17, 0.15) | 0.93 |  |
| Resistance exercise | 6 | 0.22 | (-0.10, 0.55) | 0.16 |  |
| Concurrent exercise | - | - | - | - |  |
| **Subgroup=Control group** |  |  |  |  | 0.57 |
| Intermittent fasting | 7 | -0.08 | (-0.31, 0.15) | 0.49 |  |
| Exercise | 11 | 0.07 | (-0.13, 0.27) | 0.49 |  |
| No EX and no IF | 7 | -0.05 | (-0.28, 0.19) | 0.68 |  |
| **Subgroup = IF type** |  |  |  |  | 0.34 |
| Alternate-day fasting | 3 | -0.26 | (-0.66, 0.14) | 0.20 |  |
| Time-restricted eating | 18 | 0.04 | (-0.12, 0.20) | 0.59 |  |
| Ramadan intermittent fasting | 2 | -0.27 | (-0.75, 0.21) | 0.25 |  |
| 5:2 diet | - | - | - | - |  |
| Intermittent energy restriction | 2 | 0.08 | (-0.43, 0.58) | 0.75 |  |
| **VO_2_max** |  |  |  |  |  |
| **Subgroup = sex** |  |  |  |  | 0.72 |
| Mixed | 3 | 0.39 | (-0.20, 0.97) | 0.18 |  |
| Male | 2 | 0.06 | (-0.54, 0.66) | 0.84 |  |
| Female | 21 | 0.25 | (0.12, 0.38) | <0.01 |  |
| **Subgroup = population** |  |  |  |  | 0.78 |
| Obese | 23 | 0.25 | (0.12, 0.38) | <0.01 |  |
| Non-obese | 3 | 0.19 | (-0.29, 0.66) | 0.42 |  |
| **Subgroup = age** |  |  |  |  | 0.58 |
| ≤40 | 19 | 0.23 | (0.09, 0.37) | <0.01 |  |
| >40 | 7 | 0.32 | (0.03, 0.60) | 0.03 |  |
| **Subgroup = EX type** |  |  |  |  | 0.82 |
| Aerobic exercise (moderate) | 1 | -0.03 | (-1.02, 0.96) | 0.95 |  |
| Aerobic exercise (moderate to vigorous) | - | - | - | - |  |
| High-intensity interval training | 18 | 0.25 | (0.10, 0.39) | <0.01 |  |
| Resistance exercise | - | - | - | - |  |
| Concurrent exercise | 7 | 0.28 | (-0.01, 0.57) | 0.06 |  |
| **Subgroup=Control group** |  |  |  |  | 0.33 |
| Intermittent fasting | 8 | 0.36 | (0.13, 0.58) | <0.01 |  |
| Exercise | 12 | 0.15 | (-0.05, 0.34) | 0.13 |  |
| No EX and no IF | 6 | 0.28 | (0.04, 0.52) | 0.02 |  |
| **Subgroup = IF type** |  |  |  |  | 0.87 |
| Alternate-day fasting | 3 | 0.39 | (-0.20, 0.97) | 0.19 |  |
| Time-restricted eating | 15 | 0.25 | (0.10, 0.40) | <0.01 |  |
| Ramadan intermittent fasting | - | - | - | - |  |
| 5:2 diet | 7 | 0.25 | (-0.03, 0.53) | 0.08 |  |
| Intermittent energy restriction | 1 | 0.001 | (-0.74, 0.75) | 0.99 |  |
| **Adiponection** |  |  |  |  |  |
| **Subgroup = sex** |  |  |  |  | 0.95 |
| Mixed | 4 | 0.28 | (-0.20, 0.77) | 0.22 |  |
| Male | 3 | 0.25 | (-0.29, 0.78) | 0.33 |  |
| Female | 6 | 0.32 | (0.06, 0.59) | 0.02 |  |
| **Subgroup = population** |  |  |  |  | 0.80 |
| Obese | 10 | 0.31 | (0.08, 0.55) | 0.01 |  |
| Non-obese | 3 | 0.25 | (-0.28, 0.77) | 0.33 |  |
| **Subgroup = age** |  |  |  |  | 0.93 |
| ≤40 | 6 | 0.31 | (-0.01, 0.64) | 0.06 |  |
| >40 | 7 | 0.30 | (0.02, 0.58) | 0.04 |  |
| **Subgroup = EX type** |  |  |  |  | 0.99 |
| Aerobic exercise (moderate) | 5 | 0.27 | (-0.16, 0.70) | 0.18 |  |
| Aerobic exercise (moderate to vigorous) | - | - | - | - |  |
| High-intensity interval training | 3 | 0.36 | (-0.07, 0.78) | 0.09 |  |
| Resistance exercise | 2 | 0.25 | (-0.45, 0.94) | 0.44 |  |
| Concurrent exercise | 3 | 0.30 | (-0.05, 0.66) | 0.08 |  |
| **Subgroup=Control group** |  |  |  |  | 0.84 |
| Intermittent fasting | 3 | 0.22 | (-0.21, 0.65) | 0.28 |  |
| Exercise | 7 | 0.31 | (-0.00, 0.62) | 0.05 |  |
| No EX and no IF | 3 | 0.38 | (-0.04, 0.80) | 0.07 |  |
| **Subgroup = IF type** |  |  |  |  | 0.99 |
| Alternate-day fasting | 4 | 0.28 | (-0.20, 0.77) | 0.22 |  |
| Time-restricted eating | 6 | 0.31 | (-0.02, 0.64) | 0.06 |  |
| Ramadan intermittent fasting | - | - | - | - |  |
| 5:2 diet | 3 | 0.30 | (-0.05, 0.65) | 0.08 |  |
| Intermittent energy restriction | - | - | - | - |  |
| **Leptin** |  |  |  |  |  |
| **Subgroup = sex** |  |  |  |  | 0.63 |
| Mixed | 6 | -0.34 | (-0.96, 0.28) | 0.26 |  |
| Male | 3 | -0.37 | (-1.05, 0.31) | 0.26 |  |
| Female | 8 | -0.03 | (-0.57, 0.51) | 0.90 |  |
| **Subgroup = population** |  |  |  |  | 0.58 |
| Obese | 14 | -0.17 | (-0.55, 0.21) | 0.36 |  |
| Non-obese | 3 | -0.37 | (-1.01, 0.28) | 0.24 |  |
| **Subgroup = age** |  |  |  |  | 0.61 |
| ≤40 | 9 | -0.29 | (-0.74, 0.16) | 0.19 |  |
| >40 | 8 | -0.13 | (-0.62, 0.35) | 0.57 |  |
| **Subgroup = EX type** |  |  |  |  | 0.70 |
| Aerobic exercise (moderate) | 8 | -0.13 | (-0.65, 0.38) | 0.59 |  |
| Aerobic exercise (moderate to vigorous) | - | - | - | - |  |
| High-intensity interval training | 3 | -0.53 | (-1.40, 0.34) | 0.21 |  |
| Resistance exercise | 6 | -0.20 | (-0.75, 0.36) | 0.47 |  |
| Concurrent exercise | - | - | - | - |  |
| **Subgroup=Control group** |  |  |  |  | 0.56 |
| Intermittent fasting | 2 | -0.39 | (-0.95, 0.17) | 0.15 |  |
| Exercise | 11 | -0.14 | (-0.50, 0.21) | 0.40 |  |
| No EX and no IF | 14 | -0.35 | (-0.84, 0.14) | 0.15 |  |
| **Subgroup = IF type** |  |  |  |  | 0.07 |
| Alternate-day fasting | 6 | -0.31 | (-0.71, 0.09) | 0.12 |  |
| Time-restricted eating | 6 | -0.44 | (-0.84, -0.04) | 0.03 |  |
| Ramadan intermittent fasting | 5 | 0.27 | (-0.22, 0.77) | 0.25 |  |
| 5:2 diet | - | - | - | - |  |
| Intermittent energy restriction | - | - | - | - |  |
| **C-reactive protein** |  |  |  |  |  |
| **Subgroup = sex** |  |  |  |  | 0.23 |
| Mixed | 10 | 0.10 | (-0.19, 0.38) | 0.47 |  |
| Male | - | - | - | - |  |
| Female | 2 | -0.32 | (-0.98, 0.35) | 0.31 |  |
| **Subgroup = population** |  |  |  |  | 0.11 |
| Obese | 9 | -0.07 | (-0.36, 0.22) | 0.59 |  |
| Non-obese | 3 | 0.42 | (-0.13, 0.98) | 0.12 |  |
| **Subgroup = age** |  |  |  |  | 0.75 |
| ≤40 | 5 | 0.07 | (-0.43, 0.56) | 0.76 |  |
| >40 | 7 | -0.03 | (-0.44, 0.39) | 0.89 |  |
| **Subgroup = EX type** |  |  |  |  | 0.54 |
| Aerobic exercise (moderate) | 3 | 0.00 | (-0.69, 0.69) | 1.00 |  |
| Aerobic exercise (moderate to vigorous) | 3 | 0.42 | (-0.37, 1.21) | 0.25 |  |
| High-intensity interval training | 1 | 0.01 | (-1.05, 1.07) | 0.98 |  |
| Resistance exercise | - | - | - | - |  |
| Concurrent exercise | 5 | -0.23 | (-0.81, 0.35) | 0.39 |  |
| **Subgroup=Control group** |  |  |  |  | 0.76 |
| Intermittent fasting | 3 | 0.09 | (-0.43, 0.61) | 0.70 |  |
| Exercise | 7 | 0.04 | (-0.35, 0.42) | 0.84 |  |
| No EX and no IF | 2 | -0.17 | (-0.865, 0.51) | 0.58 |  |
| **Subgroup = IF type** |  |  |  |  | 0.22 |
| Alternate-day fasting | 6 | 0.03 | (-0.32, 0.38) | 0.86 |  |
| Time-restricted eating | 1 | -0.34 | (-1.31, 0.64) | 0.45 |  |
| Ramadan intermittent fasting | 1 | -0.73 | (-1.75, 0.30) | 0.14 |  |
| 5:2 diet | 4 | 0.31 | (-0.18, 0.79) | 0.18 |  |
| Intermittent energy restriction | - | - | - | - |  |
| **Bench press** |  |  |  |  |  |
| **Subgroup = sex** |  |  |  |  | 0.28 |
| Mixed | 8 | 0.25 | (-0.09, 0.59) | 0.15 |  |
| Male | 14 | 0.05 | (-0.14, 0.24) | 0.60 |  |
| Female | 17 | -0.07 | (-0.27, 0.14) | 0.51 |  |
| **Subgroup = population** |  |  |  |  | 0.81 |
| Obese | 12 | 0.01 | (-0.24, 0.25) | 0.96 |  |
| Non-obese | 27 | 0.04 | (-0.11, 0.19) | 0.59 |  |
| **Subgroup = age** |  |  |  |  | 0.46 |
| ≤40 | 36 | 0.02 | (-0.11, 0.15) | 0.76 |  |
| >40 | 3 | 0.23 | (-0.33, 0.79) | 0.40 |  |
| **Subgroup = EX type** |  |  |  |  | 0.71 |
| Aerobic exercise (moderate) | - | - | - | - |  |
| Aerobic exercise (moderate to vigorous) | - | - | - | - |  |
| High-intensity interval training | 1 | -0.28 | (-1.06, 0.50) | 0.47 |  |
| Resistance exercise | 29 | 0.03 | (-0.11, 0.18) | 0.63 |  |
| Concurrent exercise | 9 | 0.06 | (-0.22, 0.33) | 0.68 |  |
| **Subgroup=Control group** |  |  |  |  | 0.79 |
| Intermittent fasting | 3 | 0.18 | (-0.27, 0.63) | 0.43 |  |
| Exercise | 33 | 0.02 | (-0.12, 0.16) | 0.80 |  |
| No EX and no IF | 3 | 0.03 | (-0.43, 0.48) | 0.90 |  |
| **Subgroup = IF type** |  |  |  |  | 0.52 |
| Alternate-day fasting | 3 | 0.23 | (-0.33, 0.80) | 0.40 |  |
| Time-restricted eating | 25 | 0.08 | (-0.09, 0.24) | 0.34 |  |
| Ramadan intermittent fasting | 10 | -0.07 | (-0.29, 0.16) | 0.56 |  |
| 5:2 diet | 1 | -0.28 | (-1.06, 0.50) | 0.47 |  |
| Intermittent energy restriction | - | - | - | - |  |
| **Leg press** |  |  |  |  |  |
| **Subgroup = sex** |  |  |  |  | 0.20 |
| Mixed | 6 | 0.05 | (-0.75, 0.85) | 0.90 |  |
| Male | 9 | -0.23 | (-0.87, 0.41) | 0.47 |  |
| Female | 13 | 0.58 | (-0.07, 1.22) | 0.08 |  |
| **Subgroup = population** |  |  |  |  | 0.11 |
| Obese | 10 | 0.58 | (-0.09, 1.24) | 0.09 |  |
| Non-obese | 18 | -0.09 | (-0.56, 0.39) | 0.72 |  |
| **Subgroup = age** |  |  |  |  | 0.98 |
| ≤40 | 27 | 0.14 | (-0.32, 0.60) | 0.54 |  |
| >40 | 1 | 0.11 | (-1.54, 1.76) | 0.89 |  |
| **Subgroup = EX type** |  |  |  |  | 0.08 |
| Aerobic exercise (moderate) | - | - | - | - |  |
| Aerobic exercise (moderate to vigorous) | - | - | - | - |  |
| High-intensity interval training | 1 | -0.20 | (-1.51, 1.11) | 0.76 |  |
| Resistance exercise | 20 | -0.08 | (-0.52, 0.36) | 0.71 |  |
| Concurrent exercise | 7 | 0.85 | (0.13, 1.57) | 0.02 |  |
| **Subgroup=Control group** |  |  |  |  | **<0.01** |
| Intermittent fasting | 3 | 1.41 | (0.68, 2.15) | <0.01 |  |
| Exercise | 23 | -0.08 | (-0.36, 0.21) | 0.59 |  |
| No EX and no IF | 2 | 1.31 | (0.46, 2.16) | <0.01 |  |
| **Subgroup = IF type** |  |  |  |  | 0.65 |
| Alternate-day fasting | 1 | 0.11 | (-1.55, 1.78) | 0.89 |  |
| Time-restricted eating | 17 | 0.38 | (-0.22, 0.98) | 0.21 |  |
| Ramadan intermittent fasting | 9 | -0.22 | (-1.05, 0.61) | 0.59 |  |
| 5:2 diet | 1 | -0.20 | (-1.78, 1.38) | 0.80 |  |
| Intermittent energy restriction | - | - | - | - |  |

Notes: *p* value, statistically significant p values for pooled results; *I*^2^, quantitative indicators of heterogeneity; Hedge's *g*, the effect size indicators used in the pooled;

Abbreviations: 95%CI = 95% confidence interval; K = the total number of effects included in the pooled effect size; EX = exercise; IF = intermittent fasting; EX+IF = exercise combined with intermittent fasting.

**Supplementary Table 6 (Characteristics of Athletes, Active, and Trained Studies, IF Type, and Sport)**

| **Author (year)** | **Study characteristics** | | **Participant characteristics** | | | **Intermittent fasting intervention characteristics** | **Exercise intervention characteristics** | | | | |
| --- | --- | --- | --- | --- | --- | --- | --- | --- | --- | --- | --- |
|  | **Intervention Groups** | **Sample size(female/male)** | **Age (Years)**  **Mean ± SD** | **BMI**  **Mean ± SD** | **Participant** |  | **Type** | **Protocol** | **Fre** | **Sess** | **Week** |
| **Athletes (n = 7)** | | | | | | | | | | | |
| Tayebi et al.2010 | RIF+RT  RT  RIF  No EX and no IF | RIF+RT:10(0/10)  RT:10(0/10)  RIF:10(0/10)  No EX and no IF:10(0/10) | 21.3± 1.6 | NR | Athletes (Weight lifter) | RIF: during the Ramadan phase, participants fasted from dawn until sunset, abstaining from both food and drink | RT | RT: Weightlifting technique training and hypertrophy body building.  Intensity: 65%-75% 1-RM | 3 | 90 | 30 days |
| Trabelsi et al.2013 | RIF+RE  RT | RIF+RE:8(0/8)  RT:8(0/8) | RIF+RE:25 ± 3  RT:25 ± 2 | RIF+RE:25.8 ± 0.4  RT:26.0 ± 1.7 | Athletes (Bodybuilder) | RIF: fasted for roughly 15 h each day | RT | RT: Each training session consisted of four to six specific exercises. Each exercise was performed in four sets with a load of 10 RM and 2-3 min intervals between sets. | 4 | 60 | 4 |
| Aloui et al.2018 | RIF+RT  RIF+RT  RIF | RIF+RT:10(0/10)  RIF:10(0/10) | 22.9±1.3 | 22.5 | Athletes (Amateur male football player) | RIF: participants fasted from 3:00 am (last meal before dawn) until sunset (about 19:20) and continued throughout Ramadan (about 16 hours). | RT | Each training session consisted of three sets × six 40-m round-trip sprints (20 m +180° turning) with a 4-min rest between sets and a 20-s rest between sprints | 4 | 30 | 4 |
| Moro et al.2020 | TRE+AE(M)  AE(M) | TRE+AE(M):8(0/8)  AE(M):8(0/8) | TRE+AE(M):19.38 ± 2.39  AE(M):19.38 ± 1.60 | TRE+AE(M):21.85 ± 1.65  AE(M):22.47 ± 1.83 | Athletes (Elite cyclists) | TRE: the TRE group consumed 100% of its estimated daily energy needs in an 8-h time window (from 10:00 a.m. to 6:00 p.m.) | AE(M) | AE(M): 500 ± 50 km cycling per week, mainly steady state training of moderate intensity. | 6 | 143 | 4 |
| Brady et al.2021 | TRE+AE(M)  AE(M) | TRE+AE(M):10(0/10)  AE(M):7(0/7) | TRE+AE(M):35.9 ± 8.6  AE(M):39.9 ± 3.0 | TRE+AE(M):22.52 ± 2.44  AE(M):22.32 ± 2.37 | Athletes (Male middle- and long-distance runners) | TRE: Participants in the TRE group were instructed to consume all of their dietary intake within an 8-h window, typically between 1200 and 2000 h. Only water was permitted outside the window. | AE(M) | Average weekly training load (Session-RPE method): 1718±476 AU. Training ≥5 days per week | 5 | 40 | 8 |
| Brini et al.2021 | RIF+HIIT  HIIT | RIF+HIIT:12(0/12)  HIIT:12(0/12) | RIF+HIIT:25.32 ± 2.56  HIIT:24.85 ± 1.55 | RIF+HIIT:23.43 ± 1.19  HIIT:23.41 ± 1.46 | Athletes (Professional basketball players) | RIF: The daily fasting period was about 16-17 hours. They ate only at night and ate less frequently than usual. Daytime training sessions were held between 5:00 and 6:30 p.m | HIIT | A 3-on-3 game was played in a half-court (14×15 m) with a total duration of 12 min per game (divided into three 4-min sessions with a 2-min rest between sessions). Only man - to - man strategies are allowed. | 5 | 12 | 4 |
| Richardson et al.2023 | TRE+AE(M)  AE(M) | TRE+AE(M):15(0/15)  AE(M):15(0/15) | 28.7±5.2 | 23.3 | Athletes (Long-distance runner) | TRE: daily fasting was 16 h with an 8-h feeding window | AE(M) | The weekly running volume was 52.9±10.8 km | 4 | 80 | 4 |
| **Active(4) + Trained(6) = 10** | | | | | | | | | | | |
| Trabelsi et al.2012 | RIF+AE(M)  AE(M) | RIF+AE(M):10(0/10)  AE(M):9(0/9) | RIF+AE(M):26.6 ± 3.0  AE(M):27.6 ± 1.8 | RIF+AE(M):24.6 ± 1.4  AE(M):24.5 ± 1.6 | Active | RIF: fasted for roughly 15 h each day | AE(M) | AE(M): Bicycle, running and rowing machine once each.  Intensity: 60-80% heart rate max | 3 | 40-60 | 30 days |
| Tinsley et al.2019 | TRF+RT  RT | TRE+RT:21(21/0)  RT:17(17/0) | TRE+RT:22.3 ± 3.4  RT:22.0 ± 2.4 | NR | Active | TRE: on non-training days (four days per week), participants were instructed to consume all their calories within a flexible four-hour window between 4:00 p.m. and midnight. | RT | Alternating upper and lower body workouts, completing four sets of 8–12 repetitions per exercise, with 90 s of rest between sets, three times per week. | 3 | 45-60 | 8 |
| Martínez-Rodríguez et al.2020 | TRE+HIIT  HIIT | TRE+HIIT:7(7/0)  HIIT:7(7/0) | 27 ± 6 | 21.1 ± 1.8 | Active | TRE: time-restricted feeding (fasting window >14 hours, breakfast consumed immediately after waking, followed by meal distribution within remaining hours) on alternate days (3 fasting days/week in odd weeks, 4 days/week in even weeks) | HIIT | HIIT: three sets ×10 reps ×30 seconds of all-out aerobic exercise (cycling, rowing, running) with 30 seconds of passive recovery interval. | 3 | 40 | 8 |
| Stratton et al.2020 | TRE+RT  RT | TRE+RT:13(0/13)  RT: 13(0/13) | TRE+RT:22.9 ± 3.6  RT: 22.5 ± 2.2 | TRE+RT:25.8  RT:26.4 | Active | TRE: daily 16-hour fasting with an 8-hour feeding window (12 noon to 8 PM or 1 PM to 9 PM). | RT | RT: Squats, bench press, rowing, shoulder training, etc | 3 | 60 | 4 |
| Moro et al.2016 | TRF+RT  RT | TRE+RT:17(0/17)  RT:17(0/17) | TRE+RT:29.94 ± 4.07  RT:28.47 ± 3.48 | NR | Trained | TRE: fasting for the remaining 16 h each day. | RT | Engaged in a split resistance training routine, completing three weekly sessions. Each session comprised three sets of 6–8 reps at 85–90% of 1-RM, performed to failure with 3 min rest intervals between sets and exercises. | 3 | 40-60 | 8 |
| Tinsley et al.2017 | TRE+RT  RT | TRE+RT:10(0/10)  RT:8(0/8) | TRE+RT:22.9 ± 4.1  RT:22.0± 2.4 | NR | Trained | TRE: instructed to consume all their calories within a flexible four-hour window between 4:00 p.m. and midnight. | RT | Engaged in a split resistance training routine, completing three weekly sessions. Each session comprised three sets of 6–8 reps at 85–90% of 1-RM, performed to failure with 3 min rest intervals between sets and exercises. | 3 | 45-60 | 8 |
| Tovar et al.2021 | TRE+AE(MV)  AE(MV) | TRE+AE(MV):15(0/15)  AE(MV):15(0/15) | 28.7 ± 5.2 | 23.3 | Trained | TRE: the 16/8 protocol required participants to consume all meals within a consistent 8 h daily window. During the fasting period, only water and non-caloric beverages such as unsweetened black coffee or plain tea were permitted. | AE(MV) | One day with high-intensity exercise, one day with moderate-intensity exercise | 3 | 23 | 4 |
| Correia et al.2023 | TRE+HIIT  HIIT | TRE+HIIT:18(0/18)  HIIT:18(0/18) | TRE+HIIT:23.7 ± 2.6  HIIT:23.7 ± 2.6 | 23.9 | Trained | TRE: participants ate two to three ad libitum meals within an 8 h window (1–9 p.m.), consuming only water, tea, and black coffee outside this period. | HIIT | Participants followed a structured training program during each dietary intervention, performing four sets of 8–10 repetitions at 85% of their 1-RM on leg press, bench press, leg extension, leg curl, shoulder press, and lat pulldown | 3 | 60 | 4 |
| Siedler et al.2023 | ADF+RT  RT | ADF+RT:18(18/0)  RT:20(20/0) | ADF+RT:23.3±4.4  RT:21.3±3.8 | ADF+RT:24.3  RT:22.5 | Trained | ADF: an intermittent energy-restricted diet (INT) that included a one-week period of energy balance after the second and fourth weeks of energy restriction. | RT | An alternating upper body/lower body split. | 4 | 30 | 8 |
| Correia et al.2024 | TRE+AE+HIIT  AE+HIIT | TRE+AE+HIIT:15(0/15)  AE+HIIT:15(0/15) | TRE+AE+HIIT:23.7 ± 2.6  AE+HIIT:23.7 ± 2.6 | NR | Trained | TRE: the TRE intervention used a 16/8 protocol, where participants consumed 2 to 3 meals within an 8 h window (1:00–9:00 p.m.) and were allowed only water, tea, and black coffee outside this period. | AE+HIIT | During the first 2 weeks of the intervention, participants completed 10 km runs per session with 24 h of rest between workouts. In the final 2 weeks, they continued the 10-km runs and added 1 km running intervals after each continuous run, separated by 4 min of active recovery | 3 | 60 | 4 |

**Supplementary Table 7 (Subgroup analysis: athletes, active/trained, and normal adults)**

| **No. of studies** | **K** | **Hedges' g** | **95%CI** | ***p*** | ***p*_subgroup_** |
| --- | --- | --- | --- | --- | --- |
| **Body mass** | | | | | |
| **Subgroup= population** |  |  |  |  | 0.80 |
| Athletes | 8 | -0.14 | (-0.45, 0.18) | 0.39 |  |
| Active/Trained | 23 | -0.08 | (-0.24, 0.09) | 0.37 |  |
| Normal Adults | 121 | -0.14 | (-0.18, -0.09) | <0.01 |  |
| **Body fat (%)** |  |  |  |  |  |
| **Subgroup= population** |  |  |  |  | 0.87 |
| Athletes | 3 | -0.27 | (-0.75, 0.21) | 0.27 |  |
| Active/Trained | 21 | -0.14 | (-0.32, 0.04) | 0.13 |  |
| Normal Adults | 50 | -0.18 | (-0.28, -0.08) | <0.01 |  |
| **Fat mass** |  |  |  |  |  |
| **Subgroup= population** |  |  |  |  | 0.80 |
| Athletes | 3 | -0.28 | (-0.79, 0.22) | 0.27 |  |
| Active/Trained | 21 | -0.12 | (-0.30, 0.05) | 0.16 |  |
| Normal Adults | 66 | -0.17 | (-0.24, -0.09) | <0.01 |  |
| **Fat free mass** |  |  |  |  |  |
| **Subgroup= population** |  |  |  |  | 0.98 |
| Athletes | 2 | -0.07 | (-0.66, 0.53) | 0.82 |  |
| Active/Trained | 15 | -0.01 | (-0.22, 0.20) | 0.93 |  |
| Normal Adults | 26 | -0.03 | (-0.18, 0.12) | 0.69 |  |
| **Lean body mass** |  |  |  |  |  |
| **Subgroup= population** |  |  |  |  | 0.96 |
| Athletes | 1 | -0.16 | (-1.17, 0.86) | 0.76 |  |
| Active/Trained | 7 | -0.04 | (-0.35, 0.27) | 0.79 |  |
| Normal Adults | 35 | -0.08 | (-0.18, 0.02) | 0.13 |  |
| **Total cholesterol** |  |  |  |  |  |
| **Subgroup= population** |  |  |  |  | 0.83 |
| Athletes | 3 | -0.21 | (-0.71,0.30) | 0.42 |  |
| Active/Trained | 7 | -0.07 | (-0.37, 0.22) | 0.62 |  |
| Normal Adults | 53 | -0.05 | (-0.15, 0.04) | 0.25 |  |
| **Triglyceride** |  |  |  |  |  |
| **Subgroup= population** |  |  |  |  | 0.13 |
| Athletes | 4 | -0.55 | (-1.01, -0.08) | 0.02 |  |
| Active/Trained | 8 | -0.03 | (-0.34, 0.27) | 0.84 |  |
| Normal Adults | 47 | -0.07 | (-0.18, 0.04) | 0.21 |  |
| **High density lipoprotein** |  |  |  |  |  |
| **Subgroup= population** |  |  |  |  | 0.13 |
| Athletes | 3 | -0.49 | (-0.99, 0.00) | 0.05 |  |
| Active/Trained | 8 | 0.08 | (-0.22, 0.37) | 0.61 |  |
| Normal Adults | 44 | 0.00 | (-0.10, 0.10) | 0.96 |  |
| **Low Density Lipoprotein** |  |  |  |  |  |
| **Subgroup= population** |  |  |  |  | 0.20 |
| Athletes | 3 | 0.31 | (-0.24, 0.85) | 0.27 |  |
| Active/Trained | 7 | 0.03 | (-0.33, 0.39) | 0.86 |  |
| Normal Adults | 49 | -0.16 | (-0.30, -0.02) | 0.02 |  |
| **Fasting glucose** |  |  |  |  |  |
| **Subgroup= population** |  |  |  |  | 0.16 |
| Athletes | 5 | -0.43 | (-0.86, -0.00) | 0.05 |  |
| Active/Trained | 8 | 0.06 | (-0.22, 0.35) | 0.66 |  |
| Normal Adults | 42 | -0.11 | (-0.20,-0.01) | 0.03 |  |
| **Insulin** |  |  |  |  |  |
| **Subgroup= population** |  |  |  |  | 0.23 |
| Athletes | 3 | 0.20 | (-0.37, 0.77) | 0.49 |  |
| Active/Trained | 5 | -0.13 | (-0.55, 0.28) | 0.53 |  |
| Normal Adults | 27 | -0.28 | (-0.49, -0.07) | 0.01 |  |
| **HOMA-IR** |  |  |  |  |  |
| **Subgroup= population** |  |  |  |  | 0.11 |
| Athletes | 2 | 0.24 | (-0.37, 0.85) | 0.42 |  |
| Normal Adults | 28 | -0.27 | (-0.40,-0.13) | <0.01 |  |
| **SBP** |  |  |  |  |  |
| **Subgroup= population** |  |  |  |  | 0.17 |
| Athletes | 1 | -0.26 | (-0.99, 0.49) | 0.49 |  |
| Active/Trained | 4 | -0.40 | (-0.80,-0.00) | 0.05 |  |
| Normal Adults | 37 | -0.04 | (-0.12, 0.05) | 0.39 |  |
| **DBP** |  |  |  |  |  |
| **Subgroup= population** |  |  |  |  | 0.22 |
| Athletes | 1 | -0.61 | (-1.37, 0.15) |  |  |
| Active/Trained | 4 | -0.24 | (-0.64, 0.16) |  |  |
| Normal Adults | 36 | -0.04 | (-0.13, 0.05) |  |  |
| **VO_2_max** |  |  |  |  |  |
| **Subgroup= population** |  |  |  |  | 0.47 |
| Athletes | 2 | 0.06 | (-0.54, 0.67) | 0.83 |  |
| Normal Adults | 27 | 0.28 | (0.16, 0.40) | <0.01 |  |
| **Bench press** |  |  |  |  |  |
| **Subgroup= population** |  |  |  |  | 0.36 |
| Active/Trained | 15 | -0.05 | (-0.27, 0.18) | 0.68 |  |
| Normal Adults | 26 | 0.08 | (-0.08, 0.24) | 0.32 |  |
| **Leg press** |  |  |  |  |  |
| **Subgroup= population** |  |  |  |  | 0.93 |
| Active/Trained | 1 | -0.06 | (-0.82, 0.70) | 0.88 |  |
| Normal Adults | 28 | -0.03 | (-0.23, 0.18) | 0.80 |  |


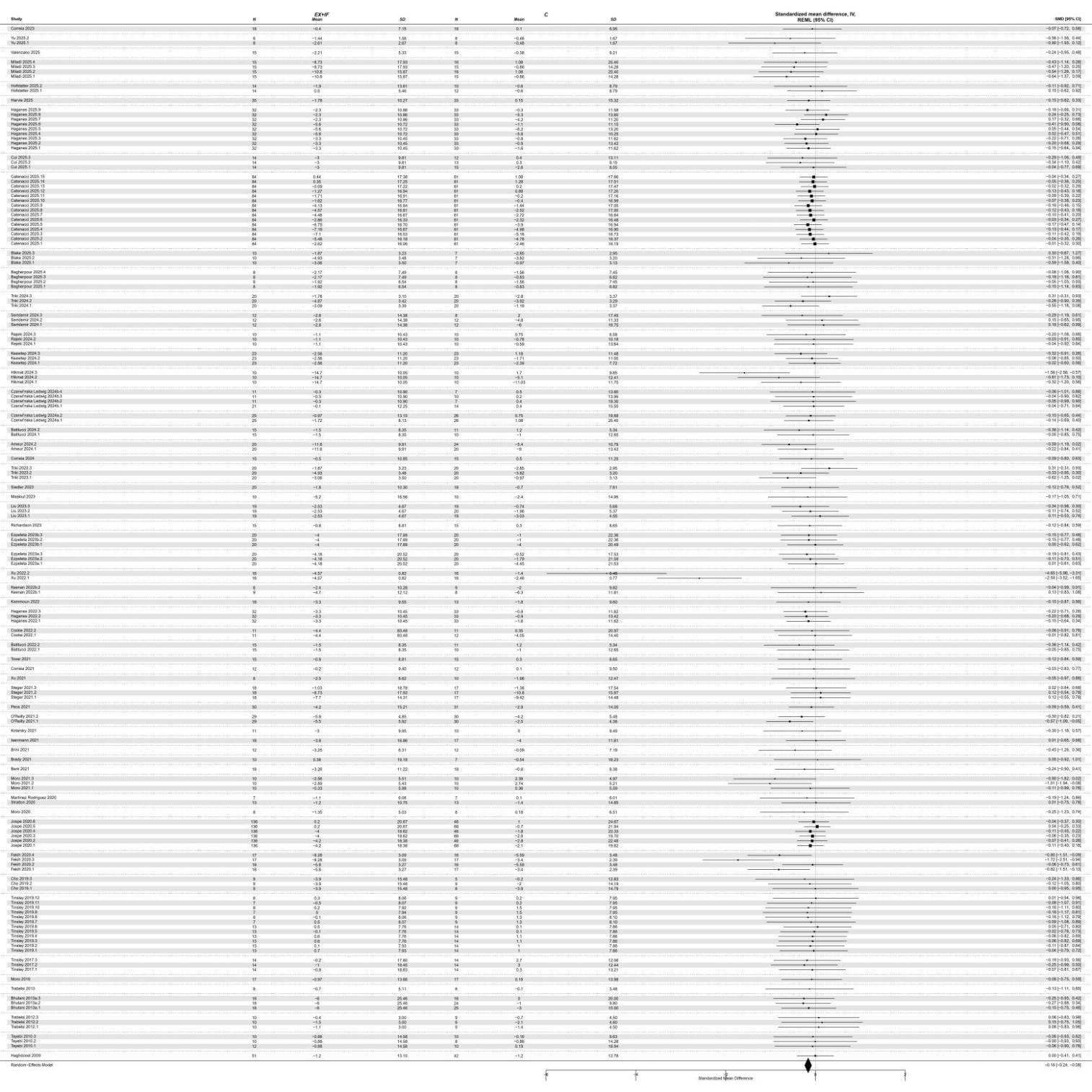


**Supplementary Fig.1A (Forest plots of body mass)**


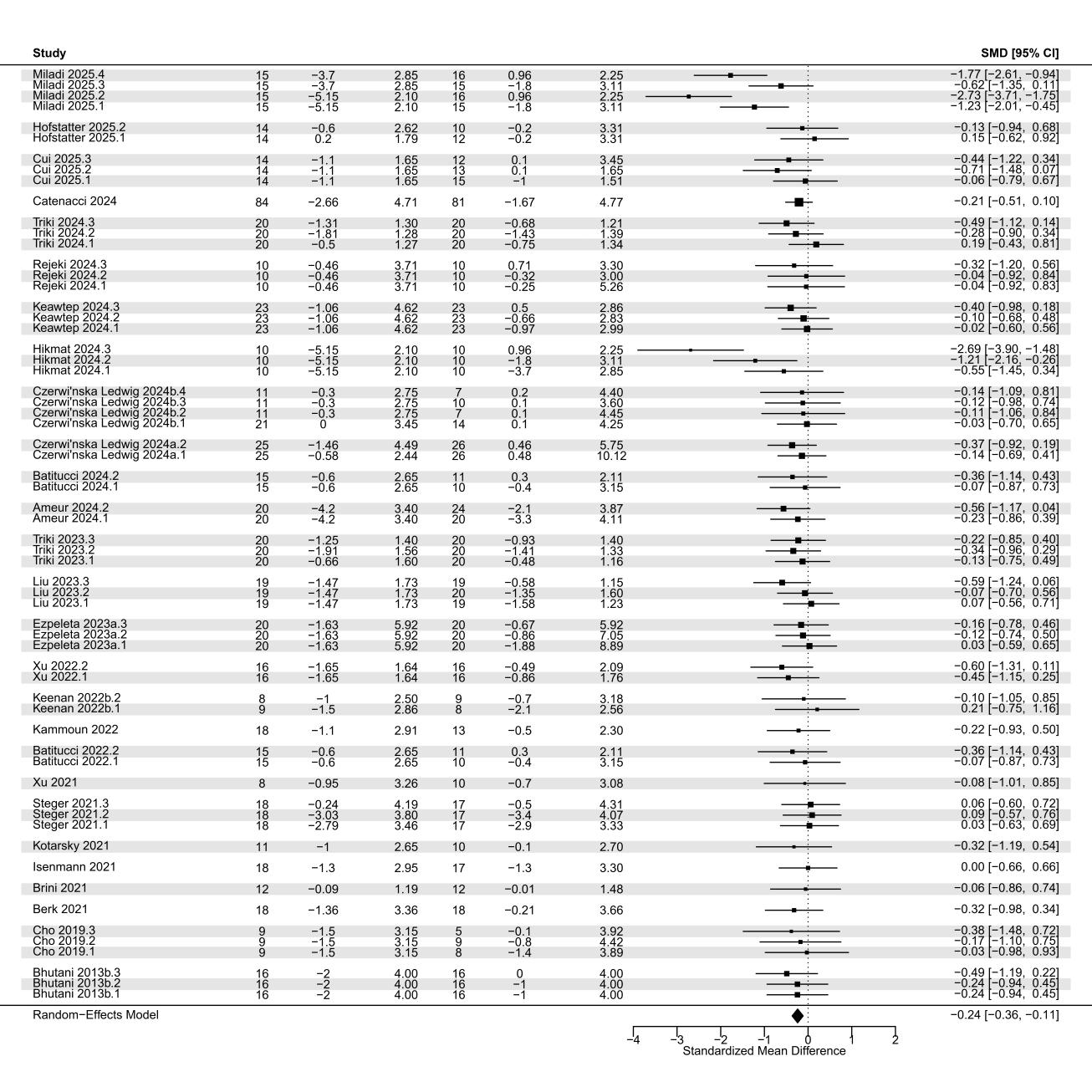


**Supplementary Fig.1B (Forest plots of BMI)**


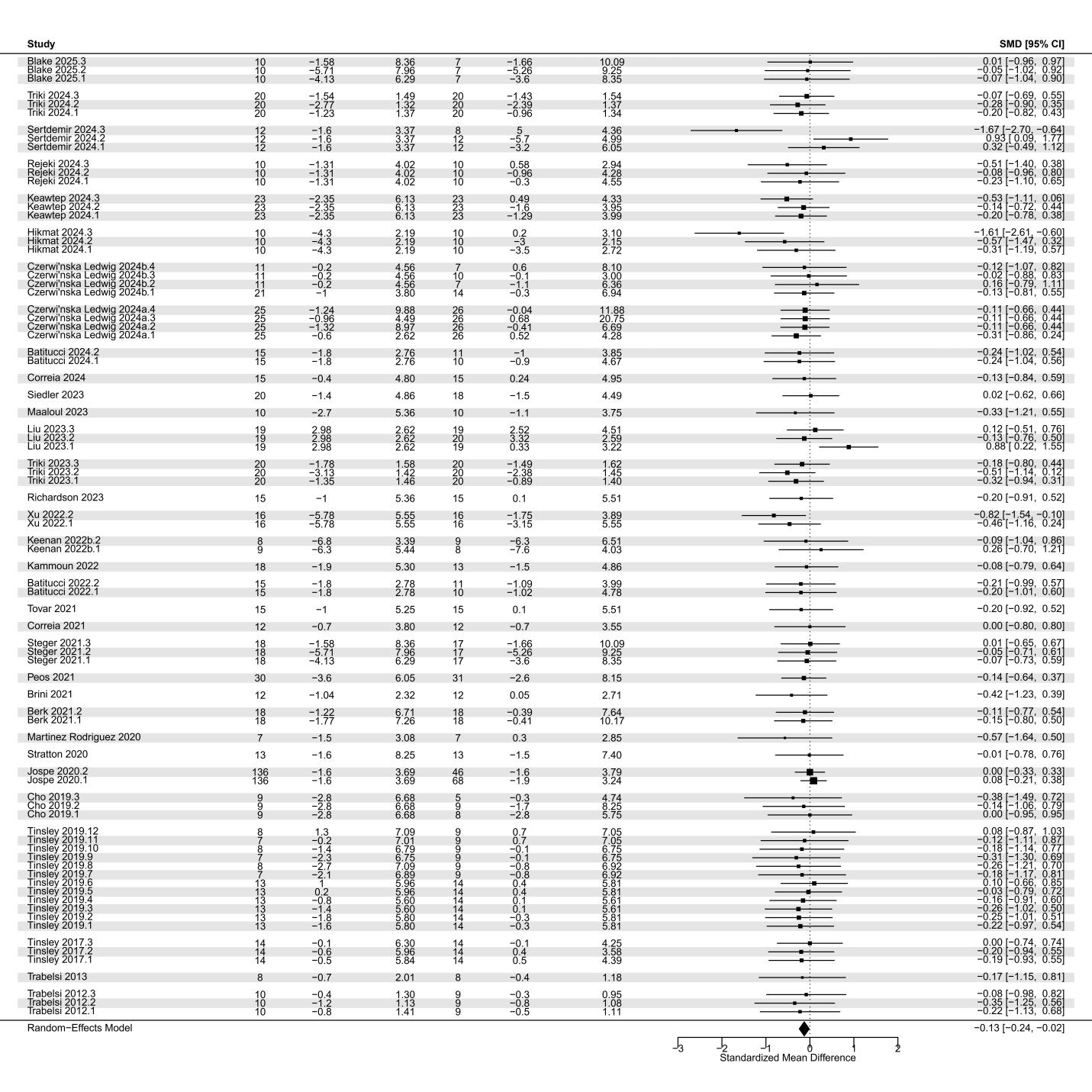


**Supplementary Fig.1C (Forest plots of body fat%)**


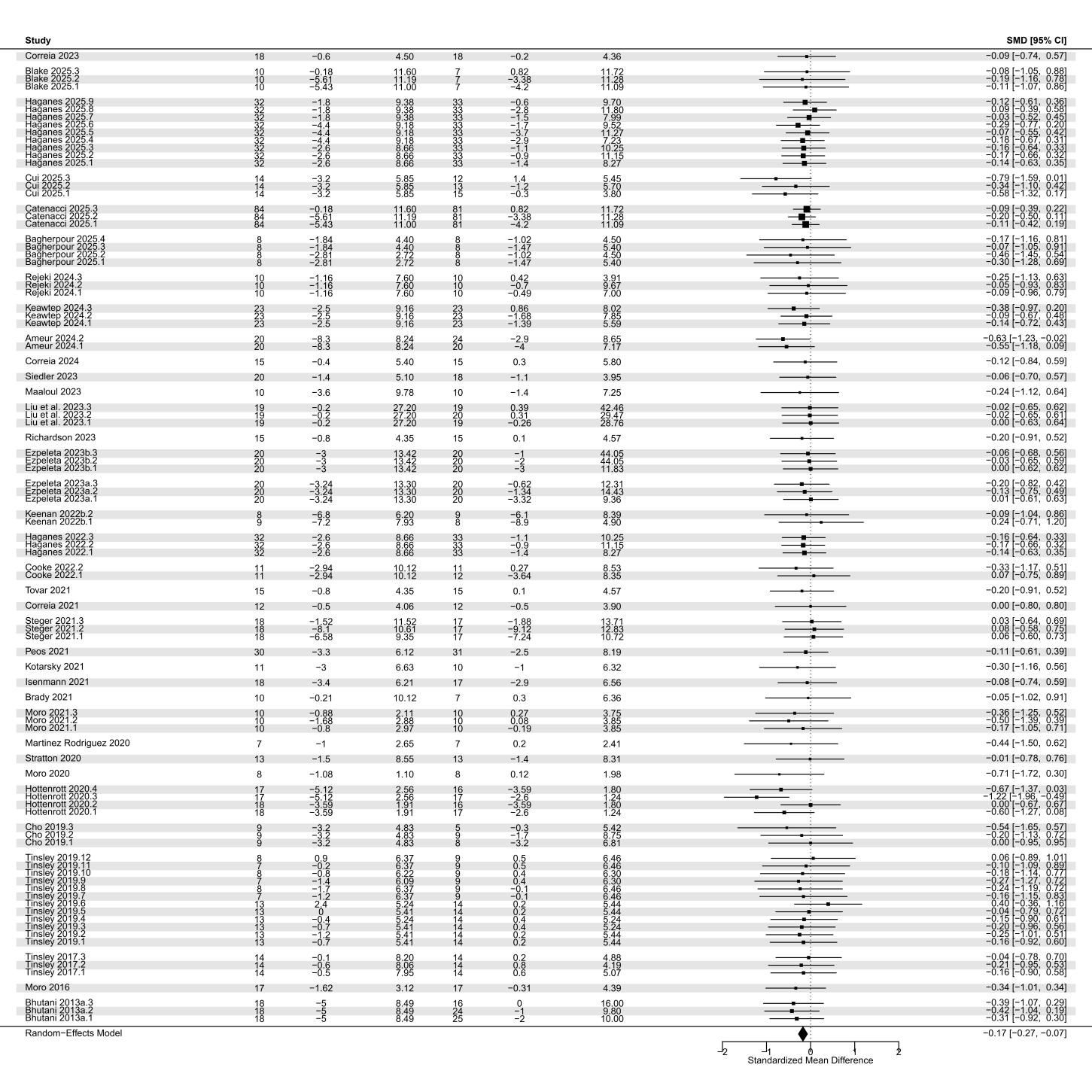


**Supplementary Fig.1D (Forest plots of fat mass)**


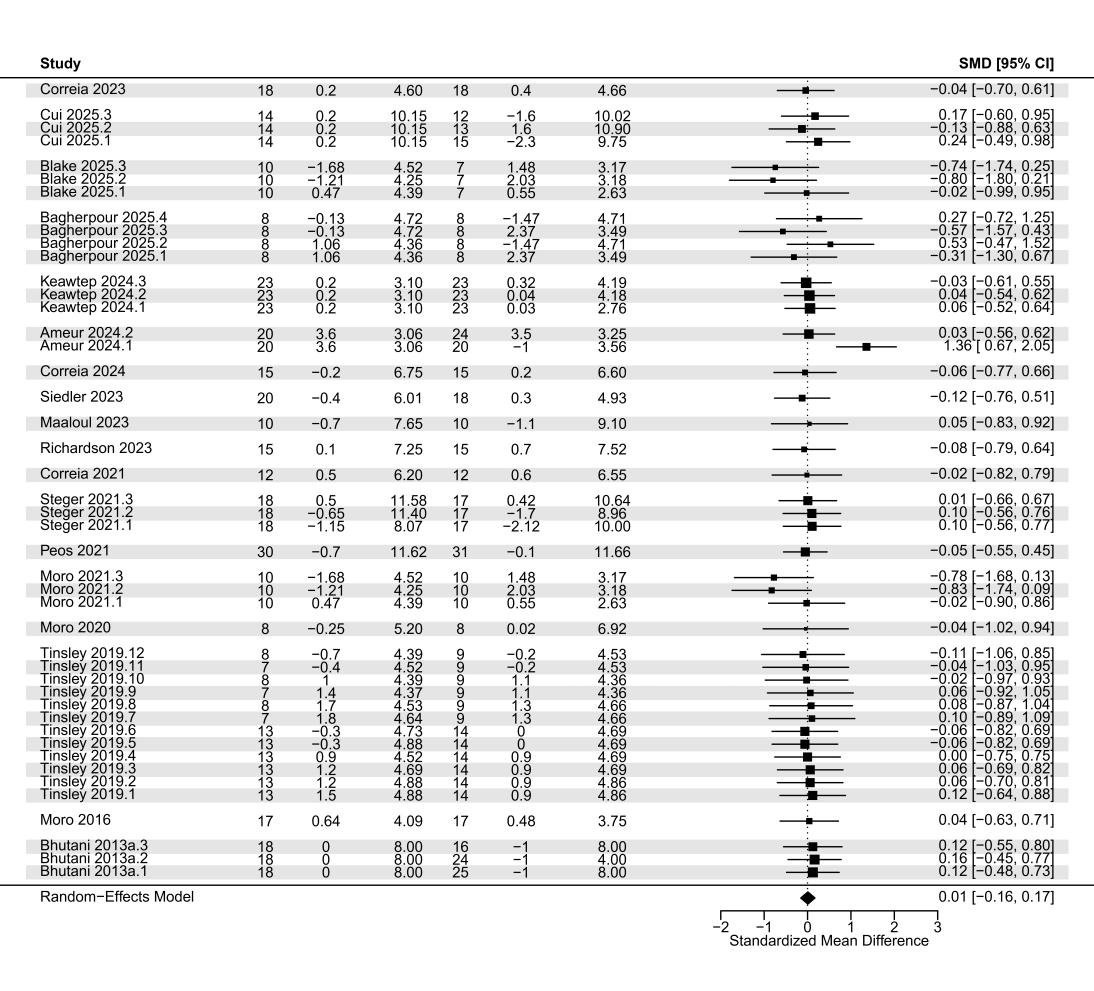


**Supplementary Fig.1E (Forest plots of fat free mass)**


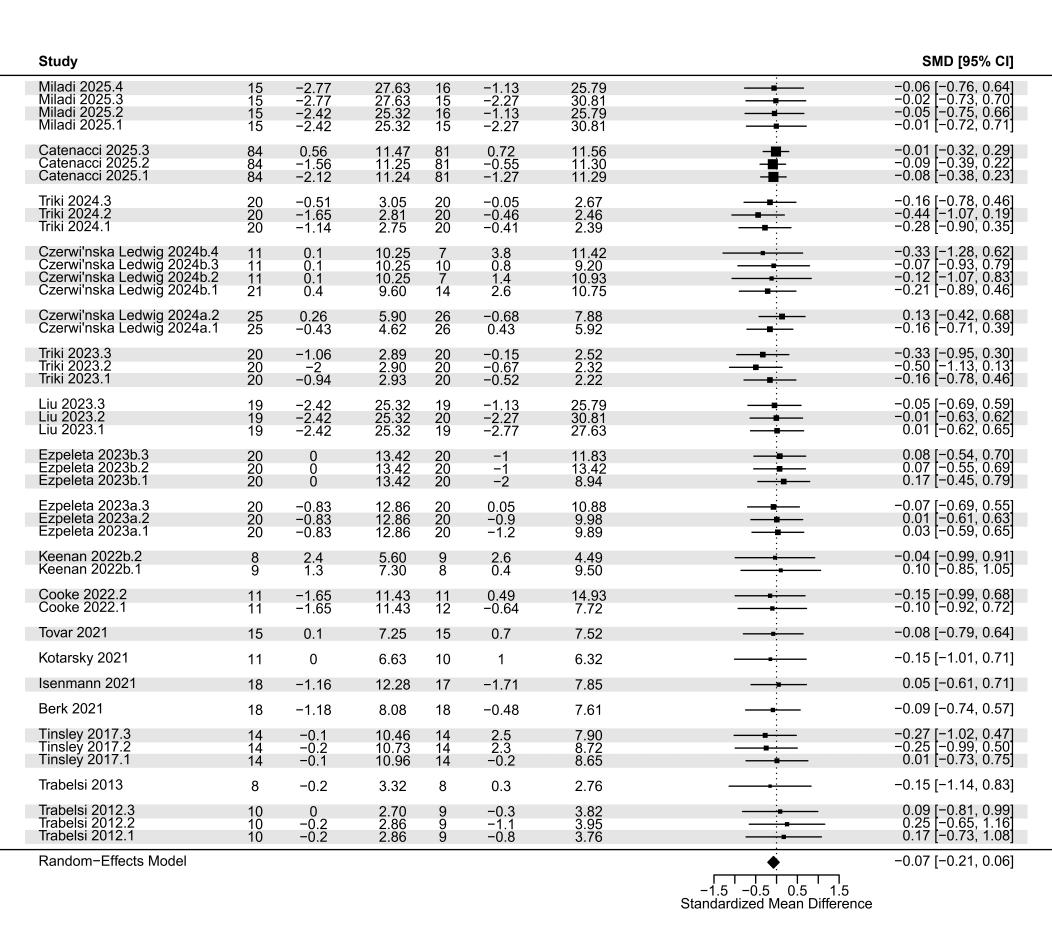


**Supplementary Fig.1F (Forest plots of lean body mass)**


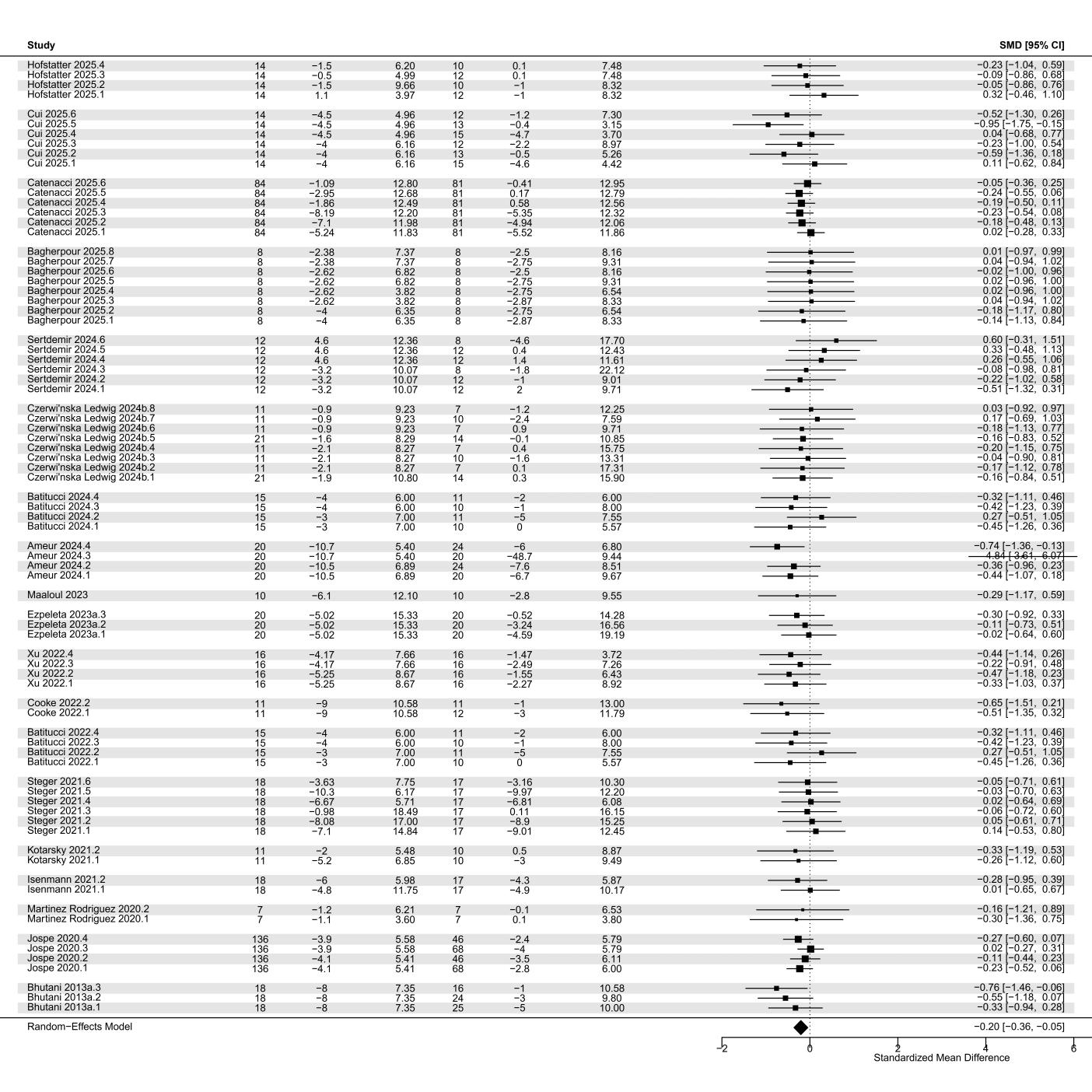


**Supplementary Fig.1G (Forest plots of waist circumference)**


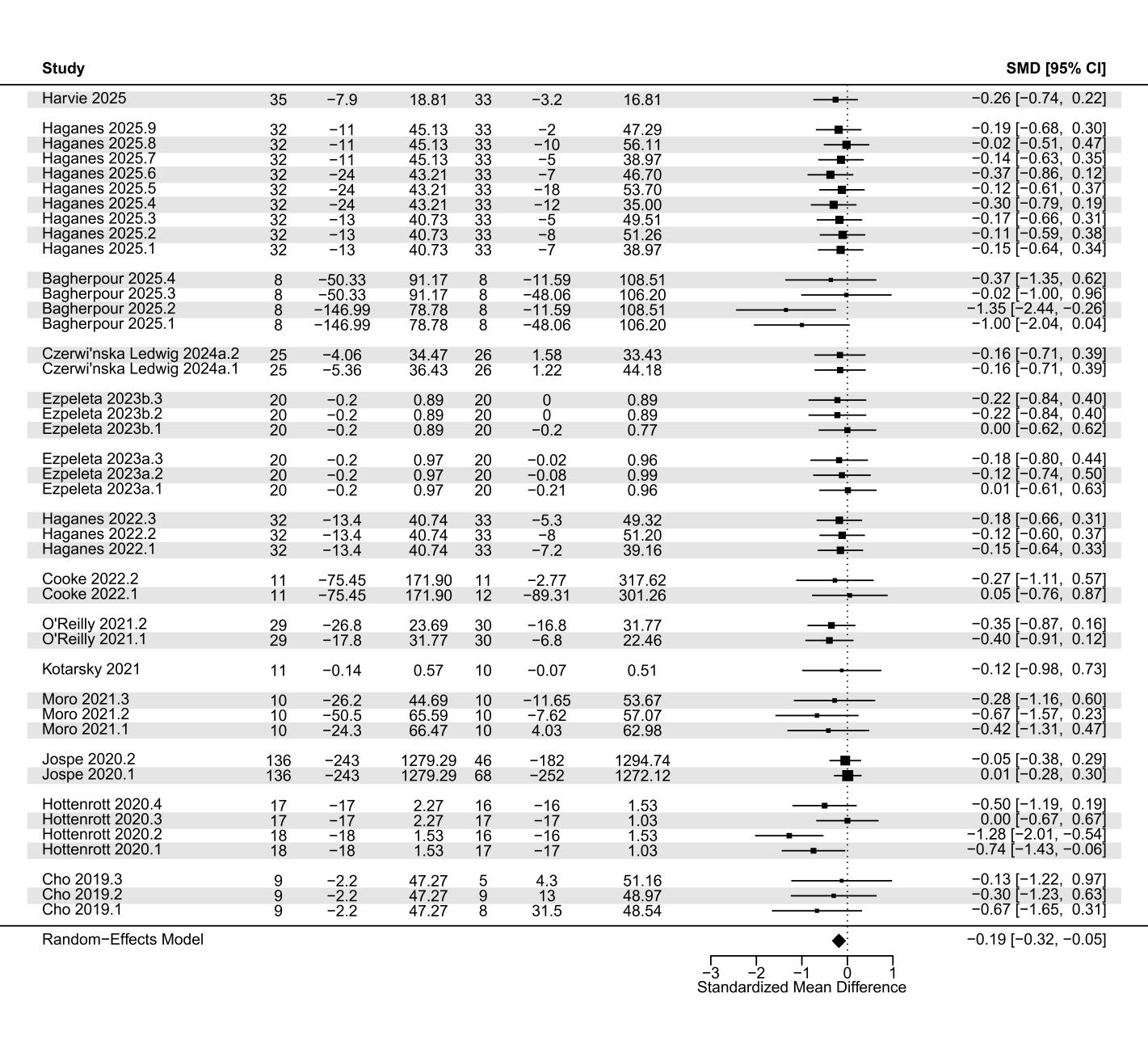


**Supplementary Fig.1H (Forest plots visceral adipose tissue)**


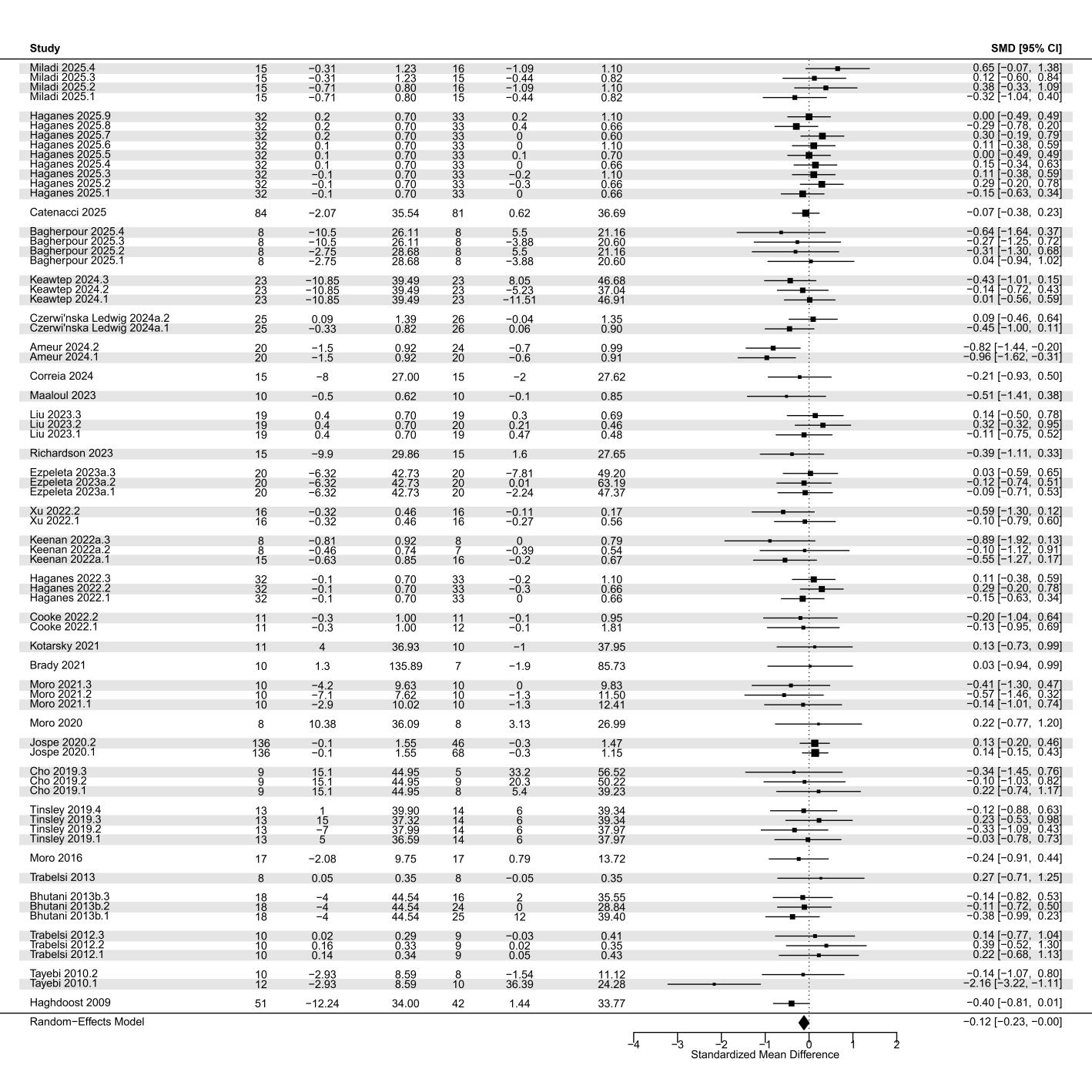


**Supplementary Fig.1I (Forest plots of total cholesterol)**


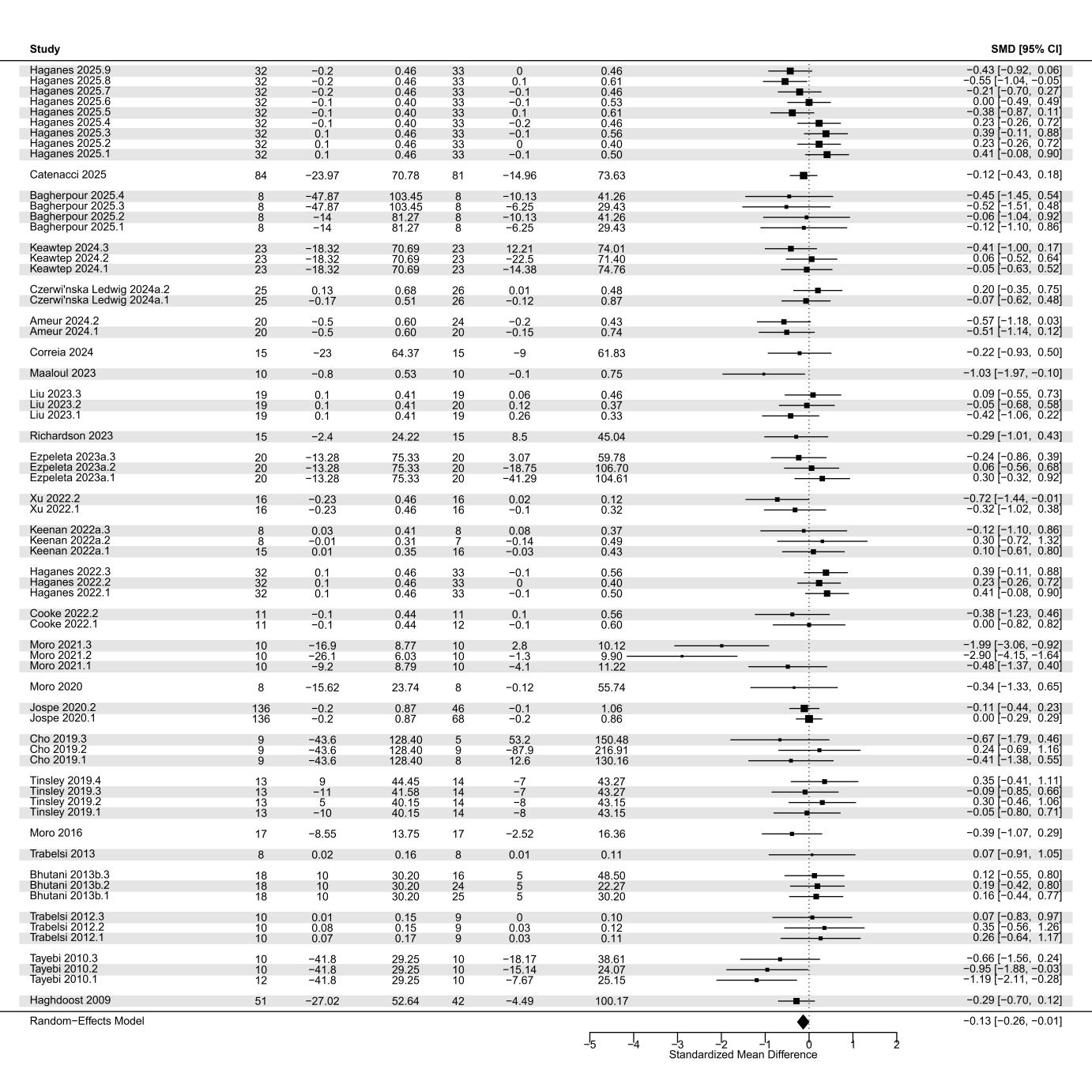


**Supplementary Fig.1J (Forest plots of triglyceride)**


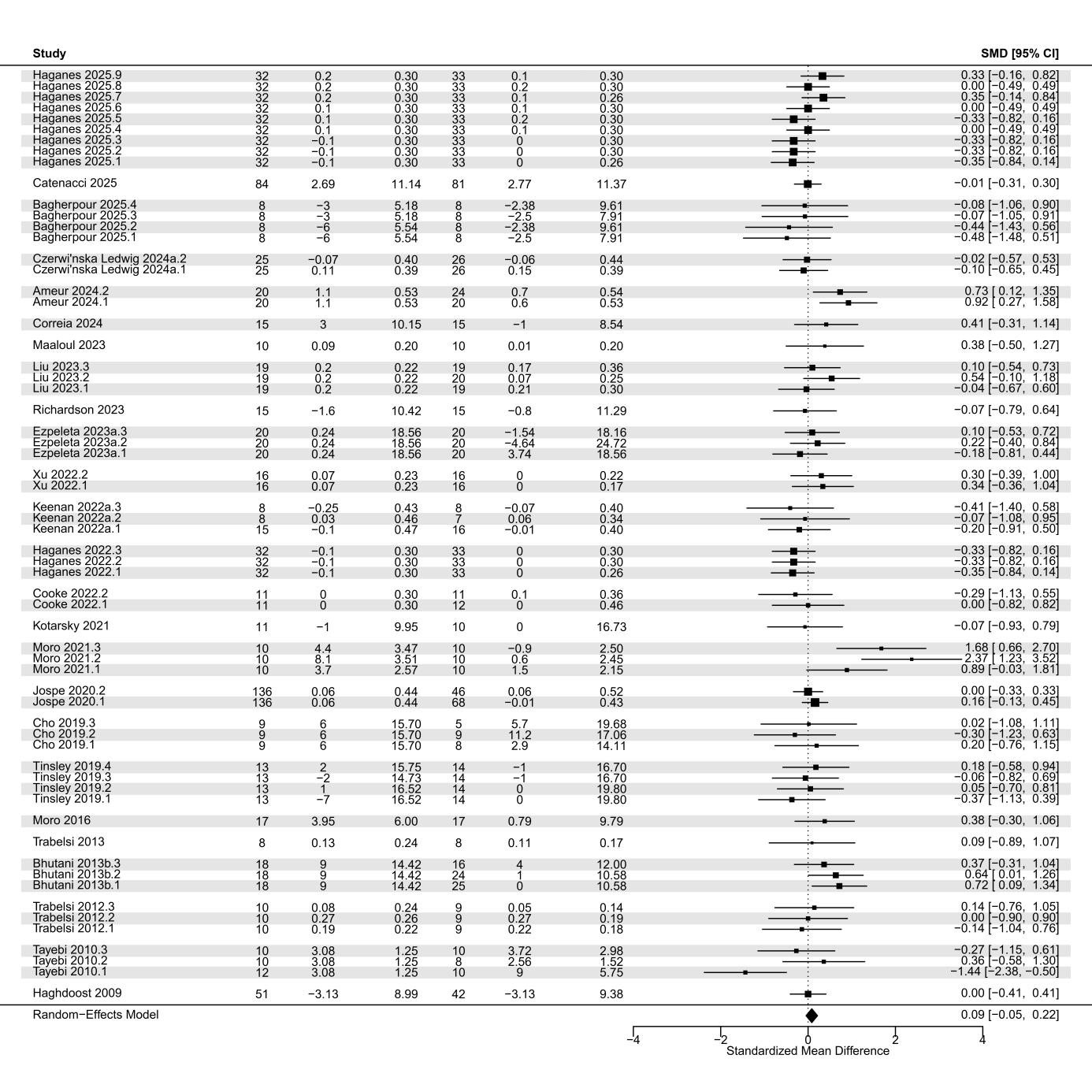


**Supplementary Fig.1K (Forest plots of high density lipoprotein)**


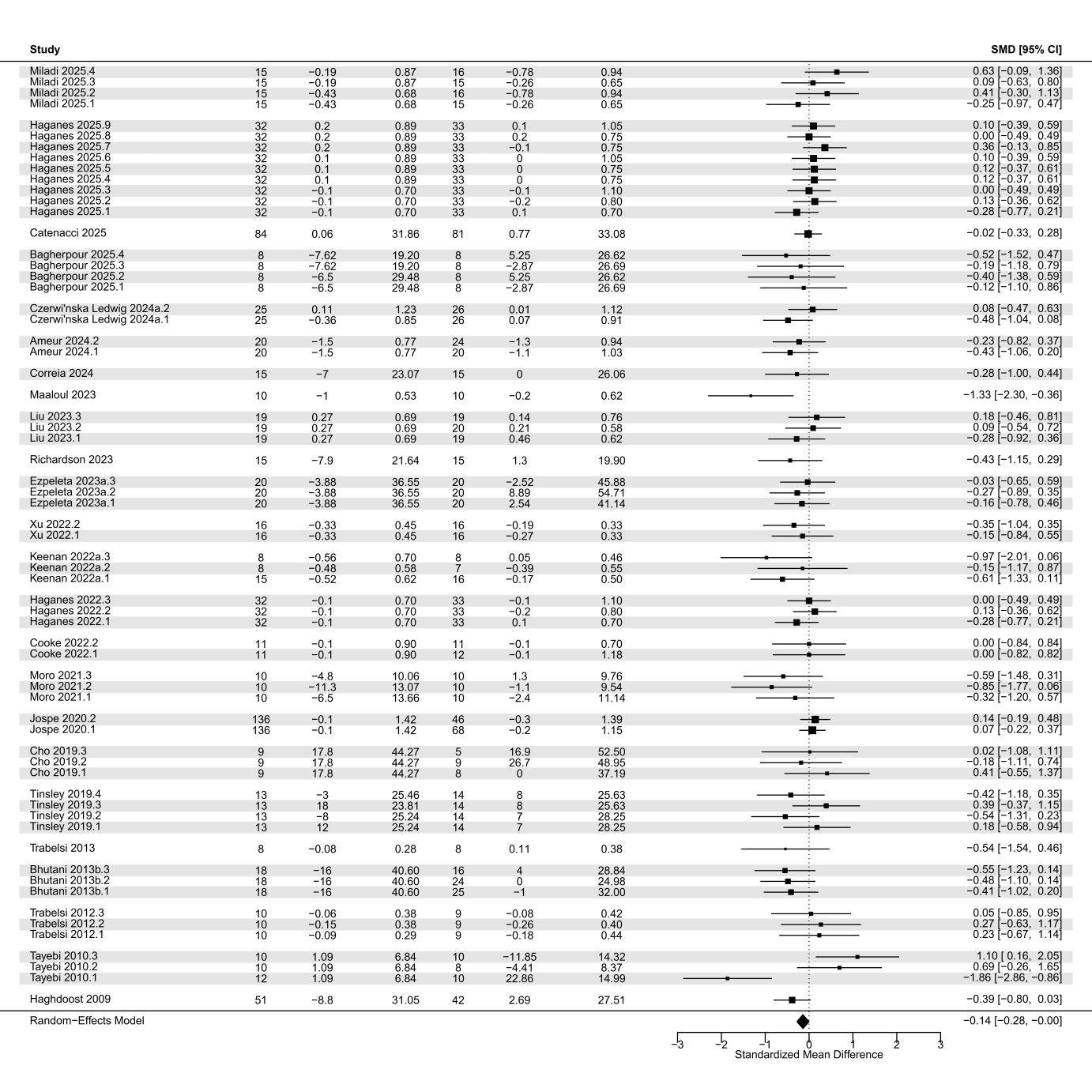


**Supplementary Fig.1L (Forest plots of low density lipoprotein)**


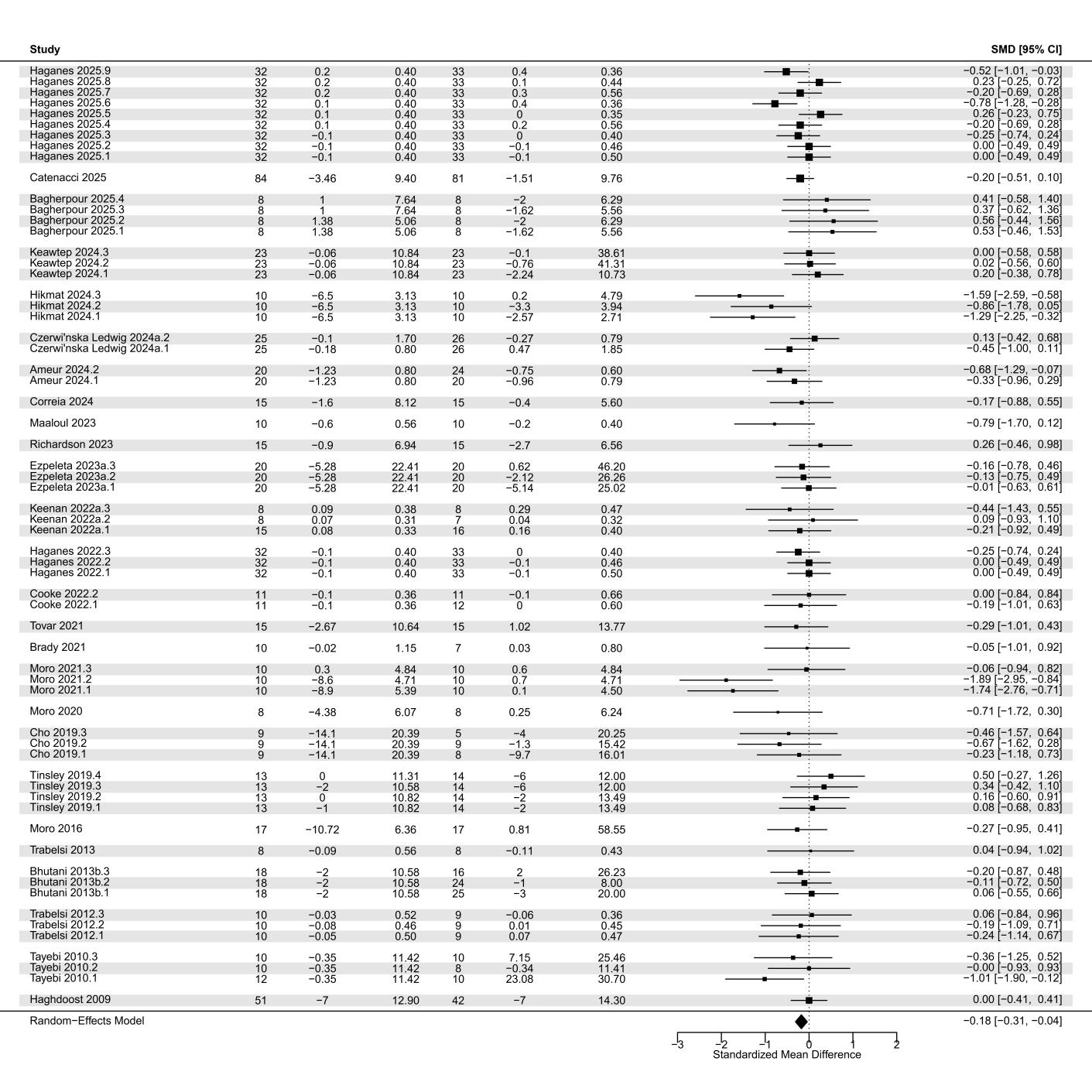


**Supplementary Fig.1M (Forest plots of fasting glucose)**


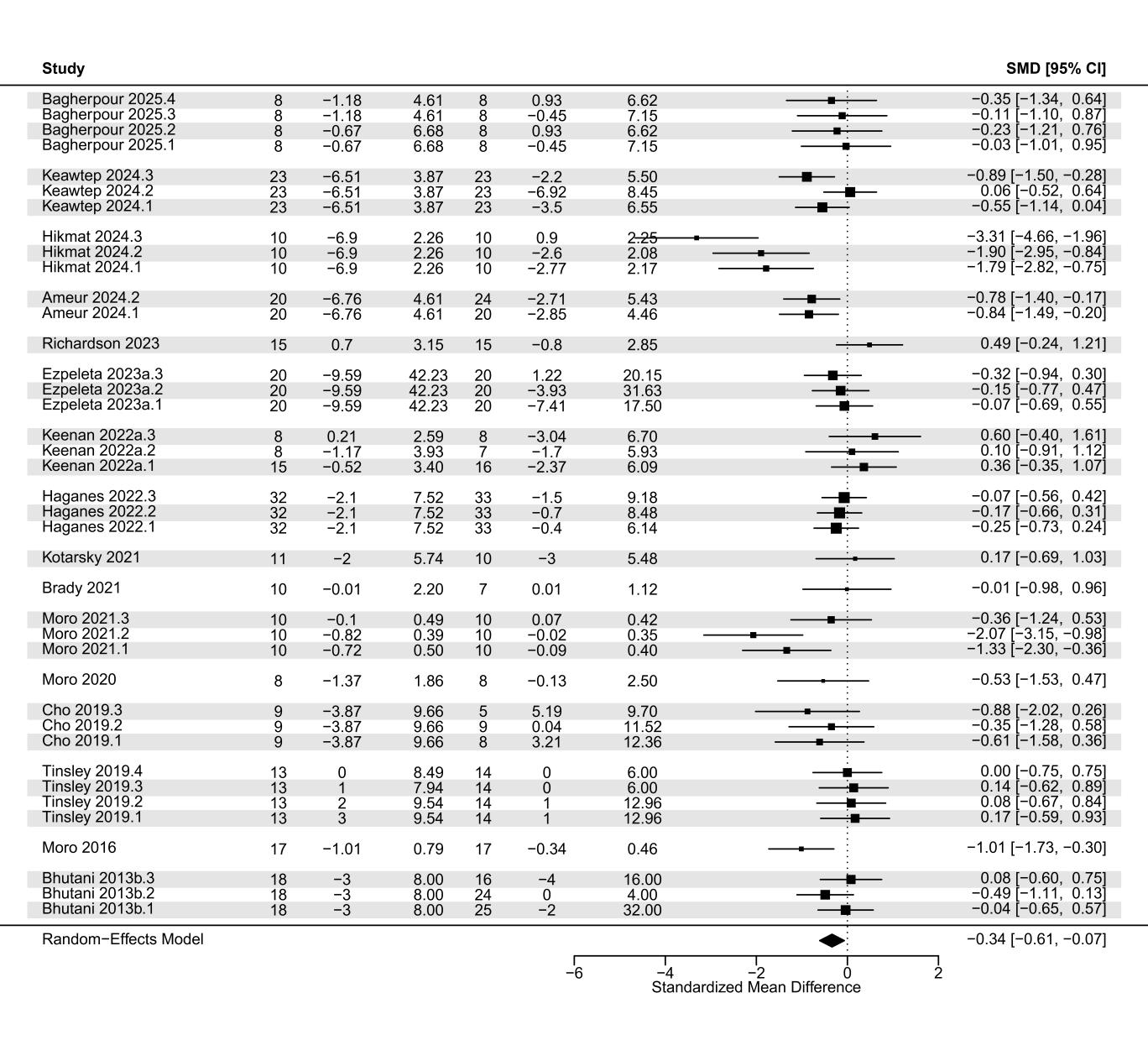


**Supplementary Fig.1N (Forest plots of insulin)**


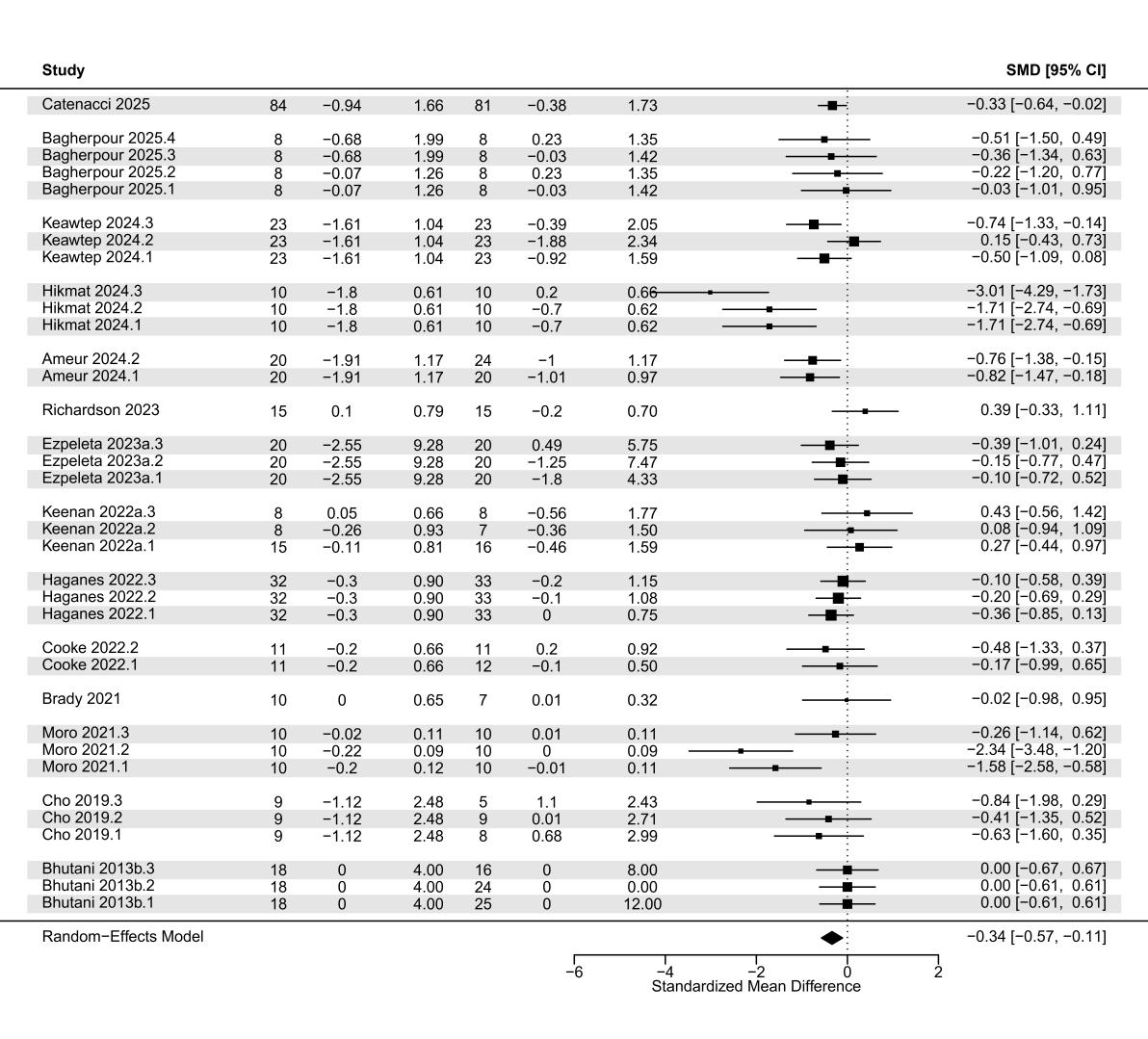


**Supplementary Fig.1O (Forest plots of HOMA-IR)**


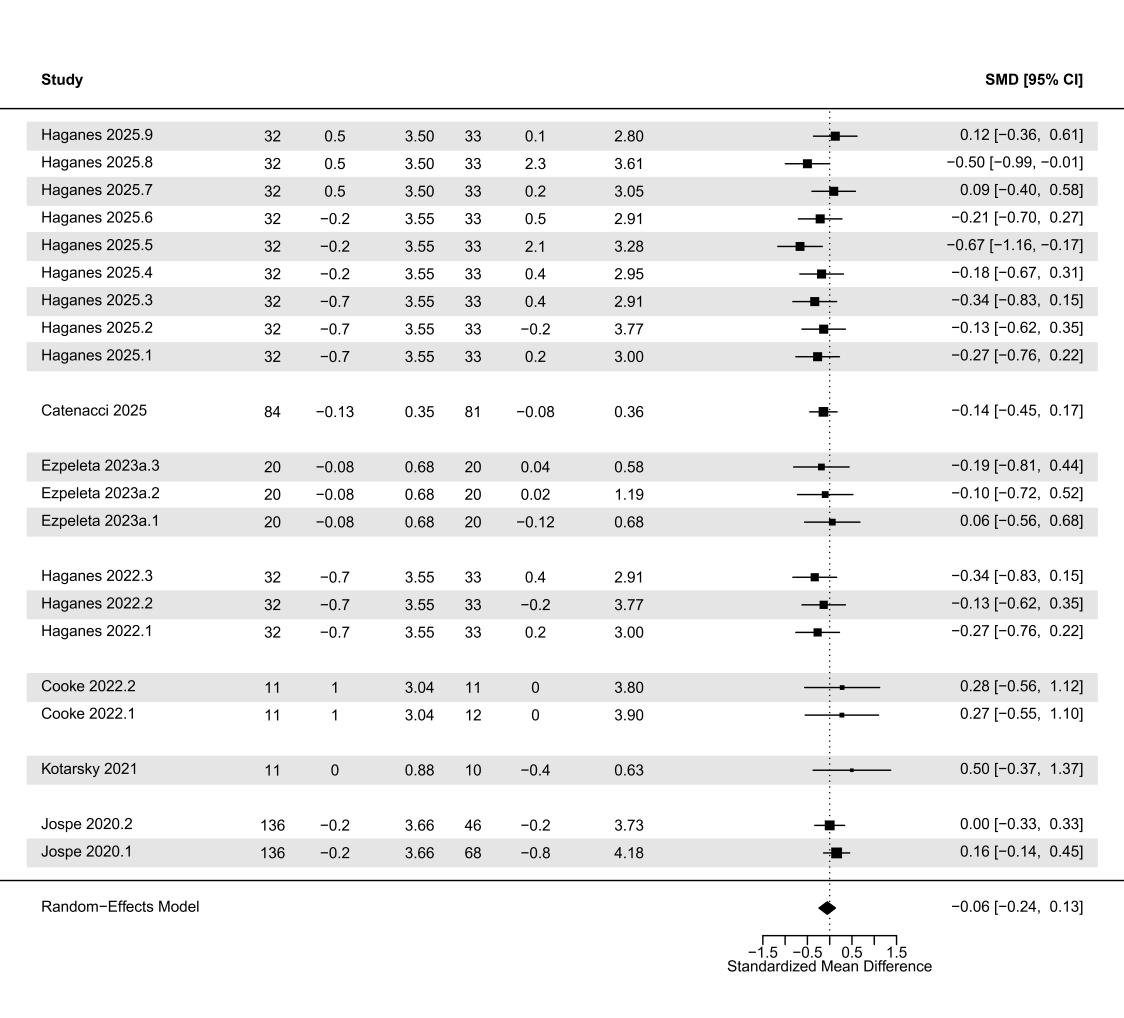


**Supplementary Fig.1P (Forest plots of HbA1C)**


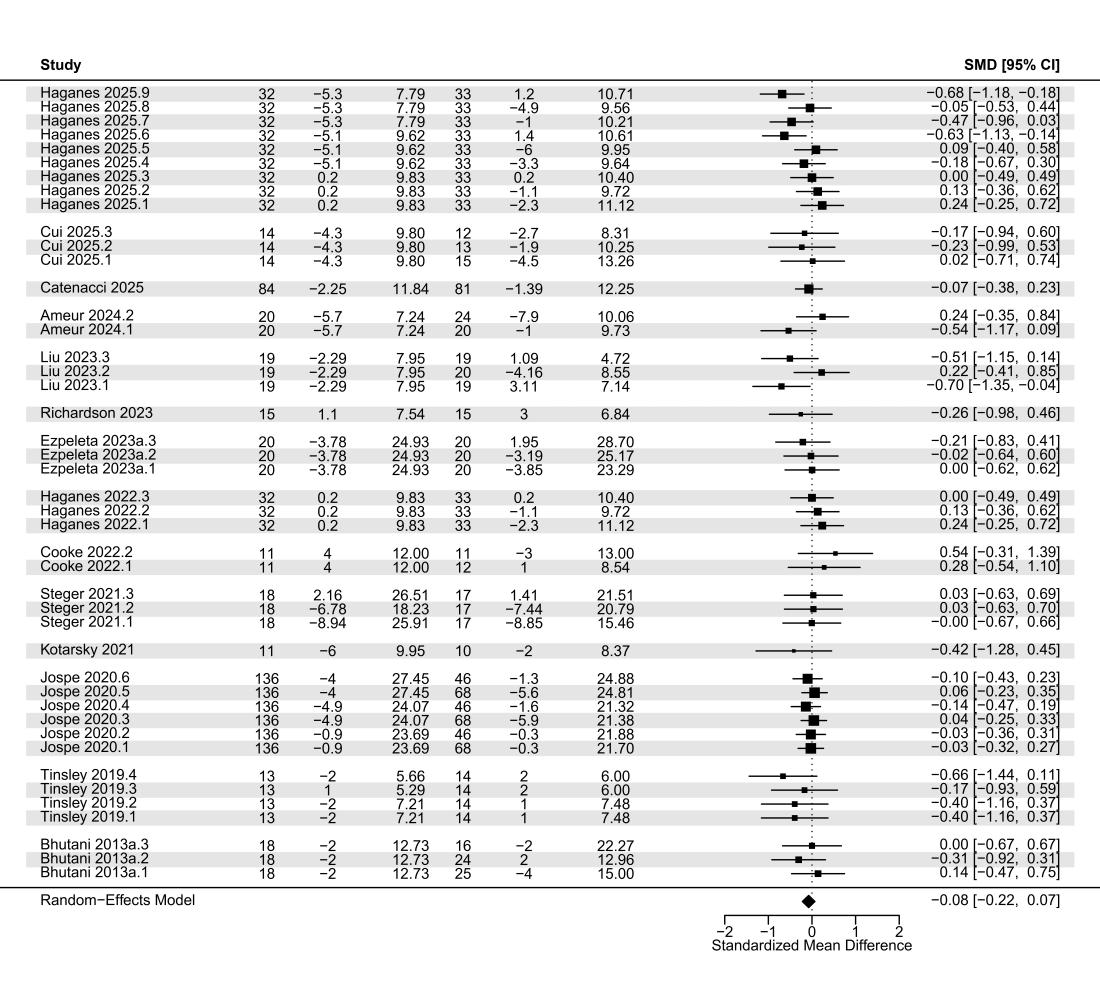


**Supplementary Fig.1Q (Forest plots of systolic blood pressure)**


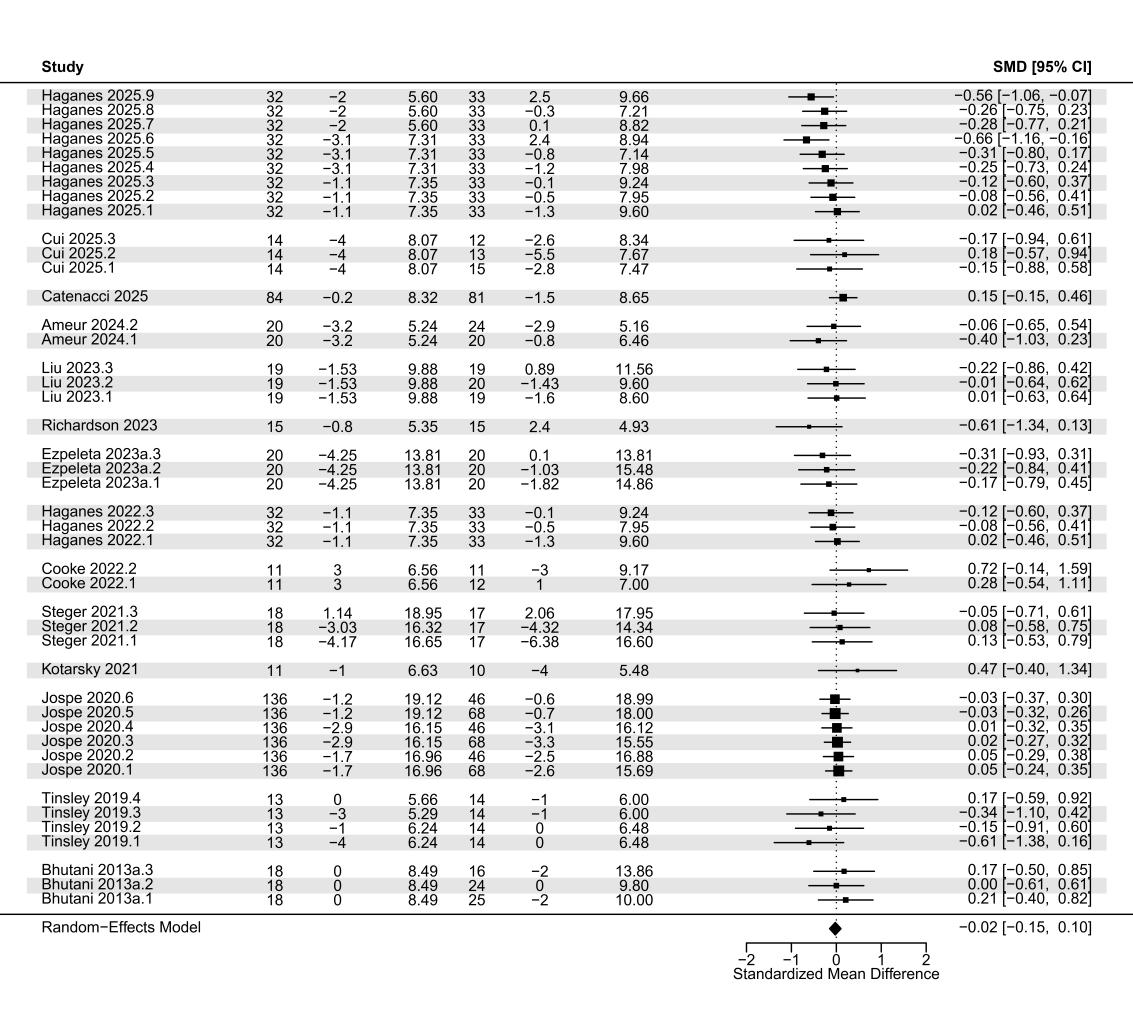


**Supplementary Fig.1R (Forest plots of diastolic blood pressure)**


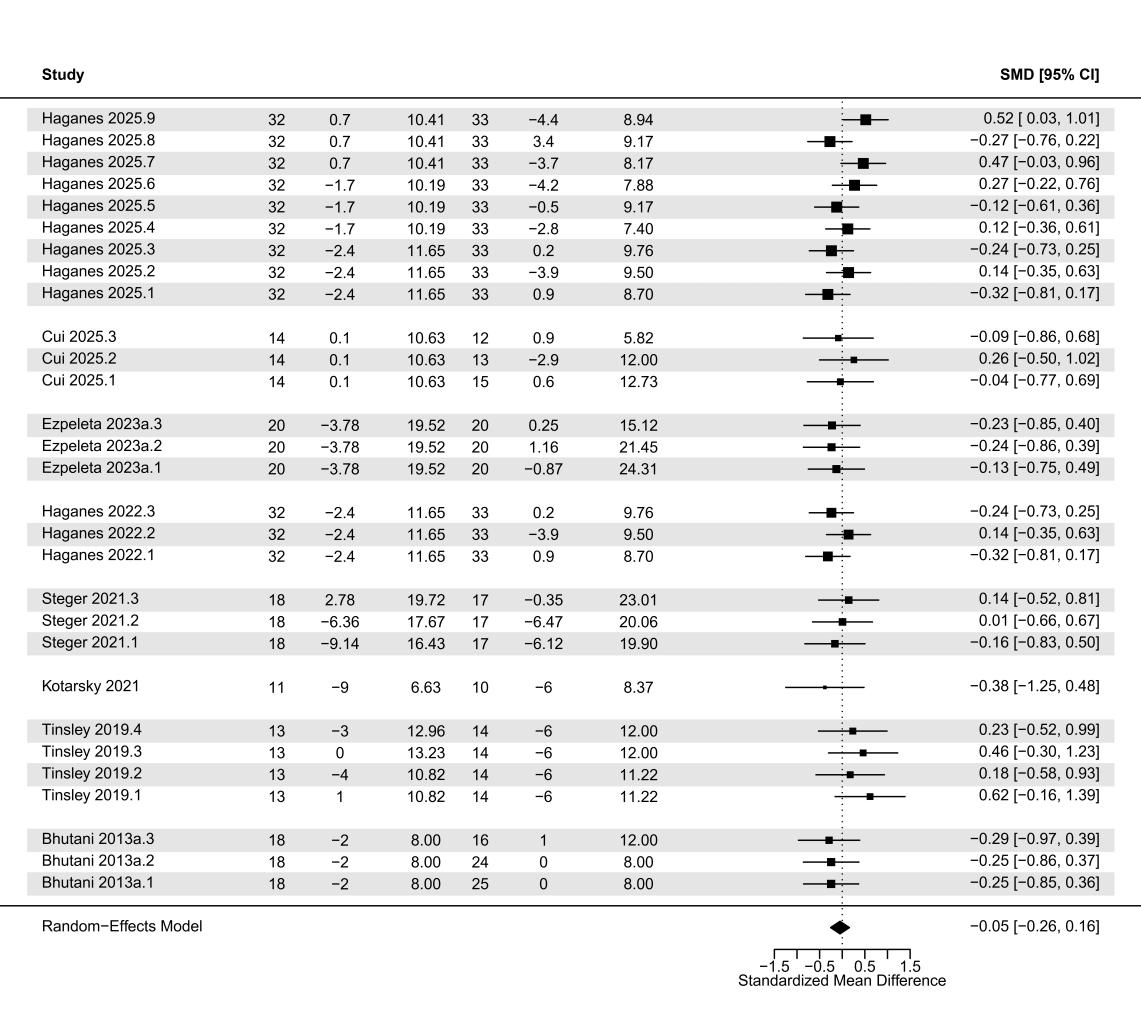


**Supplementary Fig.1S (Forest plots of heart rate)**


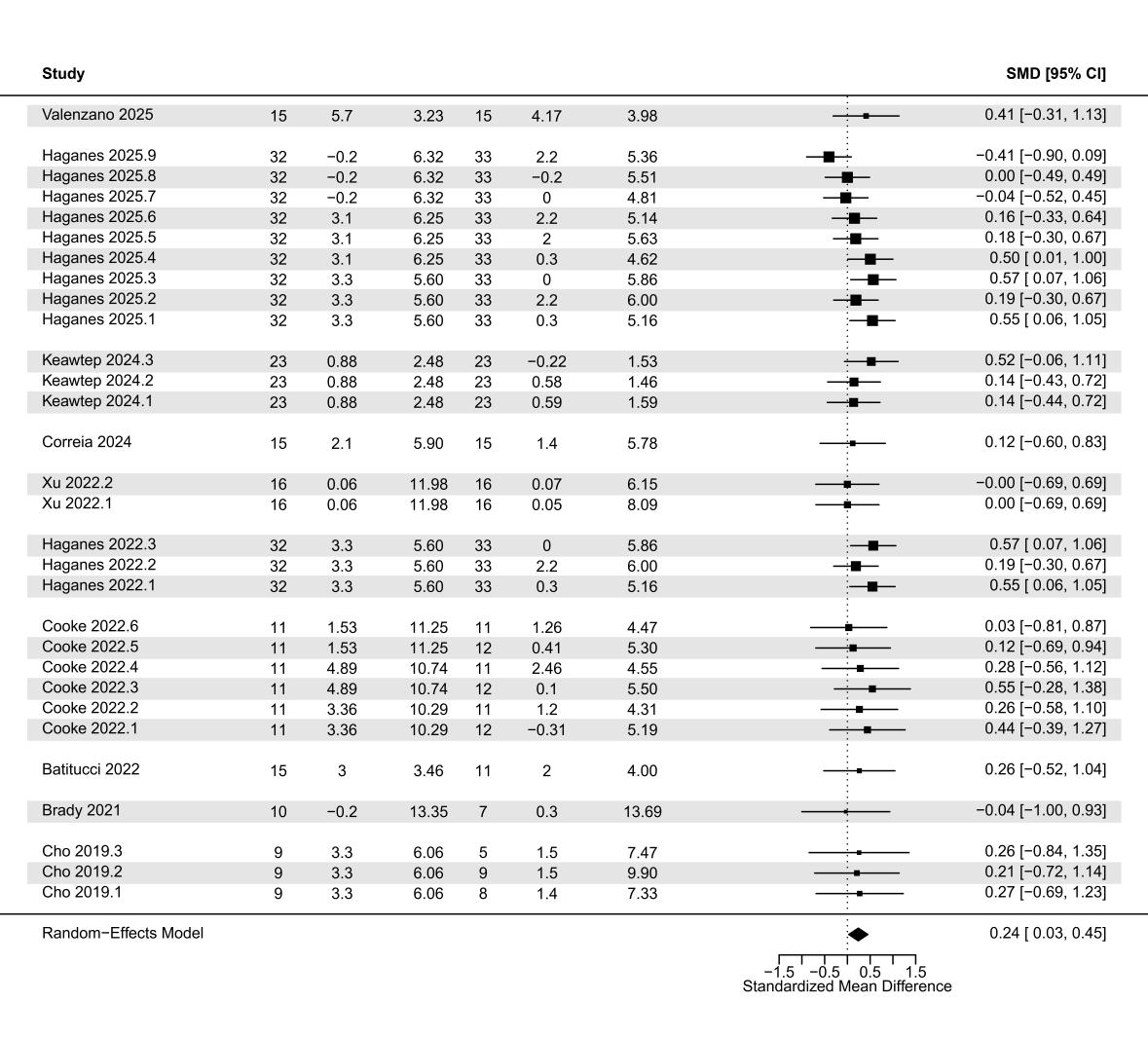


**Supplementary Fig.1T (Forest plots of VO_2_ max)**


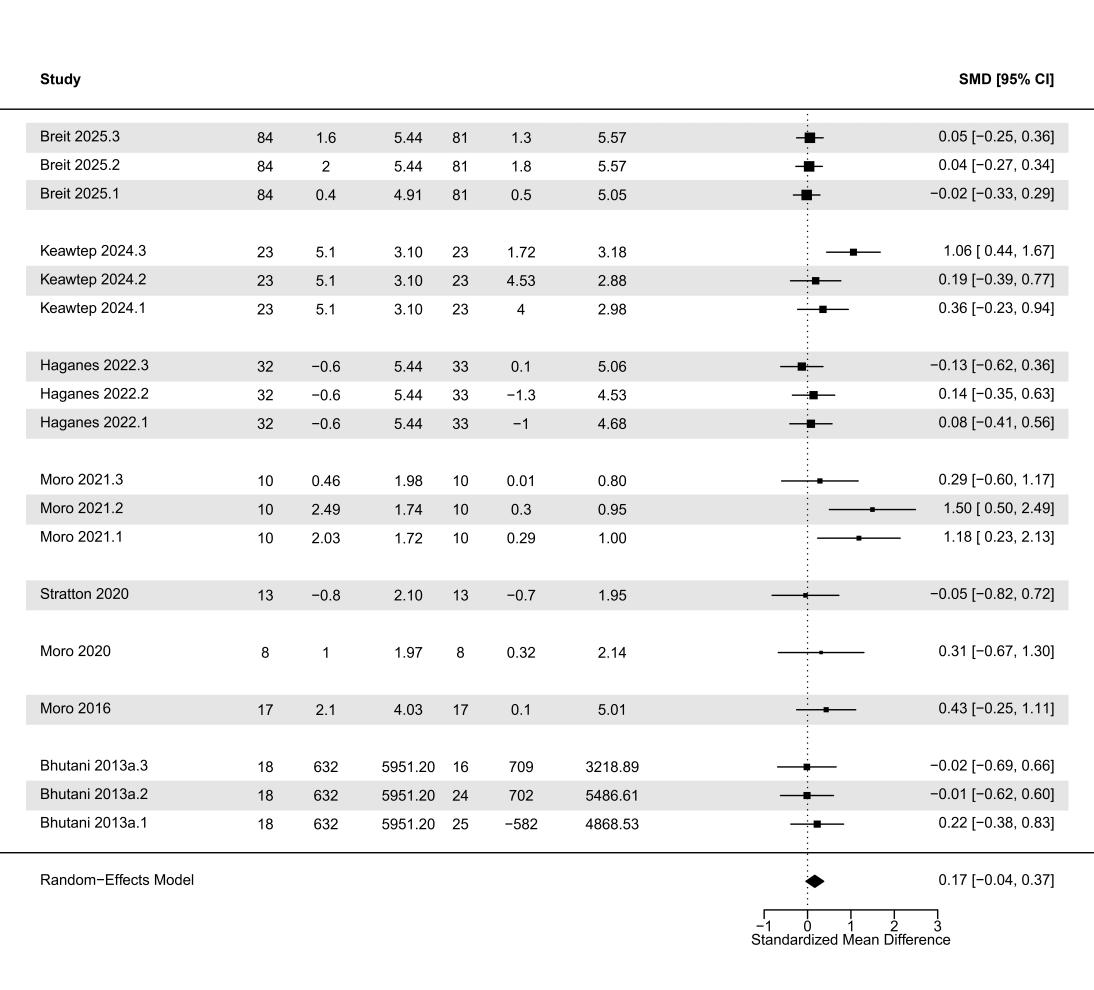


**Supplementary Fig.1U (Forest plots of adiponection)**


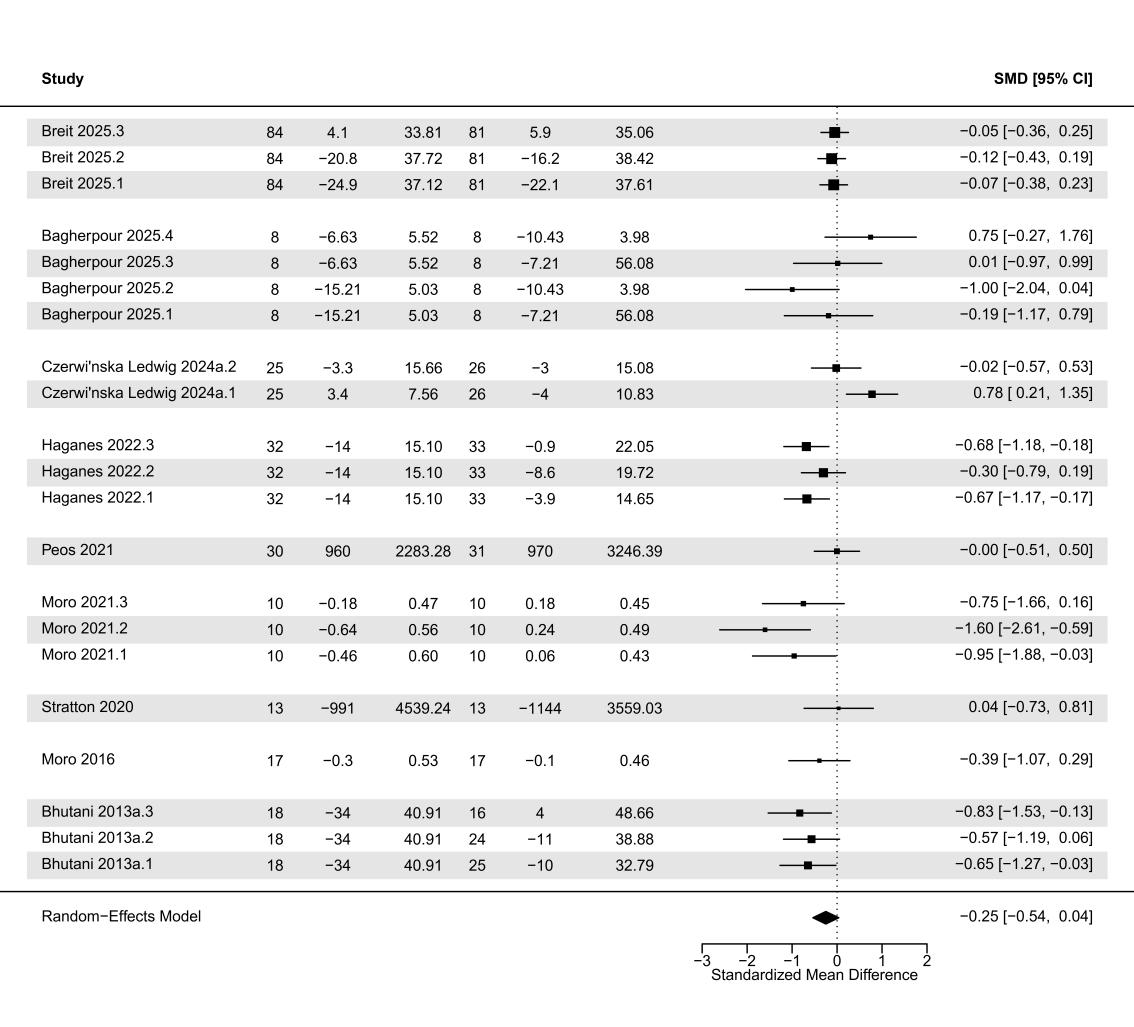


**Supplementary Fig.1V (Forest plots of leptin)**


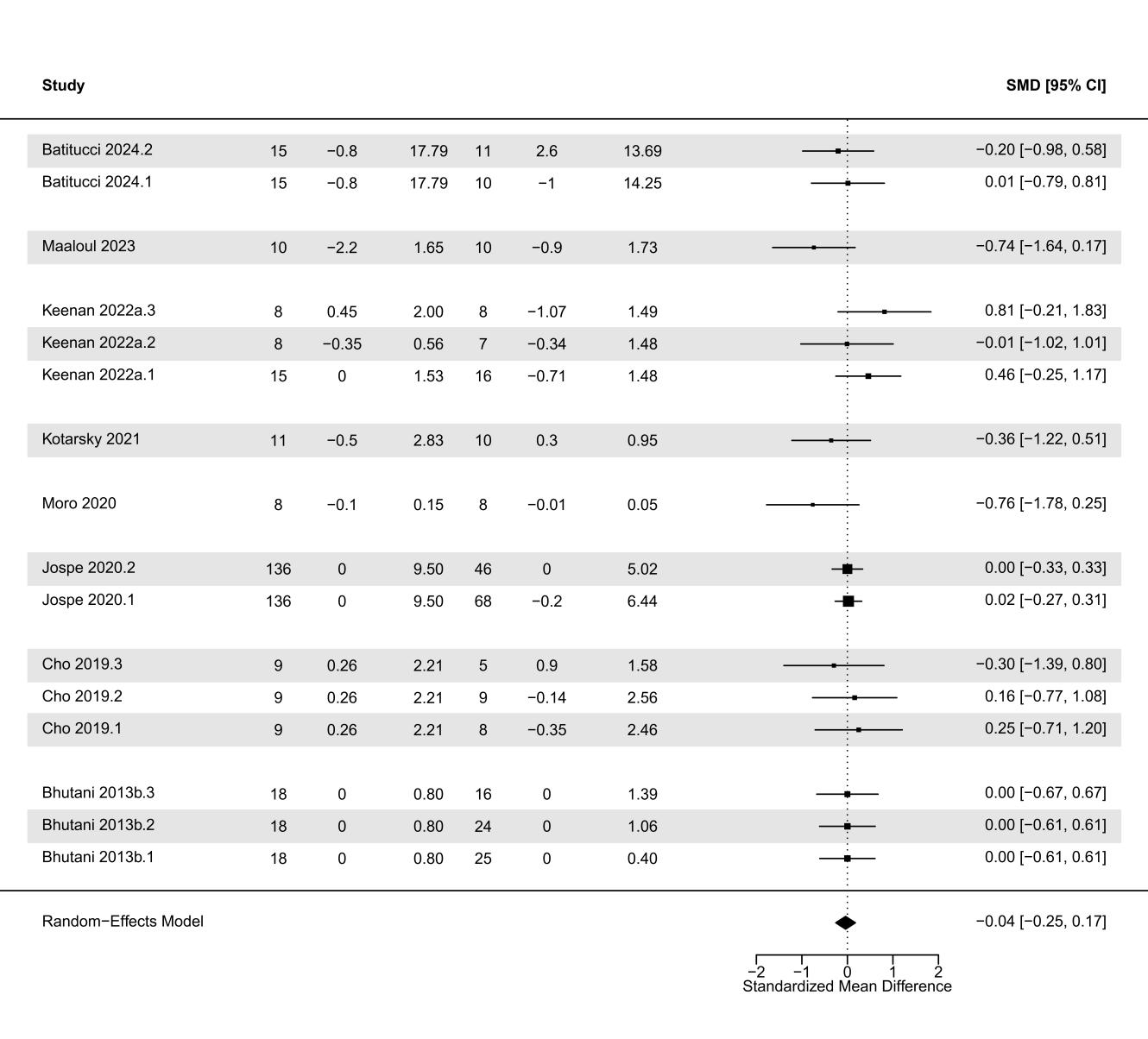


**Supplementary Fig.1W (Forest plots of CRP)**


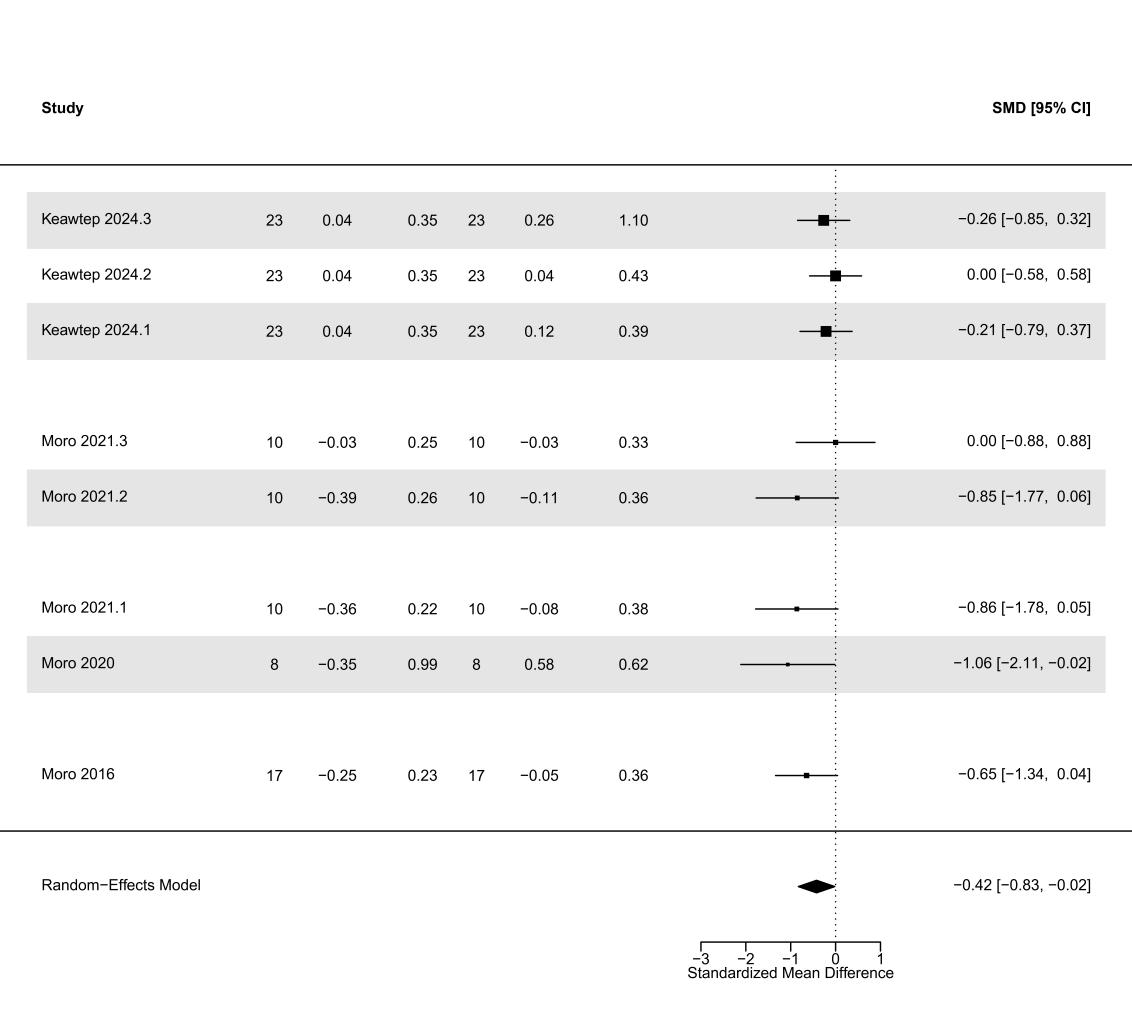


**Supplementary Fig.1X (Forest plots of IL-6)**


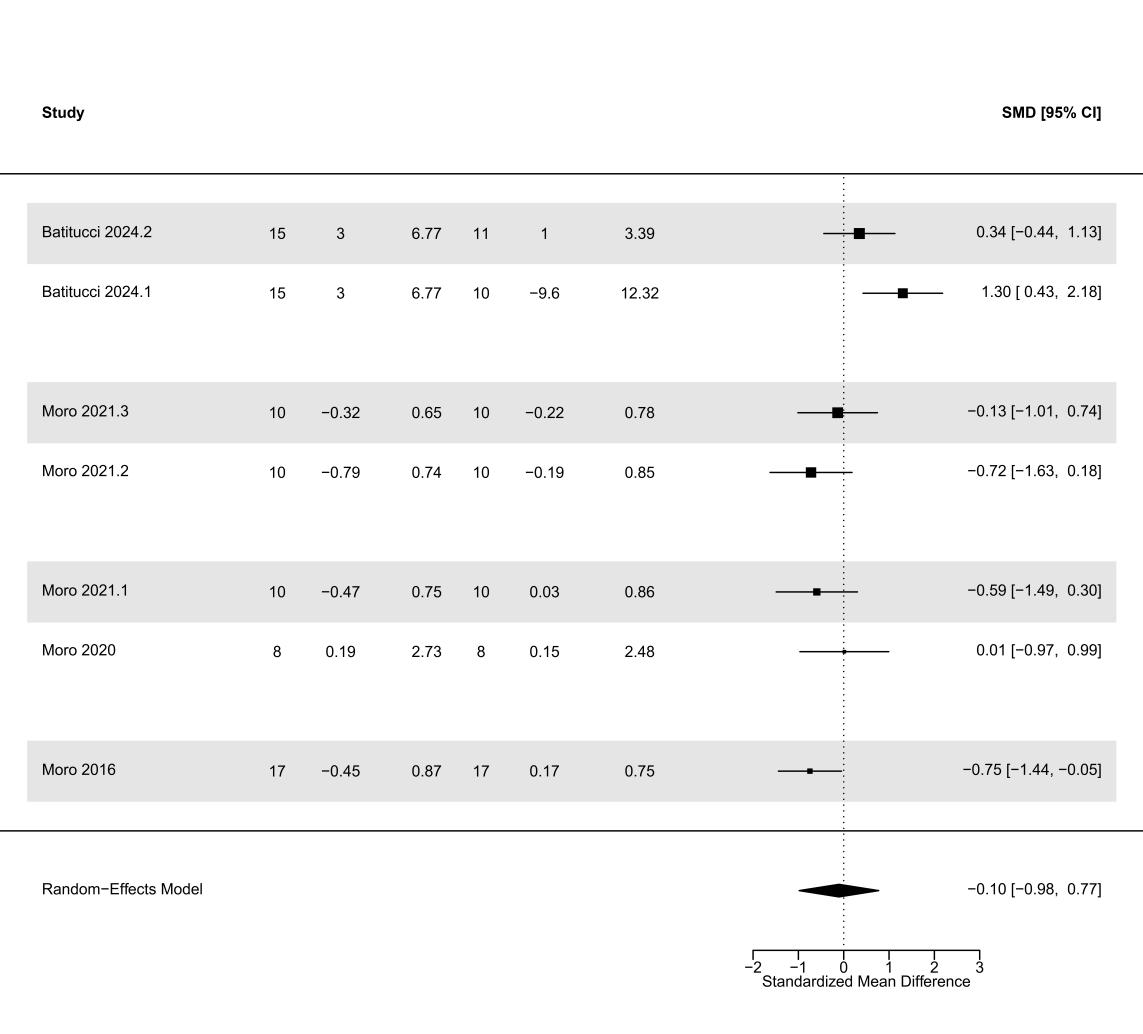


**Supplementary Fig.1Y (Forest plots of TNF-α)**


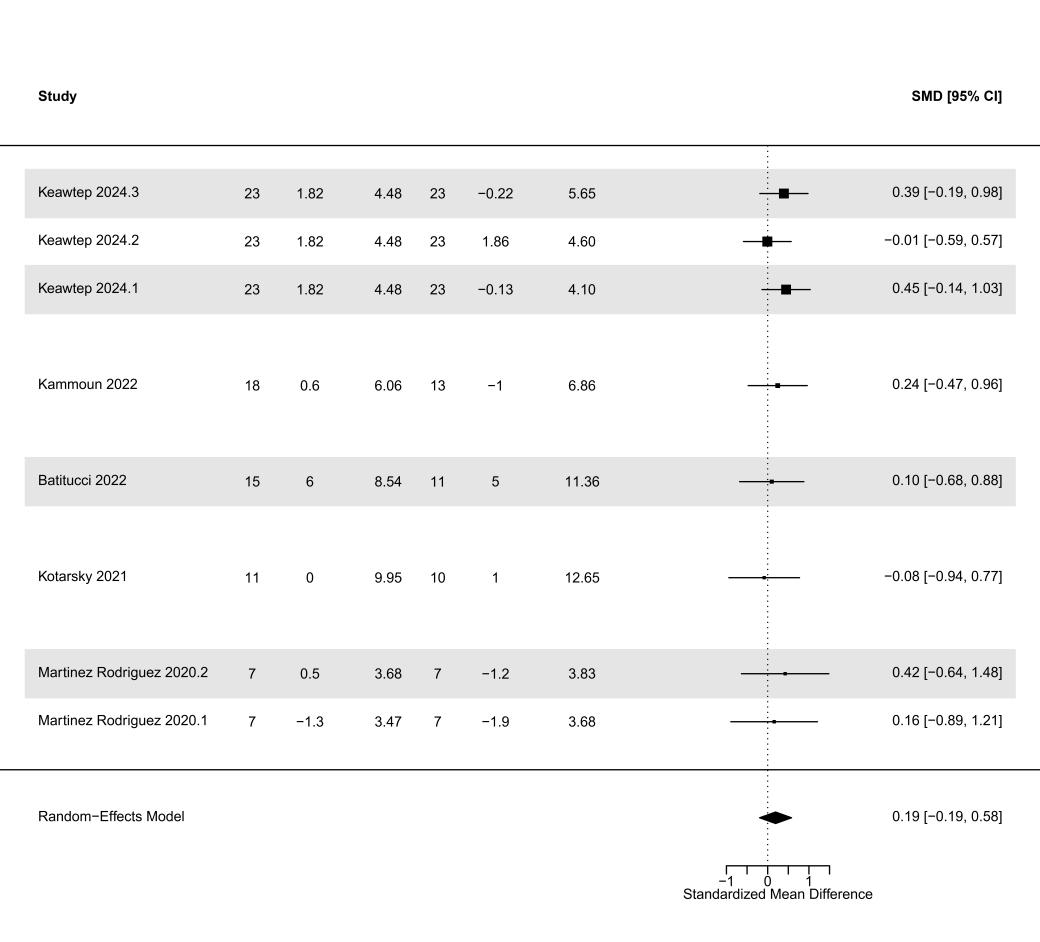


**Supplementary Fig.1Z (Forest plots of handgrip)**


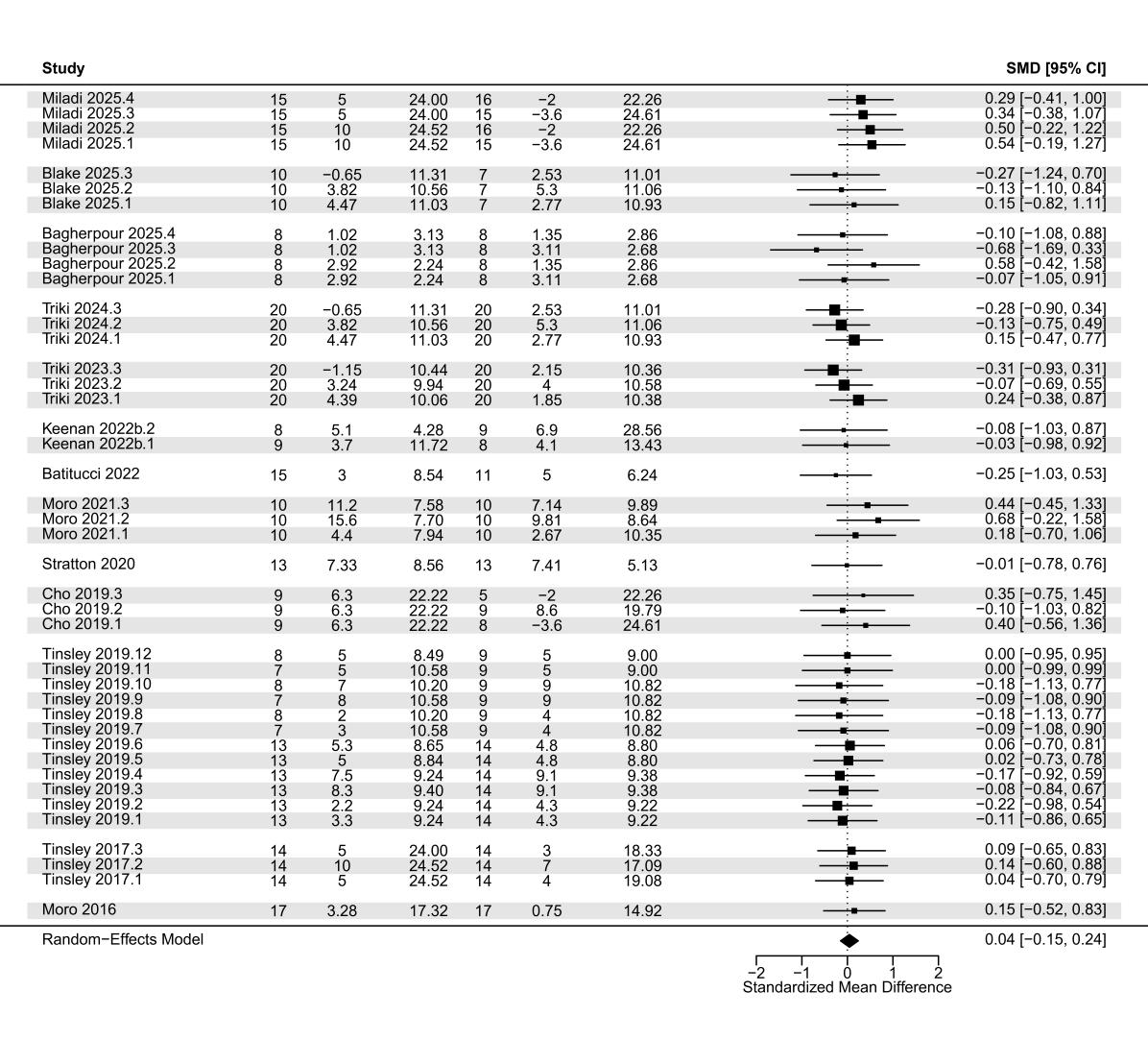


**Supplementary Fig.1AA (Forest plots of bench press)**


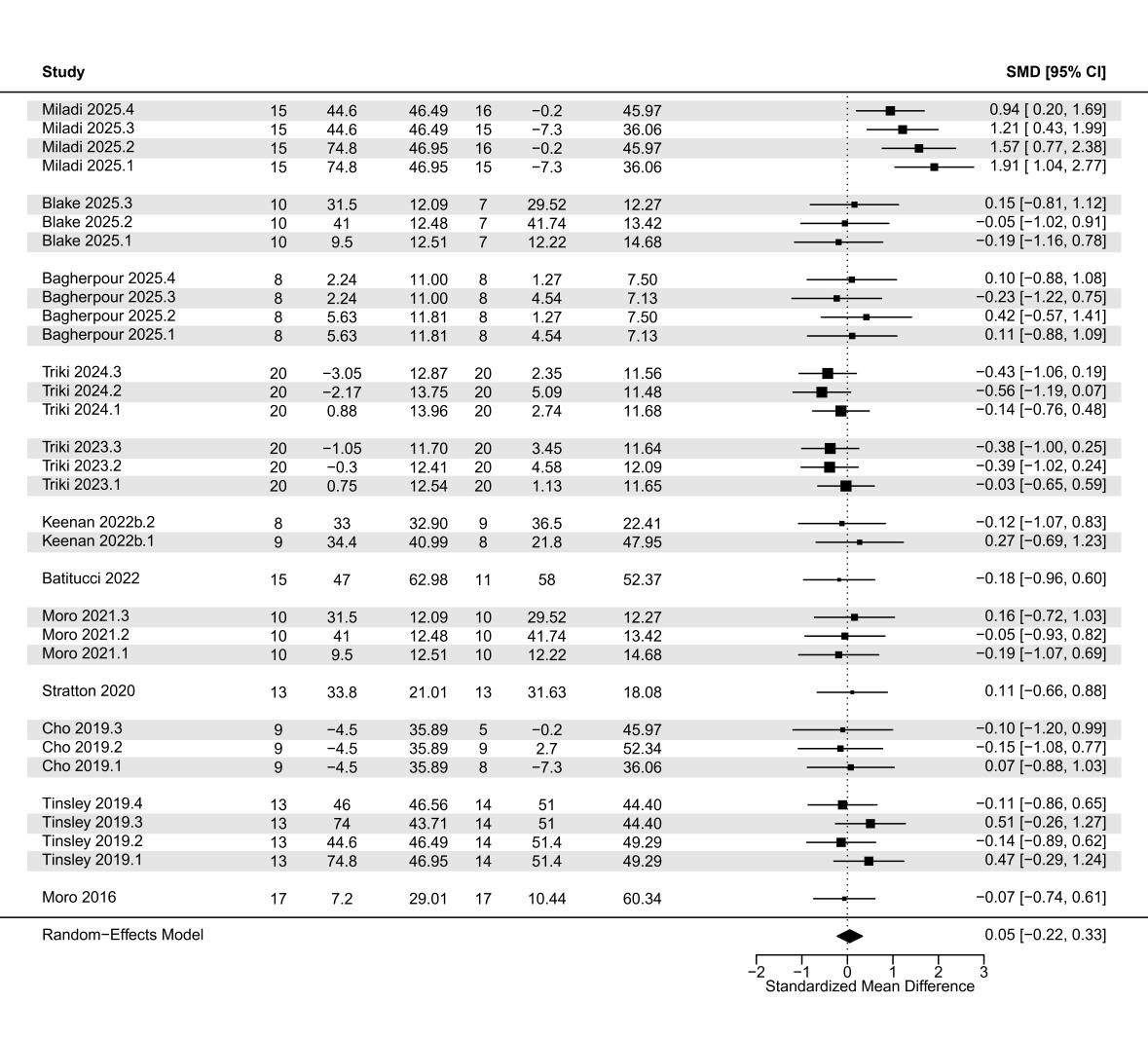


**Supplementary Fig.1AB (Forest plots of leg press)**


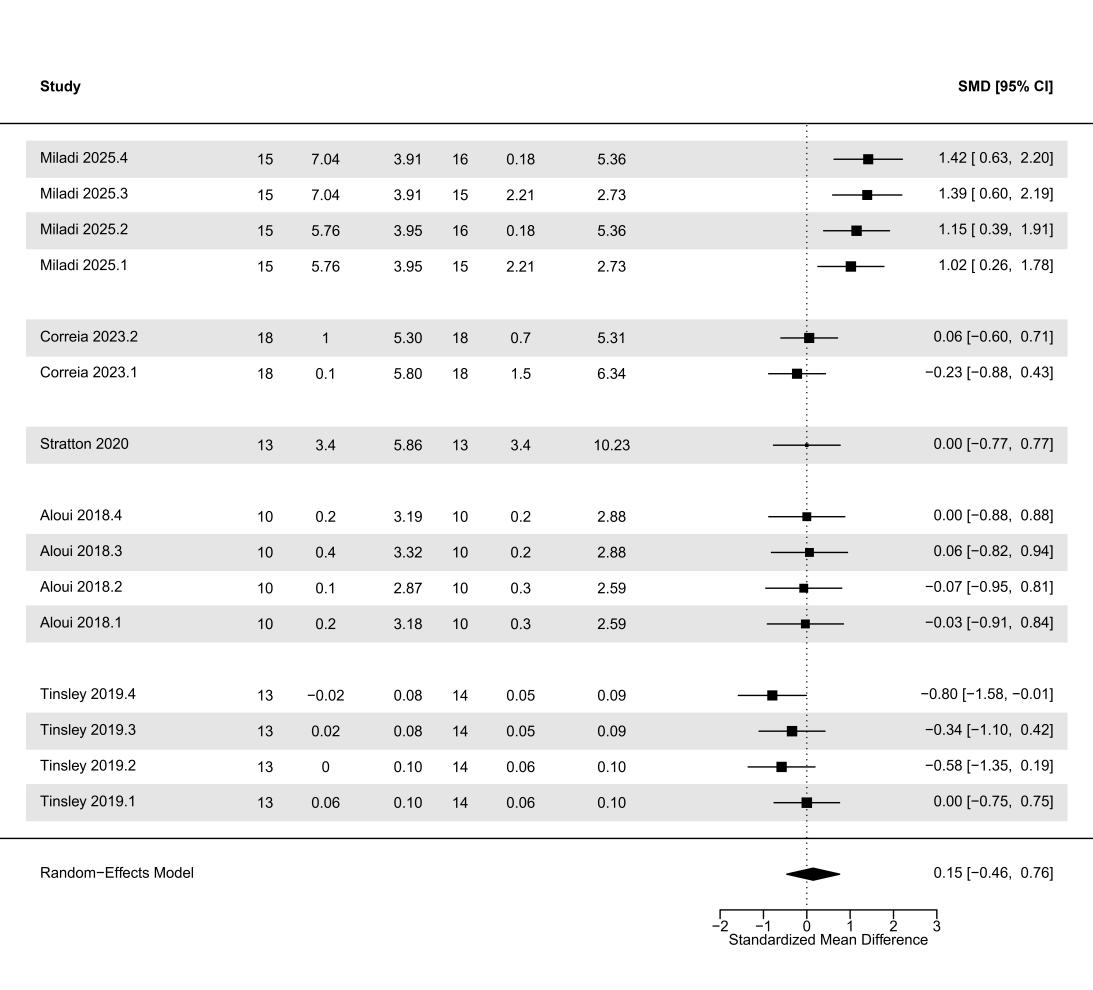


**Supplementary Fig.1AC (Forest plots of jump height)**

**
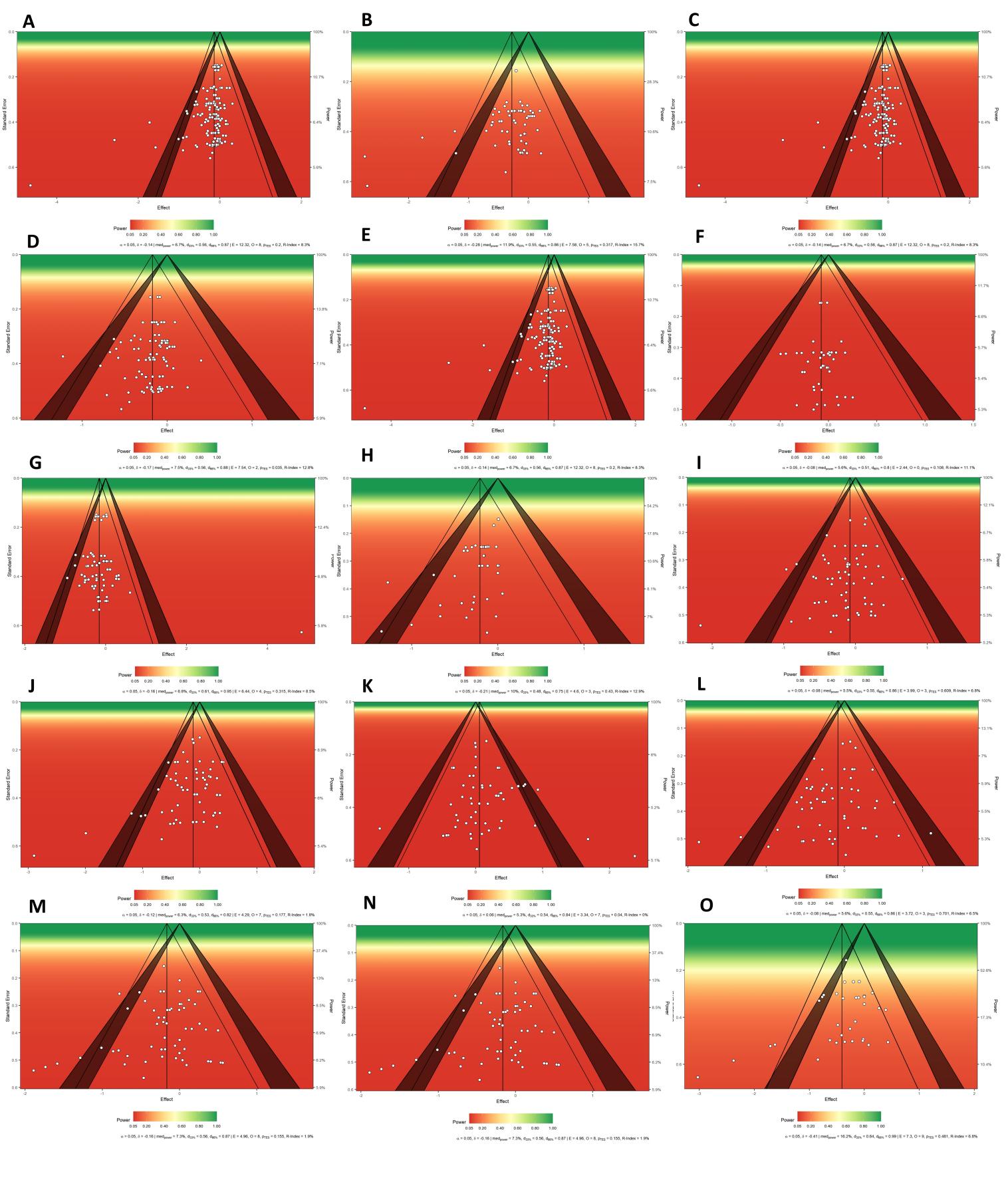
**

**Supplementary Fig.2A (Power visualization)** The vertical solid line represents the pooled effect size, and the vertical dash line represents the adjusted pooled effect size. Significance contours at .05 and .01 levels are noted by the shaded area. ***manpower*** indicates the median power of all included effect sizes. ***d33%*** and ***d66%*** indicate the true effect sizes necessary for achieving 33% and 66% levels of median power. ***E, O, and PTES*** show the results of a test of excess significance. ***R-index*** denotes the expected replicability of findings. From A to

AC, they are body mass, BMI, body fat%, fat mass, fat free mass, lean body mass, waist circumference, visceral adipose tissue, total cholesterol, triglyceride, high density lipoprotein, low density lipoprotein, fasting glucose, insulin, HOMA-IR, HAb1c, systolic blood pressure, diastolic blood pressure, heart rate, VO2max, adipnection, leptin, CRP, IL-6, TNF-α, handgrip, bench press, leg press, jump height.

**
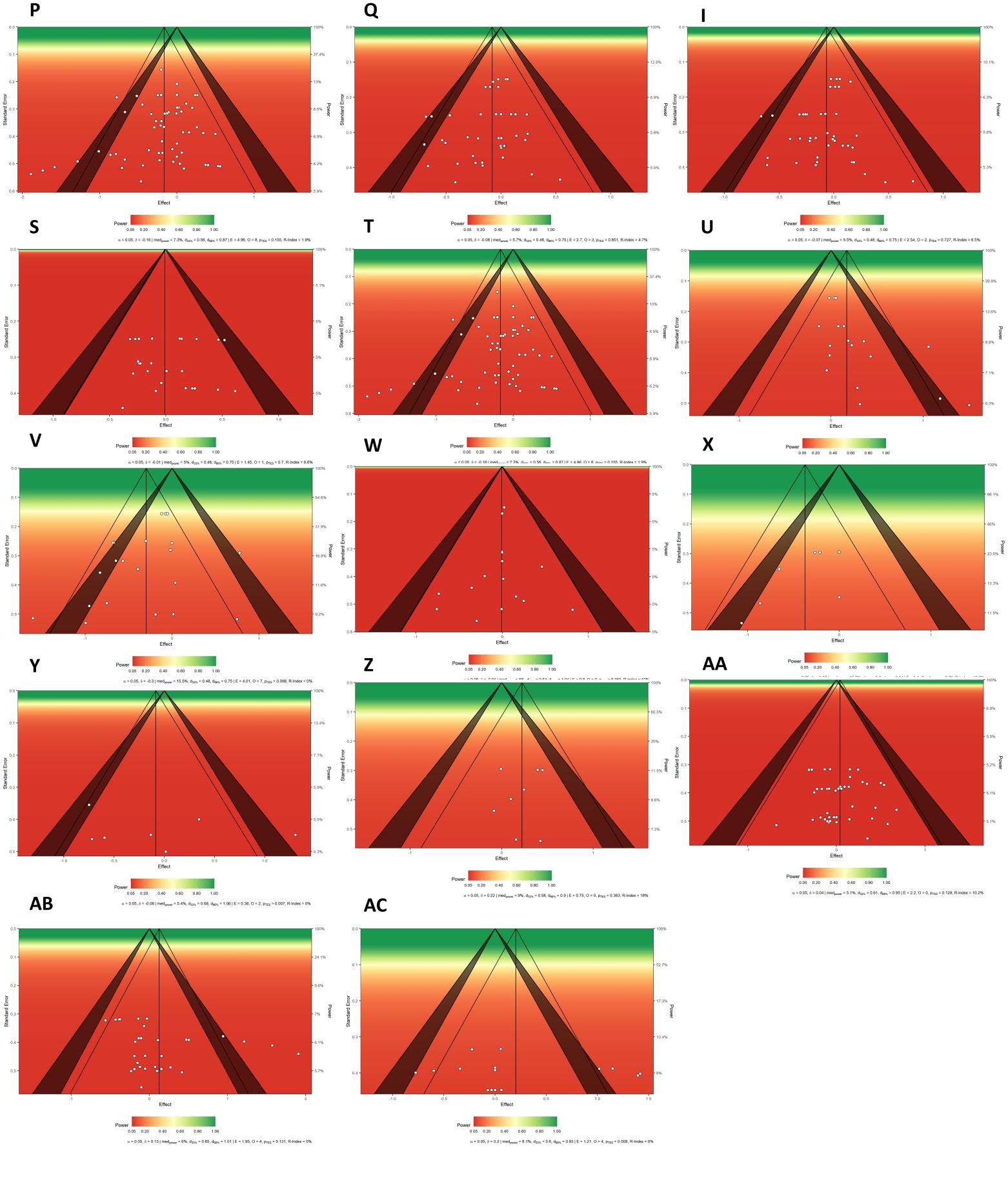
**

**Supplementary Fig.2B (Power visualization)**


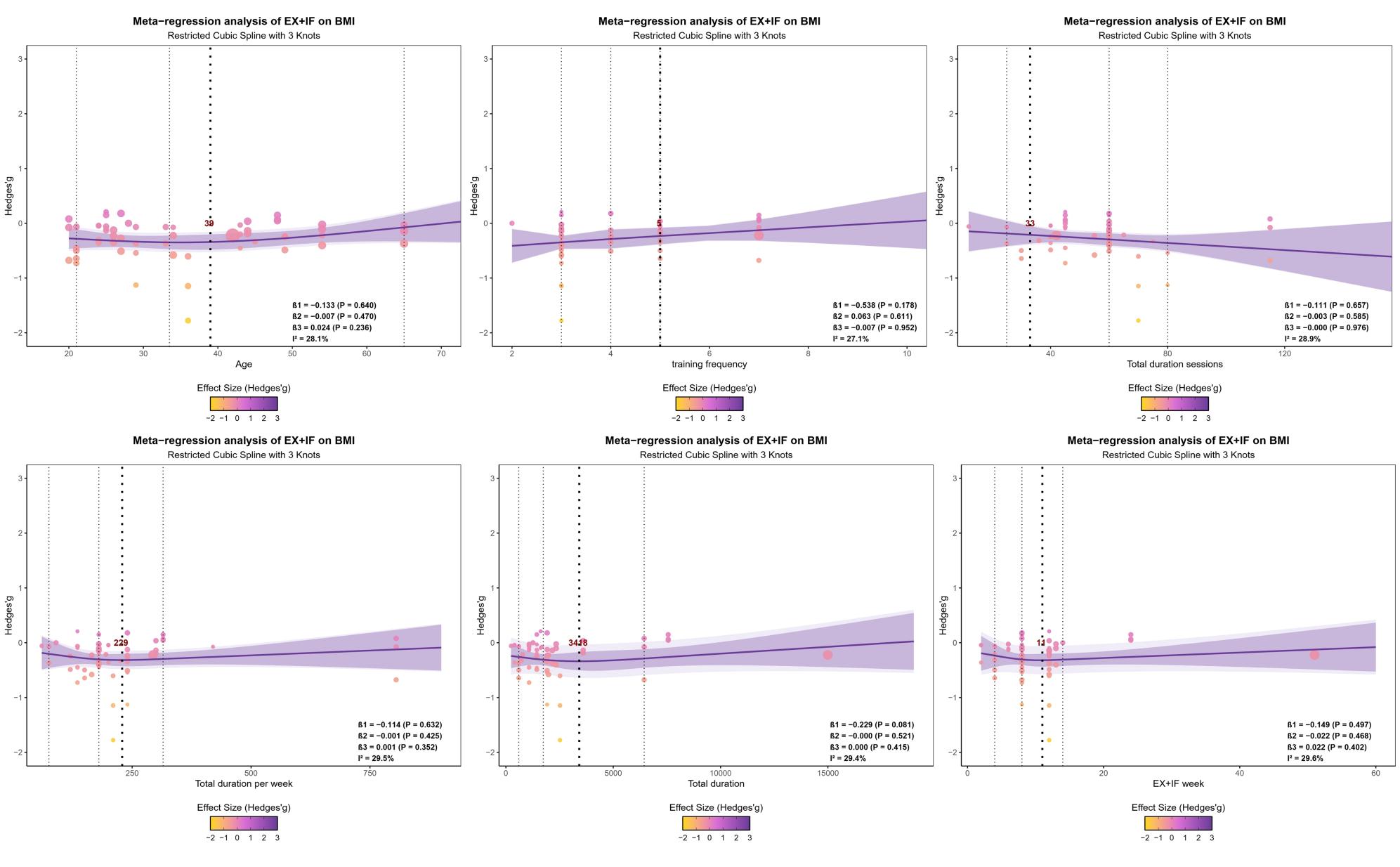


**Supplementary Fig.3A (Meta-regression analysis of EX+IF on BMI)** β0 represents the intercept; β1, β2, and β3 represent the slopes; *I*^2^ means heterogeneity; the purple shaded part represents the 95% confidence interval.


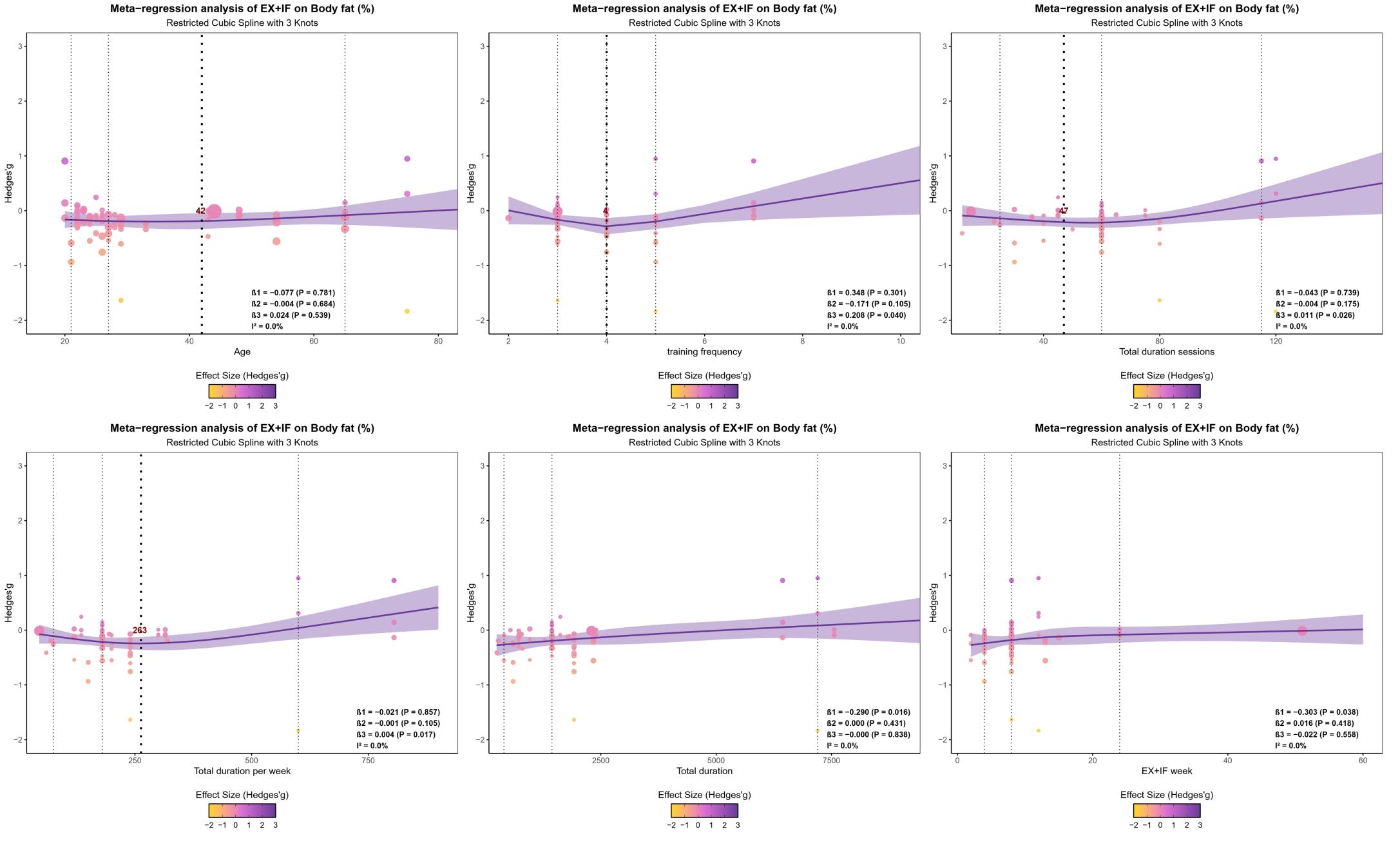


**Supplementary Fig.3B (Meta-regression analysis of EX+IF on body fat%)** β0 represents the intercept; β1, β2, and β3 represent the slopes; *I*^2^ means heterogeneity; the purple shaded part represents the 95% confidence interval.


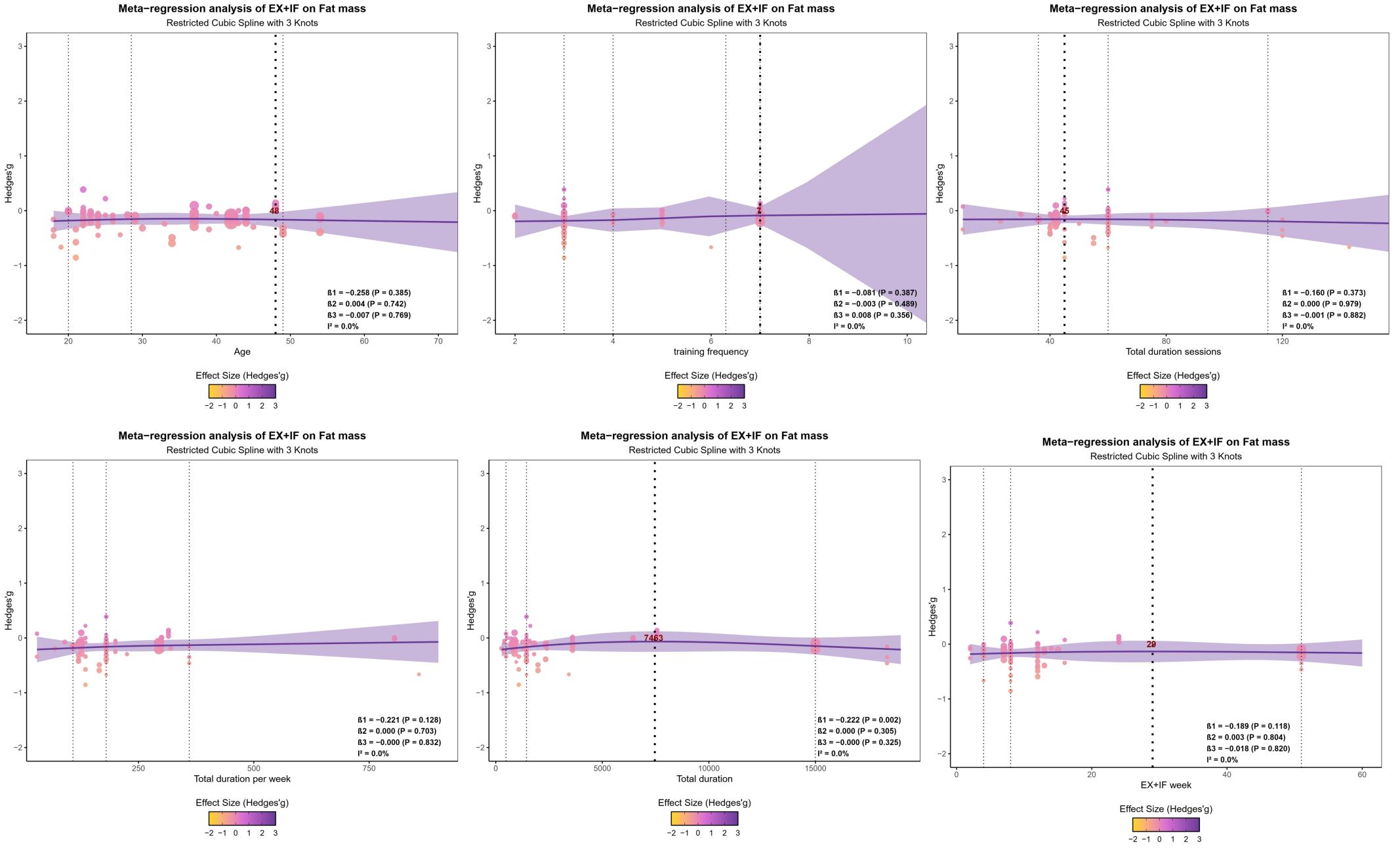


**Supplementary Fig.3C (Meta-regression analysis of EX+IF on fat mass)** β0 represents the intercept; β1, β2, and β3 represent the slopes; *I*^2^ means heterogeneity; the purple shaded part represents the 95% confidence interval.


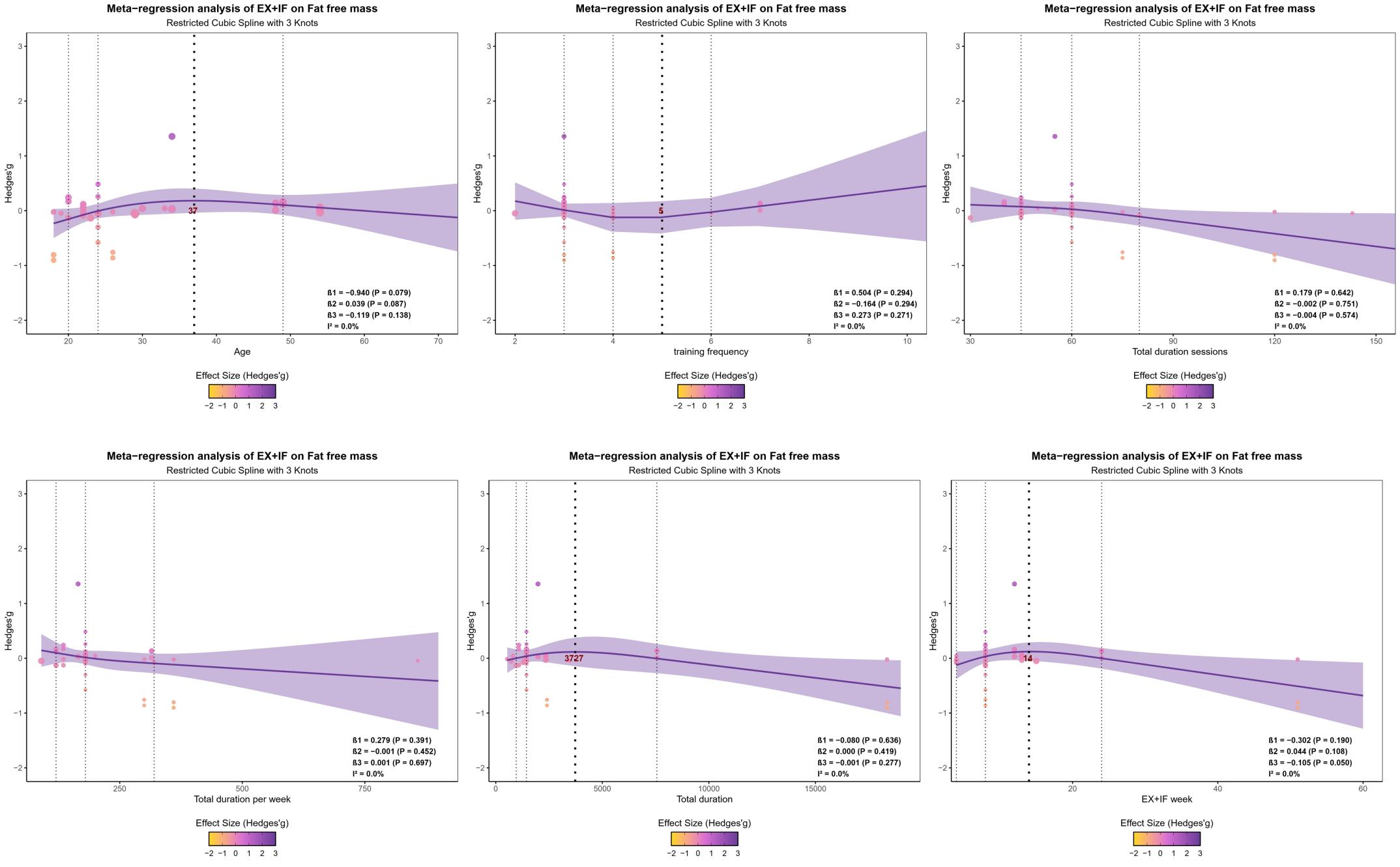


**Supplementary Fig.3D (Meta-regression analysis of EX+IF on fat free mass)** β0 represents the intercept; β1, β2, and β3 represent the slopes; *I*^2^ means heterogeneity; the purple shaded part represents the 95% confidence interval.


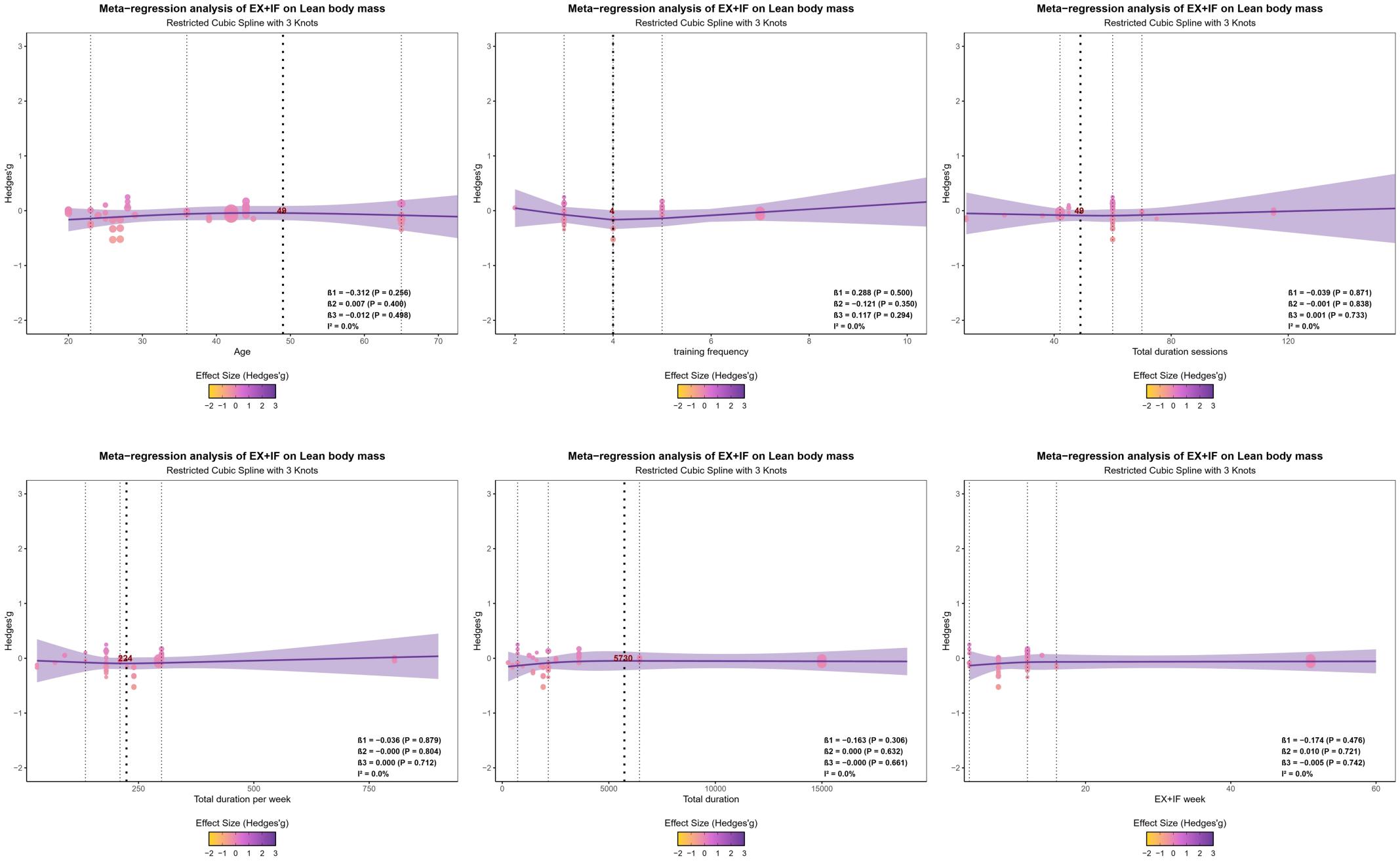


**Supplementary Fig.3E (Meta-regression analysis of EX+IF on lean body mass)** β0 represents the intercept; β1, β2, and β3 represent the slopes; *I*^2^ means heterogeneity; the purple shaded part represents the 95% confidence interval.


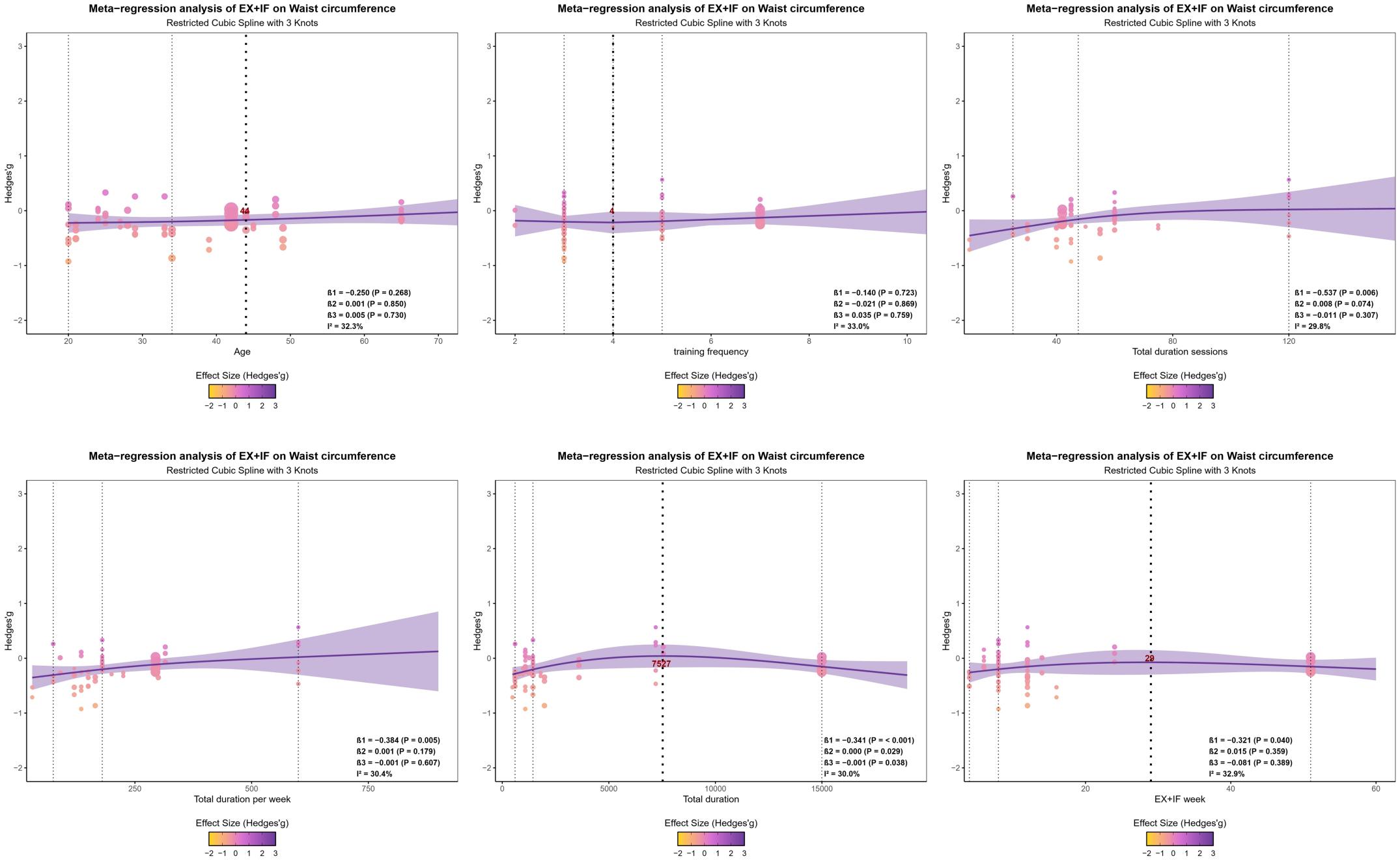


**Supplementary Fig.3F (Meta-regression analysis of EX+IF on waist circumference)** β0 represents the intercept; β1, β2, and β3 represent the slopes; *I*^2^ means heterogeneity; the purple shaded part represents the 95% confidence interval.


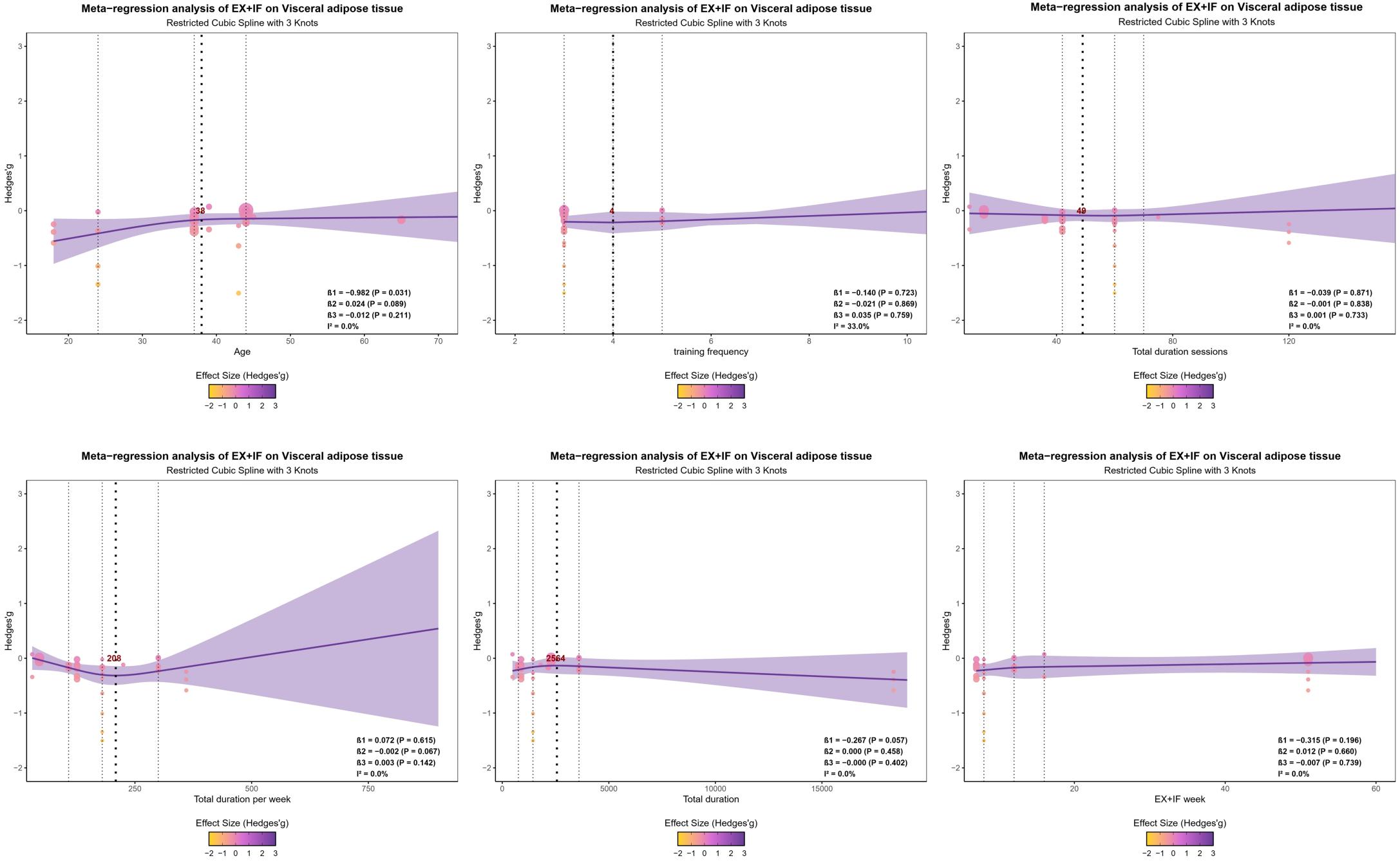


**Supplementary Fig.3G (Meta-regression analysis of EX+IF on visceral adipose tissue)** β0 represents the intercept; β1, β2, and β3 represent the slopes; *I*^2^ means heterogeneity; the purple shaded part represents the 95% confidence interval.


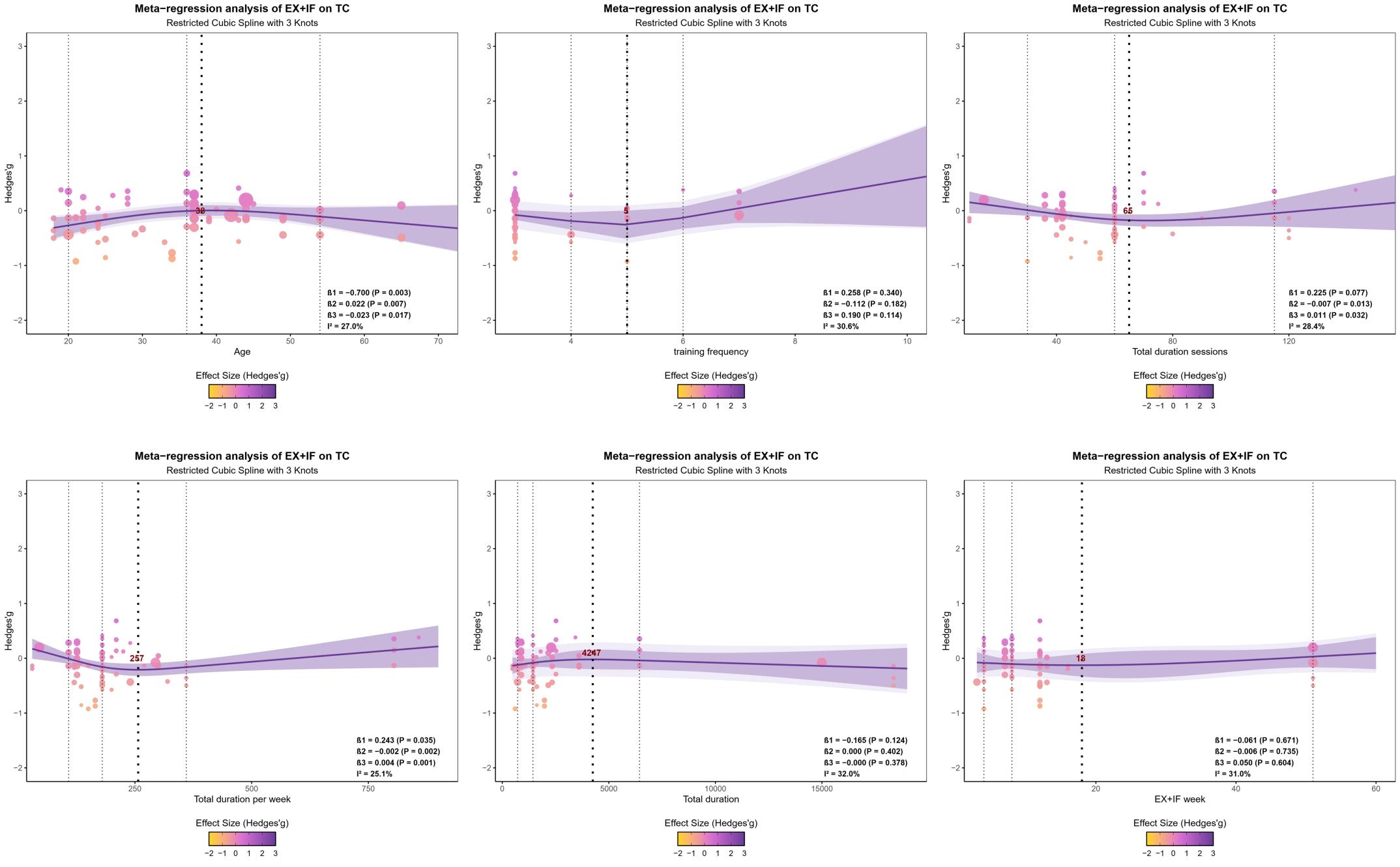


**Supplementary Fig.3H (Meta-regression analysis of EX+IF on TC)** β0 represents the intercept; β1, β2, and β3 represent the slopes; *I*^2^ means heterogeneity; the purple shaded part represents the 95% confidence interval.


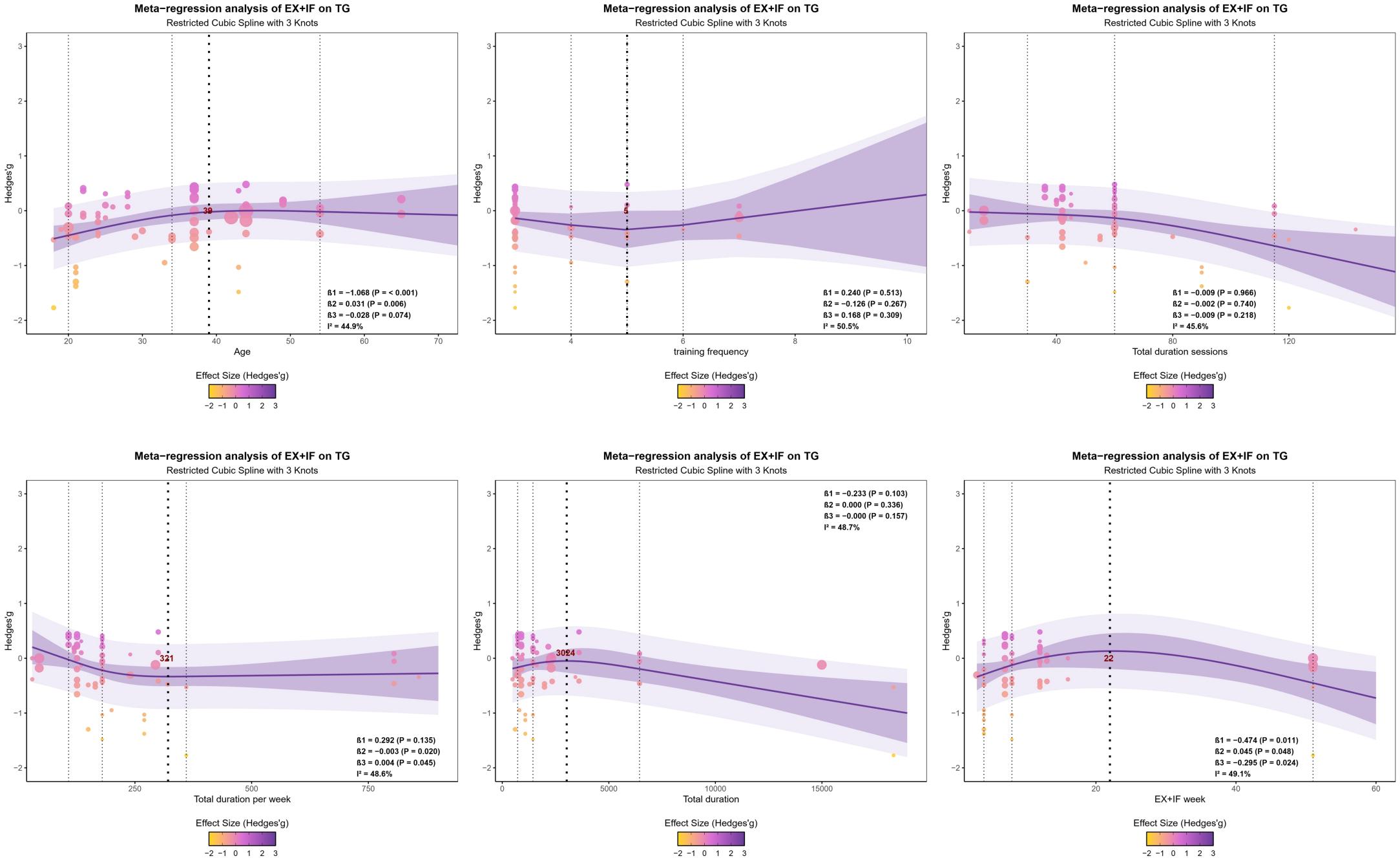


**Supplementary Fig.3I (Meta-regression analysis of EX+IF on TG)** β0 represents the intercept; β1, β2, and β3 represent the slopes; *I*^2^ means heterogeneity; the purple shaded part represents the 95% confidence interval.


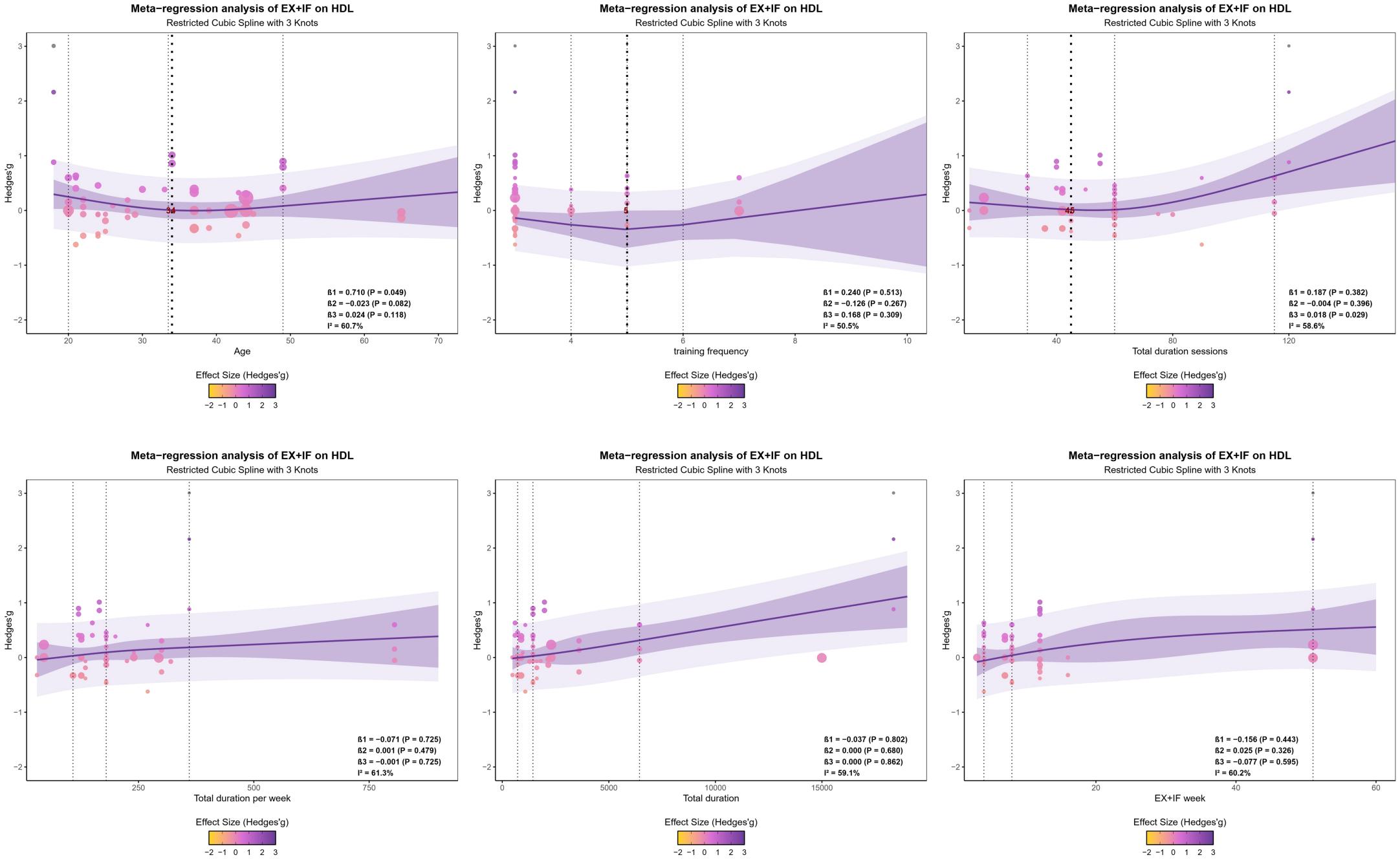


**Supplementary Fig.3J (Meta-regression analysis of EX+IF on HDL)** β0 represents the intercept; β1, β2, and β3 represent the slopes; *I*^2^ means heterogeneity; the purple shaded part represents the 95% confidence interval.


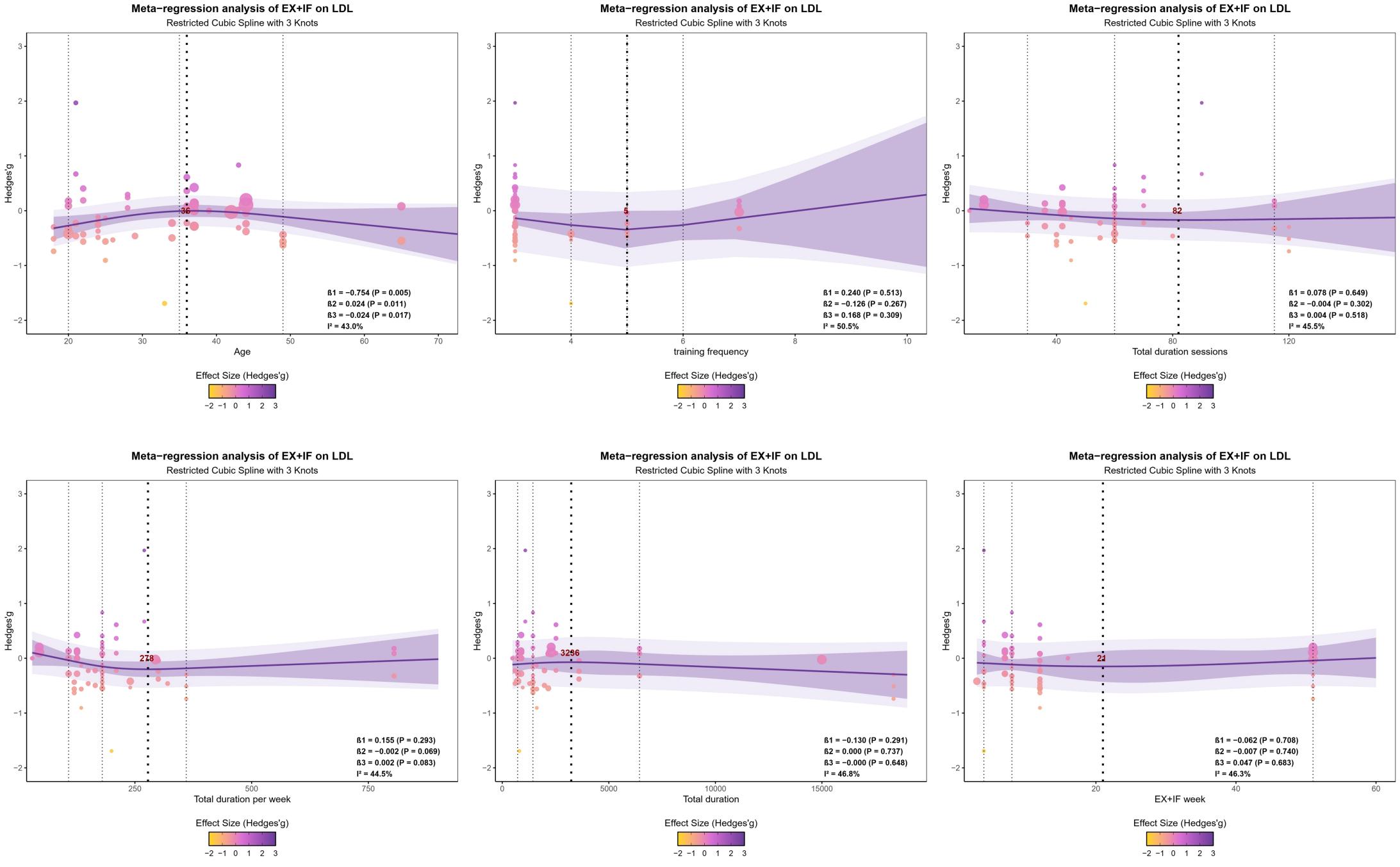


**Supplementary Fig.3K (Meta-regression analysis of EX+IF on LDL)** β0 represents the intercept; β1, β2, and β3 represent the slopes; *I*^2^ means heterogeneity; the purple shaded part represents the 95% confidence interval.


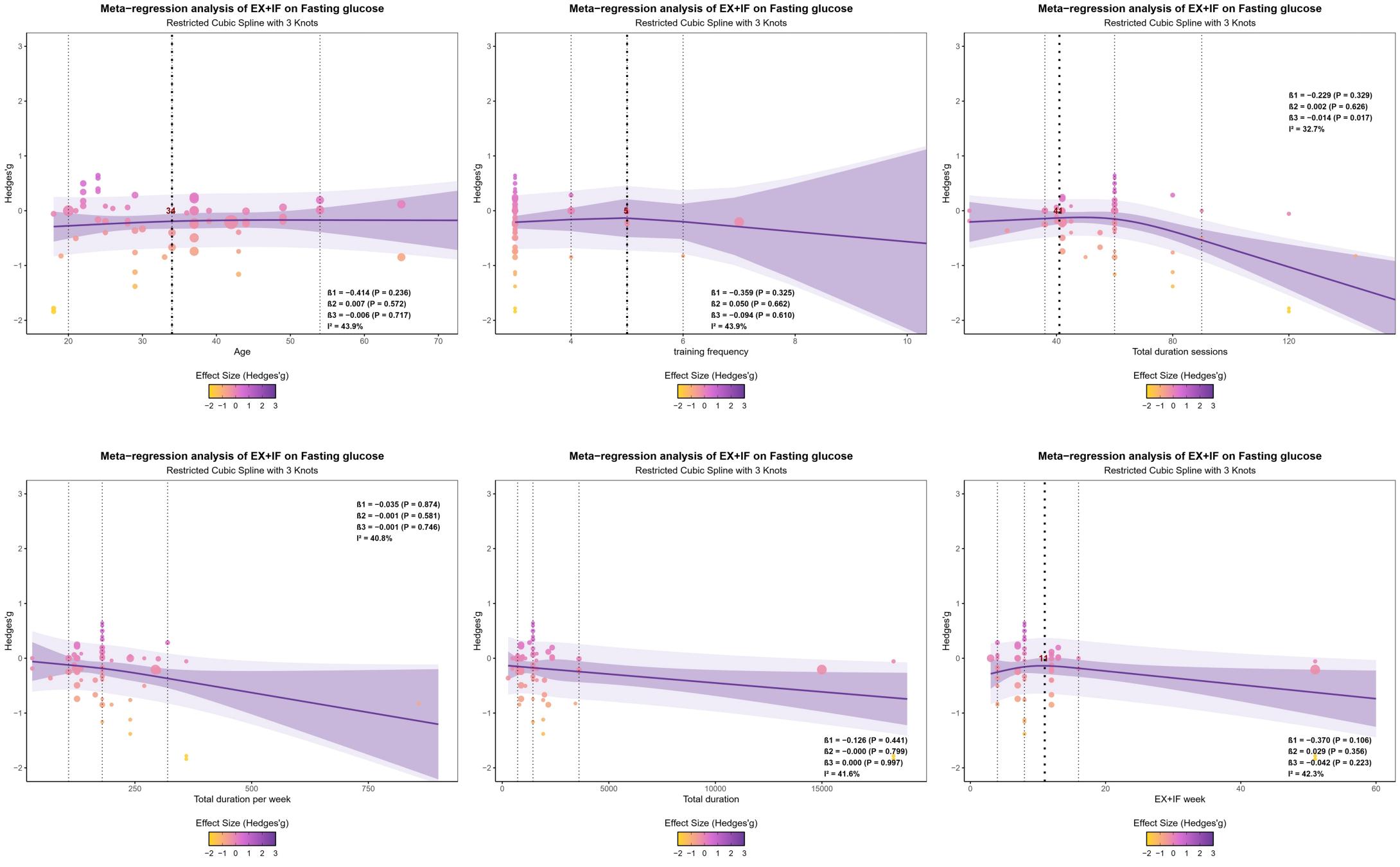


**Supplementary Fig.3L (Meta-regression analysis of EX+IF on fasting glucose)** β0 represents the intercept; β1, β2, and β3 represent the slopes; *I*^2^ means heterogeneity; the purple shaded part represents the 95% confidence interval.


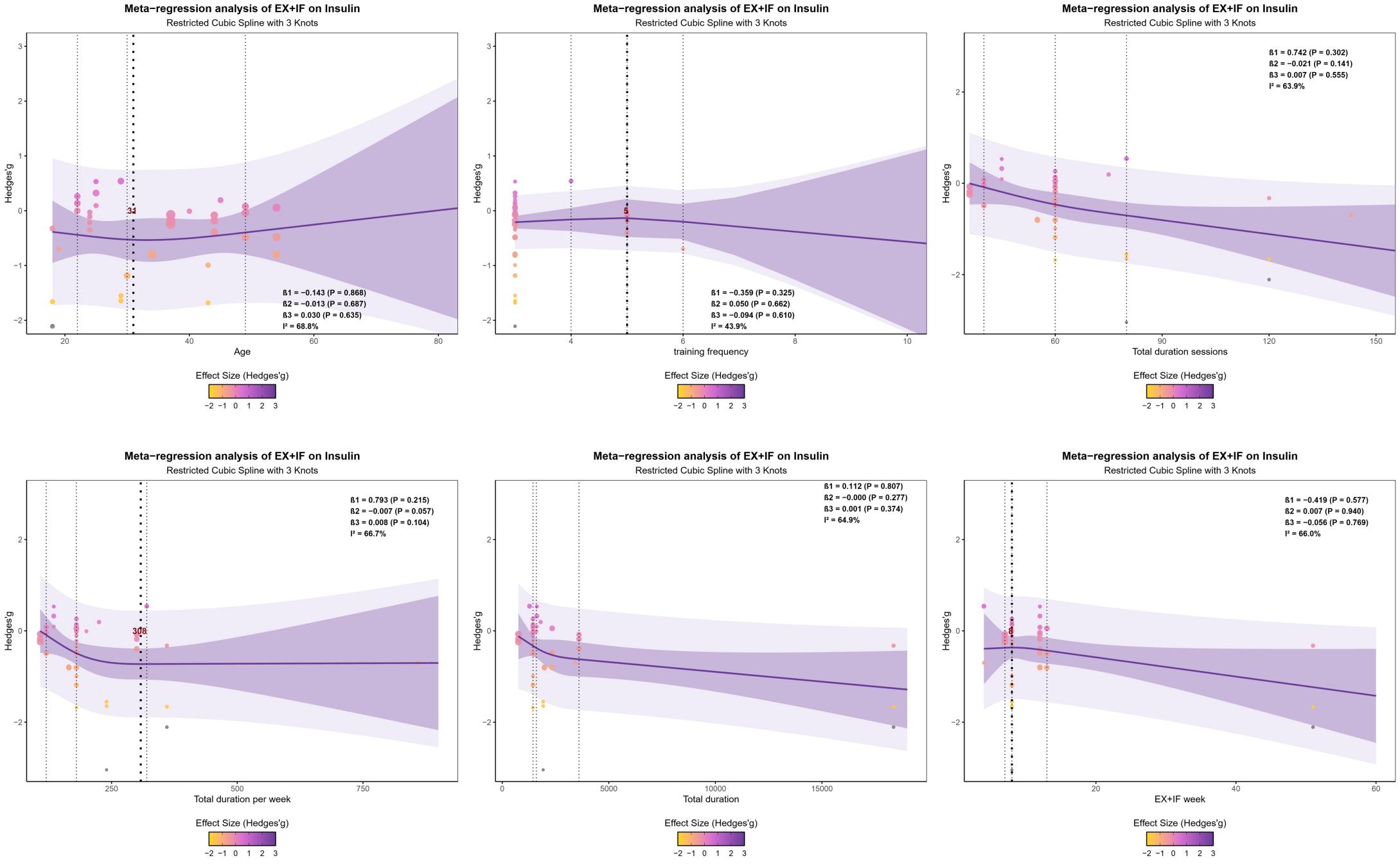


**Supplementary Fig.3M (Meta-regression analysis of EX+IF on insulin)** β0 represents the intercept; β1, β2, and β3 represent the slopes; *I*^2^ means heterogeneity; the purple shaded part represents the 95% confidence interval.


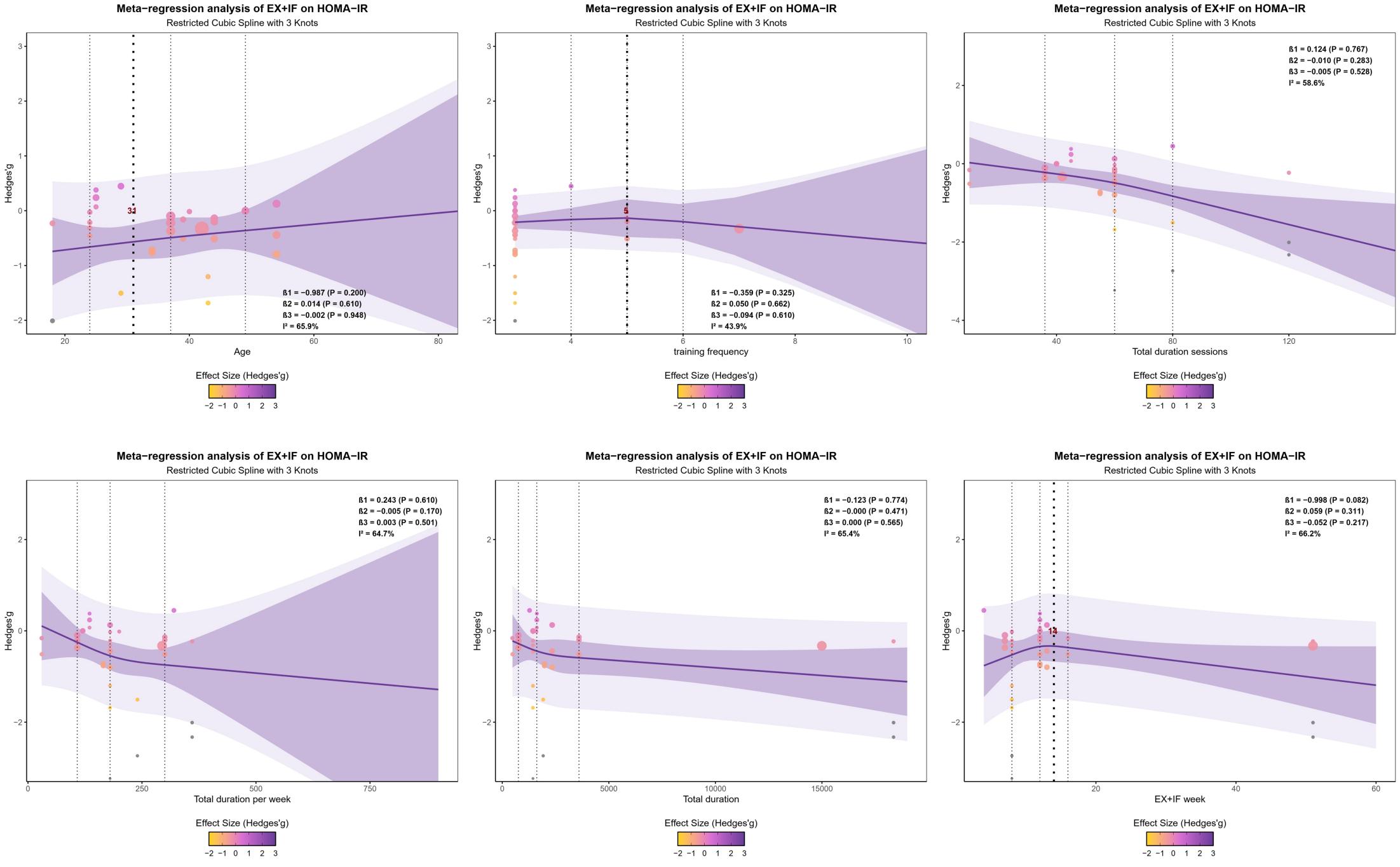


**Supplementary Fig.3N (Meta-regression analysis of EX+IF on HOMA-IR)** β0 represents the intercept; β1, β2, and β3 represent the slopes; *I*^2^ means heterogeneity; the purple shaded part represents the 95% confidence interval.


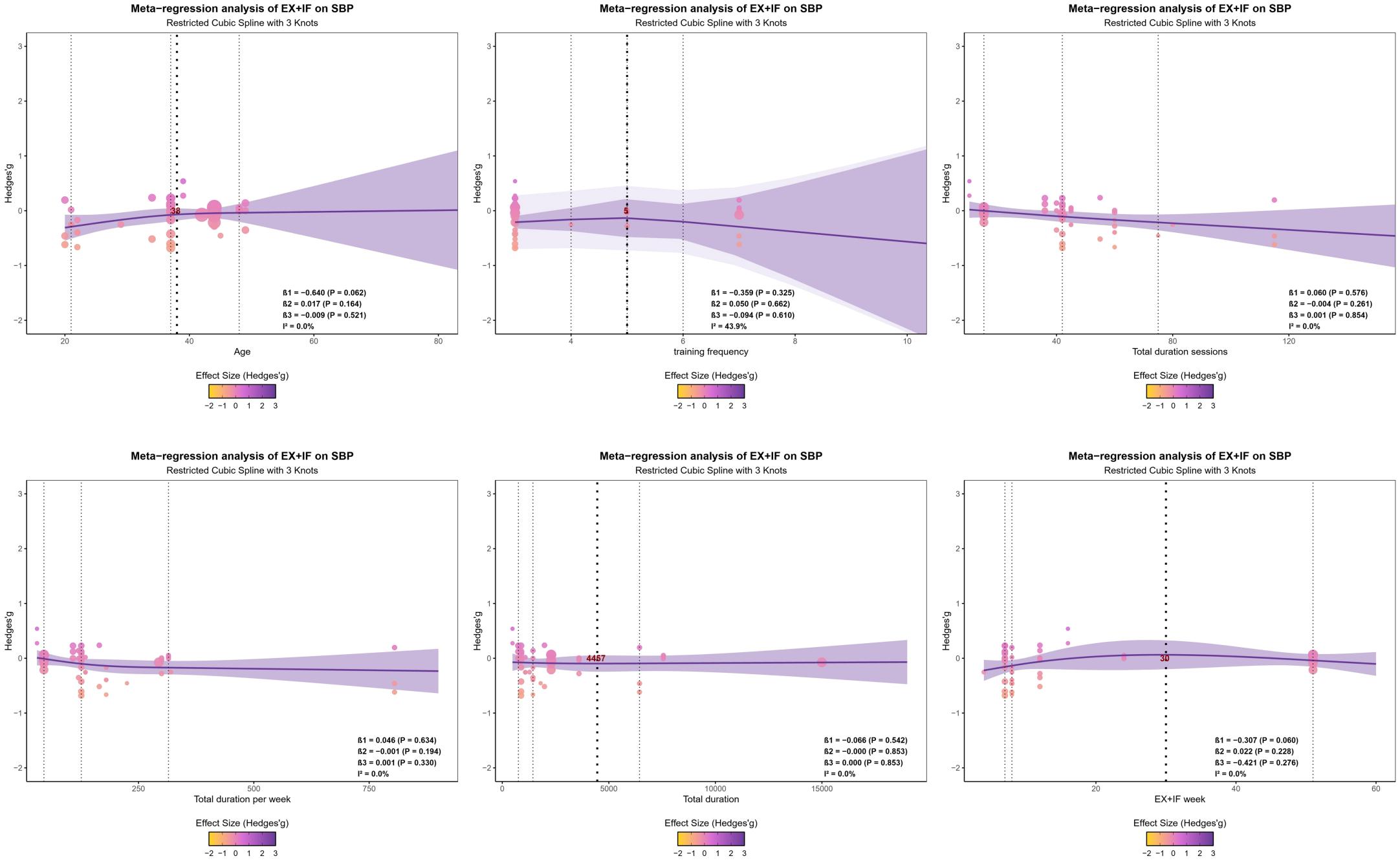


**Supplementary Fig.3O (Meta-regression analysis of EX+IF on SBP)** β0 represents the intercept; β1, β2, and β3 represent the slopes; *I*^2^ means heterogeneity; the purple shaded part represents the 95% confidence interval.


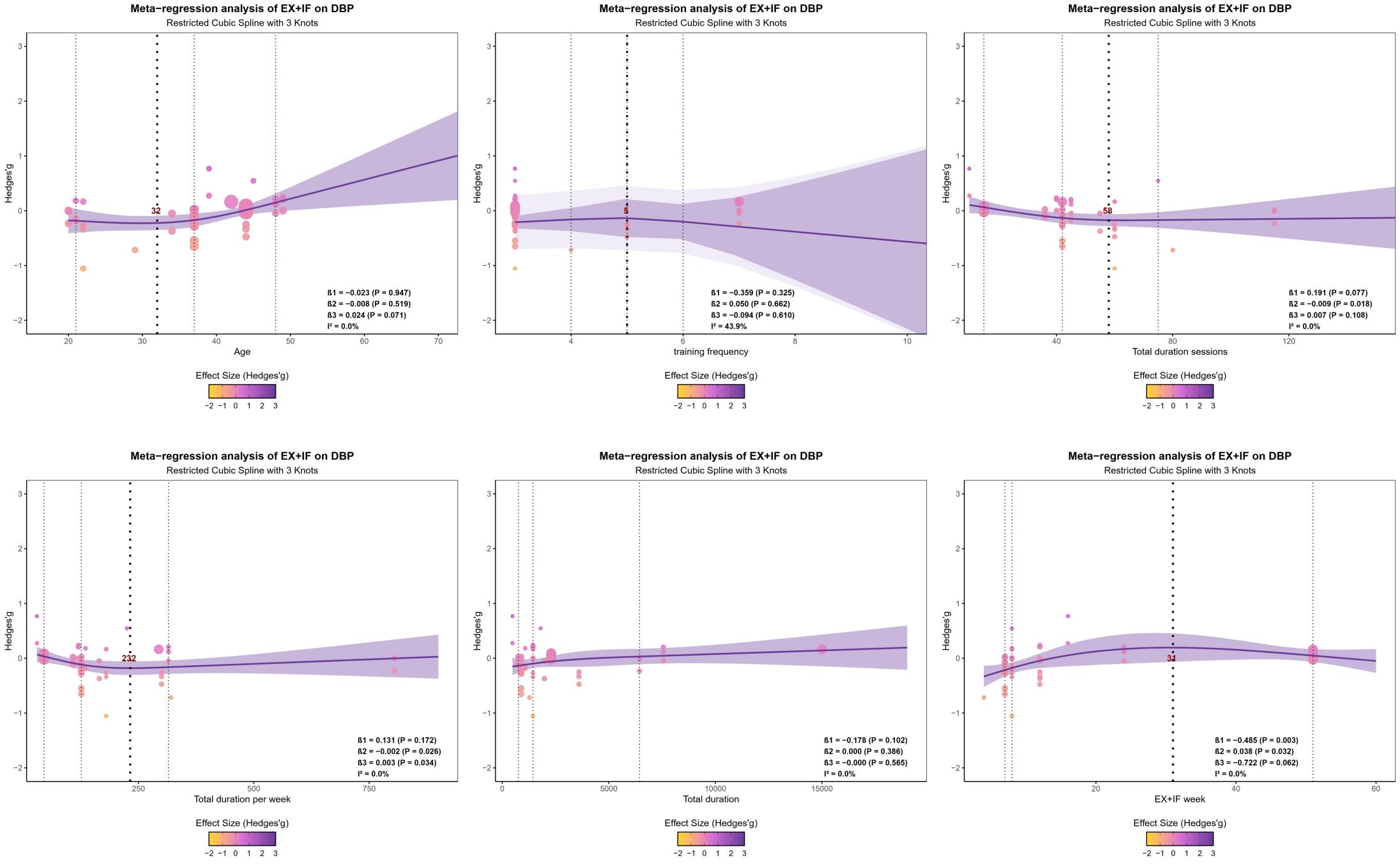


**Supplementary Fig.3P (Meta-regression analysis of EX+IF on DBP)** β0 represents the intercept; β1, β2, and β3 represent the slopes; *I*^2^ means heterogeneity; the purple shaded part represents the 95% confidence interval.


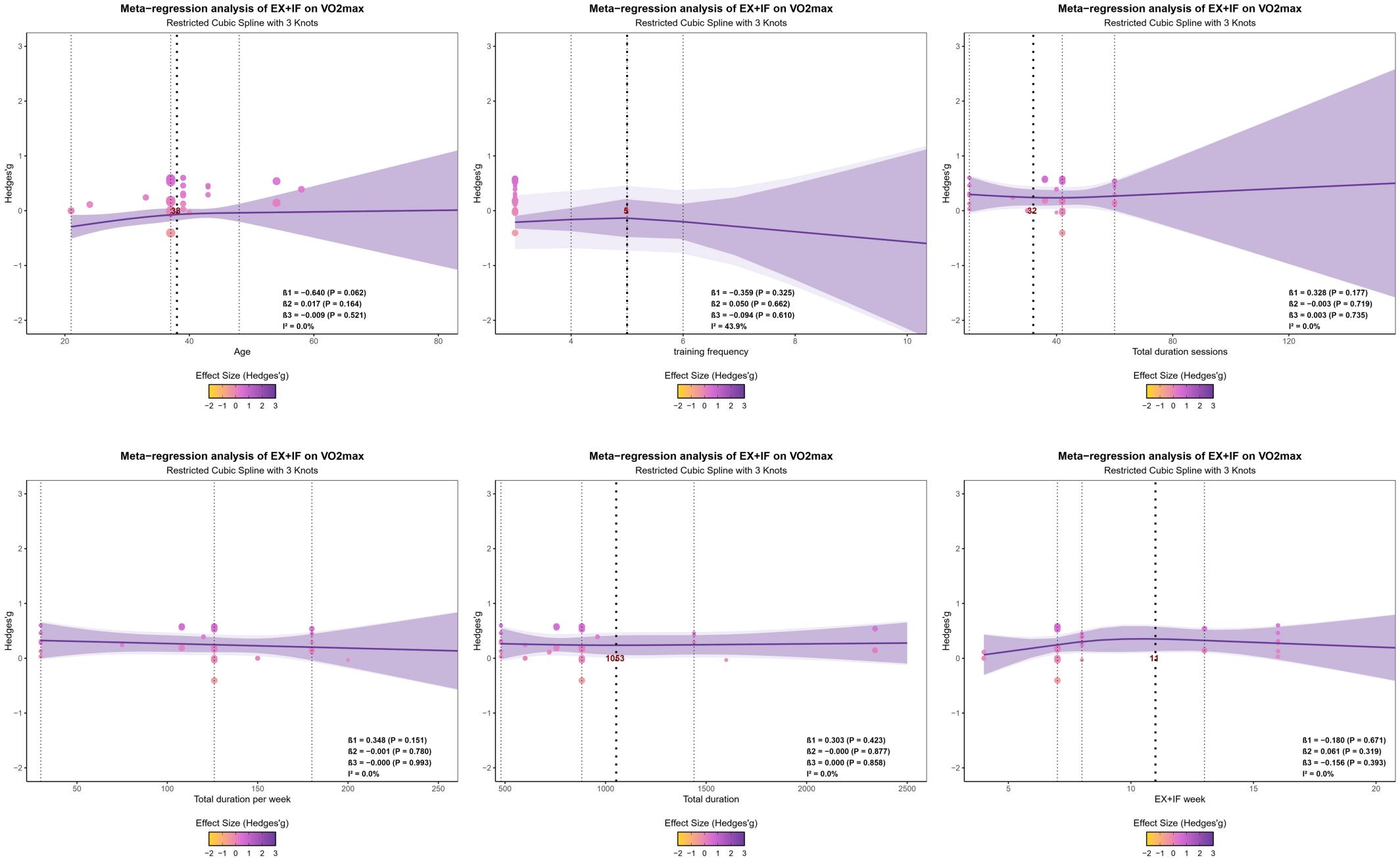


**Supplementary Fig.3Q (Meta-regression analysis of EX+IF on VO_2max_)** β0 represents the intercept; β1, β2, and β3 represent the slopes; *I*^2^ means heterogeneity; the purple shaded part represents the 95% confidence interval.


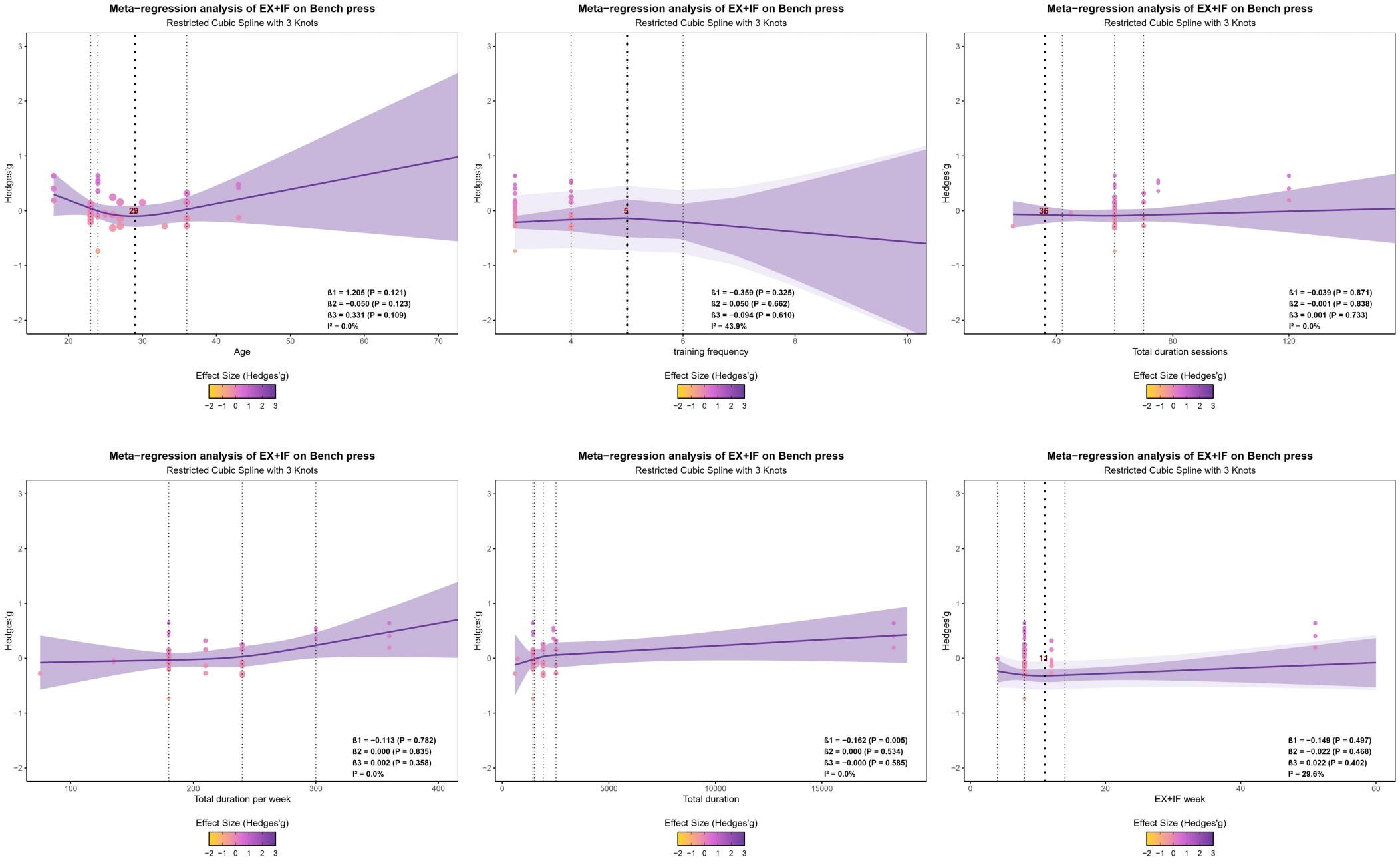


**Supplementary Fig.3R (Meta-regression analysis of EX+IF on bench press)** β0 represents the intercept; β1, β2, and β3 represent the slopes; *I*^2^ means heterogeneity; the purple shaded part represents the 95% confidence interval.


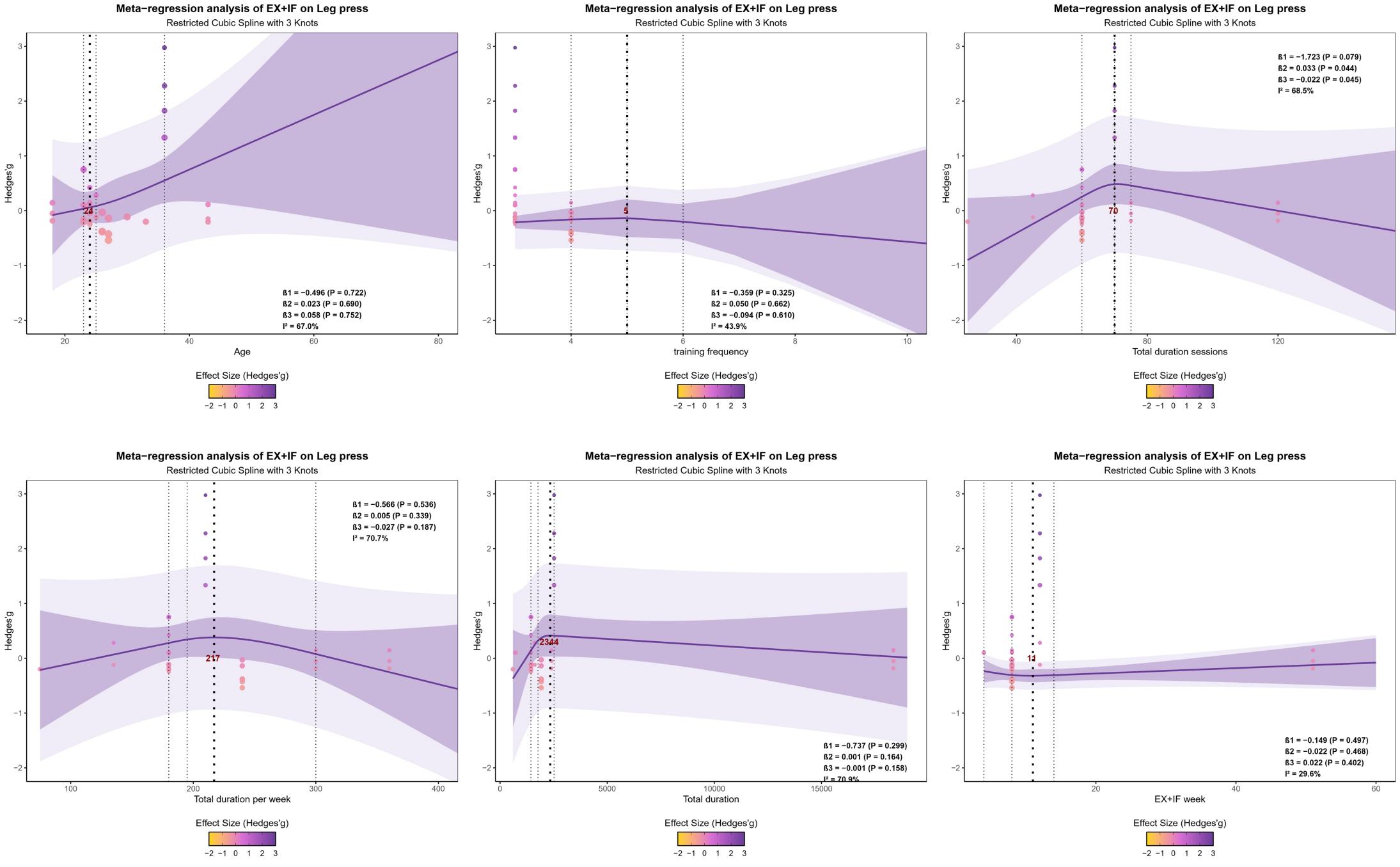


**Supplementary Fig.3S (Meta-regression analysis of EX+IF on leg press)** β0 represents the intercept; β1, β2, and β3 represent the slopes; *I*^2^ means heterogeneity; the purple shaded part represents the 95% confidence interval.


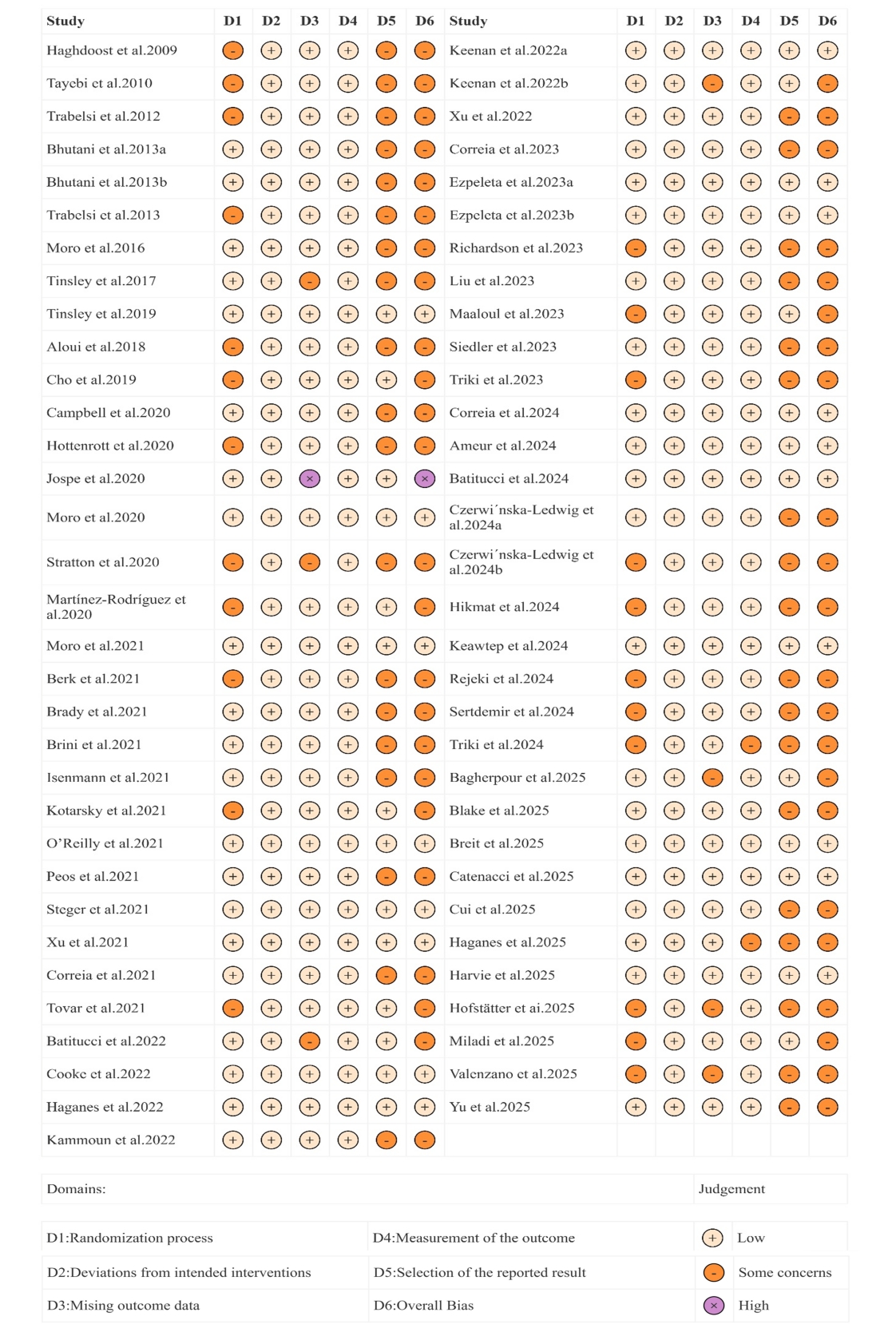


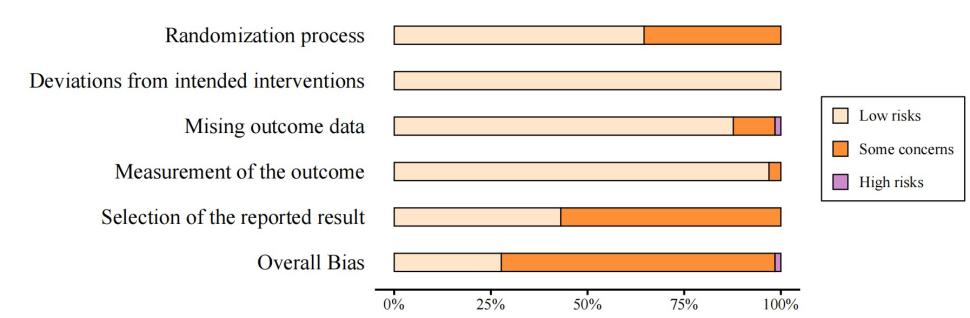


**Supplementary Fig.4 (Risk of bias for the included studies)**


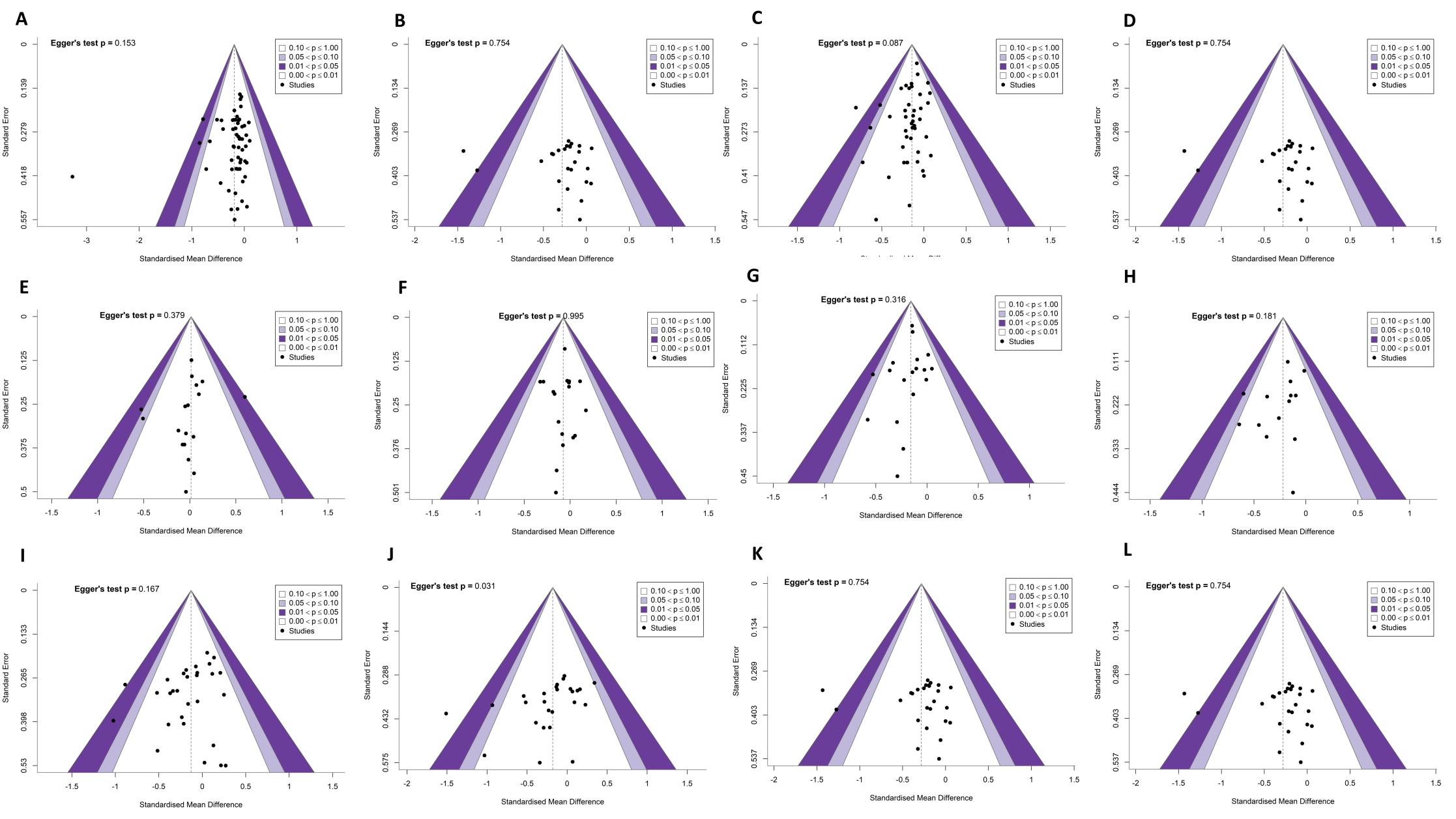


**Supplementary Fig.5A (Funnel plot of included studies)** From A to AC, they are they are body mass, BMI, body fat%, fat mass, fat free mass, lean body mass, waist circumference, visceral adipose tissue, total cholesterol, triglyceride, high density lipoprotein, low density lipoprotein, fasting glucose, insulin, HOMA-IR, HAb1c, systolic blood pressure, diastolic blood pressure, heart rate, VO_2max_, adiponectin, leptin, CRP, IL-6, TNF-α, handgrip, bench press, leg press, jump height.


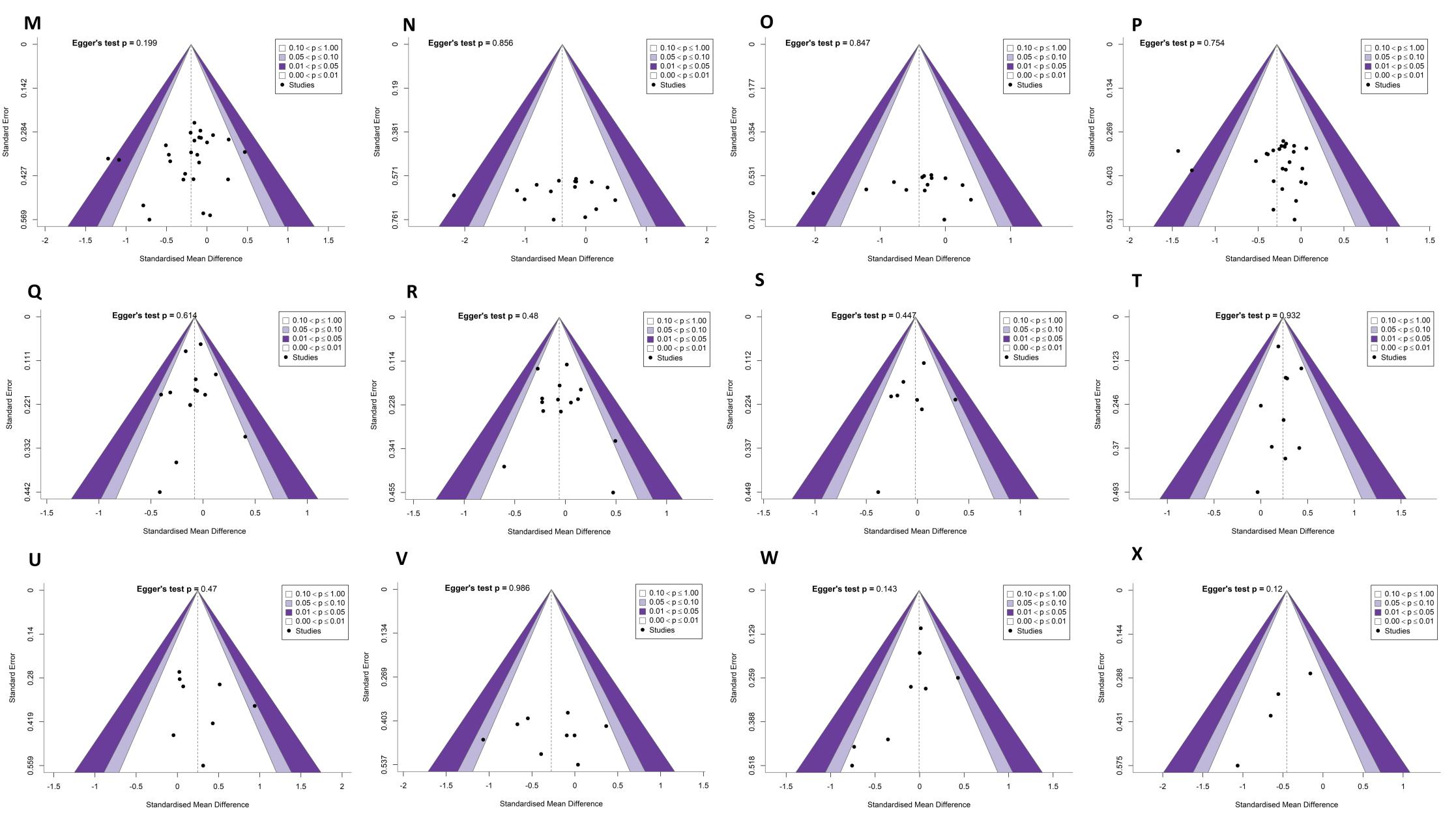


**Supplementary Fig.5B (Funnel plot of included studies)**


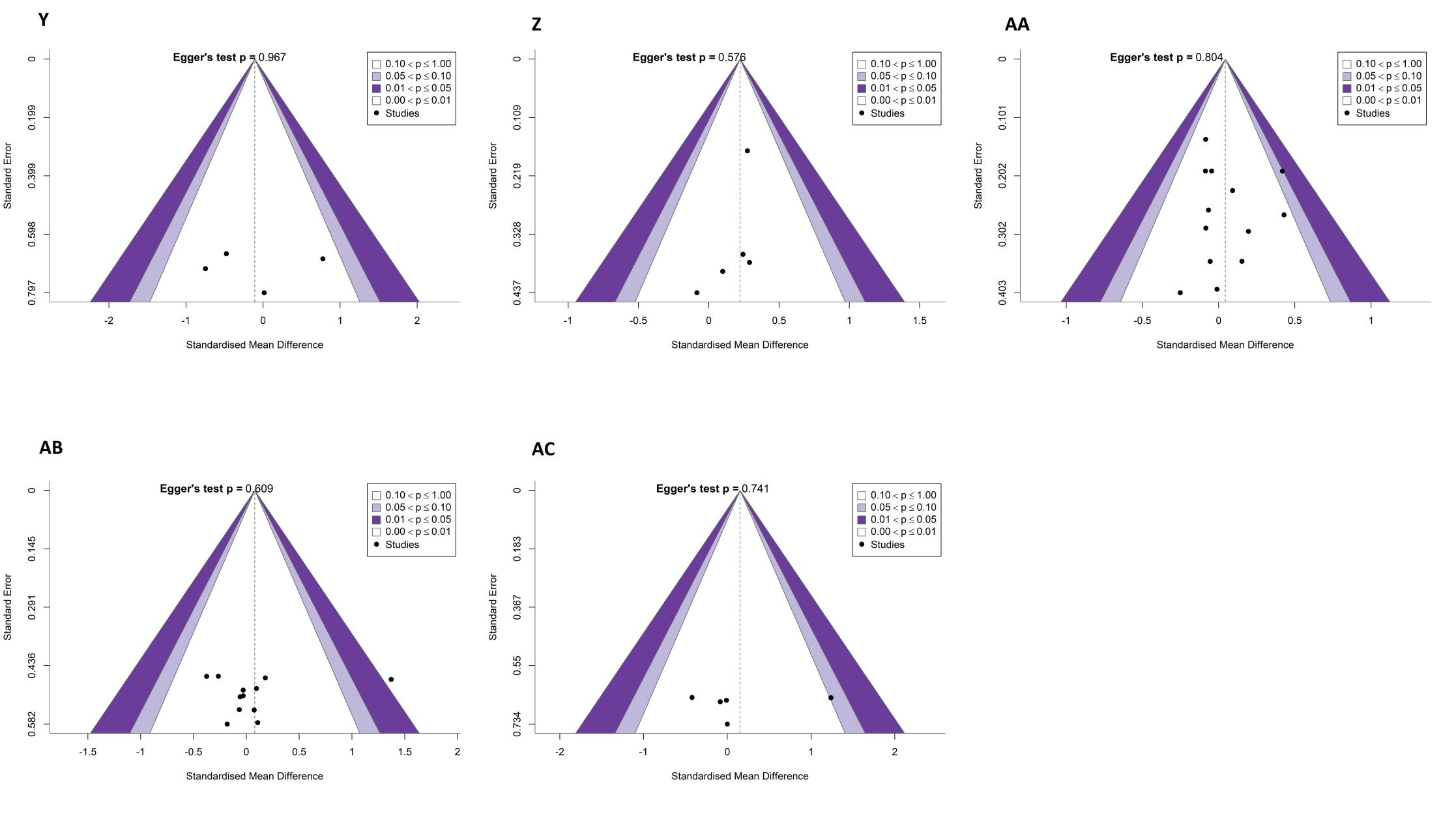


**Supplementary Fig.5C (Funnel plot of included studies)**


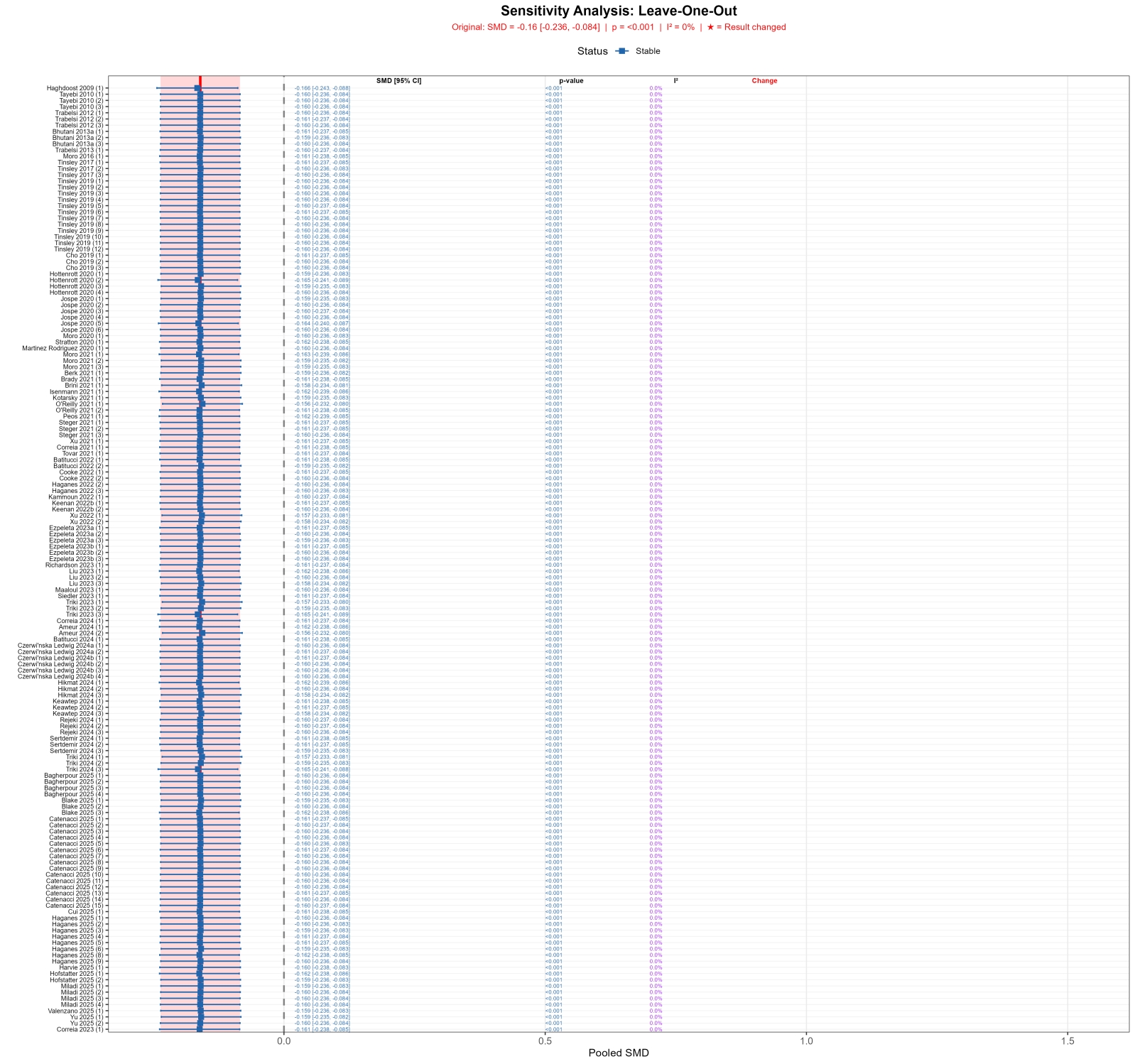


**Body mass**

**Supplementary Fig.6A (A sensitivity analysis based on leave-one-out)**


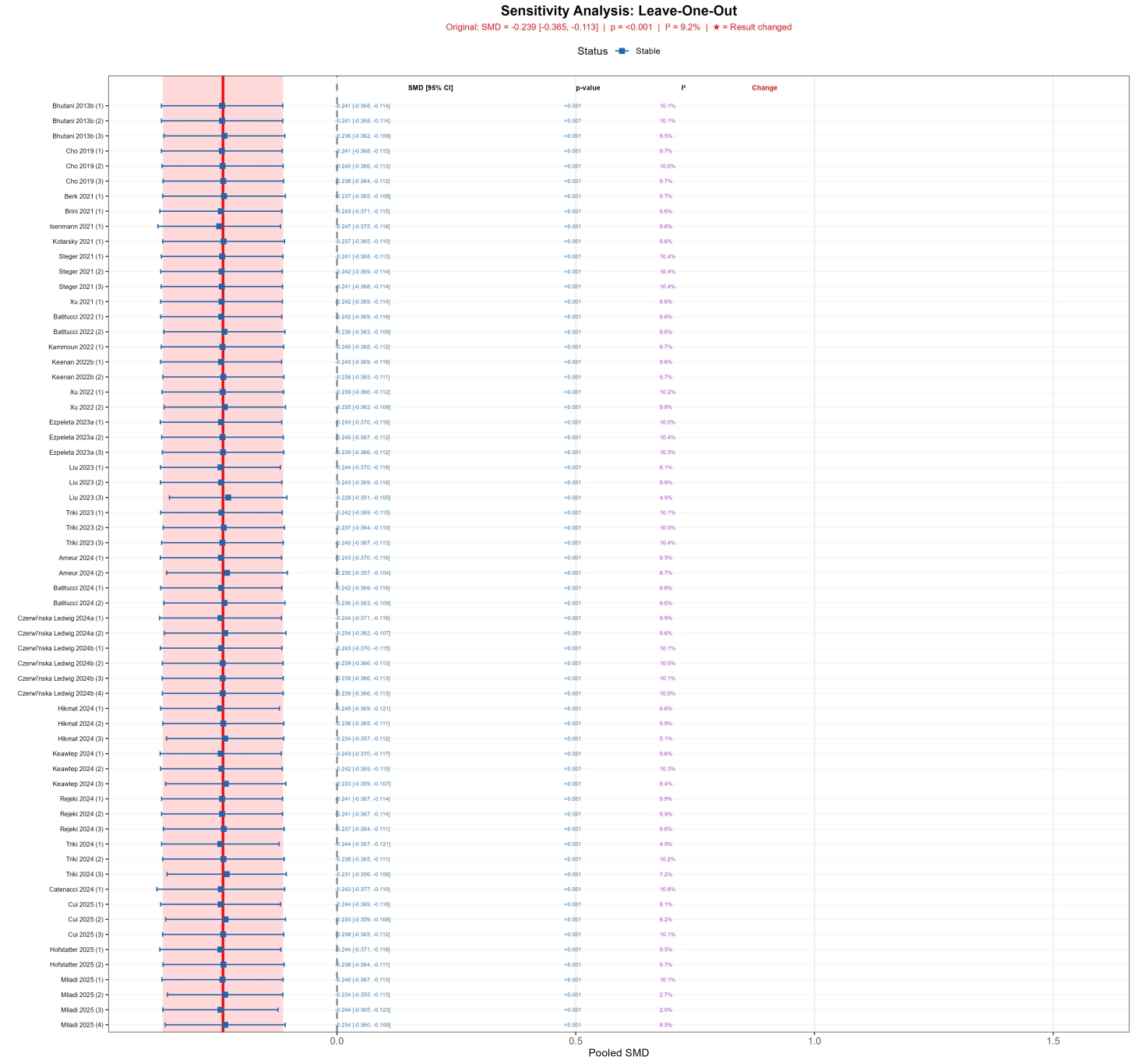


**BMI**

**Supplementary Fig.6B (A sensitivity analysis based on leave-one-out)**


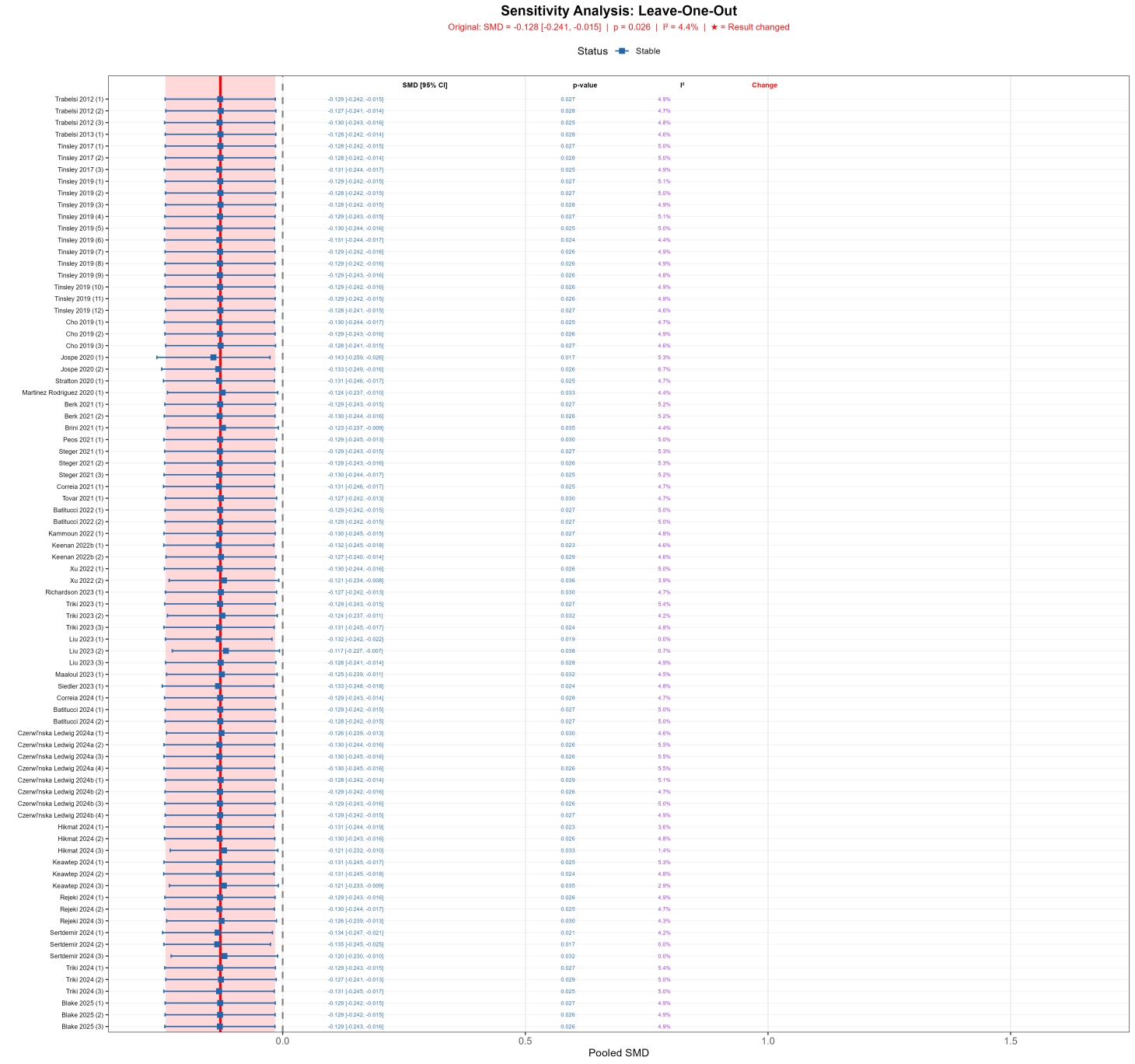


**Body fat%**

**Supplementary Fig.6C (A sensitivity analysis based on leave-one-out)**


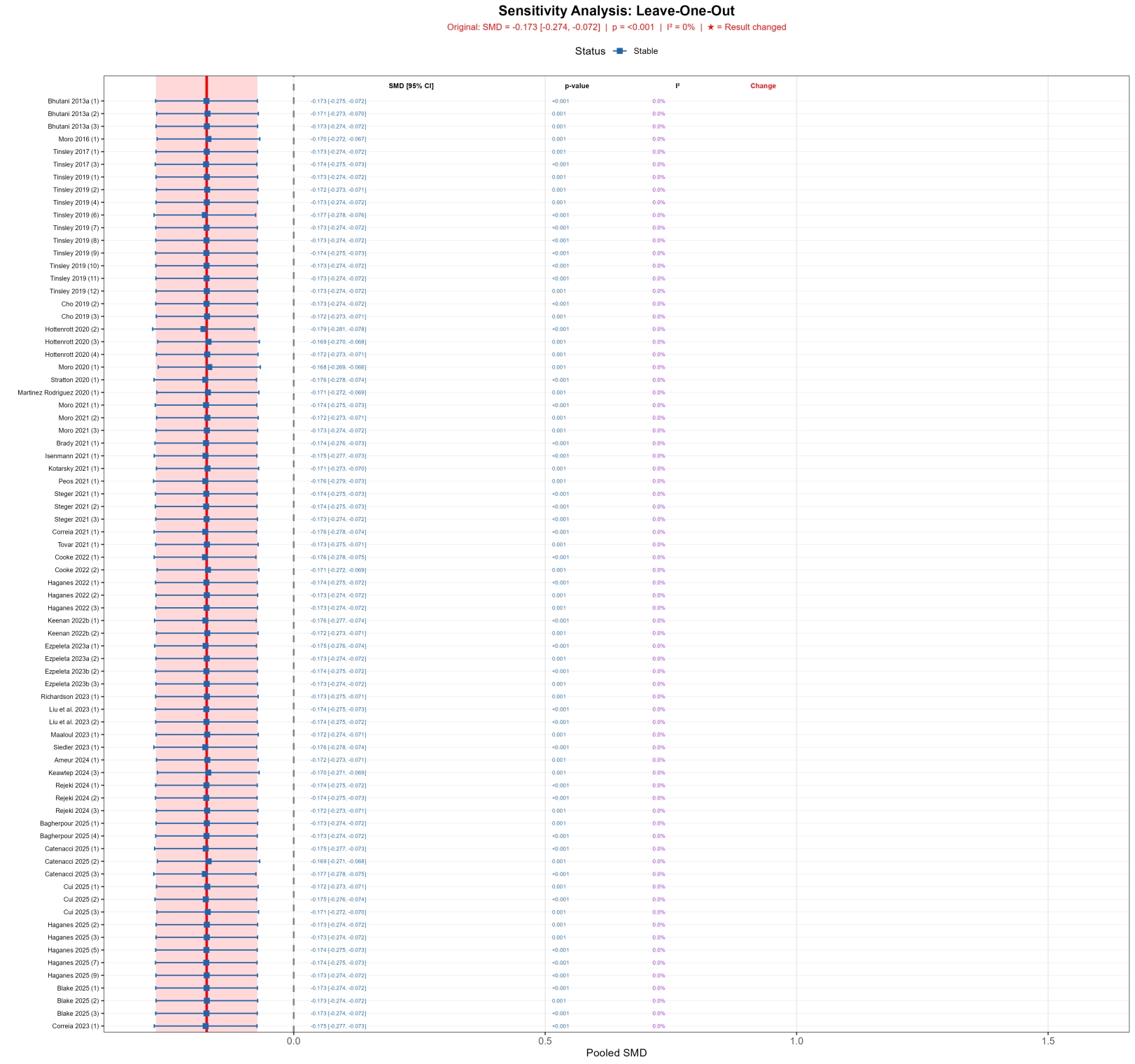


**Fat mass**

**Supplementary Fig.6D (A sensitivity analysis based on leave-one-out)**


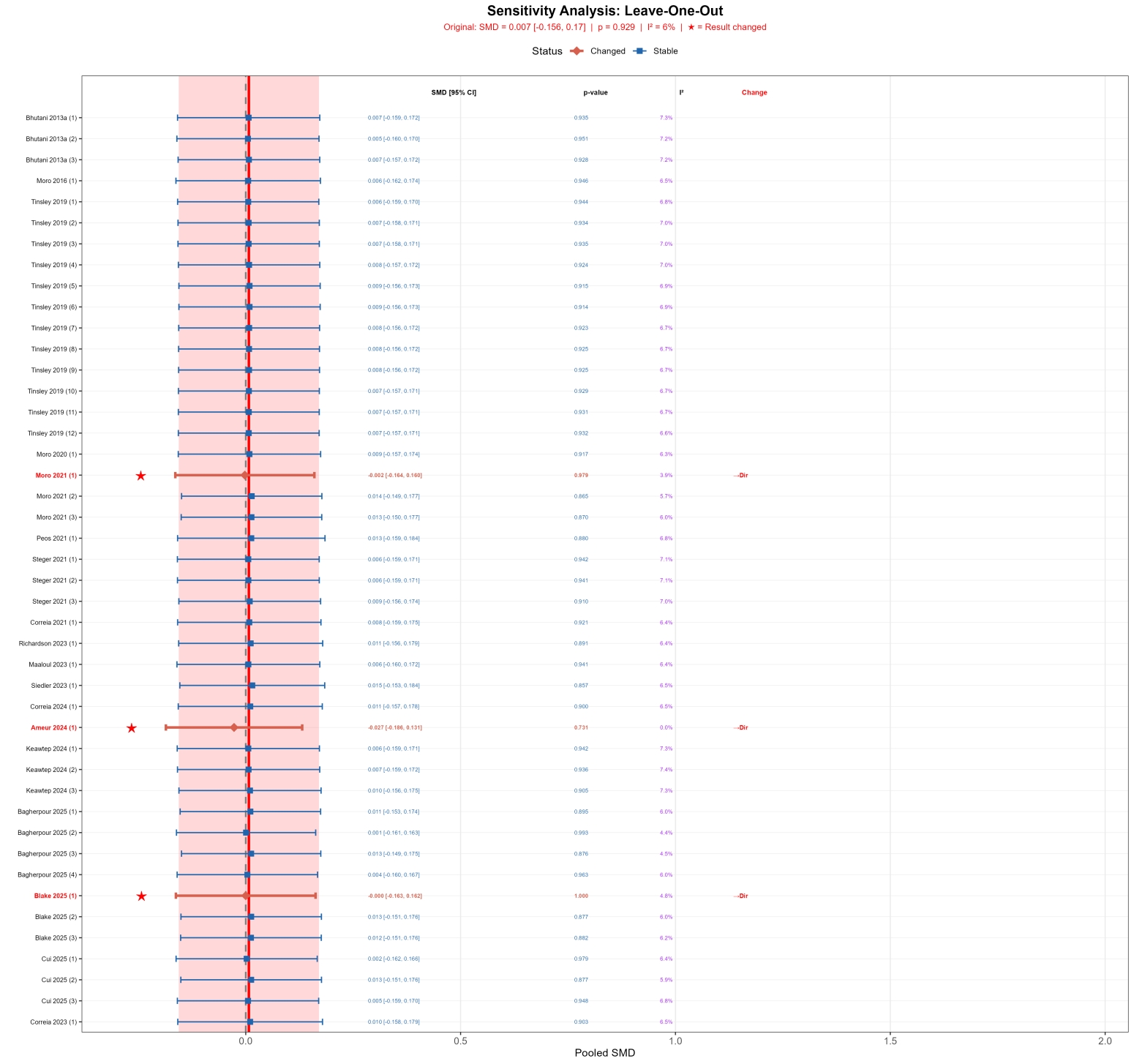


**Fat free mass**

**Supplementary Fig.6E (A sensitivity analysis based on leave-one-out)**


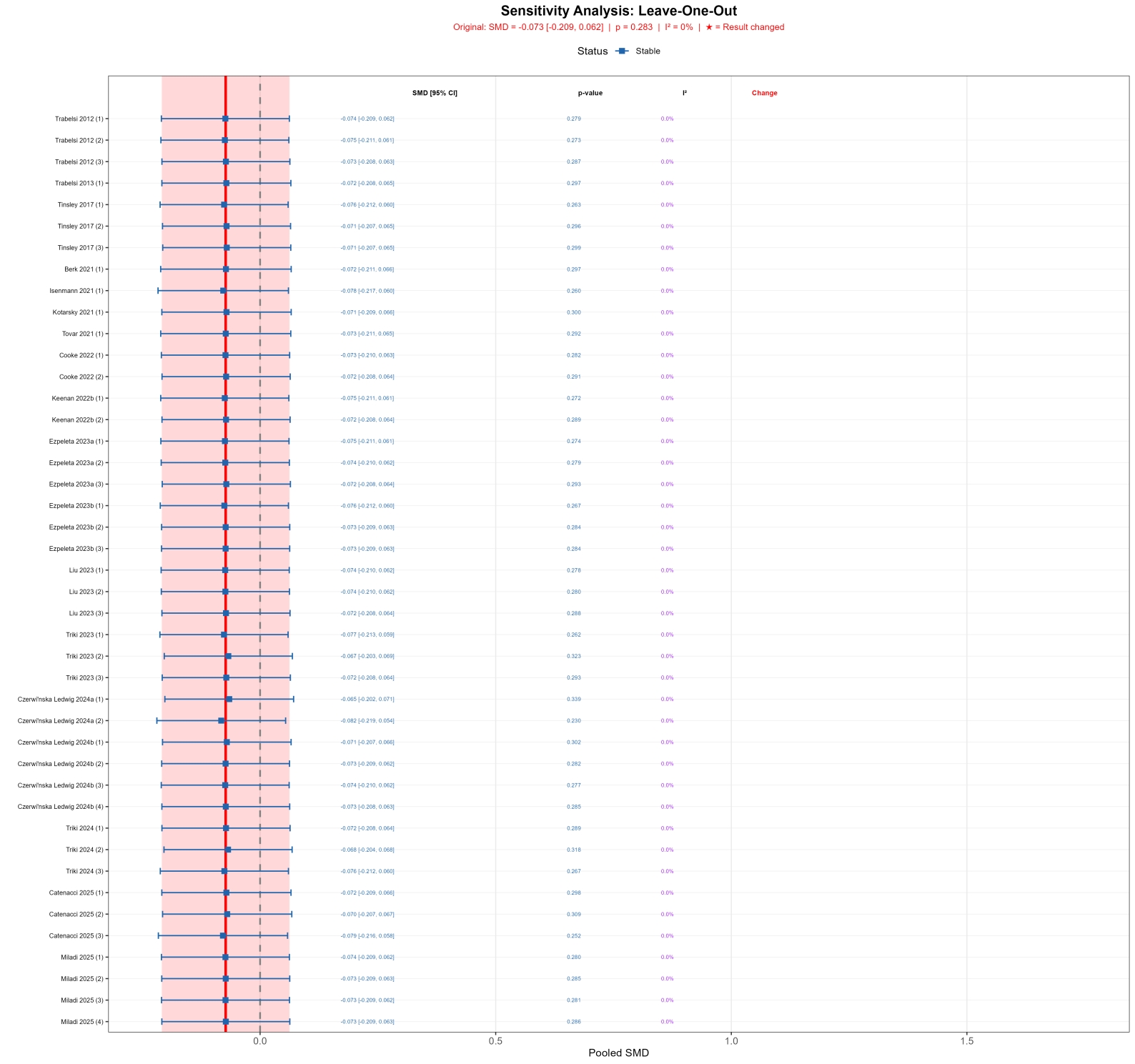


**Lean body mass**

**Supplementary Fig.6F (A sensitivity analysis based on leave-one-out)**


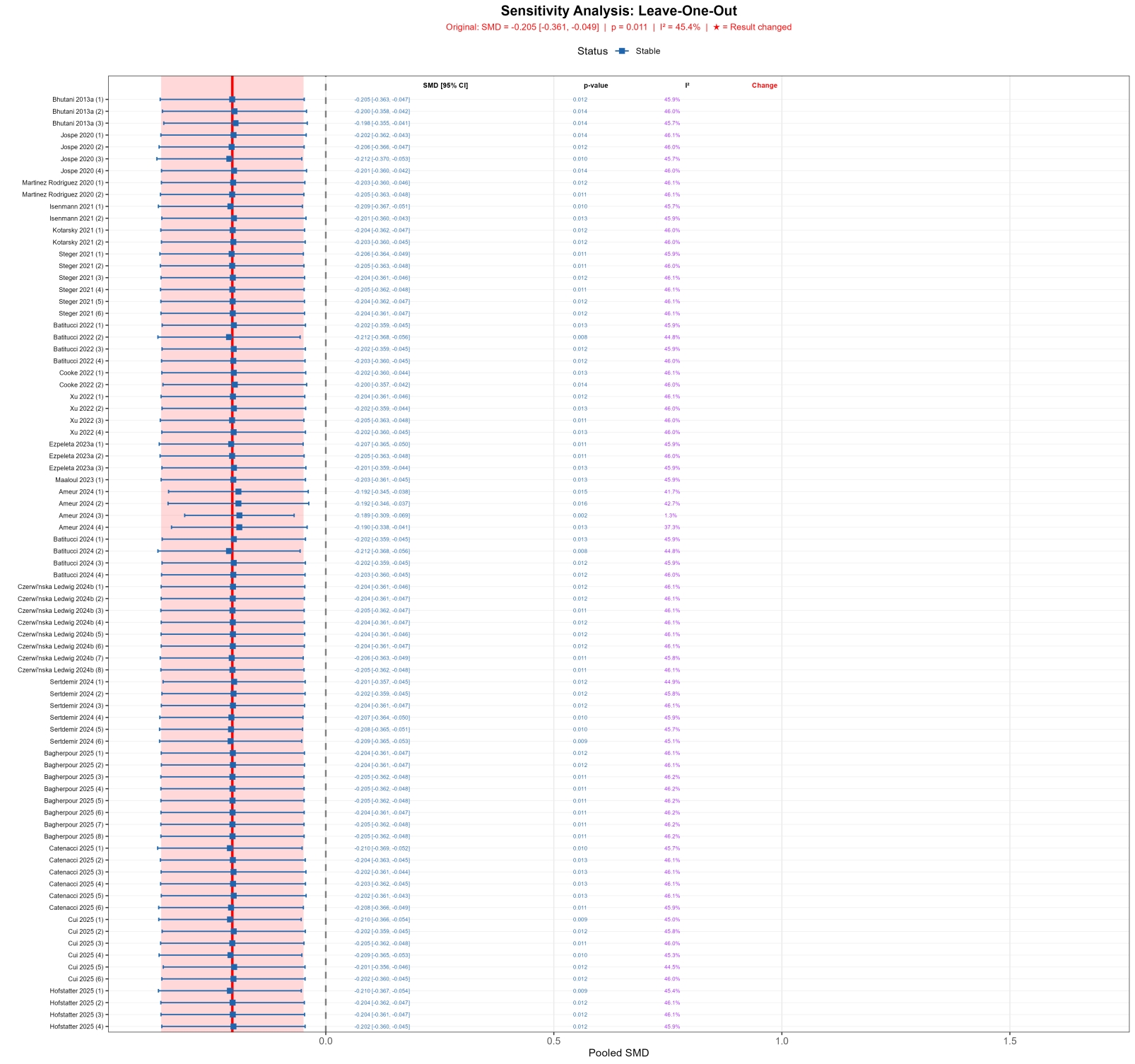


**Waist circumference**

**Supplementary Fig.6G (A sensitivity analysis based on leave-one-out)**


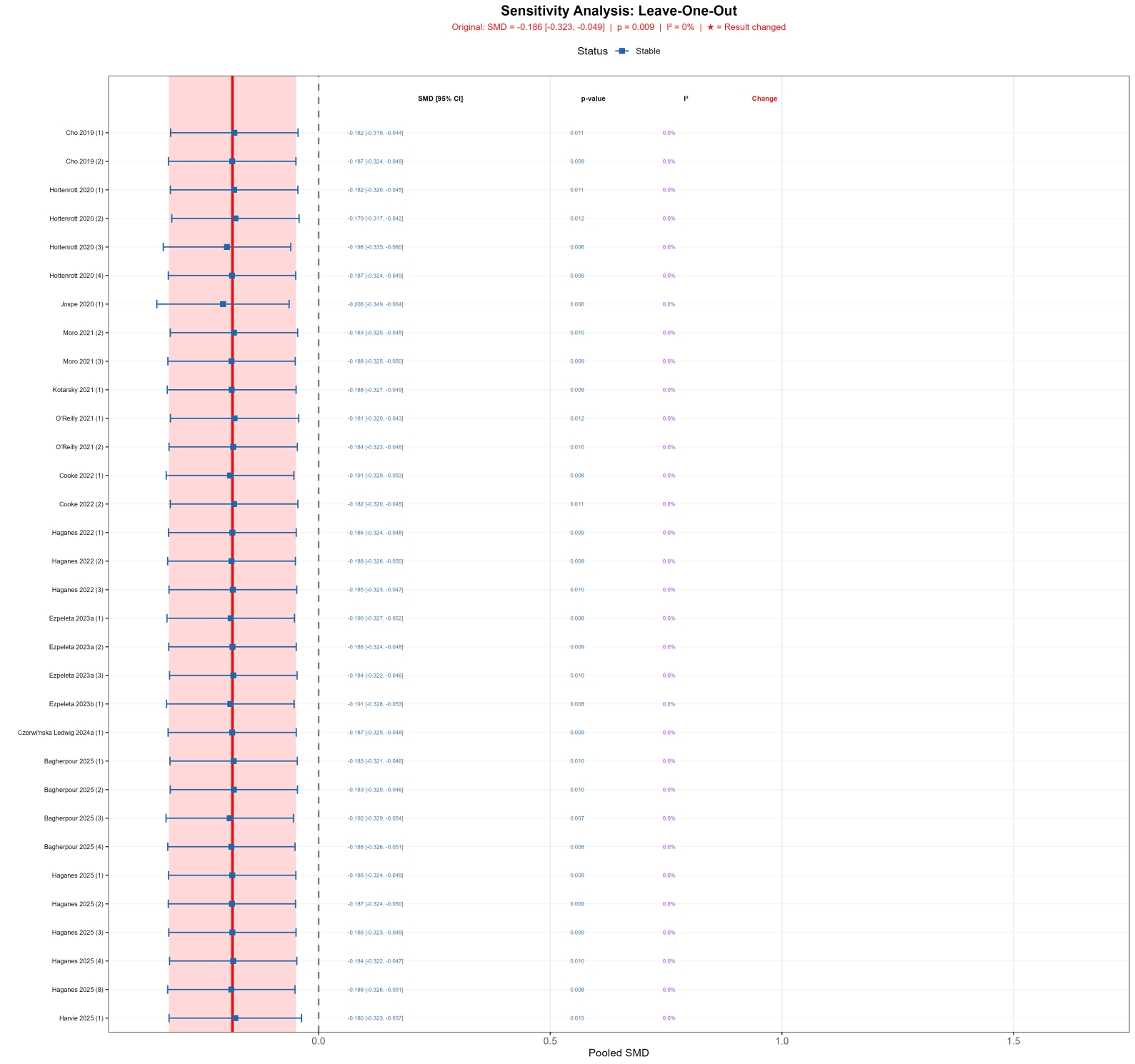


**Visceral adipose tissue**

**Supplementary Fig.6H (A sensitivity analysis based on leave-one-out)**


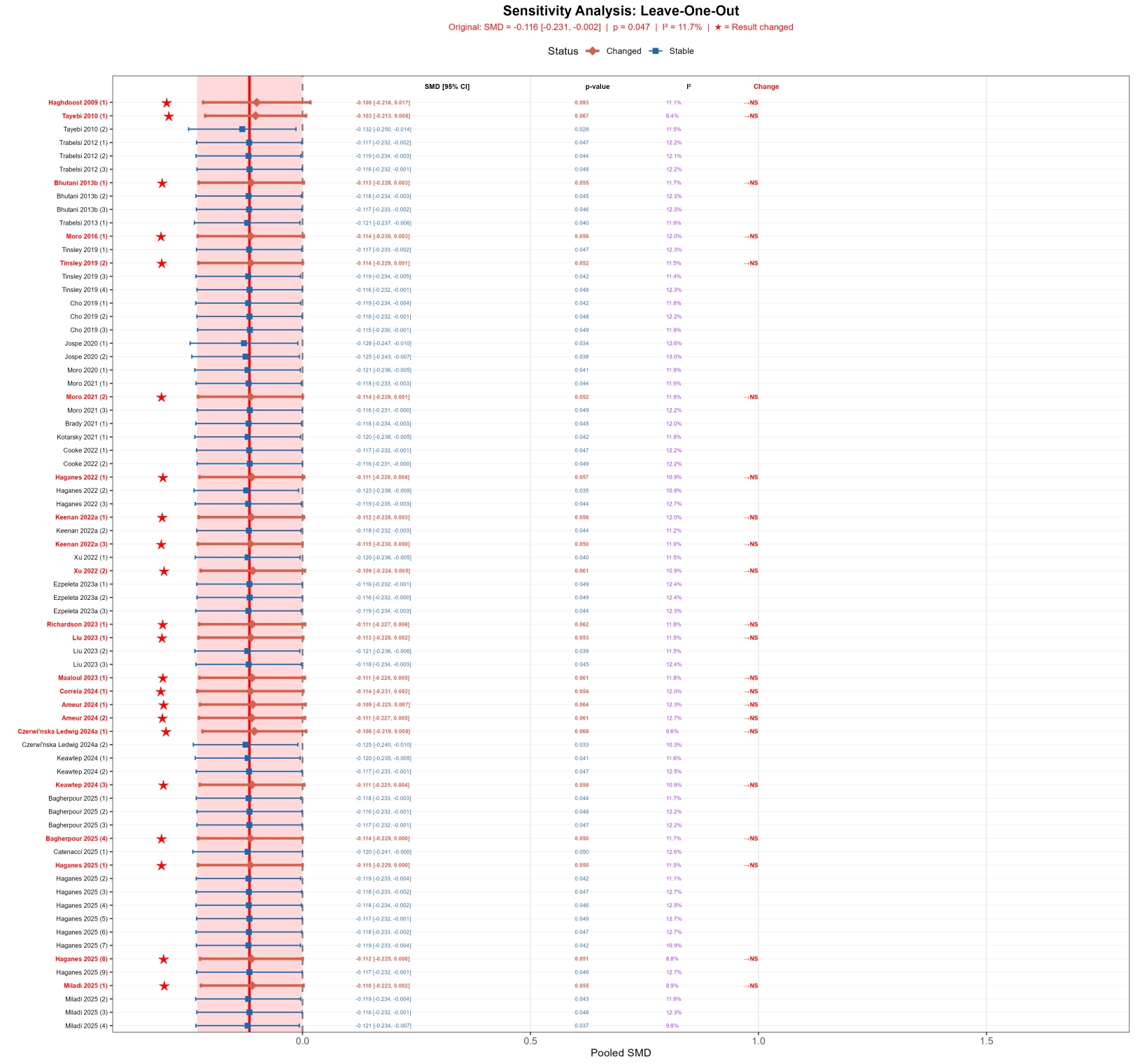


**Total cholesterol**

**Supplementary Fig.6I (A sensitivity analysis based on leave-one-out)**


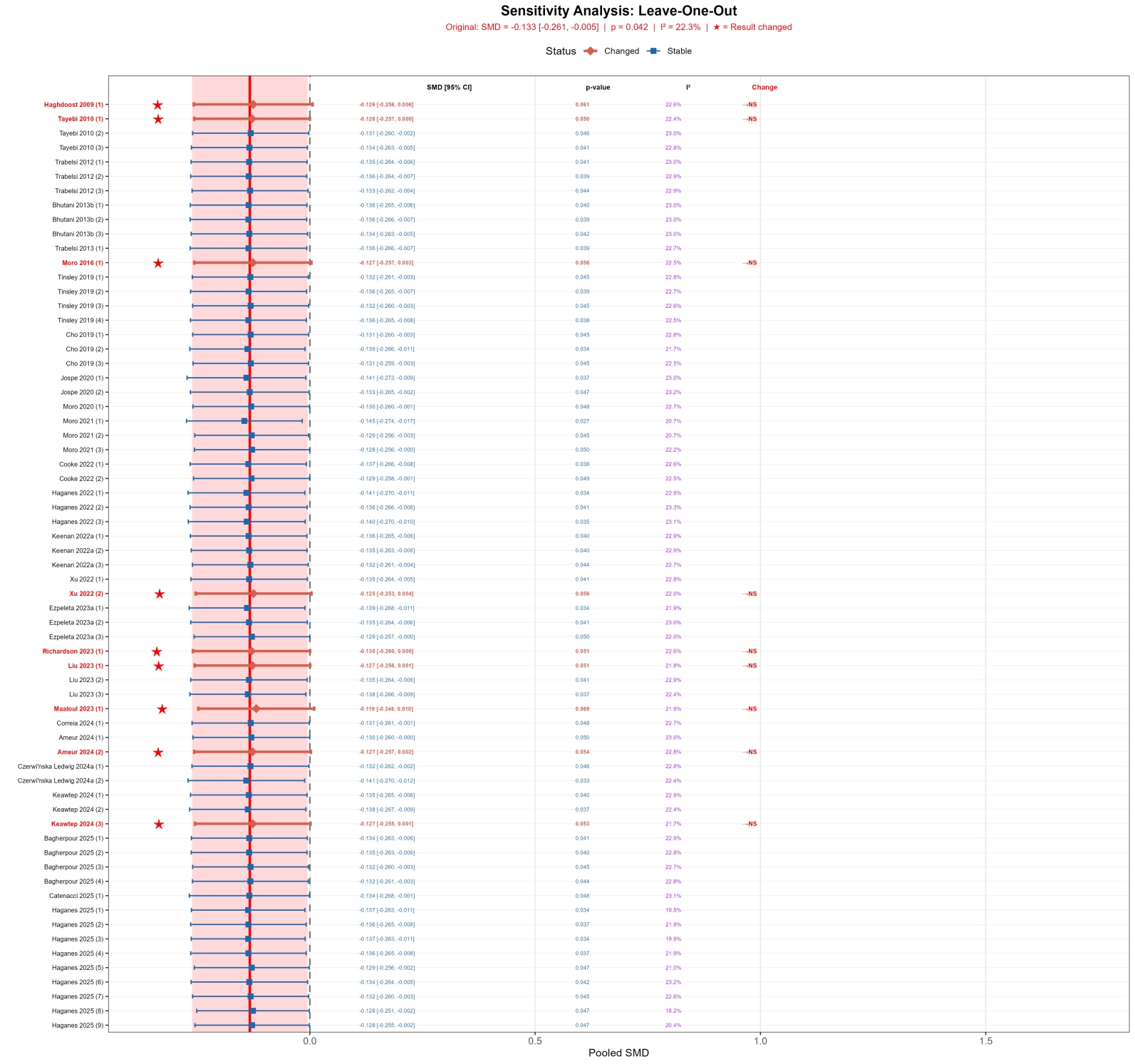


**Triglyceride**

**Supplementary Fig.6J (A sensitivity analysis based on leave-one-out)**


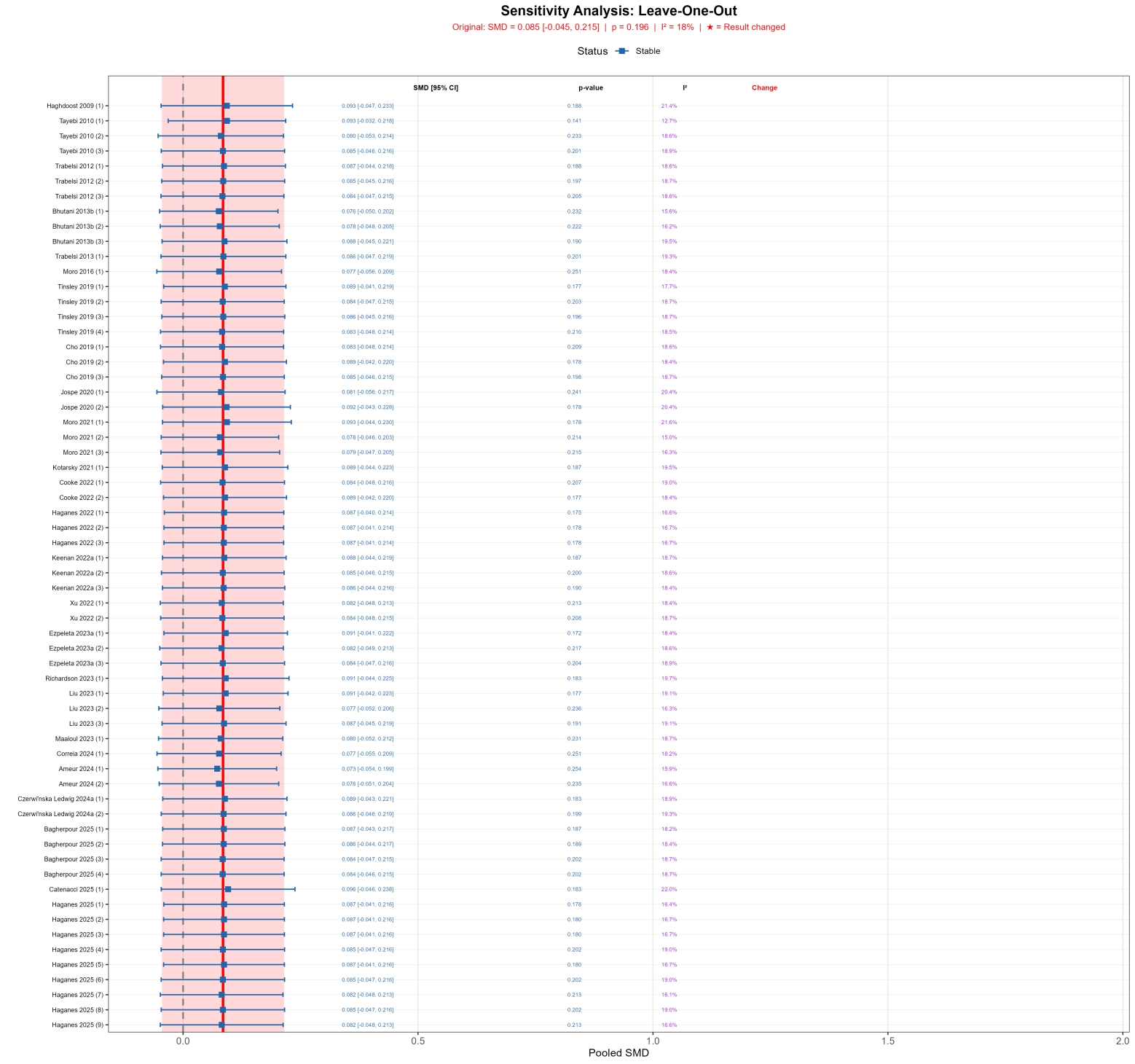


**High density lipoprotein**

**Supplementary Fig.6K (A sensitivity analysis based on leave-one-out)**


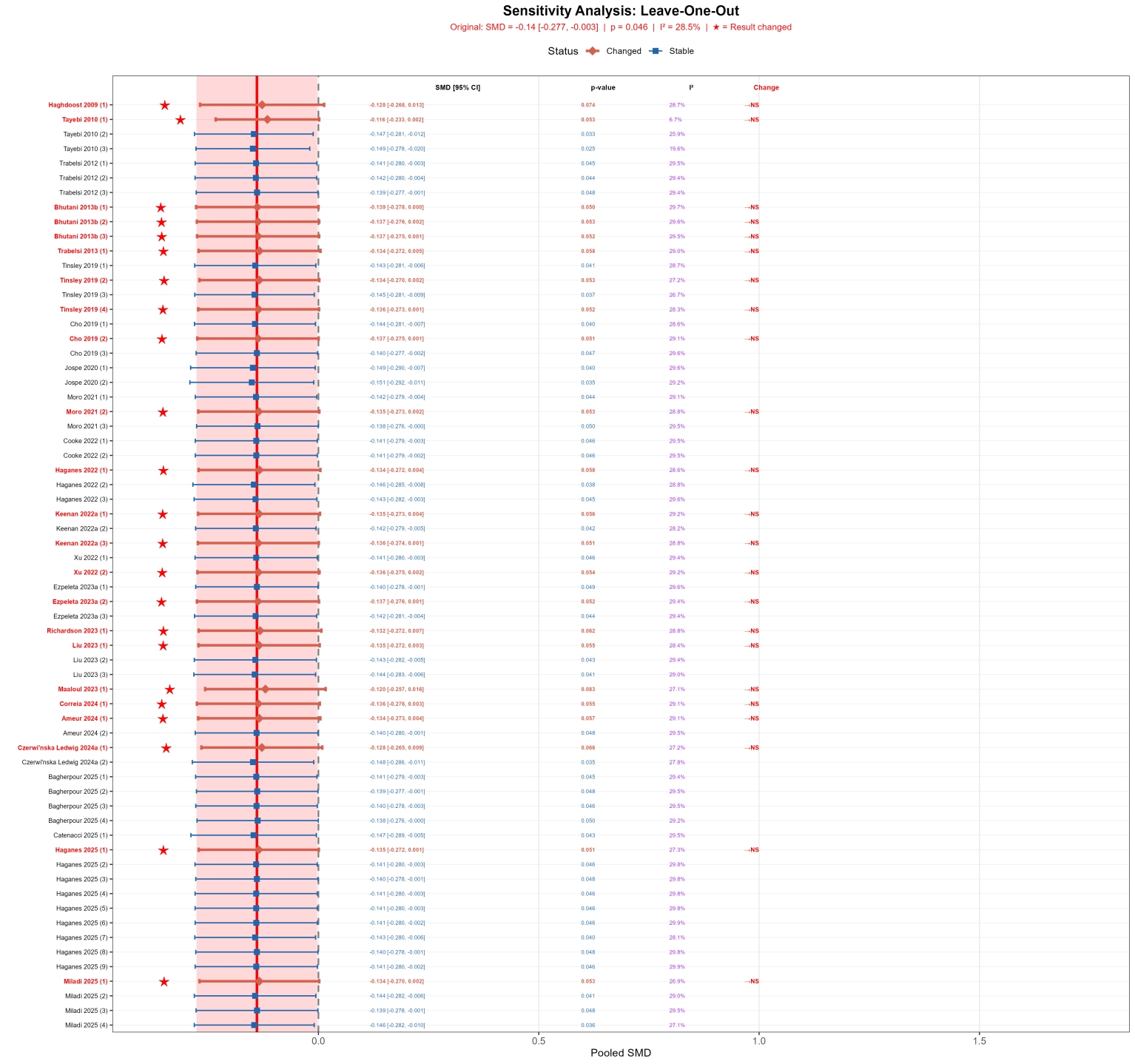


**Low density lipoprotein**

**Supplementary Fig.6L (A sensitivity analysis based on leave-one-out)**


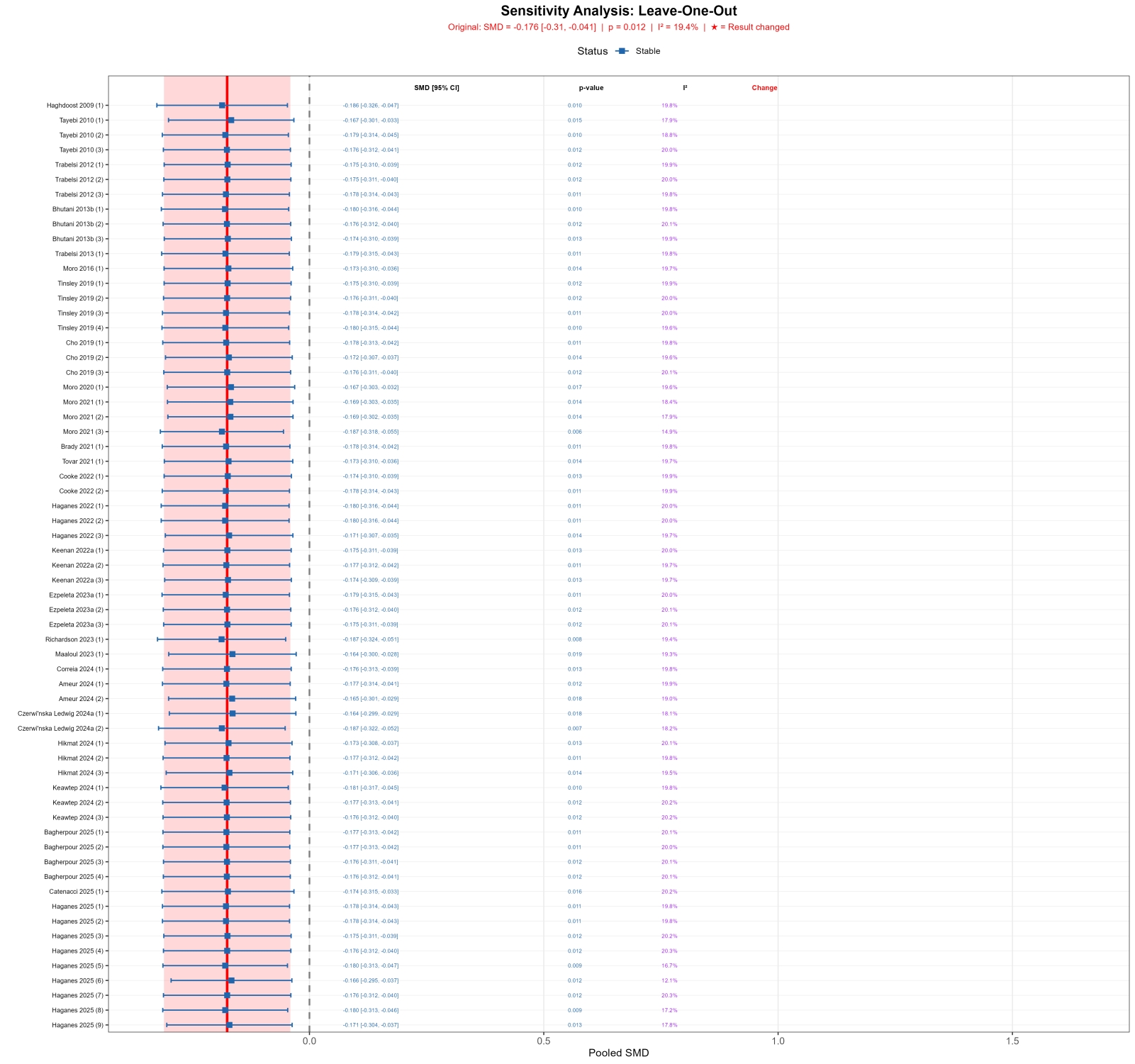


**Fasting glucose**

**Supplementary Fig.6M (A sensitivity analysis based on leave-one-out)**


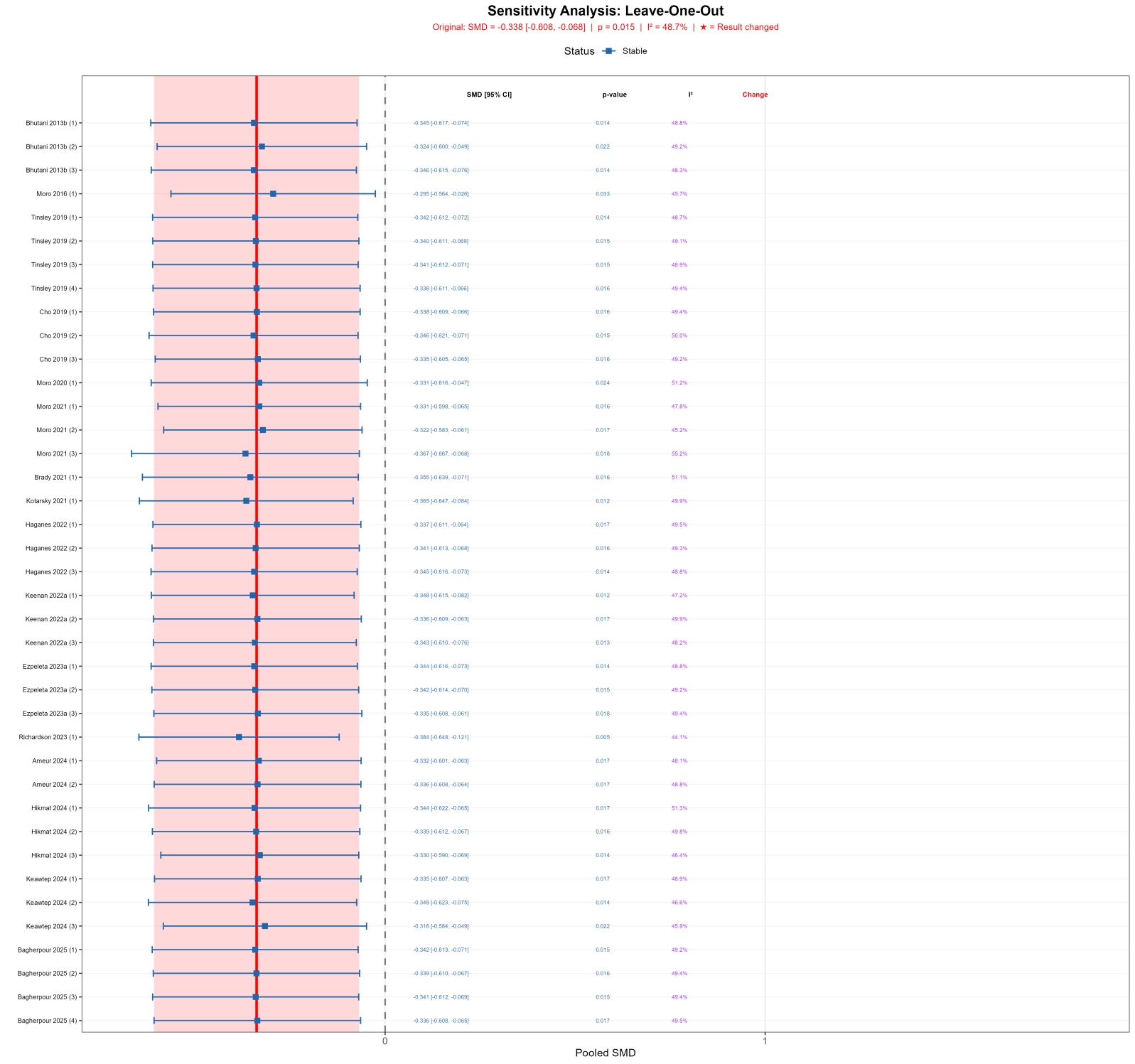


**Insulin**

**Supplementary Fig.6N (A sensitivity analysis based on leave-one-out)**


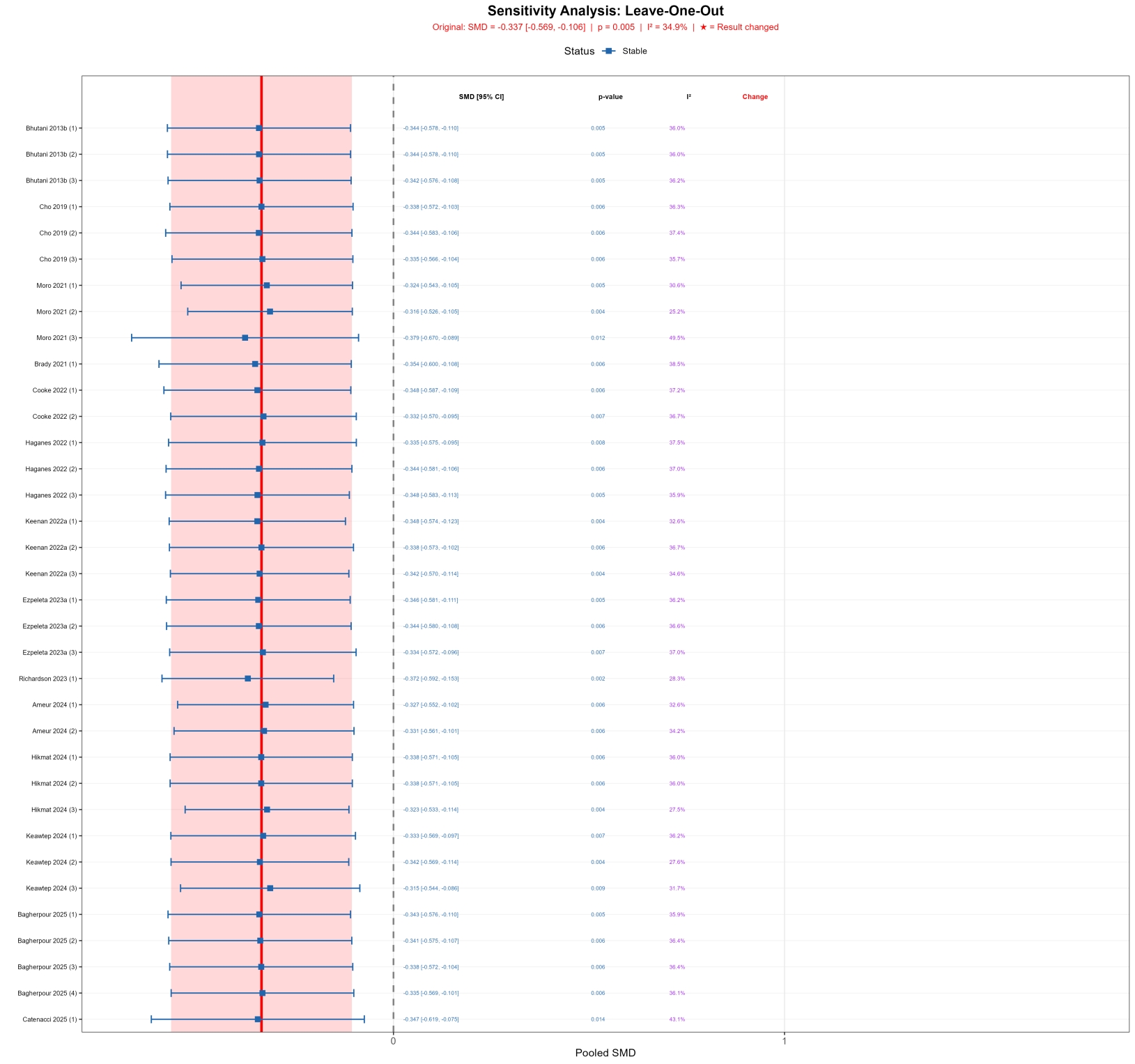


**HOMA-IR**

**Supplementary Fig.6O (A sensitivity analysis based on leave-one-out)**


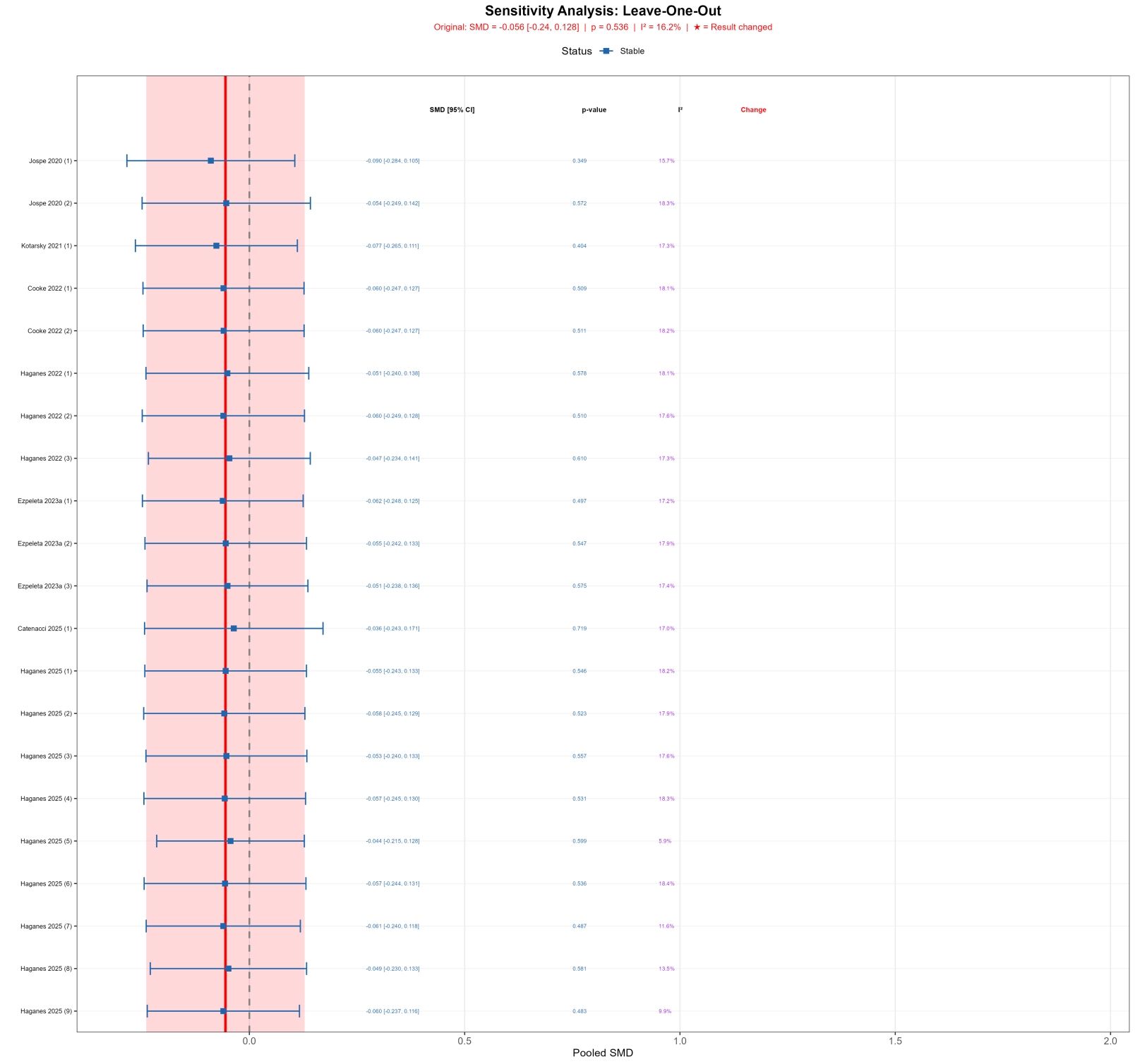


**HbA1c**

**Supplementary Fig.6P (A sensitivity analysis based on leave-one-out)**


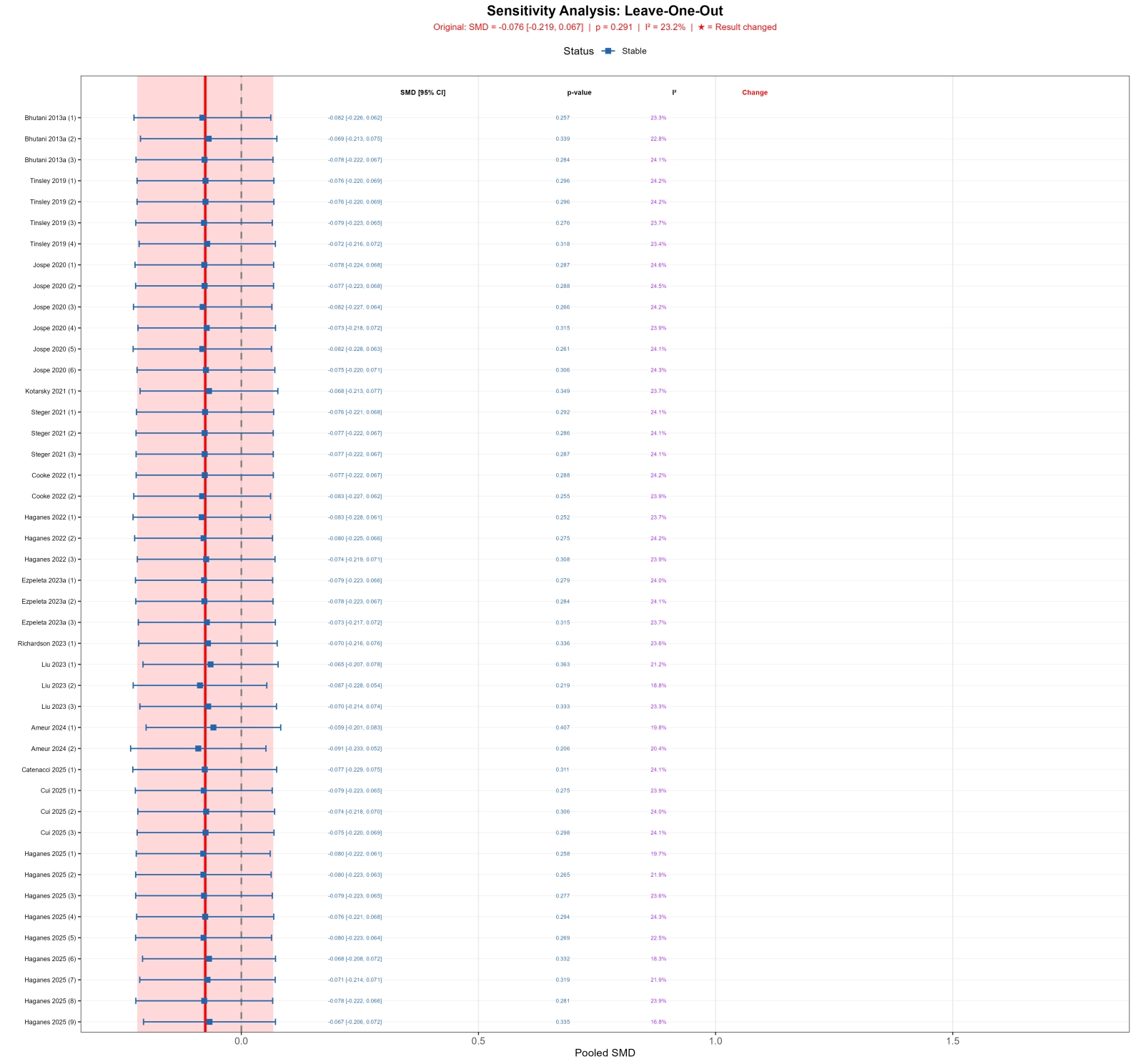


**Systolic blood pressure**

**Supplementary Fig.6Q (A sensitivity analysis based on leave-one-out)**


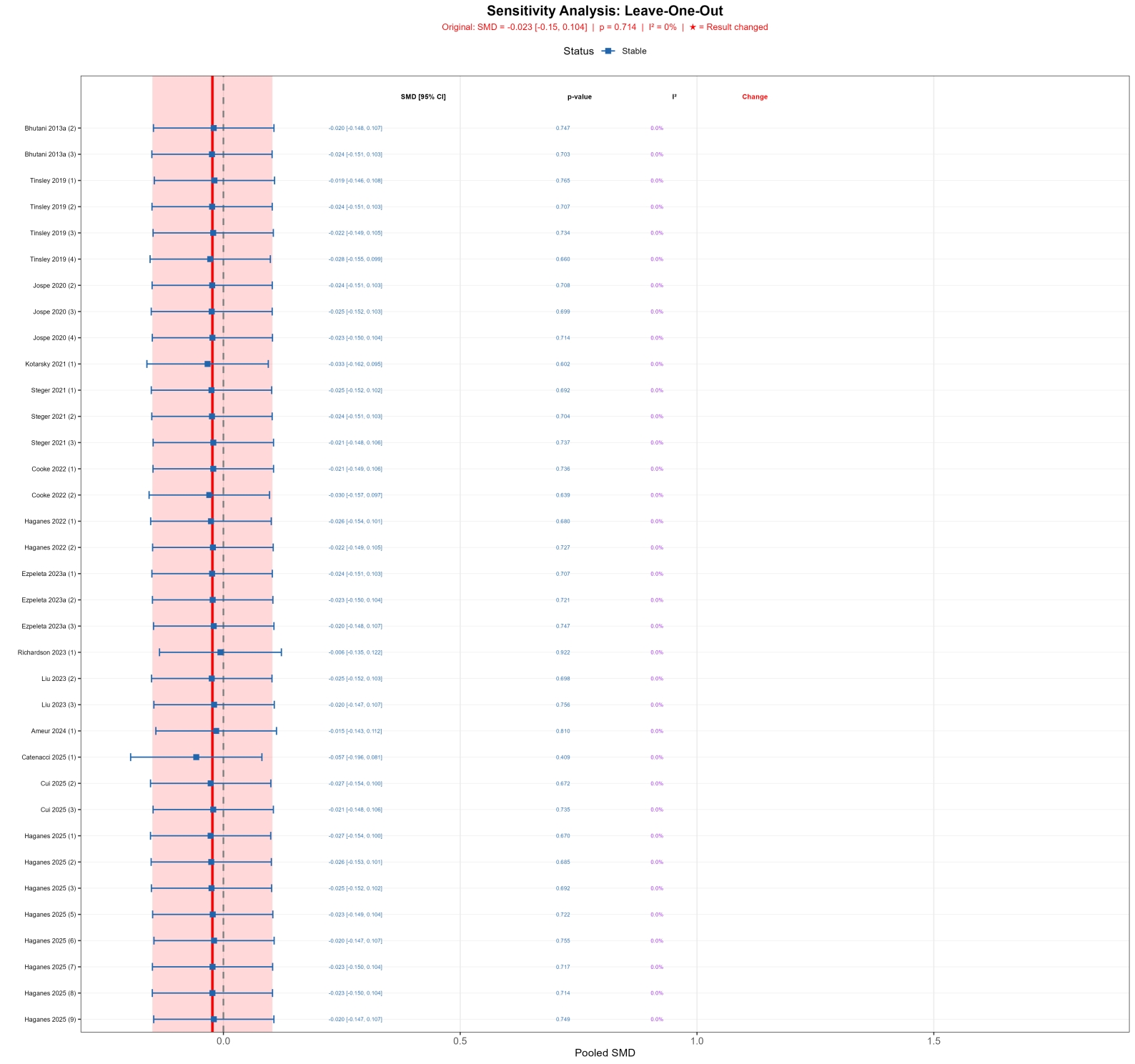


**Diastolic blood pressure**

**Supplementary Fig.6R (A sensitivity analysis based on leave-one-out)**


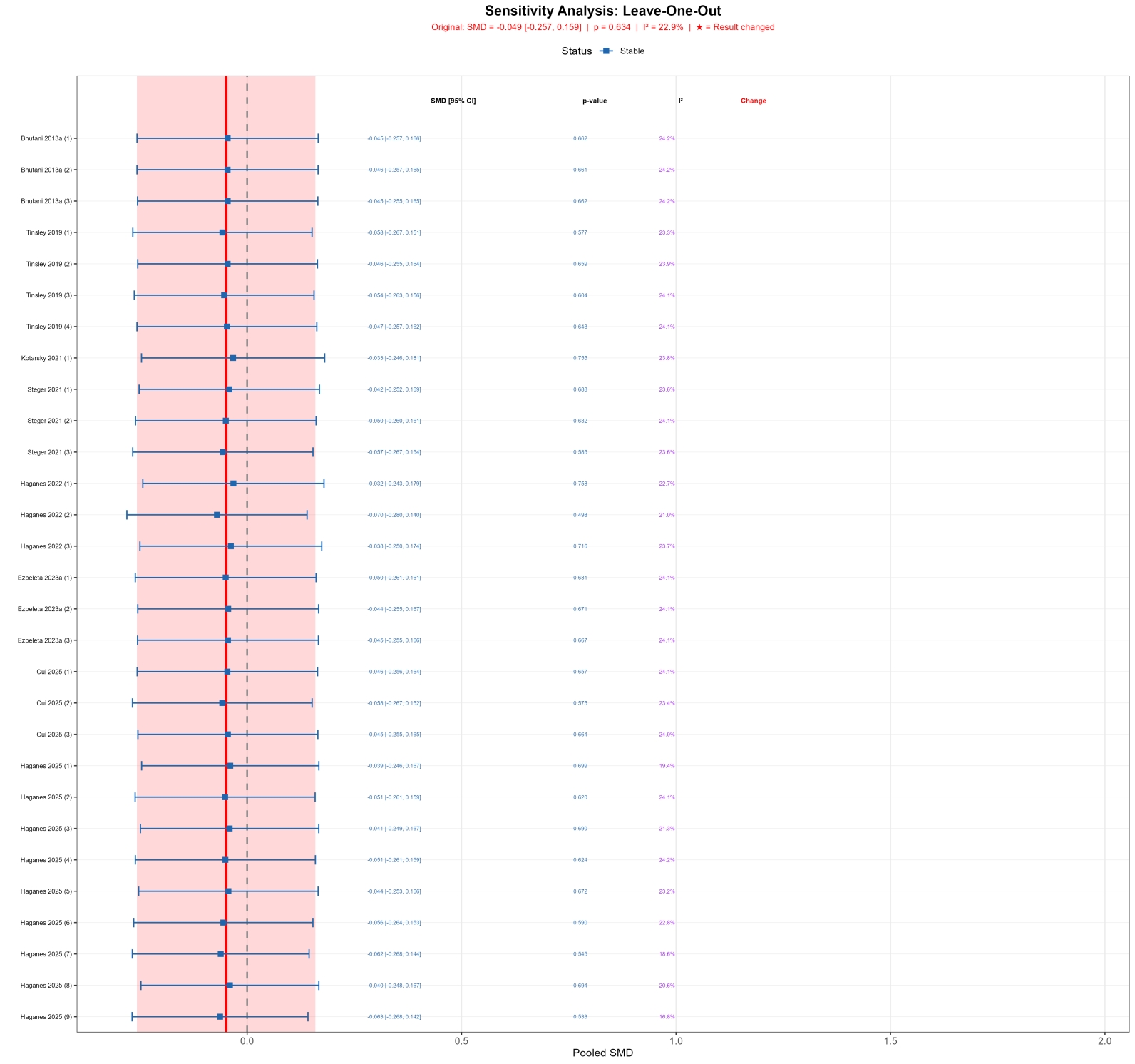


**Heart rate**

**Supplementary Fig.6S (A sensitivity analysis based on leave-one-out)**


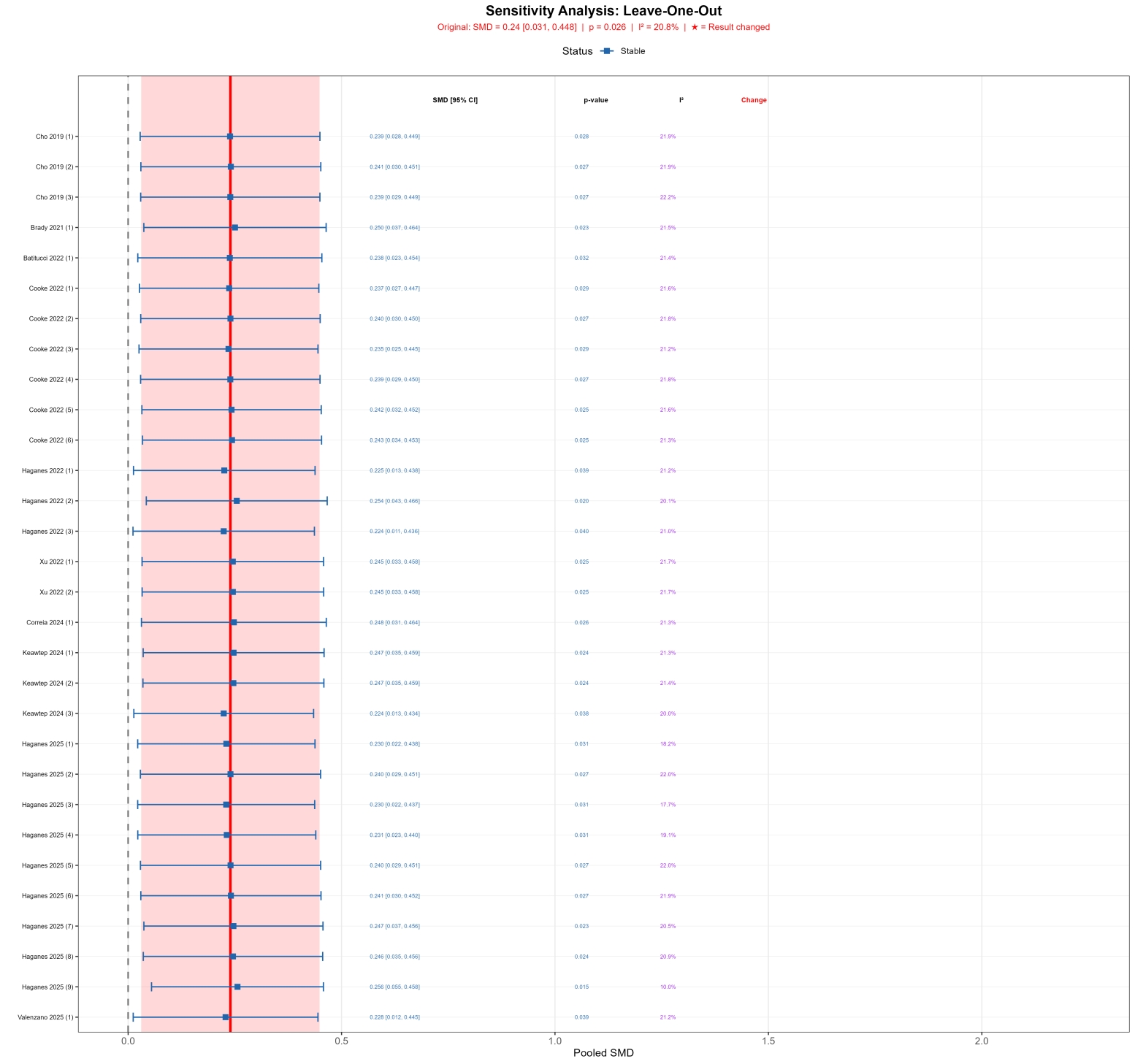


**VO_2max_**

**Supplementary Fig.6T (A sensitivity analysis based on leave-one-out)**


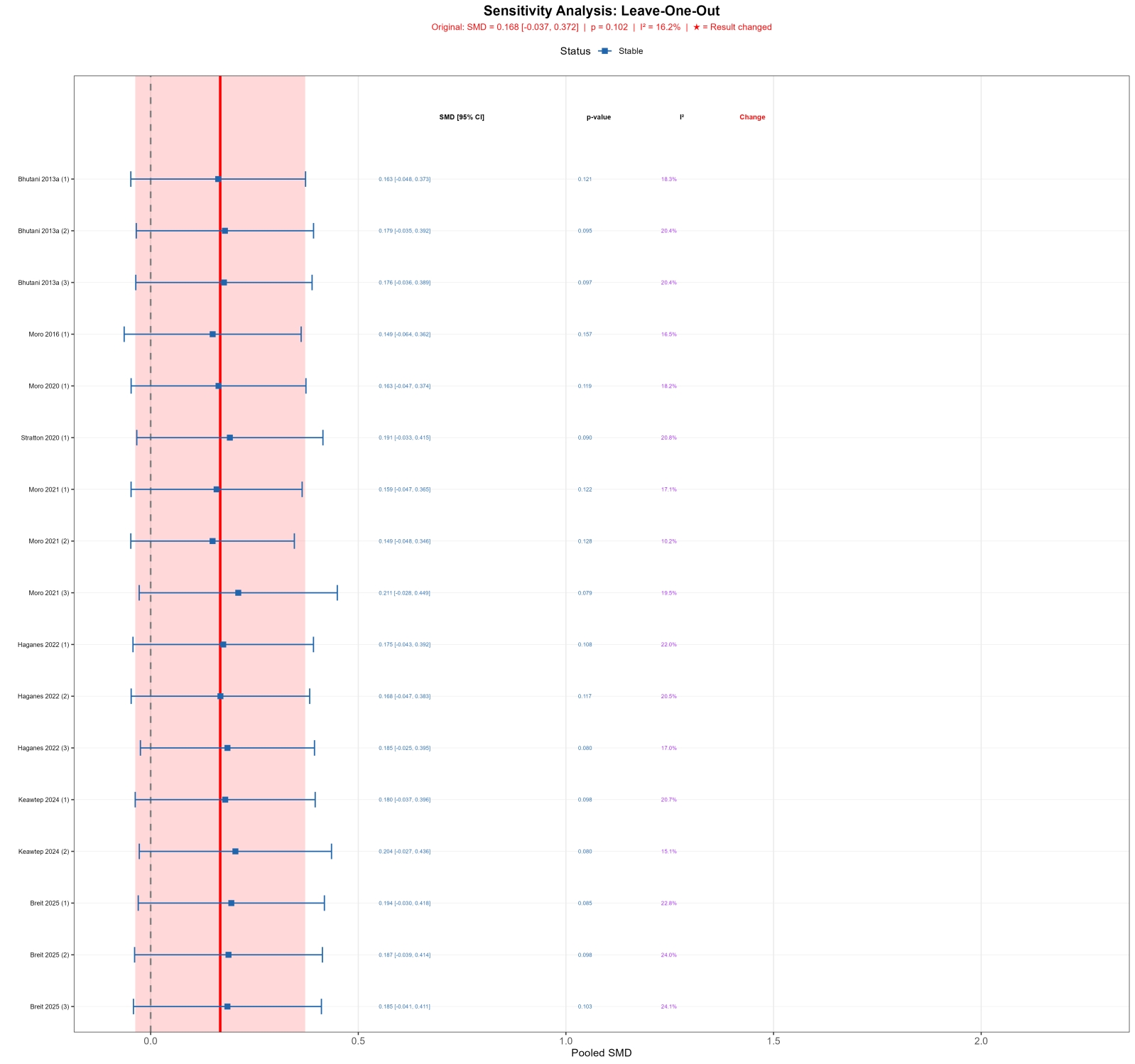


**Adiponectin**

**Supplementary Fig.6U (A sensitivity analysis based on leave-one-out)**


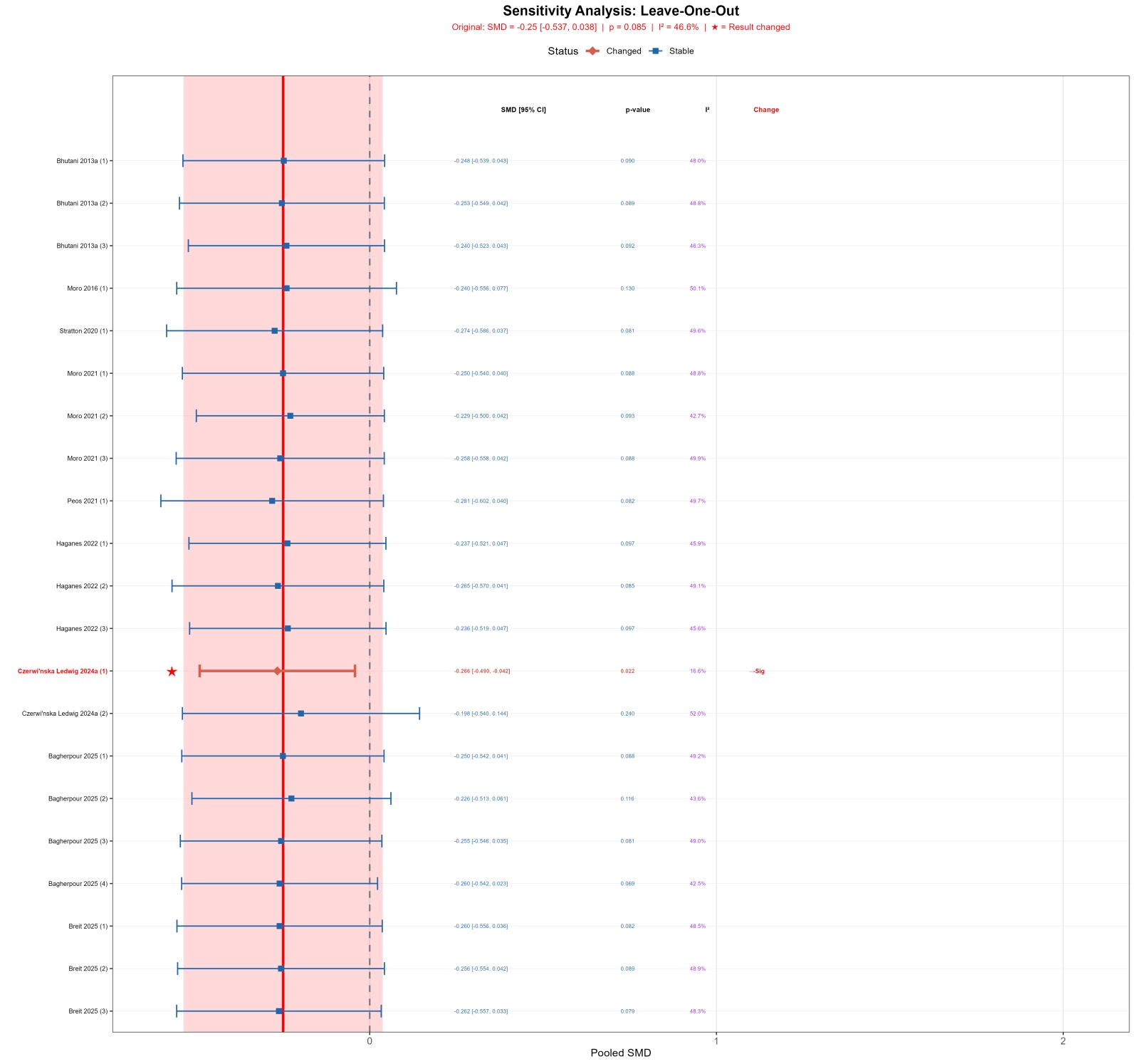


**Leptin**

**Supplementary Fig.6V (A sensitivity analysis based on leave-one-out)**


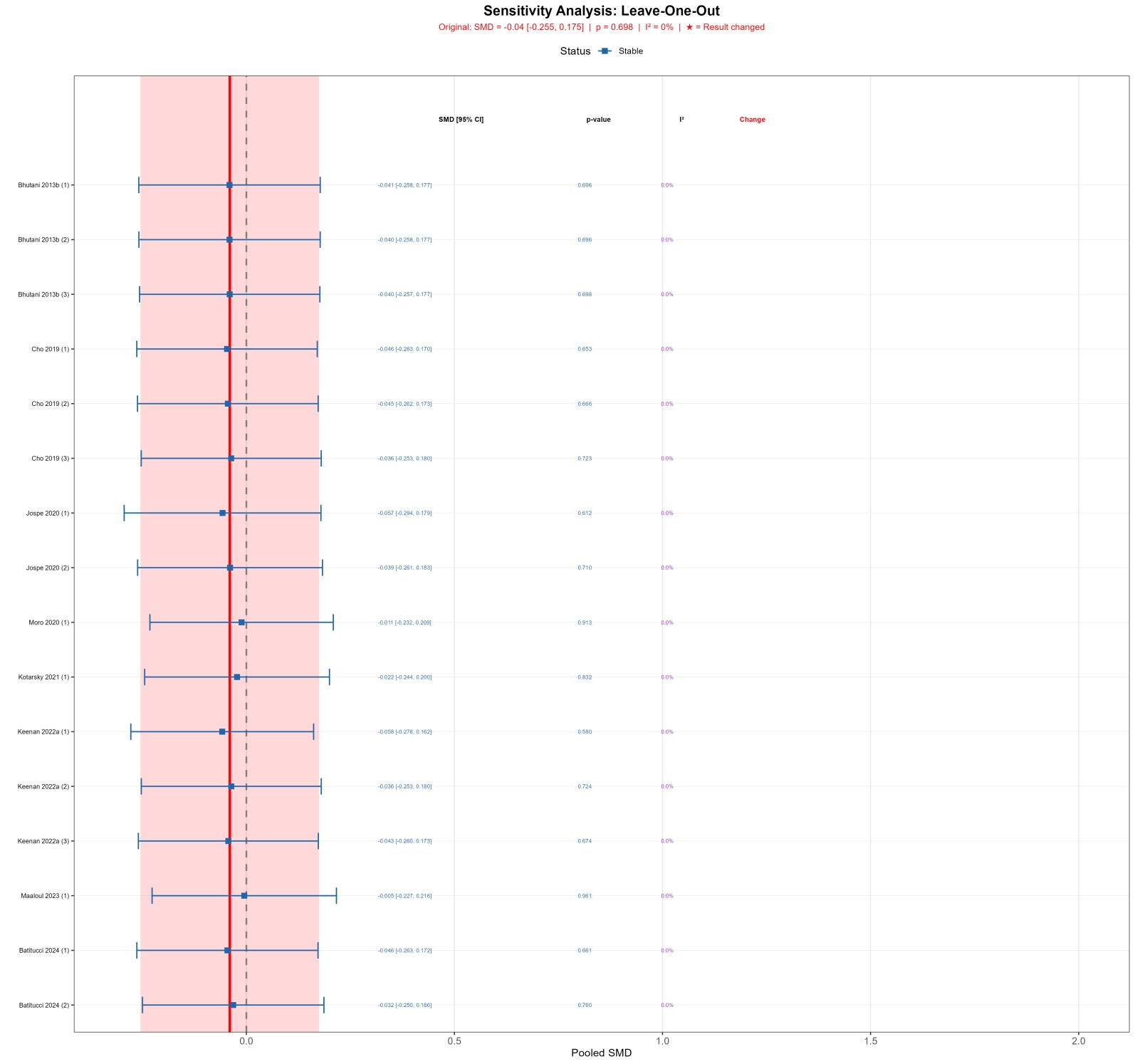


**CRP**

**Supplementary Fig.6W (A sensitivity analysis based on leave-one-out)**


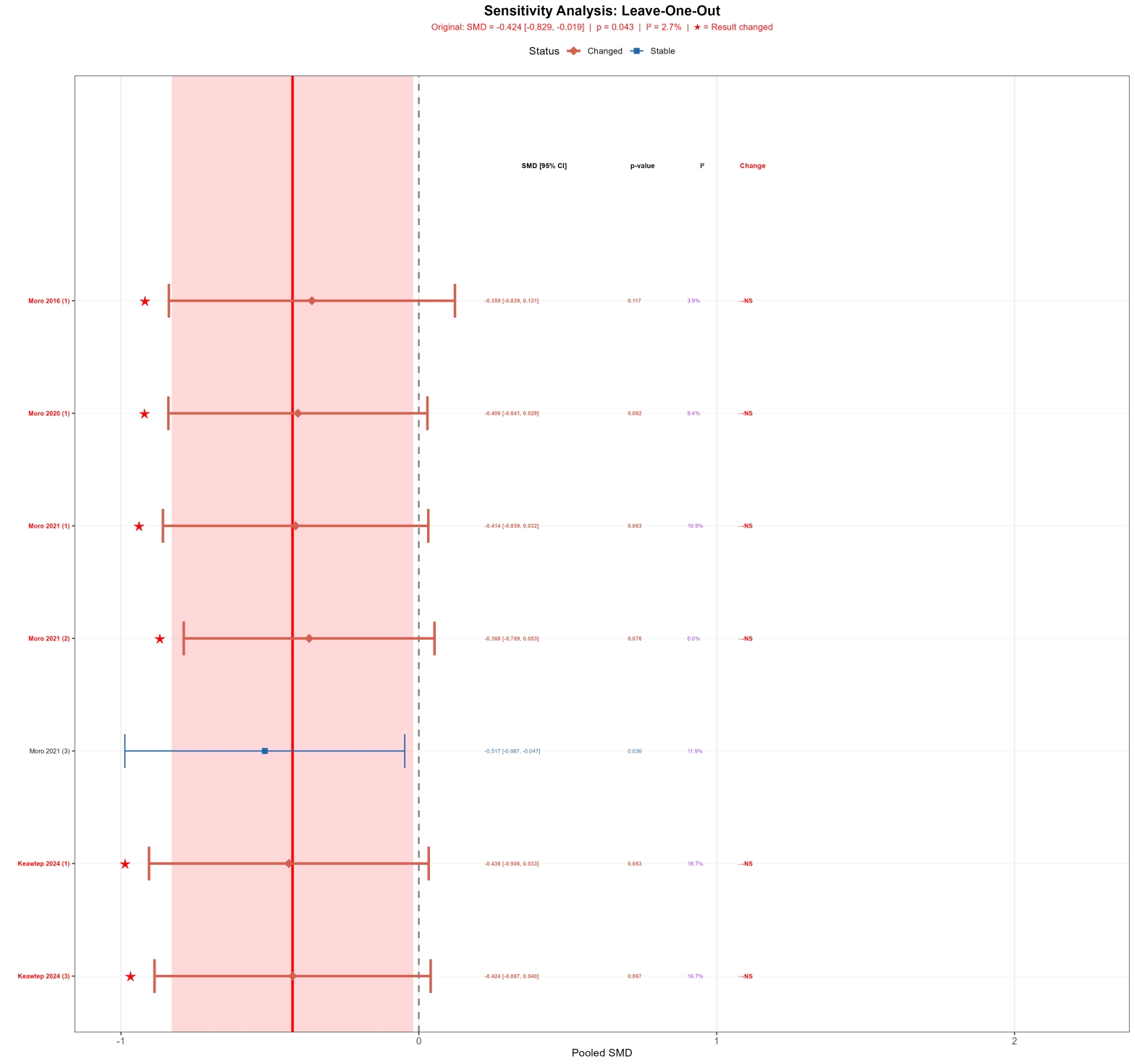


**IL-6**

**Supplementary Fig.6X (A sensitivity analysis based on leave-one-out)**


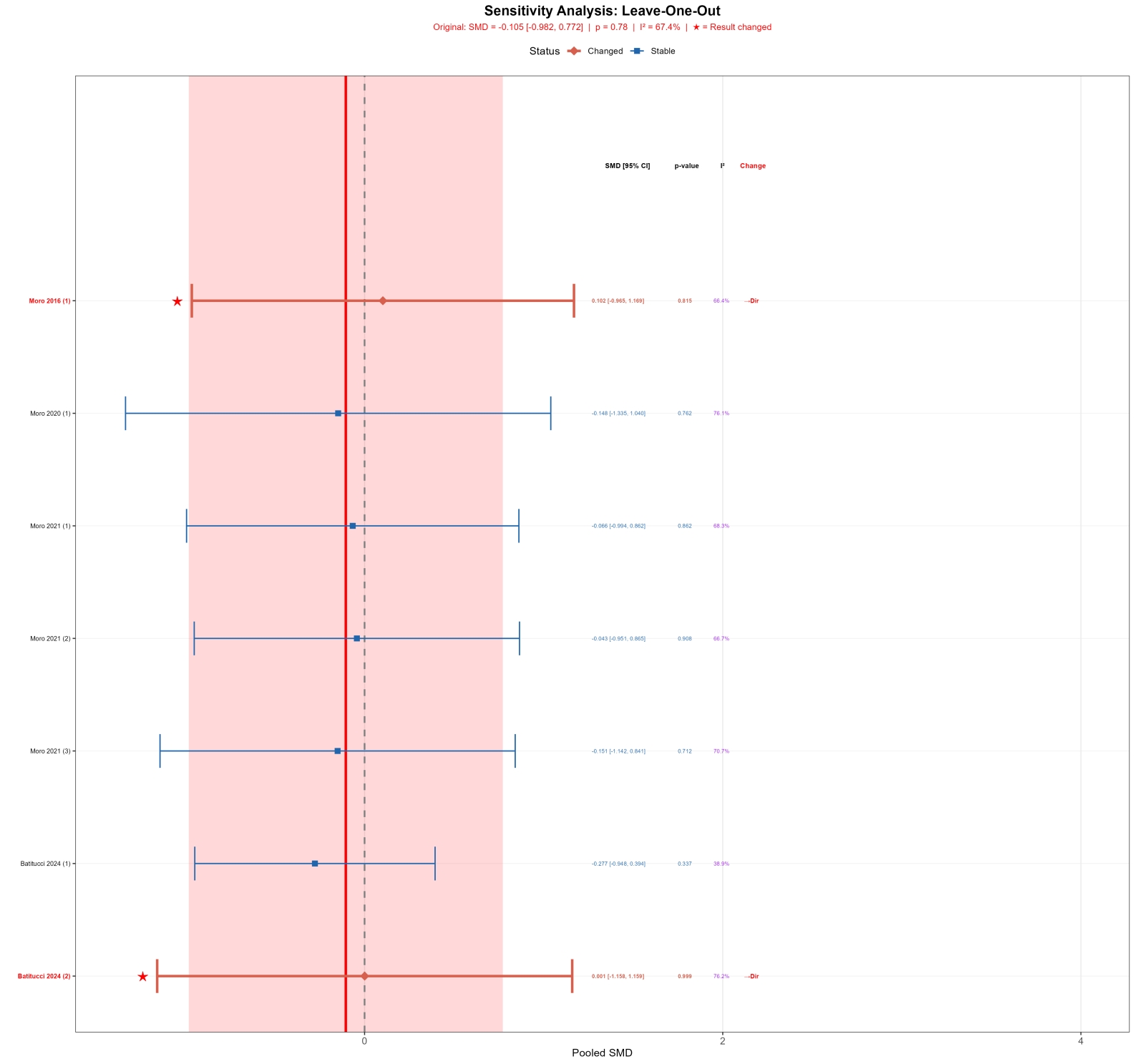


**TNF-α**

**Supplementary Fig.6Y (A sensitivity analysis based on leave-one-out)**


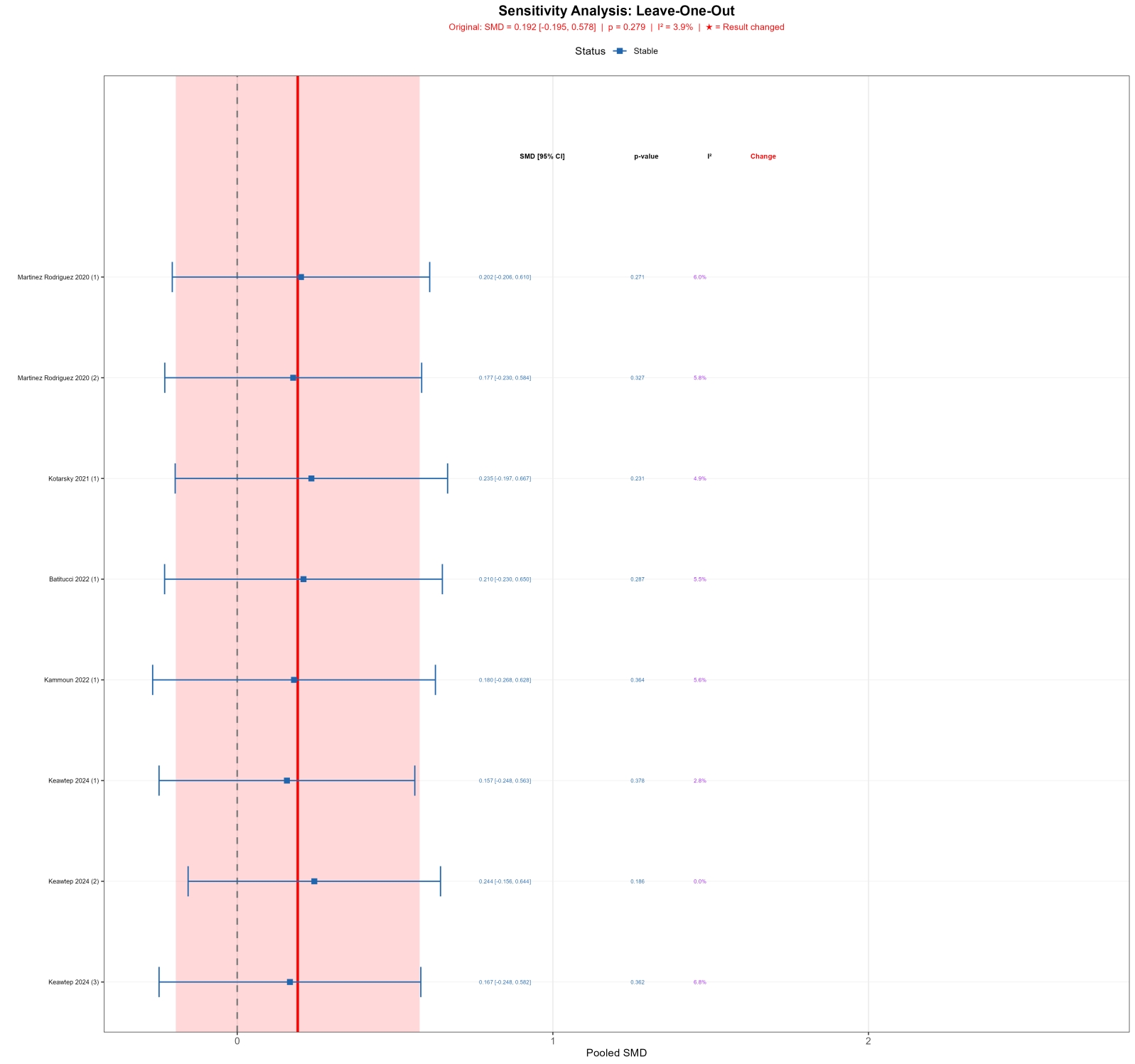


**Hand grip**

**Supplementary Fig.6Z (A sensitivity analysis based on leave-one-out)**


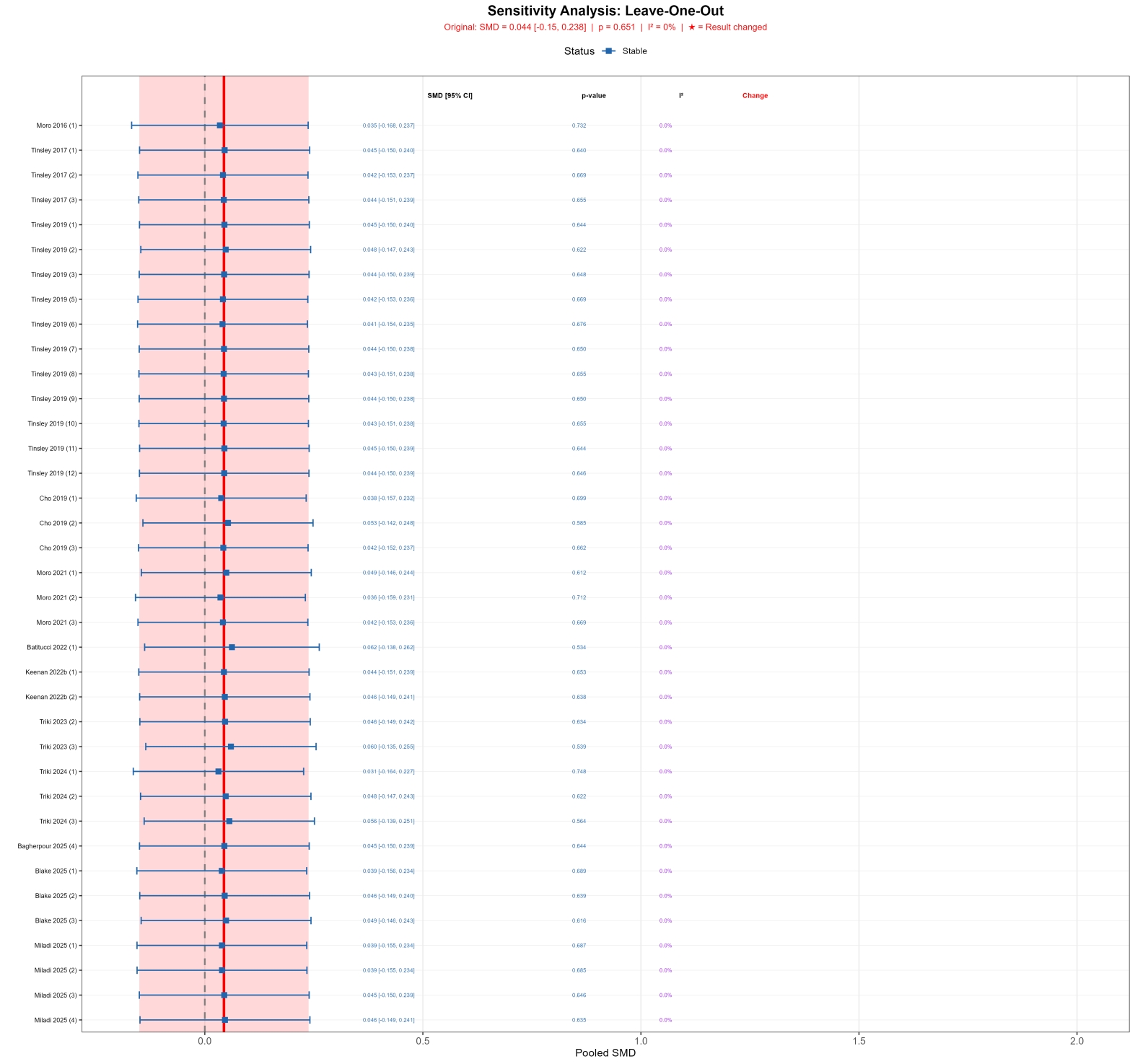


**Bench press**

**Supplementary Fig.6AA (A sensitivity analysis based on leave-one-out)**


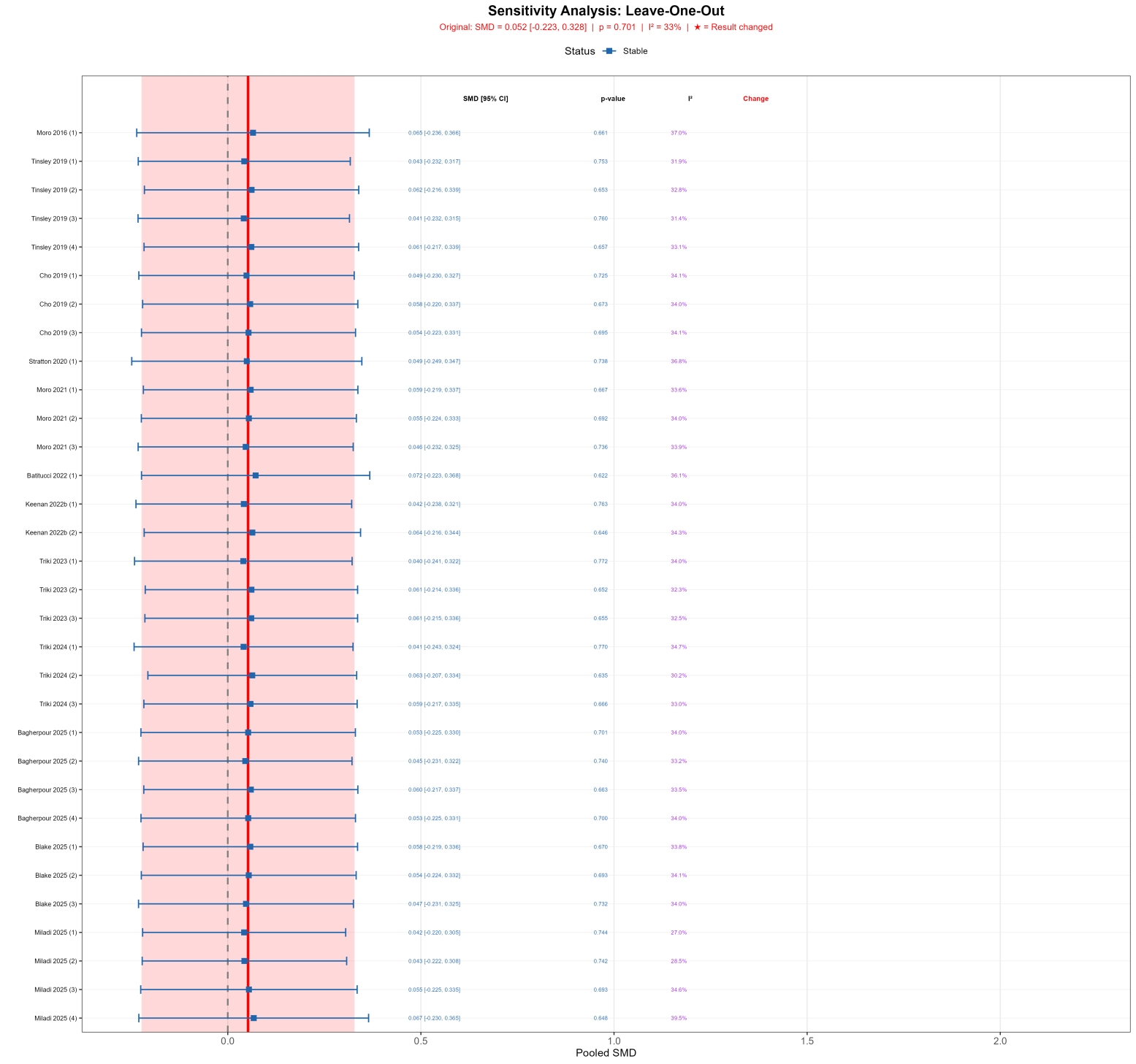


**Leg press**

**Supplementary Fig.6AB (A sensitivity analysis based on leave-one-out)**


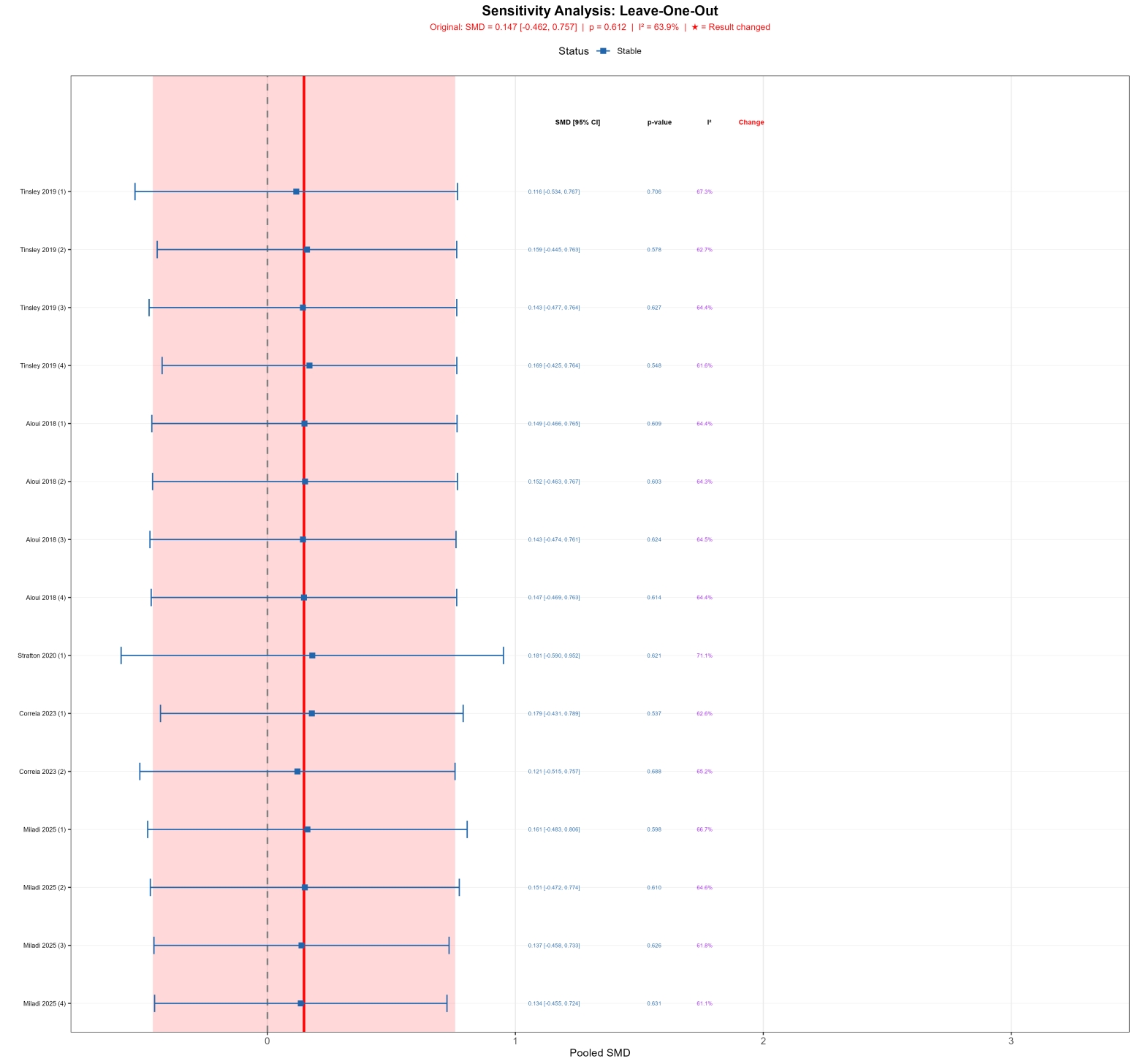


**Jump hight**

**Supplementary Fig.6AC (A sensitivity analysis based on leave-one-out)**

**
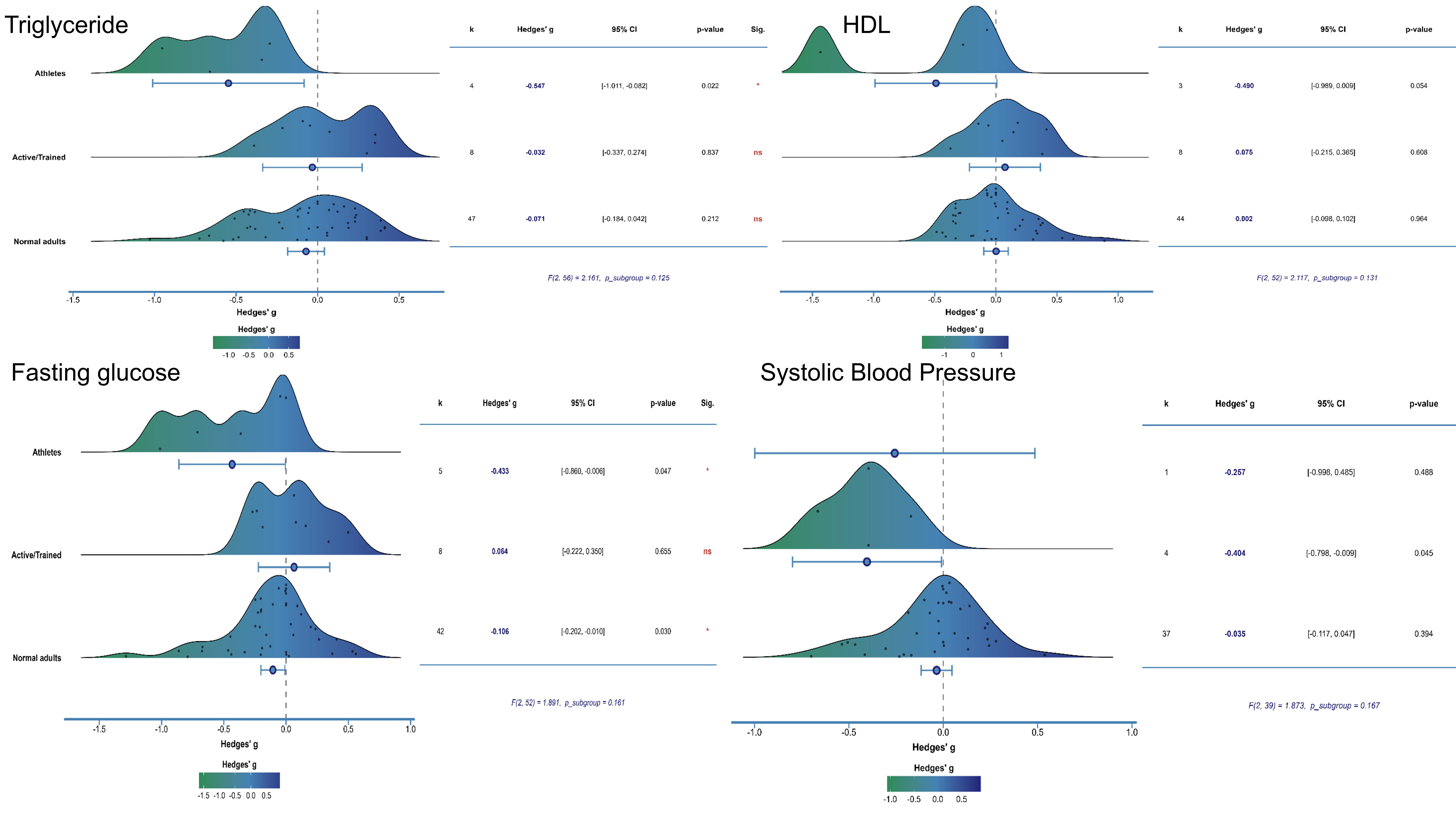
**

**Supplementary Fig.7 (Subgroup Analysis: Athletes, Active/Trained, and Normal Adults)**
